# Genome-Wide Association Study and Pathway-Level Analysis of Tocochromanol Levels in Maize Grain

**DOI:** 10.1534/g3.113.006148

**Published:** 2013-08-01

**Authors:** Alexander E. Lipka, Michael A. Gore, Maria Magallanes-Lundback, Alex Mesberg, Haining Lin, Tyler Tiede, Charles Chen, C. Robin Buell, Edward S. Buckler, Torbert Rocheford, Dean DellaPenna

**Affiliations:** * United States Department of Agriculture–Agricultural Research Service (USDA-ARS), Robert W. Holley Center for Agriculture and Health, Ithaca, New York 14853; † United States Department of Agriculture–Agricultural Research Service (USDA-ARS), U.S. Arid Land Agricultural Research Center, Maricopa, Arizona 85138; ‡ Department of Biochemistry and Molecular Biology, Michigan State University, East Lansing, Michigan, 48824; § Department of Plant Biology, Michigan State University, East Lansing, Michigan 48824; ** Department of Agronomy, Purdue University, West Lafayette, Indiana 47907; †† Institute for Genomic Diversity, Cornell University, Ithaca, New York 14853; ‡‡ Department of Plant Breeding and Genetics, Cornell University, Ithaca, New York 14853

**Keywords:** GWAS, candidate gene, vitamin E, biofortification

## Abstract

Tocopherols and tocotrienols, collectively known as tocochromanols, are the major lipid-soluble antioxidants in maize (*Zea mays* L.) grain. Given that individual tocochromanols differ in their degree of vitamin E activity, variation for tocochromanol composition and content in grain from among diverse maize inbred lines has important nutritional and health implications for enhancing the vitamin E and antioxidant contents of maize-derived foods through plant breeding. Toward this end, we conducted a genome-wide association study of six tocochromanol compounds and 14 of their sums, ratios, and proportions with a 281 maize inbred association panel that was genotyped for 591,822 SNP markers. In addition to providing further insight into the association between *ZmVTE4* (γ-tocopherol methyltransferase) haplotypes and α-tocopherol content, we also detected a novel association between *ZmVTE1* (tocopherol cyclase) and tocotrienol composition. In a pathway-level analysis, we assessed the genetic contribution of 60 *a priori* candidate genes encoding the core tocochromanol pathway (*VTE* genes) and reactions for pathways supplying the isoprenoid tail and aromatic head group of tocochromanols. This analysis identified two additional genes, *ZmHGGT1* (homogentisate geranylgeranyltransferase) and one prephenate dehydratase parolog (of four in the genome) that also modestly contribute to tocotrienol variation in the panel. Collectively, our results provide the most favorable *ZmVTE4* haplotype and suggest three new gene targets for increasing vitamin E and antioxidant levels through marker-assisted selection.

Tocochromanols are a class of lipid-soluble antioxidants synthesized by photosynthetic organisms and include the biosynthetically related compounds tocopherols and tocotrienols. Tocopherols have a saturated tail derived from phytol diphosphate, whereas the tail of tocotrienols is derived from geranylgeranyl diphosphate and contains three unconjugated double bonds. α-, β-, δ-, and γ-Tocopherols and tocotrienols refer to four species of compounds distinguished by the degree and position of methyl groups (three, two, two, and one methylations, respectively) on the aromatic ring of each compound class (DellaPenna and Mène-Saffrané 2011). A given tocopherol species generally has greater vitamin E activity than the corresponding tocotrienol species, with vitamin E activity for both classes of compounds following the order α > β >> γ > δ, and α-tocopherol (αT) having the greatest vitamin E activity on a molar basis (DellaPenna and Mène-Saffrané 2011). Although corresponding tocopherol and tocotrienol species have highly similar structures, tocotrienols show somewhat greater antioxidant capacity in several model membrane studies (Packer *et al.* 2001; Serbinova and Packer 1994; Suzuki *et al.* 1993).

Like all essential nutrients, vitamin E is required at minimal levels in the human diet to maintain optimal health. Clinical vitamin E deficiency is rare and results in various neurological conditions, including ataxia (impaired balance and coordination) and myopathy (muscle weakness). Suboptimal dietary intake has been associated with increased risk to cardiovascular disease, some cancers, and decreased immune function (Kushi *et al.* 1996; Wright *et al.* 2006), but large-scale vitamin E (as αT) intervention trials in healthy populations have been inconclusive on these roles (Traber *et al.* 2008). However, increased tocotrienol consumption has been associated with reduced serum cholesterol levels (Theriault *et al.* 1999; Wang *et al.* 1993). In the beef and pork industries, elevated tocochromanol levels in livestock feed results in meat with longer storage life, more desirable appearance, and greater consumer preference (Liu *et al.* 1995; Monahan *et al.* 1993; Sanders *et al.* 1997), presumably by inhibiting lipid peroxidation in these products. Apart from livestock feed, the shelf-life of vegetable oils and lipid-rich foods is also impacted by both the content and composition of tocochromanols present (Kamal-Eldin and Appelqvist 1996).

Maize (*Zea mays* L.) is not only a global staple crop but also a primary food source for hundreds of millions of people in developing countries. Unfortunately, the maize varieties providing grain for human consumption typically do not provide adequate daily levels of vitamin E (Fitzpatrick *et al.* 2012). In a survey of the U.S. population, 26–41% of individuals (depending on ethnicity) had blood plasma αT concentrations at levels that were associated with an increased risk for cardiovascular disease (Ford and Sowell 1999). Although studies of vitamin E sufficiency in developing countries are less widespread and comprehensive, it has been reported that dietary intake and plasma levels of αT were even lower than that in developed countries (Fares *et al.* 2011; Monteiro *et al.* 2009; Oldewage-Theron *et al.* 2010). Given the substantial variation for grain levels of tocopherols (Chander *et al.* 2008; Li *et al.* 2012; Wong *et al.* 2003) and tocotrienols (Weber 1987) among diverse maize lines, it should be possible to exploit this genetic diversity in marker-assisted selection programs that target specific endogenous tocochromanols in maize grain. Such a cost-effective and sustainable approach, termed biofortification (Bouis and Welch 2010), could make adequate levels of vitamin E available to people in developing countries that rely on maize grain as a staple, as well as for developed countries, for which seed oil is a primary source of vitamin E (Maras *et al.* 2004).

The tocochromanol biosynthetic pathway has been fully elucidated by approaches combining genetics and genomics and is highly conserved across plant species (reviewed in DellaPenna and Mene-Saffrane 2011). The committed step in synthesis of the aromatic head group for all tocochromanols is the conversion of *p*-hydroxyphenylpyruvate, from the shikimic acid pathway, to homogentistic acid (HGA) by the cytosolic enzyme *p*-hydroxyphenylpyruvic acid dioxygenase (HPPD; Figure 1). In the plastid, HGA is condensed with either phytol diphosphate by homogentisate phytyltransferase (encoded by the *VTE2* locus) or geranylgeranyl diphosphate by homogentisate geranylgeranyltransferase (HGGT) to yield the committed precursors to tocopherols and tocotrienols, respectively. Three subsequent activities, two methyltransferases and tocopherol cyclase (encoded by the *VTE3*, *VTE4*, and *VTE1* loci, respectively), generate the full chemical diversity of tocochromanols (α-, β-, δ-, and γ- tocopherols and tocotrienols). An additional source of phytol for tocopherol synthesis is provided by phytol kinase (*VTE5*), which in green seeds recycles phytol from chlorophyll degradation into the tocopherol pathway (Valentin *et al.* 2006).

**Figure 1:**
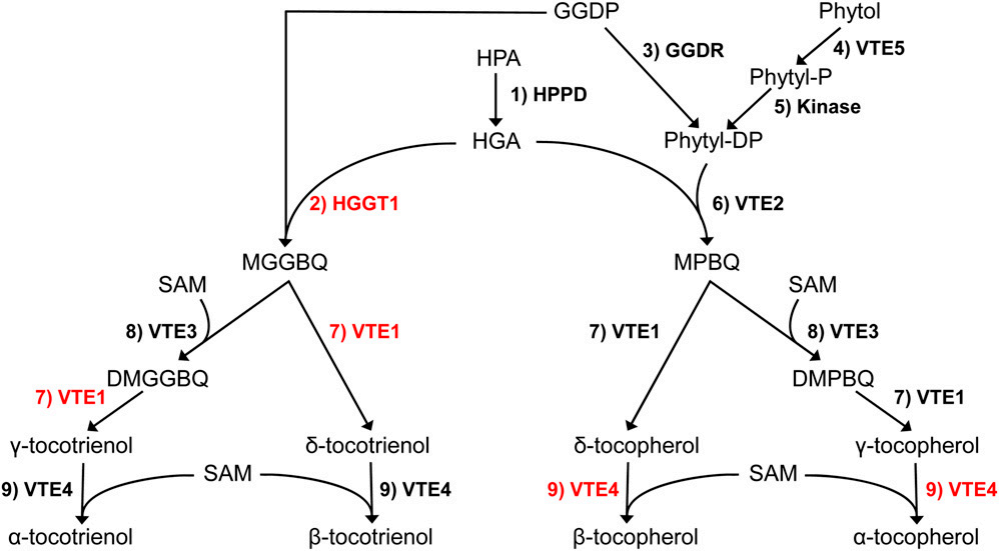
Tocochromanol biosynthetic pathway in maize grain. Enzymes in red correspond to genes that are within ±250 kb of the associated SNPs identified in our study. Compound abbreviations: HPA, *p*-hydroxyphenylpyruvic acid; HGA, homogentistic acid; GGDP, geranylgeranyl diphosphate; Pytyl-P, phytyl monophosphate; Phytyl-DP, phytyl diphosphate; MGGBQ, 2-methyl-6-geranylgeranylbenzoquinol; MPBQ, 2-methyl-6-phytylbenzoquinol; DMGGBQ, 2,3-dimethyl-5-geranylgeranylbenzoquinol; DMPBQ, 2,3-dimethyl-5-geranylgeranylbenzoquinol; SAM, S-adenosylmethionine. Reactions: 1) *p*-hydroxyphenylpyruvate dioxygenase (HPPD); 2) homogentisate geranylgeranyl transferase (HGGT1); 3) GGDP reductase (GGDR); 4) phytol kinase (VTE5); 5) unspecified kinase; 6) homogentisate phytyl transferase (VTE2); 7) tocopherol cyclase (VTE1); 8) MPBQ/MGGBQ methyl transferase (VTE3); and 9) γ-tocopherol methyl transferase (VTE4).

Since the first report of overexpressing *VTE4* to elevate αT levels in *Arabidopsis thaliana* seed (Shintani and DellaPenna 1998), many mutant and transgenic studies in a number of organisms have demonstrated roles for individual or combined *VTE* genes in regulating tocochromanol content and composition (Cahoon *et al.* 2003; DellaPenna and Mène-Saffrané 2011; Van Eenennaam *et al.* 2003). Linkage analysis studies of natural variation for tocopherol levels in seed of *Arabidopsis*, soybean, rapeseed, and sunflower have identified quantitative trait loci (QTL), including some that contain within their support intervals tocochromanol biosynthetic pathway genes, primarily *VTE3*, *VTE4*, and *HPPD* (Dwiyanti *et al.* 2011; Fritsche *et al.* 2012; Gilliland *et al.* 2006; Haddadi *et al.* 2012; Hass *et al.* 2006; Tang *et al.* 2006; Wang *et al.* 2012). In maize, *VTE4*, *HPPD*, and *VTE5* were found to reside within support intervals of QTL detected for variation of tocopherol levels in grain (Wong *et al.* 2003; Chander *et al.* 2008; Shutu *et al.* 2012). Recently, an association study identified polymorphisms within and ~85 kb upstream of *ZmVTE4* that independently associated with αT levels in maize grain (Li *et al.* 2012). In contrast to tocopherols, there have been few QTL studies for tocotrienol levels in crop species (Jackson *et al.* 2008; Sookwong *et al.* 2009).

The identification of key genes and favorable alleles associated with tocochromanol content and composition in maize grain could facilitate the molecular breeding of vitamin E and antioxidant-enriched maize. Association mapping in diverse maize germplasm with high-density single-nucleotide polymorphism (SNP) markers contributes to the process of resolving QTL down to single genes or causative variants. We conducted the present (1) genome-wide association study (GWAS) and (2) pathway-level analysis to identify genes responsible for quantitative variation of grain tocochromanol levels in a maize inbred association panel.

## Materials and Methods

### Germplasm

We evaluated an association panel of 281 diverse maize lines that captures a significant fraction of the common alleles present in temperate and tropical public maize breeding programs around the world (Flint-Garcia *et al.* 2005). This inbred association panel was grown in the summers of 2009 and 2010 at Purdue University’s Agronomy Center for Research and Education in West Lafayette, IN. The experimental field design has been previously described (Chandler *et al.* 2013). In brief, the association panel was arranged as a 14 × 20 incomplete block α-lattice design, and each incomplete block was augmented by the addition of two check entries (B73 and Mo17) at random positions. Experimental units consisted of a single line in a 3.05-m one-row plot. Each plot had an average of 10 plants. A single replicate of the complete experiment was grown in each of the two environments. At least four individual plants within a plot were self-pollinated by hand in 2009 and 2010. Harvested ears from each plot were shelled and bulked to form a representative, composite grain sample for measuring tocochromanol levels. Grain samples could not be obtained from all 281 lines, especially low yielding and late flowering lines and a total of 252 lines have phenotypic data.

### Tocochromanol extraction and quantification

Dry maize grain samples were ground using a commercial Stein Mill (Steinlite Corporation, Atchison, KS) and aliquoted into cryogenic tubes for long-term storage at −80°. For high-pressure liquid chromatography (HPLC) analysis, 15−20 mg of ground tissue was weighed into a 1.4 mL of U-bottom bar coded extraction tube (Micronic USA, Aston, PA) containing two 5-mm glass beads. Four hundred microliters of extraction buffer [60:40 v/v acetone: ethyl acetate containing 1 mg/mL butylated hydroxytoluene and 1 mg/mL DL-α-tocopherol acetate (Sigma-Aldrich, St Louis, MO) as an internal recovery control] and 150 μL of deionized water was added and extraction accomplished using a commercial paint shaker (HERO, BC, Canada) for 10 min. Samples were centrifuged for 10 min at 2500 × g in a Sorvall Legend RT centrifuge (Kendro Laboratory Products, Newtown, CT). Two-hundred microliters of the upper organic phase was then transferred into a 750-μL tube, dried in a speedvac and resuspended in 100 μL of 3:1 (v:v) methanol:methyl *tert*-butyl ether by shaking on a microplate shaker for 15 min at 2000 rpm. After centrifuging for 5 min at 2500 × g, the supernatant was transferred to a microtiter plate and 20 μL injected for HPLC analysis. Tocochromanols were resolved by chromatography at 30° on a 3-mm × 100-mm YMC C_30_ column with a 3-μm particle size (Waters, Milford, MA). Mobile phases were A: methanol:methyl *tert*-butyl ether and B: methanol:H2O (90:10, v:v) at a flow rate of 0.8 mL/min using the following gradient: 0 to 12 min: 100% B to 60% B; 12 to 17.5 min: 60% B to 22.5% B; 17.5 to 19.5 min: 22.5% B to 100% B; 19.5 to 21 min held at 100% B for re-equilibration. Tocochromanols were detected by fluorescence using 290 nm excitation and 325 nm emission and quantified relative to curves with γ-, α-, and δ-tocopherol (Matreya, LLC, Pleasant Gap, PA) and γ-, α-, and δ-tocotrienol standards (Cayman Chemical Company, Ann Arbor, MI).

Similar to other tocochromanol studies in maize (Chander *et al.* 2008; Li *et al.* 2012; Shutu *et al.* 2012; Wong *et al.* 2003), we did not quantify β-tocopherol and β-tocotrienol because they are minor components, whereas γ-tocopherol and γ-tocotrienol are major components of the maize tocochromanol profile (Panfili *et al.* 2003). β- and γ-tocopherols (and tocotrienols) have an identical structural formula and cannot be separated by reverse-phase HPLC. Normal phase HPLC can be used to resolve β- and γ-tocochromanol species, but reverse-phase HPLC is a more robust procedure for high-throughput analysis of low-level metabolites in large, diverse plant populations (Panfili *et al.* 2003).

### Phenotypic data analysis

We evaluated 20 tocochromanol traits based on HPLC data collected on grain samples from 252 lines of the maize association panel that yielded sufficient quantities of physiologically mature grain. Specifically, the phenotypes analyzed were α-tocopherol (αT), δ-tocopherol (δT), γ-tocopherol (γT), α-tocotrienol (αT3), δ-tocotrienol (δT3), γ-tocotrienol (γT3), total tocopherols (total T), total tocotrienols (total T3), total tocochromanols (total T3+T) in μg·g^−1^ seed, and the following compound ratios and proportions: αT/γT, αT3/γT3, γT/(γT+αT), γT3/(γT3+αT3), δT/(γT + αT), δT/αT, δT/γT, δT3/(γT3 + αT3), δT3/αT3, δT3/γT3, and total T/total T3. Levels of δT3 and δT were below minimum HPLC detection limits in five and three samples, respectively, and values were approximated for these samples by uniform random variables ranging from 0 to min, where min is the minimum HPLC detection value for a given compound. This approach is similar to one recommended by Lubin *et al.* (2004) for quantitative analysis of compounds above or below analytical chemistry detection limits. The 20 traits were screened for outliers in SAS version 9.3 (SAS Institute 2012) by examining the Studentized deleted residuals (Kutner *et al.* 2004) obtained from mixed linear models fitted with environment, set, block, and line as random effects.

For each trait, a best linear unbiased predictor (BLUP) for each line (Supporting Information, Table S1) was predicted from a mixed linear model fitted across environments in ASReml version 3.0 (Gilmour *et al.* 2009). The model fitting procedure has been previously described (Chandler *et al.* 2013). Variance component estimates from each final model were used to estimate heritability on a line mean basis (${\hat{h}}_{l}^{2}$; Holland *et al.*, 2003; Hung *et al.* 2012). Standard errors of the heritability estimates were approximated using the delta method (Holland *et al.* 2003). Pearson’s correlation coefficients (*r*) were used to assess the relationship between untransformed tocochromanol trait BLUPs. Before the GWAS, the Box-Cox procedure (Box and Cox 1964) was implemented in SAS version 9.3 (SAS Institute 2012) to find the most appropriate transformation that corrects for non-normality of the error terms and unequal variances. This procedure was undertaken to ensure that data adhered to the statistical assumptions made for the models fitted in the GWAS and pathway-level analysis.

### GWAS

The diversity panel was previously genotyped with the Illumina MaizeSNP50 BeadChip (Cook *et al.* 2012) and various other SNP genotyping assays (McMullen *et al.* 2009; Yu *et al.* 2006). The MaizeSNP50 genotypic data set for the maize association panel from Cook *et al.* (2012) is available for download from the Panzea database (http://www.panzea.org/genotype_search.html). In this study, the diversity panel was also genotyped with a genotyping-by-sequencing (GBS) protocol (Elshire *et al.* 2011) to further enhance genome-wide marker density. The GBS marker data set (partially imputed genotypes; January 10, 2012 version) is available for download from the Panzea database (http://www.panzea.org/dynamic/derivative_data/genotypes/Maize282_GBS_genos_imputed_20120110.zip). Removal of monomorphic and low-quality SNPs generated a data set with 591,822 SNPs, of which 294,092 SNPs had minor allele frequencies (MAFs) greater than or equal to 0.05 in the panel. Of the 294,092 SNPs used for association analysis, 7964 of them were redundant as a result of loci overlapping among the three SNP data sets. These redundant SNPs were included in the association analysis because of the variable missing data patterns and error rates attributable to the different genotyping technologies. Before association analysis, missing SNP genotypes were conservatively imputed with the major allele.

With BLUPs of each tocochromanol trait, we conducted a GWAS with 294,092 genome-wide SNPs using a univariate unified mixed linear model (Yu *et al.* 2006) that eliminated the need to recompute variance components (*i.e.*, population parameters previously determined, or P3D) (Zhang *et al.* 2010) in the Genome Association and Prediction Integrated Tool package (Lipka *et al.* 2012). To control for population structure and familial relatedness, the mixed model included principal components (Price *et al.* 2006) and a kinship (coancestry) matrix (Loiselle *et al.* 1995) that were calculated using the 34,368 nonindustry SNPs from the Illumina MaizeSNP50 BeadChip. For BLUPs of each tocochromanol trait, the Bayesian information criterion (Schwarz 1978) was used to determine the optimal number of principal components to include as covariates in the mixed model. A likelihood-ratio-based *R^2^* statistic, denoted *R^2^_LR_* (Sun *et al.* 2010), was used to assess the amount of phenotypic variation explained by the model. The Benjamini and Hochberg (1995) procedure was used to control for the multiple testing problem at false-discovery rates (FDRs) of 5% and 10%.

In addition to the unified mixed linear model, a multilocus mixed model (MLMM) (Segura *et al.* 2012) was implemented to clarify complex association signals that involved a major effect locus. The MLMM employs stepwise mixed-model regression with forward inclusion and backward elimination, thus allowing for a more exhaustive search of a large model space. In contrast to the unified mixed model with P3D, the MLMM re-estimates the variance components of the model at each step. Specifically, all SNPs on a chromosome (*i.e.*, chromosome-wide) with a major effect locus were considered for inclusion into the final model. The optimal model was selected using the extended Bayesian information criterion (Chen and Chen 2008). We then reconducted GWAS for each trait with MLMM-identified SNPs included as covariates in the unified mixed linear model for better control of major effect loci.

### Pathway-level analysis

We conducted a pathway-level analysis that used prior knowledge pertaining to the biosynthesis of tocochromanols to select a prioritized subset of candidate genes. The genes encoding enzymes leading to synthesis of the tocochromanol precursors HGA, GGDP, and phytol-PP and for the core tocochromanol pathway (*VTE1* through *VTE5*) have been well characterized in *Arabidopsis* (DellaPenna and Mène-Saffrané 2011) and *HGGT* in maize (Cahoon *et al.* 2003). These sequences were used to search the maize B73 RefGen_v2 genome assembly resulting in the identification of 60 *a priori* maize candidate genes that were grouped into three general categories: (1) aromatic head group synthesis (HGA production); (2) prenyl group synthesis; and (3) core tocochromanol pathway genes (the *VTE* genes and *HGGT*; Table S2). We then created a subset of GWAS results for each trait that included raw (unadjusted) *P*-values for the 6122 SNPs located ±250 kb of these 60 *a priori* genes. The *P*-values within each subset were corrected for multiple testing by using the Benjamini and Hochberg (1995) procedure to control the FDR at 5%.

### Linkage disequilibrium (LD) analysis

LD between pairs of SNPs was estimated by using squared allele-frequency correlations (*r*^2^) in TASSEL version 3.0 (Bradbury *et al.* 2007). Local LD (*r*^2^) and common haplotype patterns were also assessed in Haploview version 4.2 (Barrett *et al.* 2005). Haplotype blocks were defined with the confidence interval method of Gabriel *et al.* (2002). Only SNPs with a MAF ≥0.05 and less than 0.10 missing data were used to estimate LD. In contrast to the GWAS, missing SNP genotypes were not imputed with the major allele before LD analysis.

## Results

### Phenotypic variability

The extent of phenotypic variation for tocochromanol content in maize grain was assessed with the use of an inbred association panel of 252 diverse maize lines. For the six tocochromanol compounds quantified by HPLC, the most abundant was γT, whereas the least abundant was δT3 (Table 1). For both tocopherols and tocotrienols, the γ-species were the most abundant, followed by α- and δ-species. For the γ- and δ-species, the average amount of tocopherols was substantially greater than their tocotrienol counterpart and the respective tocopherol and tocotrienol species only showed very weak Pearson’s correlations (*r* = 0.14 and 0.16, respectively, Table S3). Even though the average amount of α-tocopherol was greater than its tocotrienol complement, the correlation between these two species was stronger (*r* = 0.40). Total tocopherols and tocotrienols were also very weakly correlated (*r* = 0.10) although, as expected, both were strongly correlated with total tocochromanols (*r* = 0.84 and 0.61, respectively). This suggests that synthesis of the tocopherol and tocotrienol compound classes are largely independently regulated. The average heritability on a line-mean basis for the six tocochromanol compounds and their fourteen derivative traits was 0.90, with a range of 0.78 to 0.96 (Table 1). These high heritabilities suggest that variation for these metabolite grain traits are influenced mainly by genetic rather than environmental effects.

**Table 1:** Means and ranges (in μg·g^−1^) seed for untransformed BLUPs of 20 tocochromanol grain traits evaluated on a maize inbred association panel and estimated heritability on a line-mean basis in two summer environments, in West Lafayette, Indiana, across 2 years

Trait	No. Lines	BLUPs			Heritabilities	
		Mean	SD	Range	Estimate	SE
γT	251	30.18	15.00	5.04–85.94	0.88	0.02
γT3	252	12.08	8.73	1.46–55.25	0.89	0.01
αT	252	8.19	5.50	0.70–31.35	0.91	0.01
αT3	250	7.52	3.01	2.86–22.38	0.87	0.02
δT	251	1.11	0.63	0.22–3.32	0.78	0.03
δT3	250	0.59	0.68	0.09–6.06	0.91	0.01
Total T3	252	20.34	10.66	3.77–74.59	0.90	0.01
Total T	252	39.95	15.05	13.16–95.07	0.85	0.02
Total T/Total T3	247	2.44	1.70	0.37–9.34	0.92	0.01
Total T3 + T	252	60.54	18.10	25.70–125.14	0.83	0.02
δT/(γT + αT)	251	0.03	0.02	0.0027–0.08	0.89	0.01
δT/γT	251	0.04	0.02	0.0012–0.18	0.91	0.01
δT/αT	248	0.28	0.35	0.013–2.04	0.94	0.01
γT/(γT + αT)	251	0.75	0.17	0.18–0.97	0.95	0.01
δT3/(γT3 + αT3)	251	0.03	0.02	0.0039–0.22	0.94	0.01
δT3/γT3	251	0.05	0.04	0.0046–0.25	0.89	0.01
δT3/αT3	250	0.09	0.11	0.0080–0.80	0.93	0.01
γT3/(γT3 + αT3)	251	0.55	0.18	0.096–0.92	0.95	0.01
αT/γT	250	0.40	0.42	0.018–2.21	0.88	0.01
αT3/γT3	251	1.24	1.43	0.18–11.75	0.96	0.01

BLUPs, best linear unbiased predictors; SD, standard deviation of the BLUPs; SE, standard error of the heritabilities; γT, γ-tocopherol; γT3, γ-tocotrienol; αT, α-tocopherol; αT3, α-tocotrienol; δT, δ-tocopherol; δT3, δ-tocotrienol; total T3, total tocotrienols; total T, total tocopherols; total T3 + T, total tocochromanols.

### Genome-wide analysis

**Figure 2:**
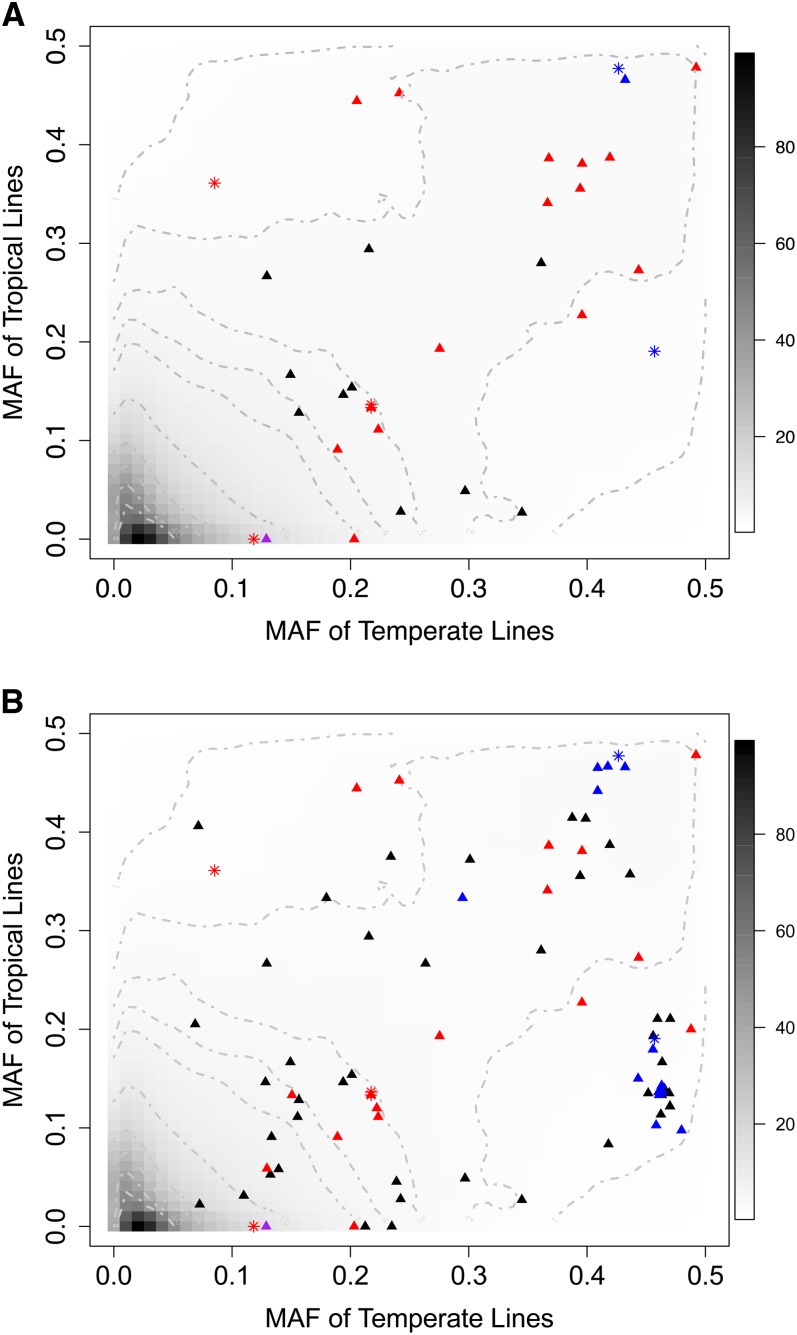
Comparison of MAFs for SNPs between temperate and tropical lines in the maize association panel. (A) Contour plot of MAFs for 591,822 SNPs between temperate (*n* = 207) and tropical (*n* = 45) maize lines. For each SNP, the minor allele across all lines was identified, followed by calculation of the frequency of this allele for temperate and tropical lines. The grayscale indicates the percentage of SNPs with each set of MAFs. Colored symbols (triangles and asterisks) indicate MAFs of SNPs that are statistically significant for at least one of the 20 tocochromanol traits at a genome-wide FDR of 5%. Significant SNPs ± 250 kb of *ZmVTE4*, *ZmVTE1*, and *ZmHGGT1* are colored red, blue, and purple, respectively. All other statistically significant SNPs are colored black. The SNPs that were included in the optimal models of the MLMM analysis are indicated with asterisks. (B) Contour plot of MAFs for 591,822 SNPs between temperate and tropical lines with additional SNPs significant at a genome-wide FDR of 10%, as in (A).

We explored the genetic basis of natural variation for tocochromanol levels in maize grain by using an association panel of diverse inbred lines genotyped with 591,822 SNP markers. Removal of SNPs with a MAF <5% resulted in a data set of 294,092 SNPs for use in a GWAS of 20 tocochromanol traits with a unified mixed linear model that controls for population structure and familial relatedness. A total of 34 SNPs were significant for at least one trait at a genome-wide FDR of 5% (Figure S1 and Table S4). Given that the sample size of the association panel (*n* = 252) only has adequate statistical power to repeatedly detect large effect QTL (Long and Langley 1999), we searched for more modest effect QTL at a genome-wide FDR of 10%. With this less conservative FDR level, we identified 52 additional SNPs significantly associated with at least one trait (Table S4). In general, this less conservative FDR level increased the density of SNPs at strong association signals that were initially detected at a genome-wide FDR of 5%. Although all of the 34 SNPs with significant associations at 5% FDR had MAFs ≥0.09 for temperate lines (*n* = 207) of the association panel, 23.5% of these SNPs had MAFs ≤0.05 for tropical lines (*n* = 45, Figure 2A). Similar results were observed for the 52 additional SNPs significant at 10% FDR (Figure 2B). These results indicate that the association panel does not provide sufficient statistical power to detect rare causative alleles from tropical germplasm.

**Figure 3:**
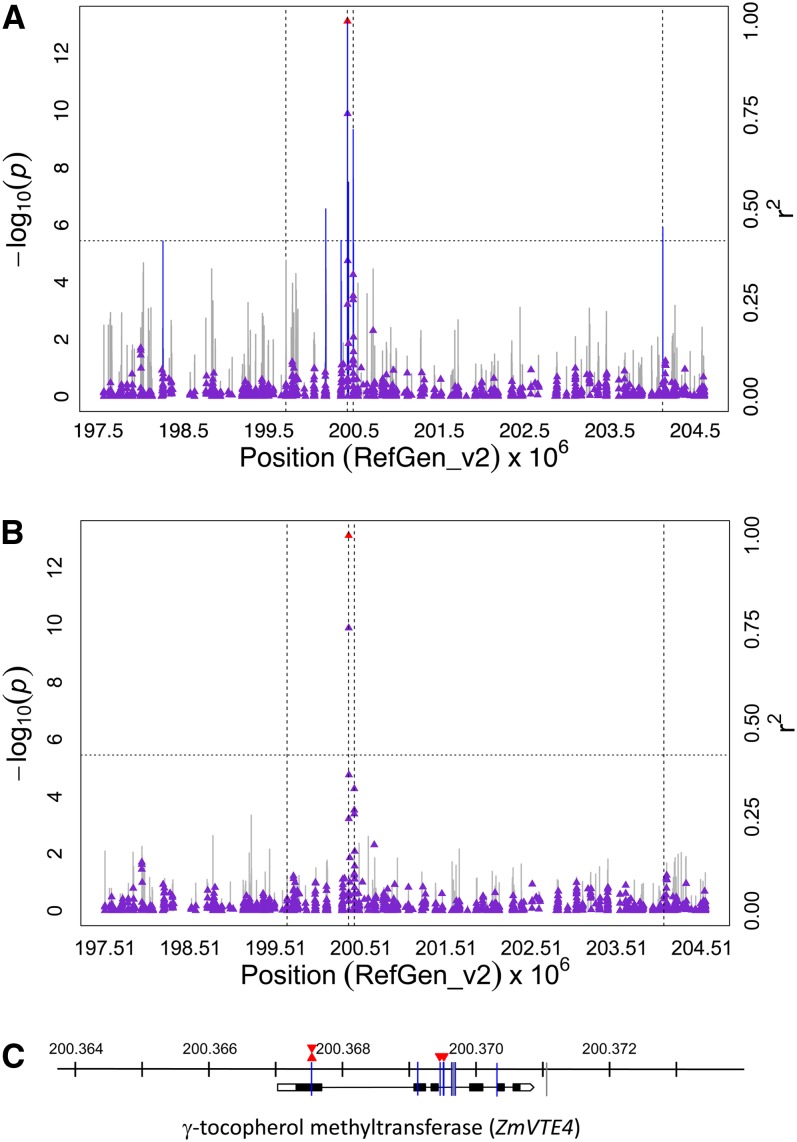
GWAS for α-tocopherol (αT) content in maize grain. (A) Scatter plot of association results from a unified mixed model analysis of αT and LD estimates (*r^2^*) across the *ZmVTE4* chromosome region. Negative log_10_-transformed *P*-values (left, y-axis) from a GWAS for αT and *r^2^* values (right, y-axis) are plotted against physical position (B73 RefGen_v2) for a 7-Mb region on chromosome 5 that encompasses *ZmVTE4*. The blue vertical lines are –log_10_ *P*-values for SNPs that are statistically significant for αT at 5% FDR, whereas the gray vertical lines are –log_10_ *P*-values for SNPs that are non-significant at 5% FDR. Triangles are the *r^2^* values of each SNP relative to the peak SNP (indicated in red) at 200,367,532 bp. The black horizontal dashed line indicates the –log_10_ *P*-value of the least statistically significant SNP at 5% FDR. The black vertical dashed lines indicate the positions of four genes (from left to right): a WYRKY transcription factor (GRMZM5G823157), *ZmVTE4* (GRMZM2G035213), a pentatricopeptide repeat-containing protein (GRMZM2G325019), and an amino acid permease (GRMZM2G161641). (B) Scatter plot of association results from a conditional unified mixed model analysis of αT and LD estimates (*r^2^*) across the *ZmVTE4* chromosome region, as in (A). The three SNPs (ss196416269, S5_200369534, and S5_200369481) from the optimal MLMM model were included as covariates in the unified mixed model to control for the *ZmVTE4* effect. (C) Gene model diagram for *ZmVTE4* with αT associated SNPs. Blue vertical lines indicate the physical position (RefGen_v2) of SNPs within ±3 kb of the open reading frame start or stop position for *ZmVTE4* that are significantly associated with αT at 5% FDR. Significant SNPs at 10% FDR are shown as gray vertical lines. The peak SNP is indicated by a red triangle, whereas the three SNPs included in the optimal MLMM model are indicated by inverted red triangles.

**Figure 4:**
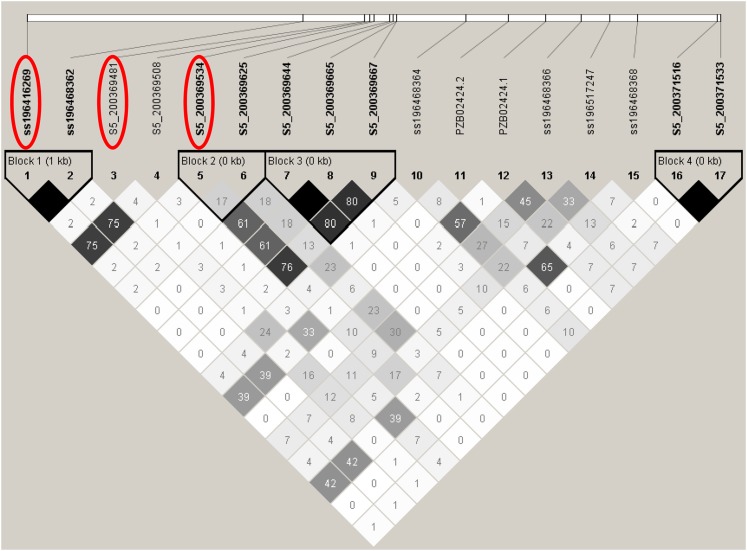
Summary of local LD and haplotype blocks for a 3.9-kb genomic region that surrounds *ZmVTE4* (Chr 5: 200,367,029–200,370,851 bp). LD plot, generated in Haploview (Barrett *et al.* 2005), indicates *r^2^* values between pairs of SNPs multiplied by 100; white, *r*^2^ = 0; shades of gray, 0 < *r*^2^ < 1; black *r^2^* = 1. Haplotype blocks (blocks 1−4) in the *ZmVTE4* genomic region were defined with the confidence interval method (Gabriel *et al.* 2002). The three SNPs included in the optimal MLMMs for α-tocopherol and its three derived trait ratios are indicated with red circles.

The strongest association signal was detected for αT on chromosome 5, with the peak SNP locus (PZB02283.1/ss196416269; 200,367,532 bp; *P*-value 7.36 × 10^−14^) located within *ZmVTE4* (GRMZM2G035213)—the gene that encodes γ-tocopherol methyltransferase (γTMT) in maize (Figure 3A). Indicative of the rapid decay of linkage disequilibrium (LD) detected at *ZmVTE4* (Figure 4), LD estimates (*r^2^*) for marker pairs with the peak SNP locus were ≤0.36 over distances of > 3.5 kb. This SNP locus was also strongly associated (*P*-values 1.88 × 10^−11^ to 9.21 × 10^−13^) with three αT-related trait ratios: αT/γT, γT/(γT + αT), and δT/αT (Figure S2, Figure S3, Figure S4). An additional 24 of the 34 SNPs significant at 5% FDR were associated with at least one of these four αT-related traits and present on chromosome 5. However, only seven of these 24 SNPs were located within 3 kb of the *ZmVTE4* start and stop codons (a conservative definition of the *ZmVTE4* gene). Of the 17 remaining significant SNPs, 15 were within 3.7 Mb of *ZmVTE4* but also within 3 kb of genes that encode other proteins such as a WYRKY transcription factor (GRMZM5G823157), a pentatricopeptide repeat-containing (PPR) protein (GRMZM2G325019), and an amino acid permease (GRMZM2G161641; see Table S4 for the full list of these genes).

Given that nearly all of the statistically significant SNPs at 5% FDR were located within a 5.9 Mb segment on chromosome 5, the multilocus mixed model (MLMM) (Segura *et al.* 2012) was used on a chromosome-wide scale to better resolve the complex association signals for αT and its three derived trait ratios. The optimal models obtained with the forward-backward stepwise approach of MLMM contained the same three SNPs (ss196416269, S5_200369481, and S5_200369534) within *ZmVTE4* that together explained 33–45% of the total phenotypic variation for the four traits (Table S5). The three MLMM-identified SNPs were essentially in linkage equilibrium (*r^2^* ≤ 0.023) with each other and tagged two of the four short *ZmVTE4* haplotype blocks detected by Haploview (Figure 4). When we reconducted GWAS using the unified mixed model with these three SNP loci as covariates, all of the SNPs on chromosome 5 previously significant at 5% FDR, as well as the two SNPs on chromosomes 6 and 9, were no longer statistically significant at an FDR of 5% or 10% for any of the four αT-related traits (Figure 3B). These findings from the conditional analysis suggest that the 19 non-*ZmVTE4* SNPs (*i.e.*, not within 3 kb of *ZmVTE4*) on chromosomes 5, 6, and 9 were likely spurious associations.

We used a haplotype approach to assess the joint effect of the three MLMM-identified *ZmVTE4* SNPs on levels of αT in maize grain. Of the five observed haplotypes in the maize association panel, αT was highest when the favorable alleles of all three MLMM-identified SNPs were combined (haplotype G, G, G), shown in Table 2. In addition, this combination of the three SNPs accounted for 48.4% of the phenotypic variation for αT. Similar results were observed for the three derivative trait ratios: αT/γT, γT/(γT+αT), and δT/αT (Table S6). The most favorable haplotype (G, G, G) had an average αT concentration of 15.95 ± 3.90 μg·g^−1^(*n* = 28), whereas the other four haplotypes (*n* = 224) averaged 7.15 ± 4.63 μg·g^−1^. When contrasting the most and least favorable haplotype classes, we observed a 5.76-fold change in average αT values. Although the most favorable haplotype occurred at a low frequency (11%) in the maize association panel, it was the only haplotype that was essentially equally distributed between temperate (*n* = 15) and tropical (*n* =13) germplasm.

**Table 2:** Haplotype effects of three *ZmVTE4* SNPs identified with an optimal MLMM for αT levels

Haplotype*^a^*	*ZmVTE4* SNPs			Haplotype Frequency			Haplotype Mean	
	ss196416269	S5_200369481	S5_200369534	Overall	Temperate	Tropical	αT, μg·g^−1^	SD
A,C,G	A	C	G	50	44	6	3.02	2.48
G,C,G	G	C	G	151	125	26	9.18	4.01
A,G,G	A	G	G	1	1	−	3.51	−
G,G,G	G	G	G	28	15	13	15.95	3.90
G,C,T	G	C	T	22	22	−	2.77	2.83
								
						R^2^_LR_ (%)*^b^*	60.2	
						Partial R^2^_LR_ (%)*^c^*	48.4	
						*P*-value*^d^*	2.2 × 10^−38^	
						Fold change*^e^*	5.76	

MLMM, multilocus mixed model; αT, alpha tocopherol; SNP, single-nucleotide polymorphism; SD, standard deviation of the untransformed BLUPs; BLUPs, best linear unbiased predictors;

a The most favorable allele for each of the *ZmVTE4* SNPs is underlined.

b R^2^_LR,_ likelihood-ratio based R^2^ statistic, percentage of total phenotypic variation explained by the unified mixed model.

c Partial R^2^_LR_, likelihood-ratio based partial R^2^ statistic, percentage of total phenotypic variation explained by the haplotypes.

d The *P*-value was from a unified mixed linear model that tested for an association between haplotypes and αT levels.

e Fold change was calculated as the ratio between the most favorable (G, G, G) and least favorable (G, C, T) haplotypes for αT levels.

**Figure 5:**
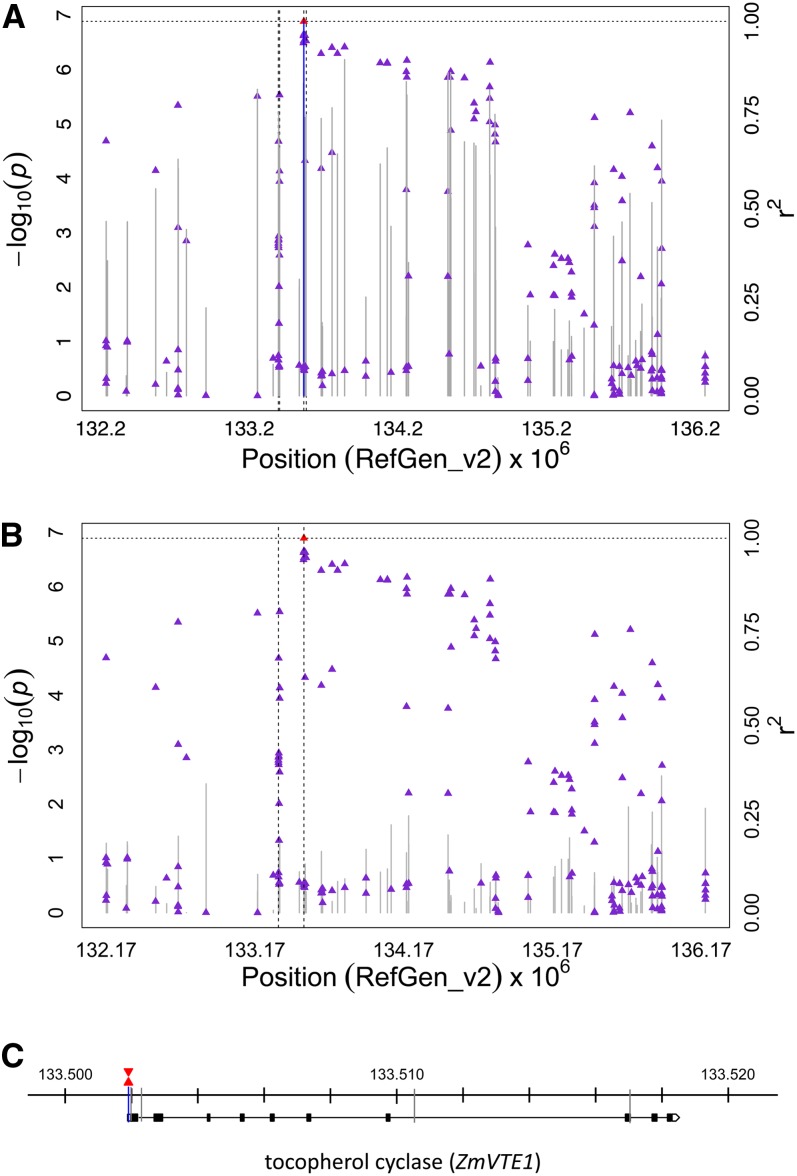
GWAS for the ratio of δT3 to the sum of γT3 and αT3 [δT3/(γT3 + αT3)] in maize grain. (A) Scatter plot of association results from a unified mixed model analysis of δT3/(γT3 + αT3) and LD estimates (*r^2^*) across the *ZmVTE1* chromosome region. Negative log_10_-transformed *P*-values (left, y-axis) from a GWAS for δT3/(γT3 + αT3) and *r^2^* values (right, y-axis) are plotted against physical position (B73 RefGen_v2) for a 4-Mb region on chromosome 5 that encompasses *ZmVTE1*. The blue vertical lines are –log_10_ *P*-values for SNPs that are statistically significant for δT3/(γT3+αT3) at 5% FDR, whereas the gray vertical lines are –log_10_ *P*-values for SNPs that are nonsignificant at 5% FDR. Triangles are the *r^2^* values of each SNP relative to the peak SNP (indicated in red) at 133,501,858 bp. The black horizontal dashed line indicates the –log_10_ *P*-value of the least statistically significant SNP at 5% FDR. The black vertical dashed lines indicate the positions of two genes (from left to right): a transcription factor (GRMZM2G105494) and *ZmVTE1* (GRMZM2G009785). (B) Scatter plot of association results from a conditional unified mixed model analysis of δT3/(γT3+αT3) and LD estimates (*r^2^*) across the *ZmVTE1* chromosome region, as in (A). The SNP (S5_133501858) from the optimal multi-locus mixed model (MLMM) model was included as a covariate in the unified mixed model to control for the *ZmVTE1* effect. (C) Gene model diagram for *ZmVTE1* with δT3/(γT3 + αT3) associated SNPs. Blue vertical lines indicate the physical position (RefGen_v2) of SNPs within +/− 3 kb of the open reading frame start or stop position for *ZmVTE1* that are significantly associated with δT3/(γT3 + αT3) at 5% FDR. Significant SNPs at 10% FDR are shown as gray vertical lines. The peak SNP is indicated by a red triangle, while the SNP included in the optimal MLMM model is indicated by an inverted red triangle.

A moderately strong association signal relative to *ZmVTE4* was identified for the ratio of δT3 to the sum of γT3 and αT3 [δT3/(γT3 + αT3)] on chromosome 5, with the peak SNP locus (S5_133501858; 133,501,858 bp; *P*-value 1.29 × 10^−7^) located 70 bp from the transcriptional start site of the gene that encodes tocopherol cyclase, *ZmVTE1* (GRMZM2G009785; Figure 5A). As expected for this recombinationally suppressed pericentromeric region of chromosome 5 (Gore *et al.* 2009), LD estimates (*r^2^*) for marker pairs with the peak SNP locus revealed long-range patterns of LD (*i.e.*, > 50 kb) limiting mapping resolution, especially downstream of *ZmVTE1*. We also identified two SNPs (S5_133333397; *P*-value 3.06 × 10^−7^ and S5_133333561; *P*-value 3.12 × 10^−7^) within a putative transcription factor (GRMZM2G105494; Figure S5A) and ~168 kb away from *ZmVTE1* that were significantly associated with δT3 and δT3/(γT3 + αT3) at the 5% and 10% FDR levels, respectively. When the FDR was relaxed to 10%, an additional 27 SNPs spanning a 3.3-Mb interval that included *ZmVTE1* were associated with at least one of these two tocotrienol-related traits (Table S4).

As was conducted for the four αT-related traits, MLMM was used on a chromosome-wide level to better clarify association signals for the two highly correlated tocotrienol-related traits (*r* = 0.81). The optimal model obtained for δT3 and δT3/(γT3 + αT3) contained only a single SNP (S5_133333561 and S5_133501858, respectively) and explained 9–10% of the total phenotypic variation (Table S5). Both of these SNPs were significant for their respective traits at 5% FDR in the initial GWAS with the unified mixed model (Table S4). Although separated by a distance of 168.3 kb, these two SNPs were in moderate linkage disequilibrium (*r^2^* = 0.42). However, only S5_133333561 was previously significantly associated with both tocotrienol-related traits at 10% FDR. When we reconducted GWAS using the unified mixed model with one of these two SNPs as covariates for their respective traits, all of the SNPs previously significant for these two traits at 5% or 10% FDR were no longer significant at either FDR level (Figure 5B; Figure S5B).

### Pathway-level analysis

In a large-scale GWAS, it is challenging to identify variants with moderate to weak effect sizes because of the need to control for the problem of simultaneously testing hundreds of thousands of SNP markers (*i.e.*, multiplicity). Given this limitation and the potential that additional *a priori* candidate genes from well-characterized biochemical pathways may contribute to variability of tocochromanol levels in maize grain (Chander *et al.* 2008; Shutu *et al.* 2012; Wong *et al.* 2003), we retested for an association between the 20 tocochromanol traits and 6,122 SNPs within ± 250 kb of the *a priori* candidate gene set of 60 loci. The corresponding FDR adjustment only considered this prioritized subset of 6122 SNPs from the initial GWAS results (see Materials and Methods and Table S2). Of the retested 6122 SNPs, 91 were statistically significant at 5% FDR for at least one of 15 tocochromanol traits. Not surprisingly, 64 of these significant SNPs were within ± 250 kb of *ZmVTE4* and *ZmVTE1*, whereas new findings consisted of 27 significant SNPs within ± 250 kb of an additional 11 candidate genes. Of these 11 candidates, one was from the tocochromanol biosynthetic pathway, six were from the prenyl group synthesis pathway, and four were from the aromatic head group pathway (Table S7).

Because of the moderate-to-strong association signals at *ZmVTE1* and *ZmVTE4*, we also conducted three additional pathway-level analyses that accounted separately or jointly for the potential confounding effects of these two loci (Platt *et al.* 2010). With the three *ZmVTE4* SNPs identified by MLMM included as covariates, 39 significant SNPs at 5% FDR were identified within ±250 kb of six genes that were members of the tocochromanol biosynthetic (2), prenyl group synthesis (2), and aromatic head group (2) pathways (Table 3; Table S7). All but one of these 39 SNPs were a subset of the 91 significant SNPs detected in the pathway-level analysis without the inclusion of *ZmVTE4* or *ZmVTE1* SNPs as covariates. When the two MLMM identified *ZmVTE1* SNPs were similarly included as covariates, we identified 57 significant SNPs at 5% FDR that were within ±250 kb of 10 genes involved in the tocochromanol biosynthetic (2), prenyl group synthesis (4), and aromatic head group (4) pathways. With the exception of a SNP on chromosome 9, these SNPs were also previously detected in the pathway-level analysis that did not control for *ZmVTE4* or *ZmVTE1* effects. Taken together, these two conditional analyses detected 12 of the 13 genes previously detected in the pathway-level analysis without the inclusion of MLMM identified *ZmVTE4* or *ZmVTE1* SNPs as covariates. Although the gene encoding a hydroxymethylbutenyl 4-diphosphate synthase (GRMZM2G137409) was not reidentified in either conditional analysis, a gene that encodes for a shikimate kinase (GRMZM2G070218) was newly detected when the three MLMM identified *ZmVTE4* SNPs were included as covariates.

**Table 3:** Statistically significant results from the pathway-level analysis of 20 tocochromanol grain traits when SNPs identified from the MLMM analysis are excluded or included as covariates in the unified mixed model

			Significant Associations			
Category	Candidate Gene	Function	No Covariates*^a^*	Three *ZmVTE4* SNPs as Covariates*^b^*	Two *ZmVTE1* SNPs as Covariates*^c^*	Three *ZmVTE4* SNPs and two *ZmVTE1* SNPs as Covariates*^d^*
AHG	GRMZM2G573867	3-dehydroquinate synthase	αT, δT/αT, αT/γT, γT/(γT + αT)		αT, δT/αT	
AHG	GRMZM2G124365	chorismate mutase	αT3, Total T3, αT, δT/αT		αT3, Total T3, αT, δT/αT	
AHG	GRMZM2G138624	isochorismatase hydrolase	αT3		αT3	
AHG	GRMZM2G437912	prephenate dehydratase	Total T3	Total T3	Total T3, γT3	Total t3, γT3
AHG	GRMZM2G070218	shikimate kinase		δT/αT		
PG	GRMZM2G493395	1-deoxy-D-xylulose 5-phosphate synthase	αT/γT, γT/(γT + αT)		αT/γT, γT/(γT + αT)	
PG	AC209374.4_FG002	2-C-methyl-D-erythritol 2,4-cyclodiphosphate synthase	αT, αT/γT, γT/(γT + αT), δT/αT		αT, αT/γT, γT/(γT + αT), δT/αT	
PG	GRMZM2G172032	2-C-methyl-D-erythritol 4-phosphate cytidyltransferase	δT3/(γT3 +αT3), δT3	δT3/(γT3 + αT3)		
PG	GRMZM2G027059	4-hydroxy-3-methylbut-2-enyldiphosphate reductase	δT3, αT	δT3	αT	
PG	GRMZM2G137409	hydroxymethylbutenyl 4-diphosphate synthase	δT3/γT3			
PG	GRMZM2G133082	isopentenyl pyrophosphate isomerase	γT/(γT + αT)		γT/(γT+αT)	
TP	GRMZM2G009785	tocopherol cyclase (*ZmVTE1*)	δT/γT, δT3, δT3/(γT3 + αT3), δT3/αT3, δT3/γT3	δT/γT, δT3, δT3/(γT3 + αT3), δT3/αT3, δT3/γT3		
TP	GRMZM2G035213	γ-tocopherol methyltransferase (*ZmVTE4*)	αT3, αT3/γT3, γT3/(γT3 + αT3), αT, αT/γT, δT/αT, γT/(γT + αT)		αT3, αT3/γT3, γT3/(γT3 + αT3), αT, αT/γT, δT/αT, γT/(γT + αT), δT/(γT + αT)	
TP	GRMZM2G173358	homogentisic acid geranylgeranyl transferase *ZmHGGT1*)	Total T3, Total T/Total T3, γT3, γT3/(γT3 + αT3), αT3	Total T3, Total T/Total T3, γT3, γT3/(γT3 + αT3)	Total T3, Total T/Total T3, γT3, γT3/(γT3 + αT3), αT3	Total T3, total T/total T3, γT3, γT3/(γT3 + αT3)

SNP, single-nucleotide polymorphism; MLMM, multilocus mixed model; AHG, aromatic head group; PG, prenyl group synthesis; TP, tocochromanol pathway.

a At least one SNP within ±250 kb of the gene open reading frame (ORF) start or stop position is associated with at least one of the indicated traits at a candidate gene-wide 5% false-discovery rate (FDR) using the unified mixed model without covariates.

b At least one SNP within ±250 kb of the gene ORF start or stop position is associated with at least one of the indicated traits at a pathway-wide 5% FDR using the unified mixed model with the three *ZmVTE4* SNPs identified in the MLMM analysis included as covariates.

c At least one SNP within ±250 kb of the gene ORF start or stop position is associated with at least one of the indicated traits at a pathway-wide 5% FDR using the unified mixed model with the two *ZmVTE1* SNPs identified in the MLMM analysis included as covariates.

d At least one SNP within ±250 kb of the gene ORF start or stop position is associated with at least one of the indicated traits at a pathway-wide 5% FDR using the unified mixed model with the three *ZmVTE4* SNPs and two *ZmVTE1* SNPs identified in the MLMM analysis included as covariates.

Controlling for the effect of both QTL with the three *ZmVTE4* and two *ZmVTE1* SNPs as covariates resulted in a more stringent conditional analysis with only 10 SNPs, all for tocotrienol-related traits, being significant at 5% FDR within ±250 kb of two candidate genes (Table 3; Table S7). The start codon for a gene encoding a prephenate dehydratase (GRMZM2G437912) was 23.4 kb away from two SNPs (S2_59013838 and S2_59013840) that were significantly associated with both γT3 and total tocotrienols. We also identified three SNPs (S9_92313446, ss196492047, and S9_92346116) significantly associated with total tocotrienols and/or γT3 that were located 137.4 to 170.1 kb upstream of the start codon for a gene that encodes a homogentisate geranylgeranyl transferase (*ZmHGGT1*, GRMZM2G173358). In addition, two SNPs (S9_92548696 and S9_92554465) that were located 61.4 to 67.2 kb downstream of the *ZmHGGT1* stop codon were associated with γT3 and/or the ratio of total tocopherols to total tocotrienols (total T/total T3), whereas three SNPs (S9_92718671, S9_92718674, S9_92718709) located 231,403 to 231,441 bp downstream of the stop codon were significantly associated with both γT3 and the ratio of γT3 to the sum of γT3 and αT3 [γT3/(γT3+αT3)]. Although there were five additional SNPs within ±3 kb of the start and stop codons for both the prephenate dehydratase and *ZmHGGT1* genes, none were significantly associated at 5% FDR with any of the tocochromanol-related traits in the pathway-level analysis.

## Discussion

Vitamin E was one of the first vitamin biosynthetic pathways to be fully elucidated in plants, primarily from studies in the model photosynthetic organisms *A. thaliana* and *Synechocystis* sp. PCC6803 (reviewed in DellaPenna and Mène-Saffrané 2011). In concordance with transgenic and mutagenesis studies of the core tocochromanol pathway genes (*HPPD*, *VTE1* through *5* and *HGGT*; reviewed in DellaPenna and Mène-Saffrané 2011), QTL data from several species show intervals containing these genes are important for determining seed tocopherol composition and content, but also that numerous other unknown loci are involved, often with contributions similar to that of presumed core pathway loci (Chander *et al.* 2008; Dwiyanti *et al.* 2011; Fritsche *et al.* 2012; Gilliland *et al.* 2006; Haddadi *et al.* 2012; Hass *et al.* 2006; Jackson *et al.* 2008; Li *et al.* 2012; Shutu *et al.* 2012; Sookwong *et al.* 2009; Tang *et al.* 2006; Wang *et al.* 2012; Wong *et al.* 2003). Aside from *ZmVTE4* (Li *et al.* 2012), the degree to which additional pathway genes and other loci contribute to natural variation for tocochromanols in maize grain is still unclear. To address this issue, we conducted two complementary studies for tocopherol and tocotrienol traits in maize grain: a GWAS and a parallel study of 60 *a priori* candidate genes for the same traits. This provides the most thorough forward genetic analysis of natural variation for tocopherols and tocotrienols in any system.

We first assessed the near complete chemical diversity of tocochromanols (α-, δ-, and γ- tocopherols and tocotrienols) in grain from a maize association panel that was assembled to represent the genetic diversity found in public sector maize breeding programs worldwide (Flint-Garcia *et al.* 2005). With a 7.8- to 67.3-fold range in phenotypic variation for the six tocochromanol compounds, the worldwide maize germplasm pool appears to possess diversity for this pathway at a level needed to markedly increase vitamin E and antioxidant levels in grain. The high estimates of line-mean basis heritability for the six compounds and their thirteen derivative traits imply that QTL are predominantly responsible for this tremendous range of phenotypic variation. Furthermore, these estimates of heritability suggest that the tocochromanol traits should respond well to artificial selection in a maize biofortification breeding program. The efficiency of artificial selection on this tremendous phenotypic variation could be enhanced substantially through connecting phenotypic information to molecular markers associated with QTL alleles that enhance the contents of vitamin E and antioxidant in grain.

The maize association panel was scored for 536,988 SNPs with a GBS protocol (Elshire *et al.* 2011)—a nearly 10-fold addition to the SNPs that were previously scored on the panel with the MaizeSNP50 BeadChip (Cook *et al.* 2012) and several other types of genotyping assays (McMullen *et al.* 2009; Yu *et al.* 2006). This greater SNP marker density enhanced a GWAS of tocochromanol traits in maize grain that resulted in the identification of significant associations at a genome-wide level for two core tocochromanol pathway genes, *ZmVTE4* (γTMT) and *ZmVTE1* (tocopherol cyclase). The strong associations of *ZmVTE4* SNPs with αT (the product of γTMT) are in agreement with results from a GWAS conducted by Li *et al.* (2012) to identify genetic variants that control tocopherol content in maize grain. With a panel of 478 inbred lines also scored with the MaizeSNP50 BeadChip, Li *et al.* (2012) identified three SNPs within *ZmVTE4*, as well as two separately scored indels within the *ZmVTE4* 5′ UTR and promoter regions that were significantly associated with αT at a Bonferroni-corrected threshold of 5%. Similar to Li *et al.* (2012), we did not identify significant genome-wide associations at 5% FDR for additional core pathway genes when only SNPs from the MaizeSNP50 BeadChip were considered. It was only with the addition of the GBS SNPs that a statistically significant association of *ZmVTE1* SNPs with δT3/(γT3 + αT3) was observed.

Our strongest detected associations were between *ZmVTE4* SNPs and four αT-related traits, but additional highly significant associations were dispersed across multiple LD blocks of a 13.3-Mb genomic interval that surrounds *ZmVTE4*. The MLMM method consistently resolved this complex region into three independent association signals represented by one MaizeSNP50 BeadChip SNP and two GBS SNPs, all located within *ZmVTE4*. Although not as extensive, Li *et al.* (2012) identified six additional SNPs within a 2.4-Mb region surrounding *ZmVTE4* that were also significantly associated with αT content. They showed that only one of these six SNPs, located within an intron of a PPR protein ~85 kb upstream of *ZmVTE4*, influenced αT content independently of the SNPs and indels within *ZmVTE4* and suggested it marked a *ZmVTE4* enhancer element. In our study of a different maize association panel scored for a ~10-fold greater number of SNPs, we also identified significant associations with PPR protein SNPs (including the same intronic SNP), but the optimal models obtained with MLMM always only included three *ZmVTE4* SNPs. Thus, our findings do not support the presence of an enhancer element ~85 kb upstream of *ZmVTE4*. However, our results do provide a second line of evidence for multiple *ZmVTE4* alleles (*i.e.*, allelic heterogeneity), but with the caveat that multiple rare variants could also have created highly significant independent signals of synthetic association over an extensive genomic region (Dickson *et al.* 2010).

Although association signals were dramatically less dispersed for two tocotrienol-related traits relative to the four αT-related traits, the MLMM method was still needed to clarify associations within a 3.3-Mb interval that surrounds the *ZmVTE1* locus. Interestingly, a SNP located 70 bp from the transcriptional start site of *ZmVTE1* was identified by MLMM for δT3/(γT3 + αT3), whereas a SNP within a putative transcription factor (GRMZM2G105494), ~168 kb away from *ZmVTE1*, was identified by MLMM for δT3. This was an unexpected result given that these two tocotrienol traits are strongly correlated (*r* = 0.81), thus they are more likely to be under highly similar genetic control. From a biosynthetic perspective, it is straightforward to rationalize *ZmVTE1* as a causative gene affecting tocotrienol levels as tocopherol cyclase from a variety of organisms have been shown to utilize both tocopherol and tocotrienol substrates (phytylated and geranylgeranylated compounds, respectively) *in vitro* and *in vivo* (Kumar *et al.* 2005; Porfirova *et al.* 2002; Sattler *et al.* 2003; Stocker *et al.* 1996). In contrast, the putative transcription factor, which contains a predicted basic helix−loop−helix DNA-binding domain, has not previously been implicated in the regulatory control of tocochromanol biosynthesis, though this is possible as it is expressed at low levels in developing embryos (Sekhon *et al.* 2011). With the available data it is impossible to differentiate whether *ZmVTE1* is affected by a distant regulatory element marked by the SNP within the putative basic helix-loop-helix transcription factor, whether the transcription factor directly impacts δT3 levels or whether a combination of complex LD patterns and allelic heterogeneity (Atwell *et al.* 2010) contributed to a stronger association of δT3 levels with the transcription factor than causative variants at *ZmVTE1*. Regardless, long-range LD throughout the *ZmVTE1* genomic interval severely restricts our ability to identify the causal gene or variants with the MLMM method, thus large biparental populations are needed to more finely resolve causative variants at the *ZmVTE1* locus.

We showed that tocochromanol traits in maize grain are highly heritable, but *ZmVTE4* (33–45%) and *ZmVTE1* (9–10%) accounted for only a portion of the estimated heritability for their associated traits. Why is the heritability still unexplained or “missing” for these traits? Even though GBS increased the number of SNPs scored in the panel by ~10-fold and enabled a GWAS of the Flint-Garcia *et al.* (2005) maize association panel with unprecedented marker coverage, complete genome coverage (*r^2^* >0.8) for a GWAS in diverse maize with such a rapid breakdown of LD (Remington *et al.* 2001) may require an additional order of magnitude of polymorphisms to be scored (Gore *et al.* 2009). This is especially important if the missing heritability of tocochromanol traits in the association panel is predominantly explained by numerous small effect QTL (Buckler *et al.* 2009; Yang *et al.* 2010). In addition, a sample size of only 252 lines does not provide adequate statistical power to identify QTL with small effects (Long and Langley 1999). Importantly, the weakening of statistical power as a result of incomplete genome-wide marker coverage and small sample size will be intensified if causal variants have lower MAFs (*i.e.*, rare causative alleles) than the genotyped SNPs used for the GWAS (Yang *et al.* 2010)—an unfavorable situation that likely occurred in our GWAS of tocochromanol grain traits (Figure 2, A and B). We intend to address the “missing heritability” problem by determining the genetic basis of these same tocochromanol traits in the maize nested association mapping panel (McMullen *et al.* 2009).

We conducted a pathway-level analysis to reduce the magnitude of the multiple-testing problem relative to a genome-wide analysis and provide an opportunity to detect variants with relatively weaker effect sizes that may not necessarily be significant at the genome-wide level. Association signals were considered for SNPs within ±250 kb of the 60 candidate genes to identify any distant regulatory elements, as had been previously reported from QTL fine mapping studies of the *Vgt1* and *tb1* genes (Clark *et al.* 2006; Salvi *et al.* 2007). As expected the two genes with significant genome-wide associations, *ZmVTE4* and *ZmVTE1*, accounted for 70% of the significantly associated SNPs in this pathway-level analysis, with pathway-level association occurring for a larger number of traits (Table S7). In addition to the strong genome-wide association with δT3/(γT3 + αT3), *ZmVTE1* showed more modest pathway associations with δT3, δT3/αT3, δT3/γT3, and one tocopherol trait, δT/γT. Similarly, in addition to the four strongly associated, genome-wide αT-related traits, *ZmVTE4* also had pathway-level associations with three tocotrienol traits: αT3, αT3/γT3, and γT3/(γT3 + αT3). Thus, genome-wide associations suggested *ZmVTE4* and *ZmVTE1* only impacted specific tocopherols and tocotrienols, respectively, a finding at odds with the known biosynthetic activities of the encoded enzymes toward both tocopherols and tocotrienols (Cahoon *et al.* 2003; Porfirova *et al.* 2002; Soll *et al.* 1980; Soll and Schultz 1979; Stocker *et al.* 1996). Pathway-level association analysis resolves this apparent inconsistency by demonstrating that that *ZmVTE4* and *ZmVTE1* both impact tocopherols and tocotrienols, albeit with different levels of contributions, a finding consistent with their known *in vitro* and *in vivo* activities toward both classes of tocochromanols (Cahoon *et al.* 2003; Porfirova *et al.* 2002; Soll *et al.* 1980; Soll and Schultz 1979; Stocker *et al.* 1996).

The remaining 30% of the significant SNPs in the pathway-level analysis showed modest associations to 11 of the remaining 58 candidate genes. Because of the large impacts of *ZmVTE4* and *ZmVTE1*, these 11 candidate genes were reassessed in a model containing the *ZmVTE4* and *ZmVTE1* SNPs identified with MLMM as covariates, which reduced the number of candidate genes with significant associations from 11 to 2: *ZmHGGT1* (encoding homogentisate geranylgeranyl transferase) and GRMZM2G437912, which encodes one of at least four prephenate dehydratase paralogs in the maize genome. Both loci were associated exclusively with tocotrienol traits. Relative to homogentisate phytyltransferase−type enzymes (*e.g.*, *ZmVTE2*), HGGTs show a marked preference for GGDP over phytol-PP (Zhang *et al.* 2013). When expressed in plants that lack tocotrienols monocot HGGT was shown to be sufficient to confer tocotrienol synthesis, consistent with *ZmHGGT1* playing a central role in determining natural variation in total tocotrienols levels. Arogenate is a key intermediate leading to phenylalanine or tyrosine via prephenate/arogenate dehydratase or prephenate dehydrogenase, respectively (Maeda and Dudareva 2012). The association of a prephenate/arogenate dehydratase parolog with total tocotrienols levels suggests reduction in its activity makes more arogenate available for tyrosine synthesis, the head group precursor for all tocochromanols. The likely reason for association only with tocotrienol levels is because of the endosperm specific expression of the gene (Sekhon *et al.* 2011), a tissue greatly enriched in tocotrienols. The same is likely true of the stronger association of *ZmVTE1* with tocotrienols. *ZmVTE1* is expressed at high constitutive levels in tocopherol-rich embryo tissue and unlikely to be limiting in the embryo, while in the tocotrienol-rich endosperm it is expressed at much lower levels and more likely to be a limiting activity in this tissue across the association panel (Grams *et al.* 1970; Weber 1987).

Our study identified a *ZmVTE4* haplotype that was associated with high levels of αT (15.95 ± 3.90 μg·g^-1^) in maize grain, the tocochromanol compound with the highest vitamin E activity (DellaPenna and Mène-Saffrané 2011). Selection on this most favorable *ZmVTE4* haplotype comprised of the three MLMM-identified SNPs was estimated to produce an unprecedented maximal 5.76-fold increase of αT levels in maize grain, nearly twice that reported in a previous association study of the locus (Li *et al.* 2012). This haplotype of *ZmVTE4* occurred at an 11% frequency in the maize association panel and importantly was distributed equally between temperate and tropical germplasm. Therefore, some of the inbreds containing this haplotype can be readily used as donors for marker-assisted selection within their respective germplasm type, be it temperate or tropical. Inbreds with best adaptation to the targeted breeding region can therefore be identified and would likely not have the issues of linkage drag of unadapted alleles surrounding the *ZmVTE4* locus. In this study we have also reported the first association of loci with natural variation for tocotrienol levels in maize grain: *ZmVTE1*, *ZmHGGT1*, and one of at least four prephenate dehydratase paralogs. Importantly, these three novel associations provide a foundation for tocotrienol biofortification in maize and can provide the foundation for producing grain that balances enhanced antioxidant capacity with vitamin E activity considerations. In general, the observed low correlations between the individual tocopherol and tocotrienol compounds suggest that these traits can be independently manipulated genetically; thus. MAS of these and other yet to be identified loci could be potentially used to specifically modify the tocochromanol profile for optimal vitamin E and antioxidant contents within a single maize grain.

## Supplementary Material

Supporting Information
