# Supplementary material for: Genome-Wide Association Study and Pathway-Level Analysis of Tocochromanol Levels in Maize Grain
Source: G3 (Bethesda). 2013 Aug 1;3(8):1287–99. doi: 10.1534/g3.113.006148 (PMC3737168; doi:10.1534/g3.113.006148)
Supplement: Supporting Information [file supp_3_8_1287__index.html]

Genome-Wide Association Study and Pathway-Level Analysis of Tocochromanol Levels in Maize Grain — Supporting Information 

# Genome-Wide Association Study and Pathway-Level Analysis of Tocochromanol Levels in Maize Grain

## Supporting Information for Lipka *et al.*, 2013

**Files in this Data Supplement:**

- Supporting Information - Figures S1-S5 and Tables S1-S7 (PDF, 5.7 MB)
- Figure S1 - Genome-wide association study of 20 tocochromanol grain traits (PDF, 2.9 MB)
- Figure S2 - Genome-wide association study (GWAS) for the ratio of α- to γ-tocopherol (αT/γT) in maize grain (PDF, 341 KB)
- Figure S3 - Genome-wide association study (GWAS) for the ratio of γ- to (γ- + α-tocopherols) [γT/(γT+αT)] in maize grain (PDF, 347 KB)
- Figure S4 - Genome-wide association study (GWAS) for the ratio δ- to α-tocopherol (δT/αT) in maize grain (PDF, 345 KB)
- Figure S5 - Genome-wide association study (GWAS) for δ-tocotrienol (δT3) in maize grain (PDF, 397 KB)
- Table S1 - Best linear unbiased predictors (BLUPs) of the 20 tocochromanol traits used for the genome-wide association study (GWAS) and pathway level analysis (PDF, 578 KB)
- Table S2 - Genomic information for the 60 *a priori* candidate genes (PDF, 365 KB)
- Table S3 - Correlation matrix for untransformed BLUPs of the 20 tocochromanol grain traits (PDF, 314 KB)
- Table S4 - Statistically significant results from the genome-wide association study of 20 tocochromanol grain traits (PDF, 572 KB)
- Table S5 - Multi-locus mixed-model (MLMM) results from an analysis of αT, αT/γT, γT/(γT+αT), δT/αT, δT3/(γT3+αT3), and δT3 (PDF, 361 KB)
- Table S6 - Haplotype effects of three ZmVTE4 SNPs identified with an optimal multi-locus mixed model (MLMM) for αT/γT, δT/αT, and γT/(γT+αT) (PDF, 423 KB)
- Table S7 - Statistically significant results from the candidate gene association study of 20 tocochromanol grain traits (a) without and with none of the SNPs, (b) with the three SNPs within ZmVTE4, (c) with the two SNPs in the region including ZmVTE1, and (d) with all five SNPs identified in the multi-locus mixed-model (MLMM) analysis included as covariates (PDF, 1 MB)
