# Supplementary material for: Genome-Wide Association Study and Pathway-Level Analysis of Tocochromanol Levels in Maize Grain
Source: G3 (Bethesda). 2013 Aug 1;3(8):1287–99. doi: 10.1534/g3.113.006148 (PMC3737168; doi:10.1534/g3.113.006148)
Supplement: Supporting Information [file supp_g3.113.006148_006148SI.pdf]

G3: Genes | Genomes | Genetics

Genome-Wide Association Study and Pathway Level Analysis of Tocochromanol Levels in Maize Grain

Alexander E. Lipka*[,1], Michael A. Gore[§,2], Maria Magallanes-Lundback[†], Alex Mesberg[†], Haining Lin[‡,3] Tyler Tiede[**], Charles Chen[§§,4], C. Robin Buell[‡], Edward S. Buckler*[,§§,††], Torbert Rocheford**, and Dean DellaPenna[†]

*United States Department of Agriculture – Agricultural Research Service (USDA-ARS), Robert W. Holley Center for Agriculture and Health, Ithaca, NY, 14853
[§]United States Department of Agriculture – Agricultural Research Service (USDA-ARS), U.S. Arid Land Agricultural Research Center, Maricopa, AZ, 85138
[†]Department of Biochemistry and Molecular Biology, Michigan State University, East Lansing, MI, 48824
[‡]Department of Plant Biology, Michigan State University, East Lansing, MI, 48824
**Department of Agronomy, Purdue University, West Lafayette, IN, 47907
[§§]Institute for Genomic Diversity, Cornell University, Ithaca, NY, 14853
[††]Department of Plant Breeding and Genetics, Cornell University, Ithaca, NY, 14853

DOI: 10.1534/g3.113.006148

Footnotes:

1 A. E. Lipka and M. A. Gore contributed equally to this work

2 Current Address: Department of Plant Breeding and Genetics, Cornell University, Ithaca, NY, 14853

3 Current Address: DuPont Pioneer, Johnston, IA, 50131

4 Current Address: International Maize and Wheat Improvement Center (CIMMYT), Texcoco, Mexico

Figure S1 Genome wide association study of 20 tocochromanol grain traits. Manhattan Plots (Gibson, 2010) of association results from a unified mixed model analysis of each tocochromanol grain trait. Negative $\log_{10}$-transformed *P*-values (y-axis) from a GWAS are plotted against physical position (B73 RefGen_v2) on each of 10 chromosomes. Chromosomes are alternatingly colored.

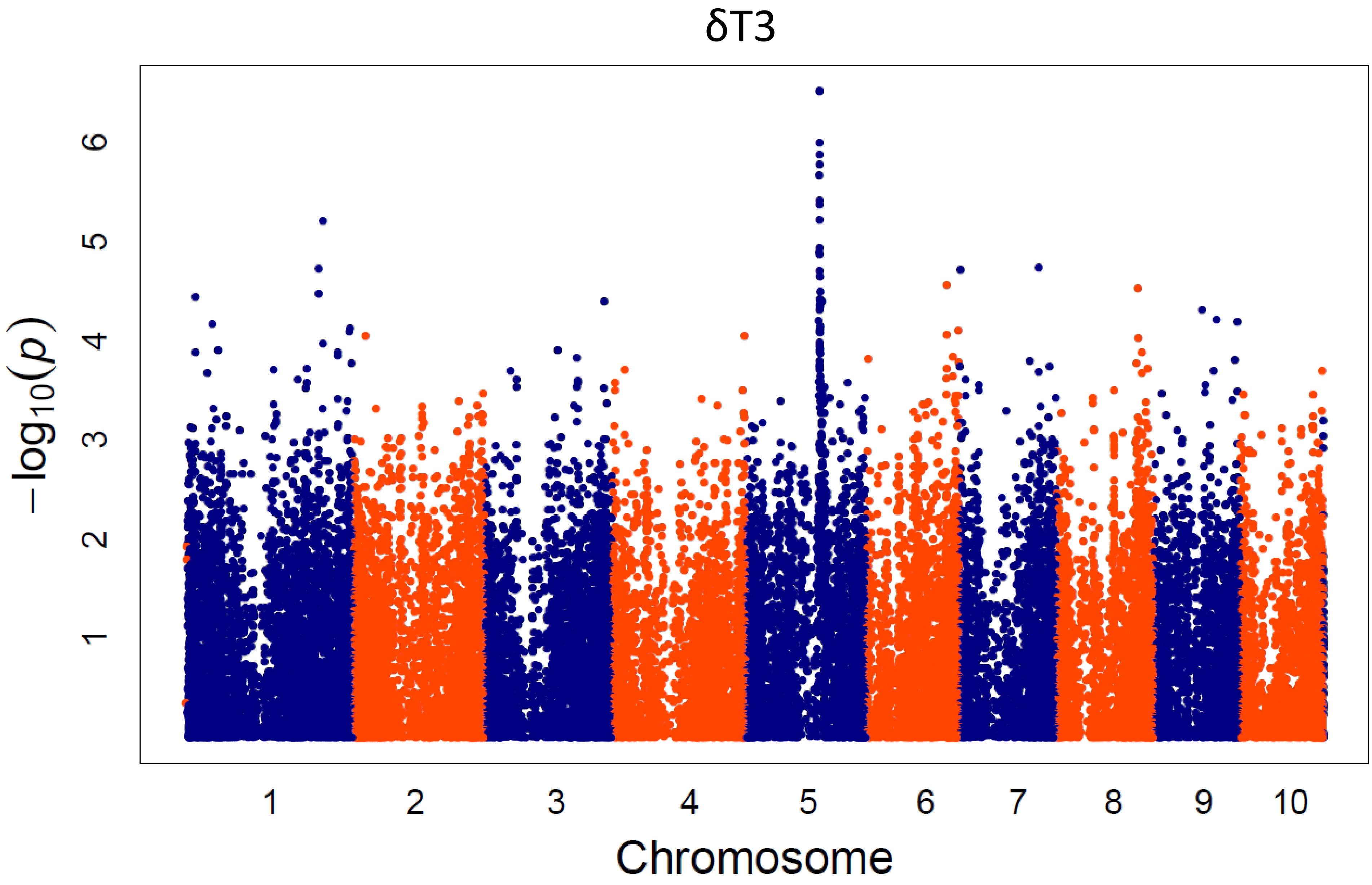
δT3
−log10(p)
1
2
3
4
5
6
1
2
3
4
5
6
7
8
9
10
Chromosome

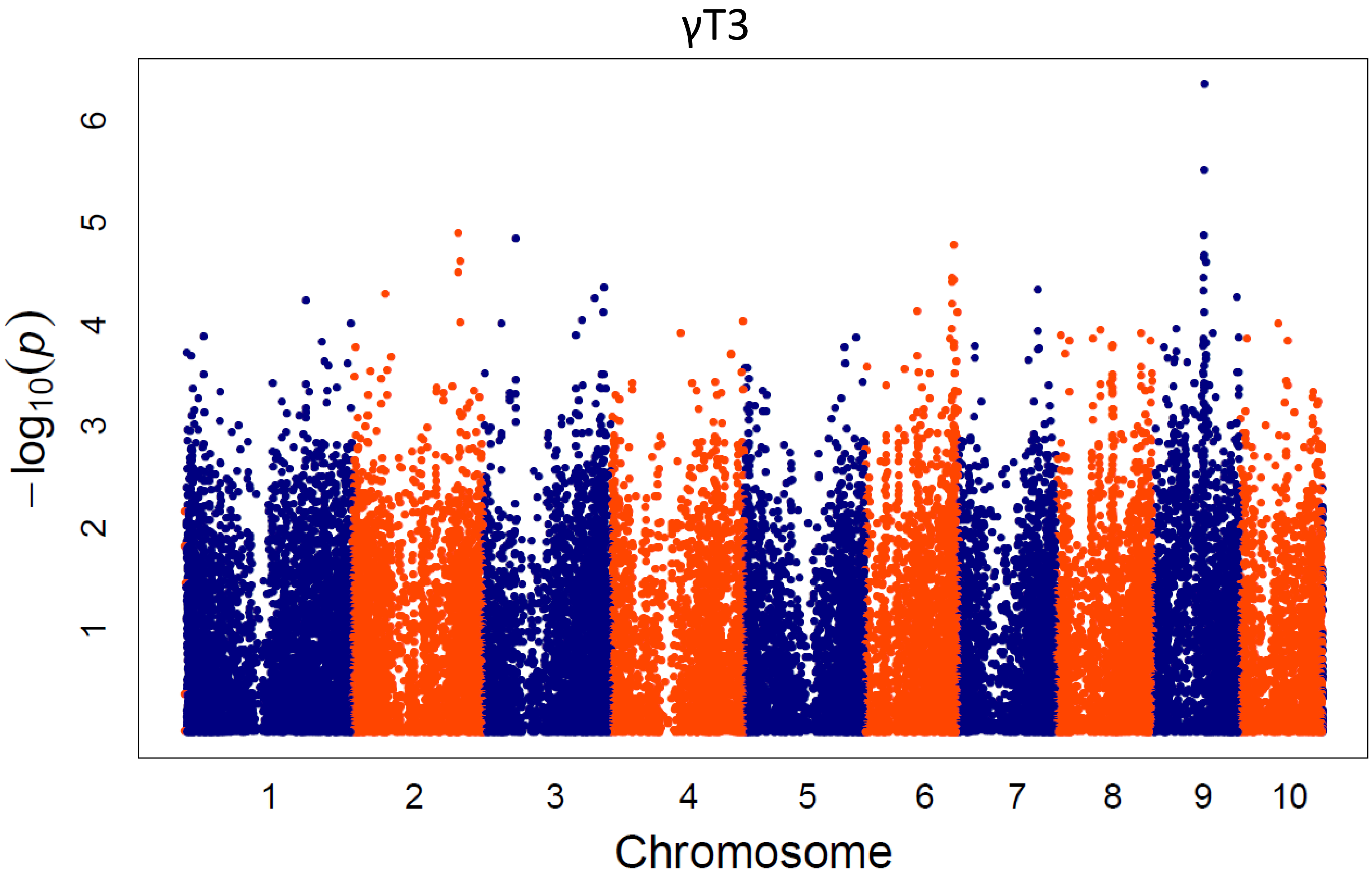
γT3
6
5
4
3
2
1
−log10(p)
1
2
3
4
5
6
7
8
9
10
Chromosome

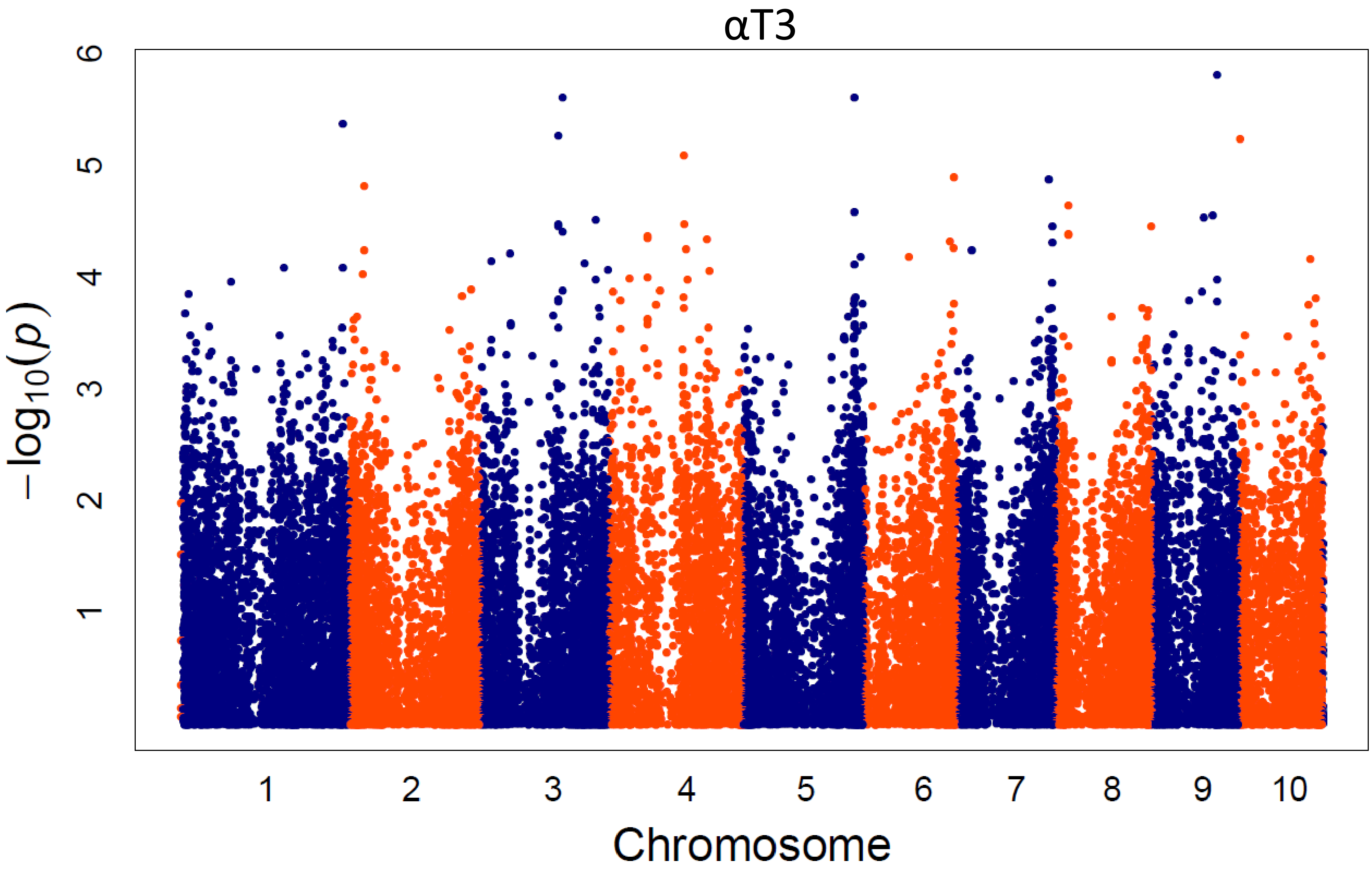
αT3
−log10(p)
6
5
4
3
2
1
1
2
3
4
5
6
7
8
9
10
Chromosome

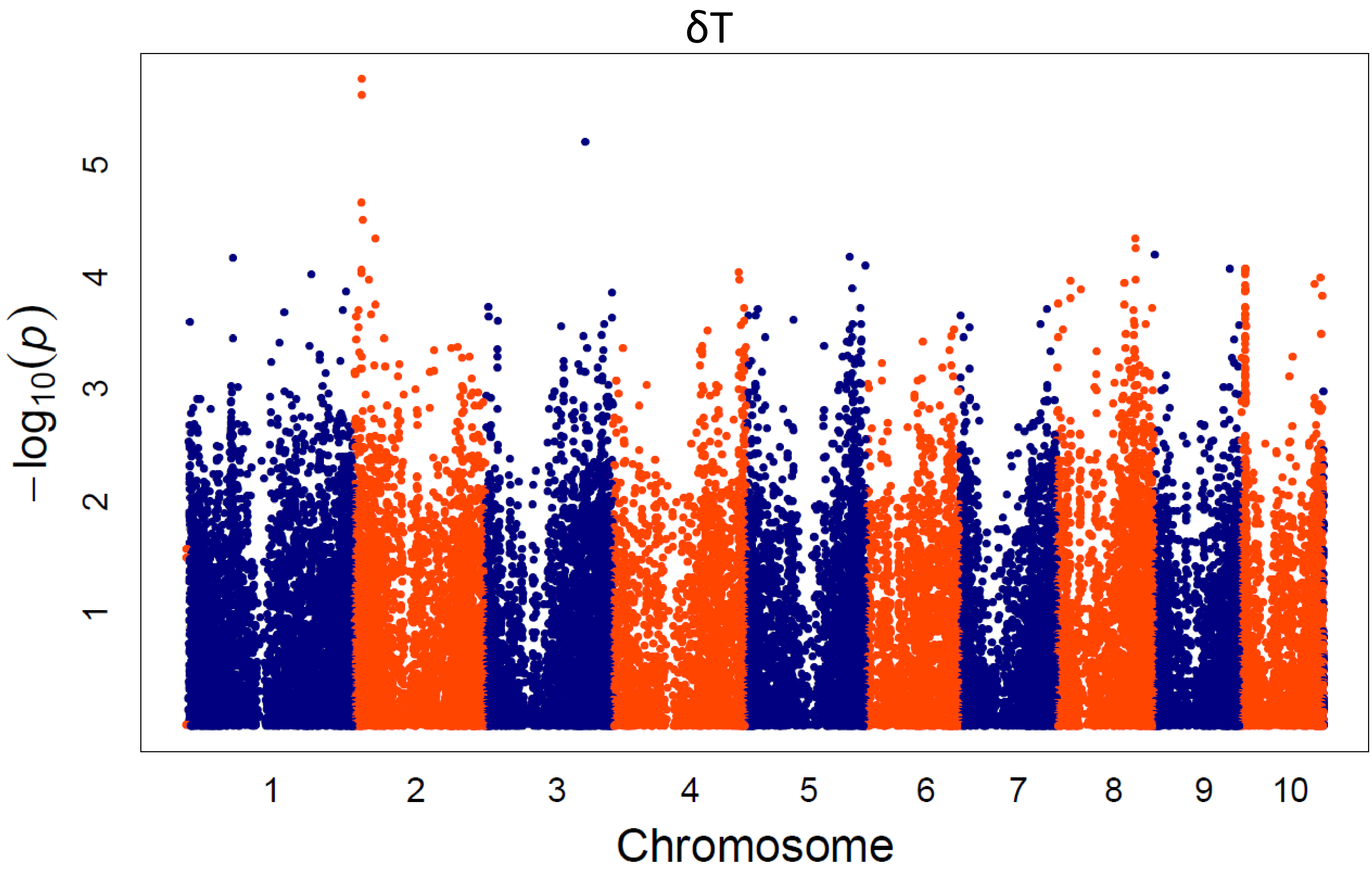

δT
−log10(p)
1
2
3
4
5
1
2
3
4
5
6
7
8
9
10
Chromosome

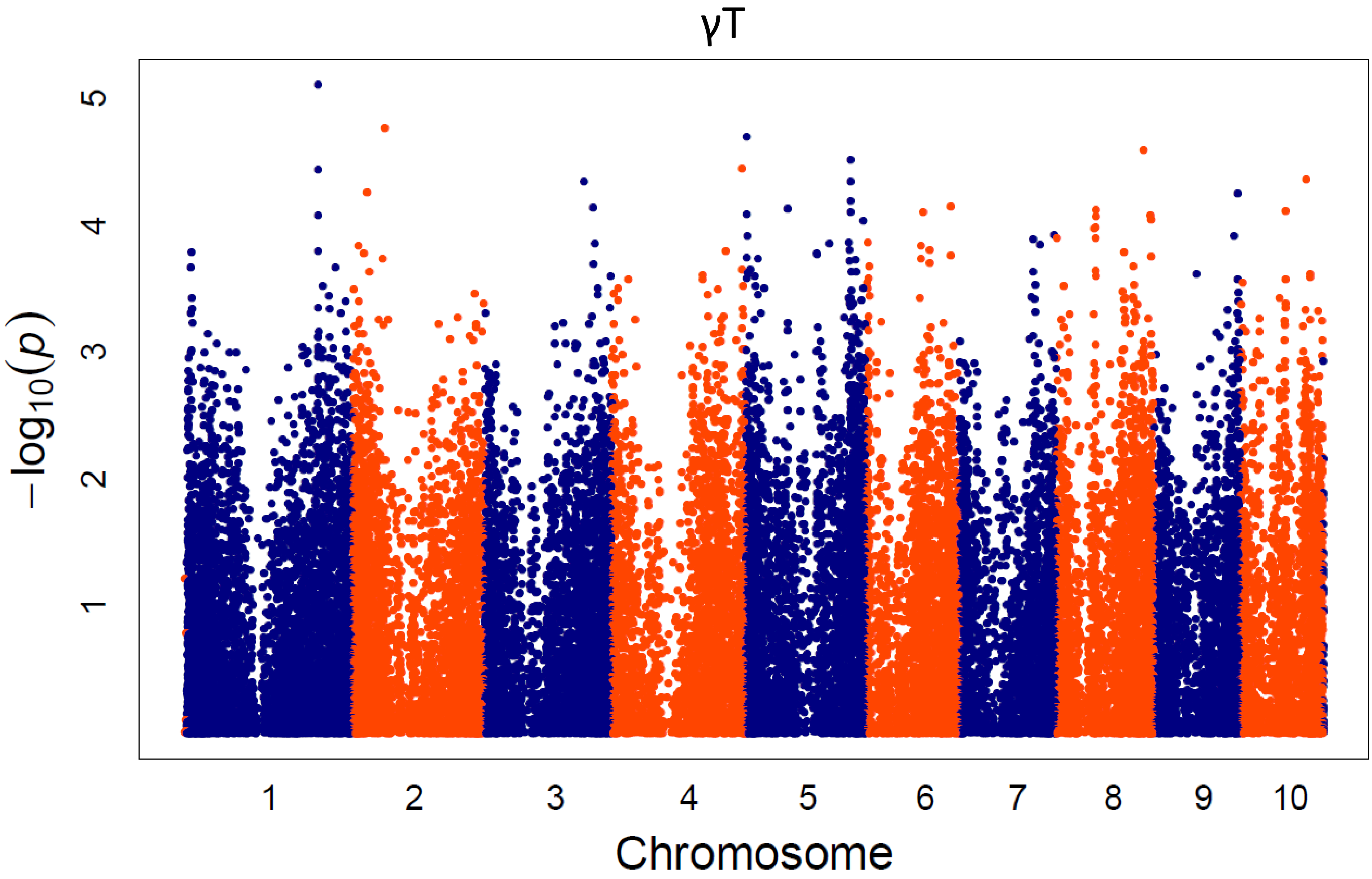
γT
5
4
3
2
1
$-\log_{10}(p)$
1
2
3
4
5
6
7
8
9
10
Chromosome

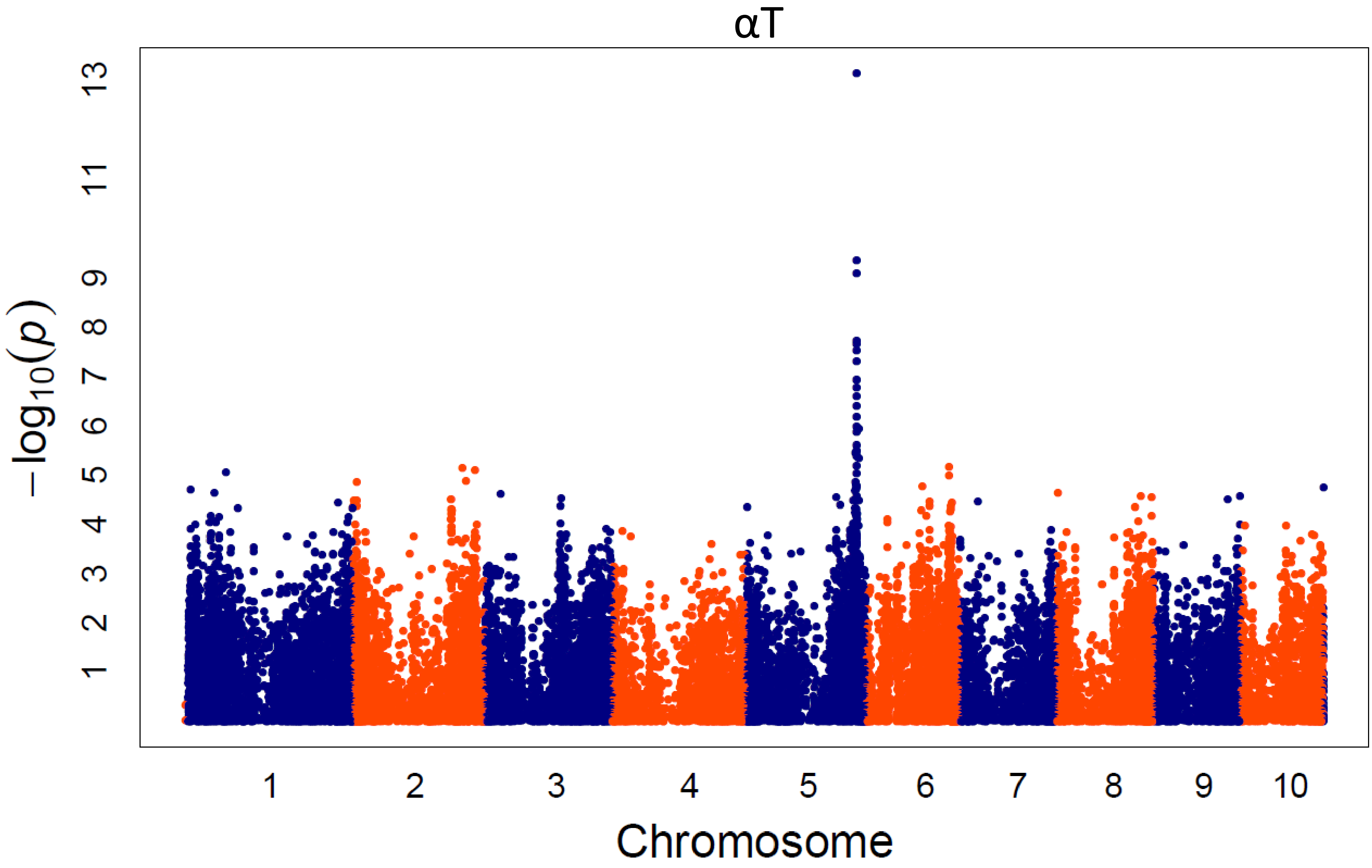
αT
13
11
9
8
7
6
5
4
3
2
1
$-\log_{10}(p)$
1
2
3
4
5
6
7
8
9
10
Chromosome

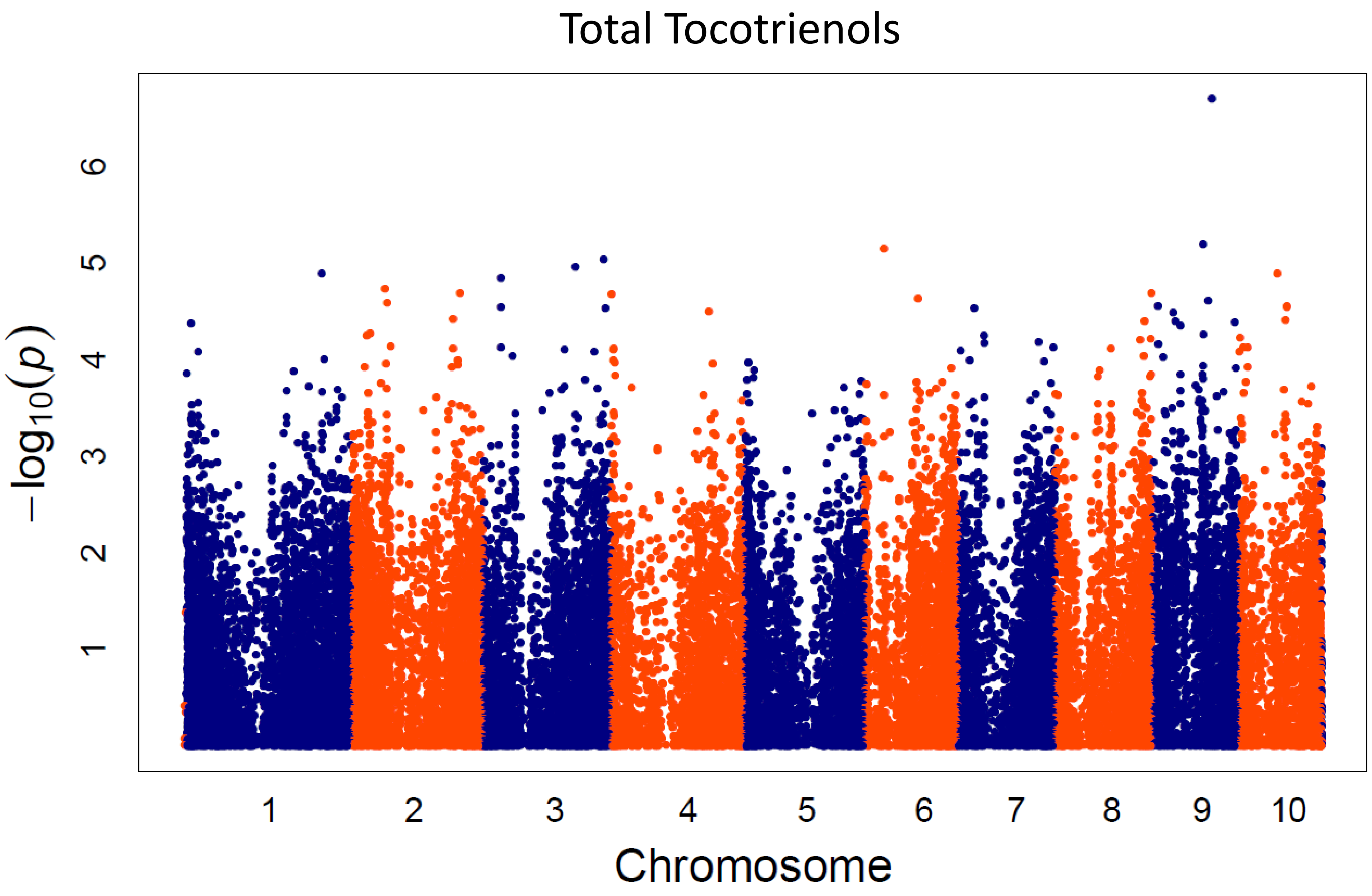
Total Tocotrienols
−log10(p)
1
2
3
4
5
6
1
2
3
4
5
6
7
8
9
10
Chromosome

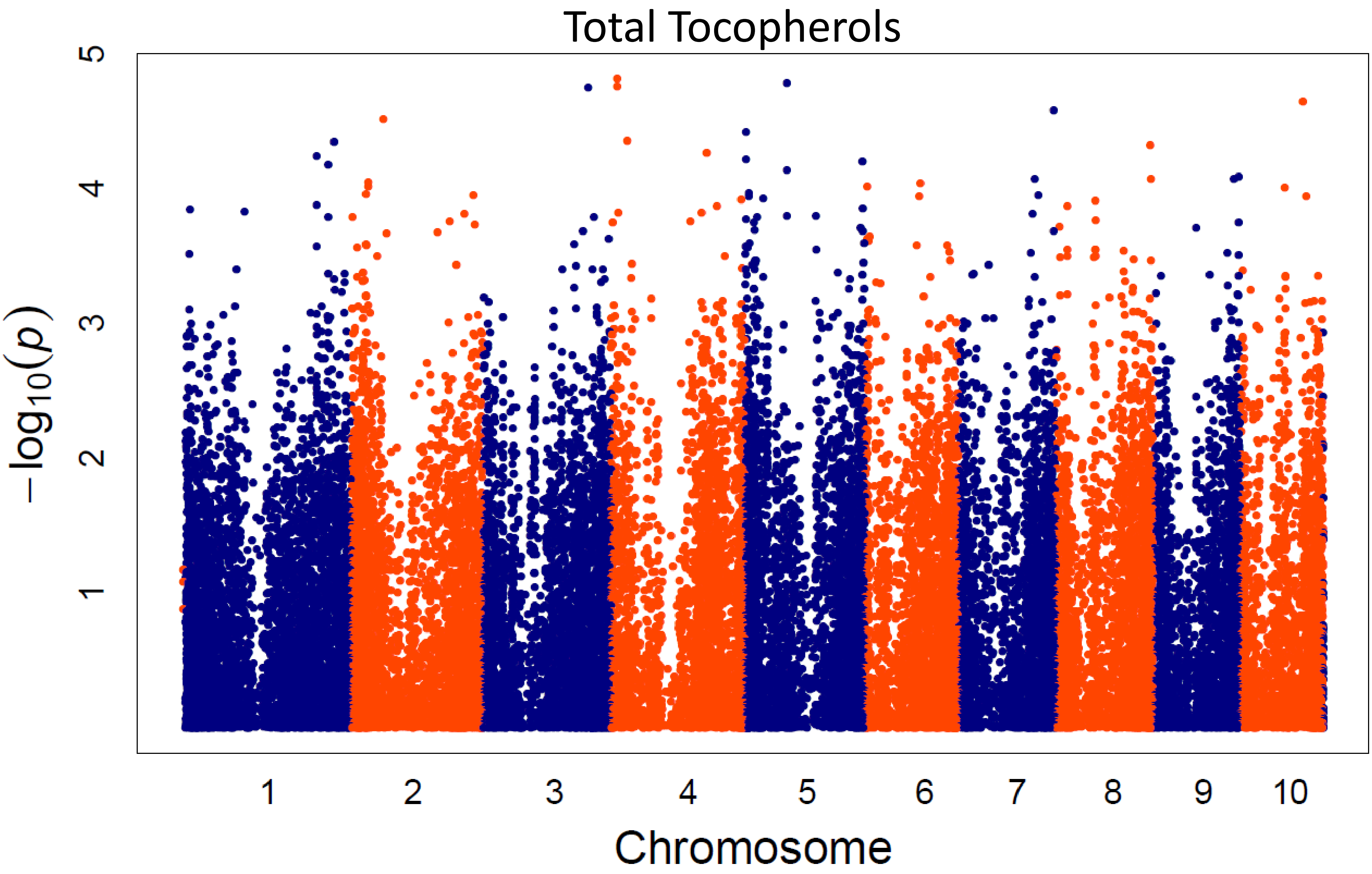
Total Tocopherols
5
4
3
2
1
$-\log_{10}(p)$
1
2
3
4
5
6
7
8
9
10
Chromosome

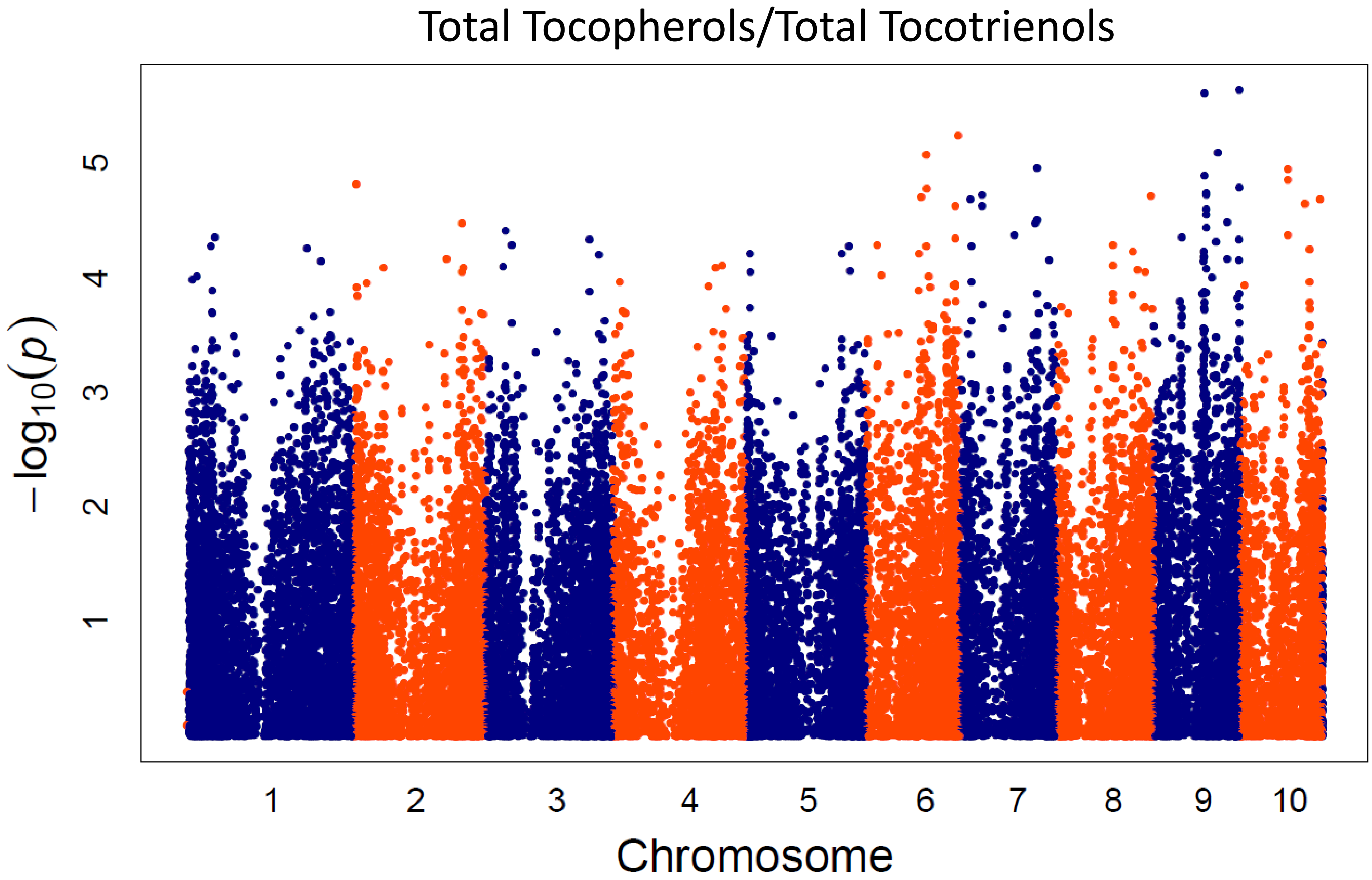

Total Tocopherols/Total Tocotrienols
$-\log_{10}(p)$
1
2
3
4
5
1 2 3 4 5 6 7 8 9 10
Chromosome

Total Tocochromanols

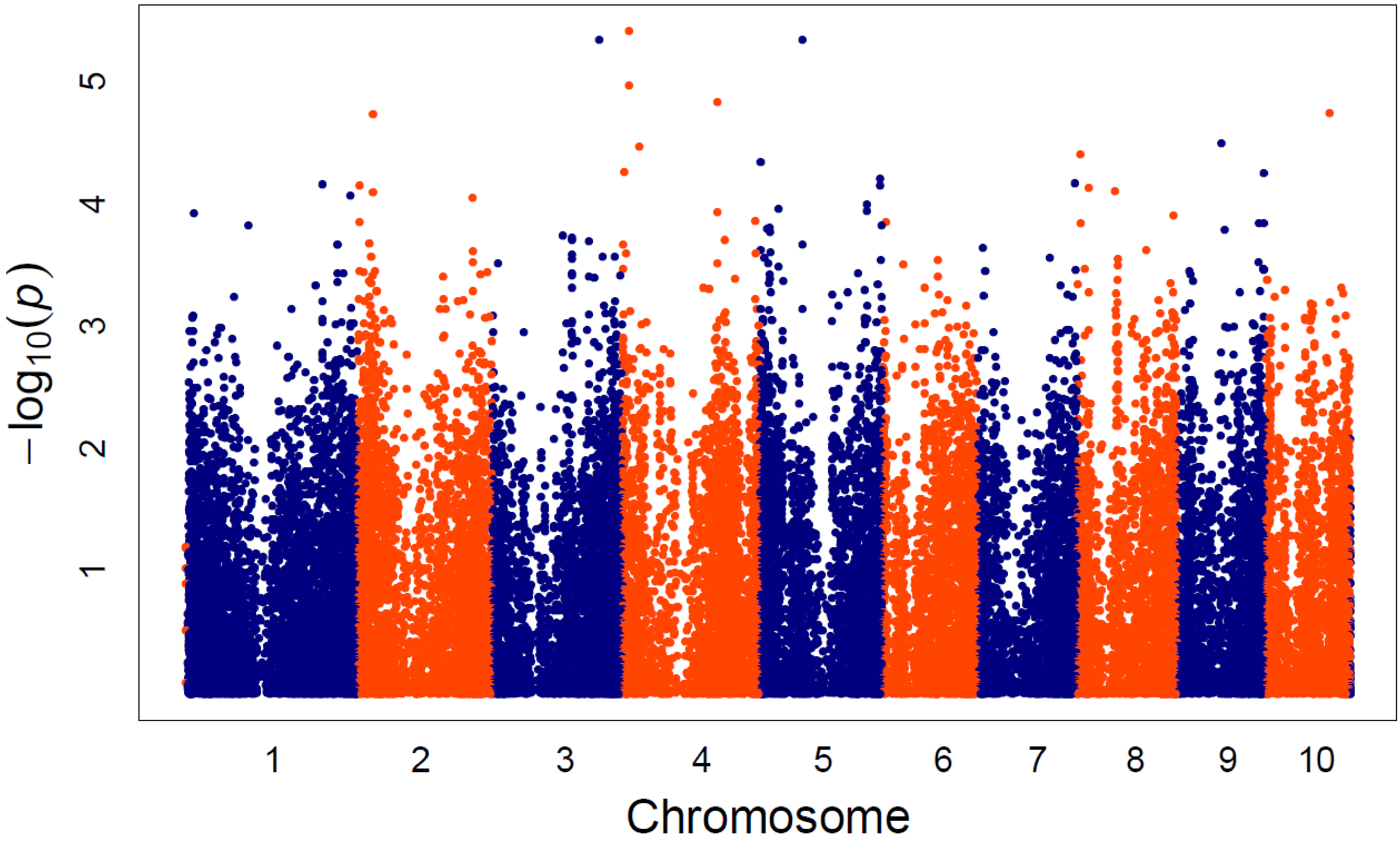

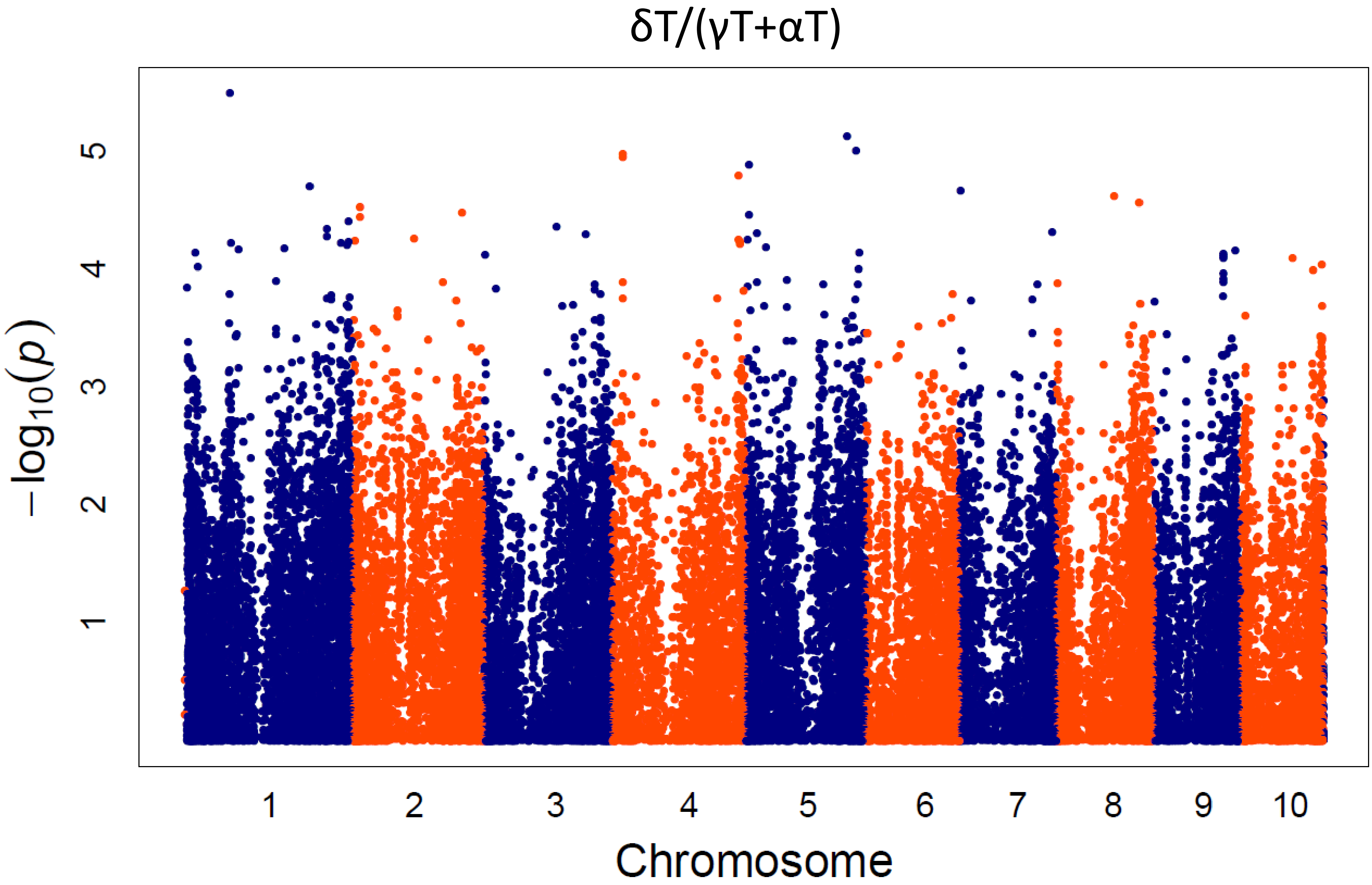
δT/(γT+αT)
−log10(p)
1
2
3
4
5
1
2
3
4
5
6
7
8
9
10
Chromosome

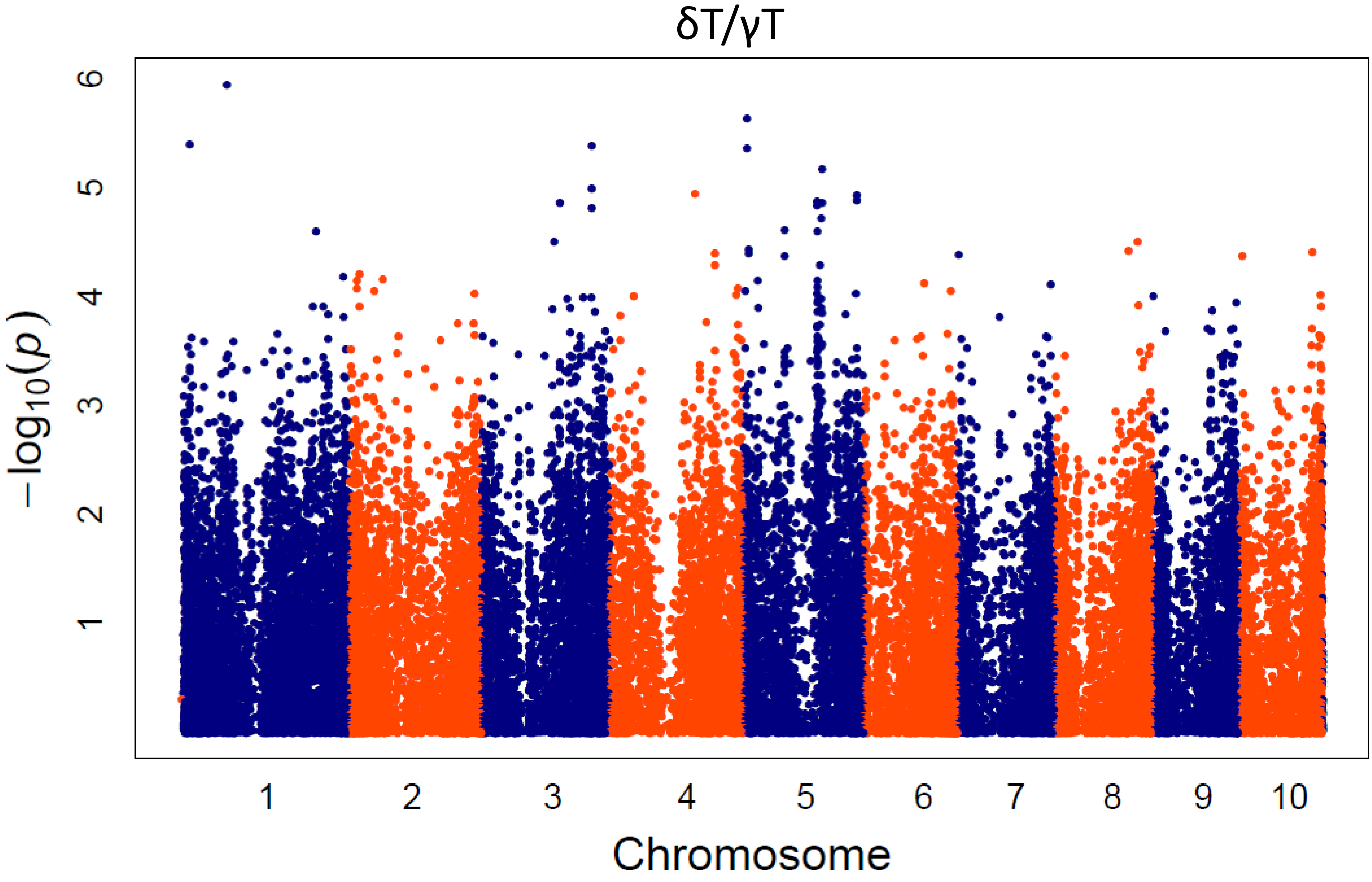

δT/γT
−log10(p)
1
2
3
4
5
6
1
2
3
4
5
6
7
8
9
10
Chromosome

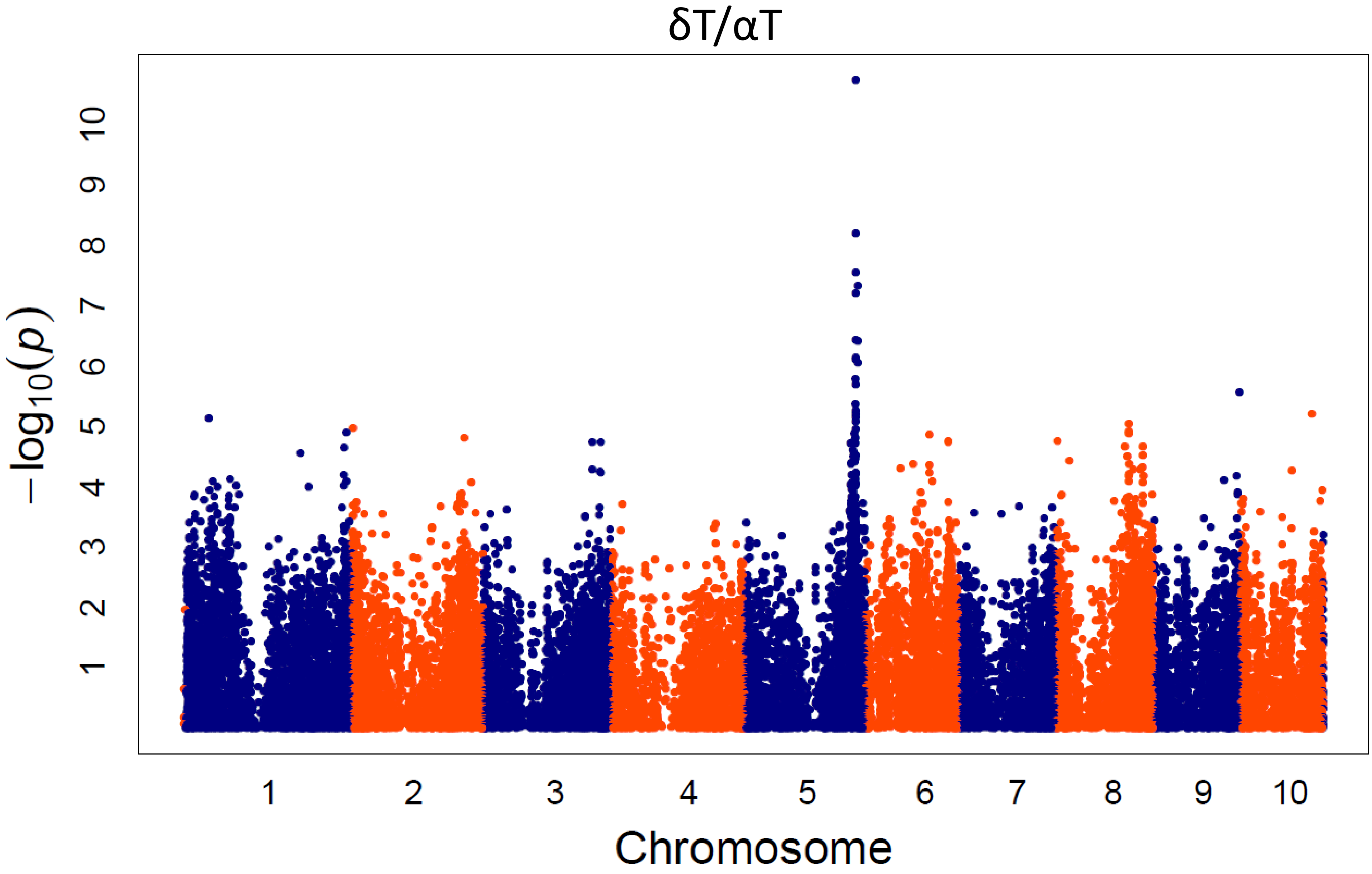
δT/αT
$-\log_{10}(p)$
1
2
3
4
5
6
7
8
9
10
Chromosome

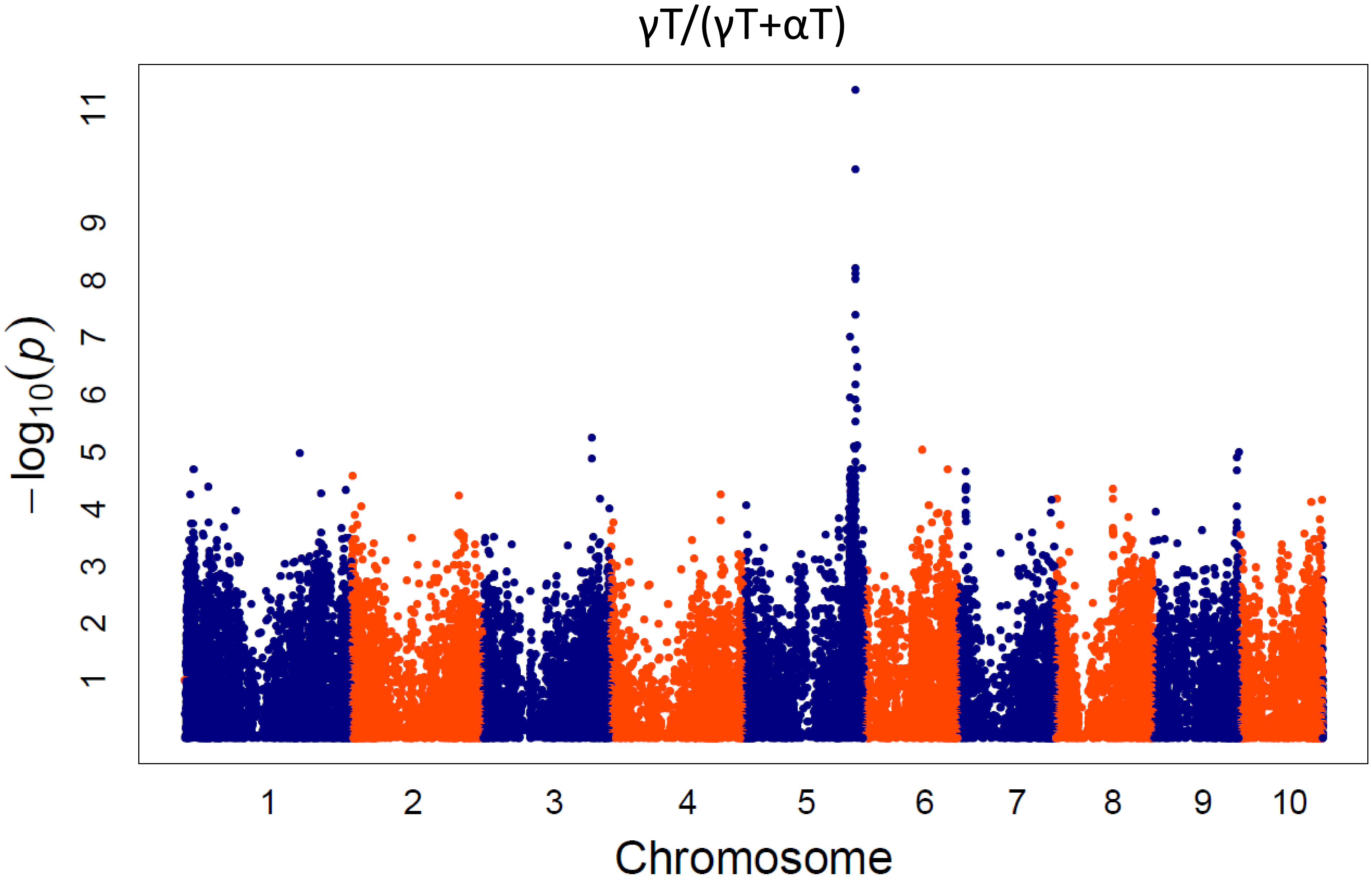
γT/(γT+αT)
−log10(p)
1
2
3
4
5
6
7
8
9
11
1
2
3
4
5
6
7
8
9
10
Chromosome

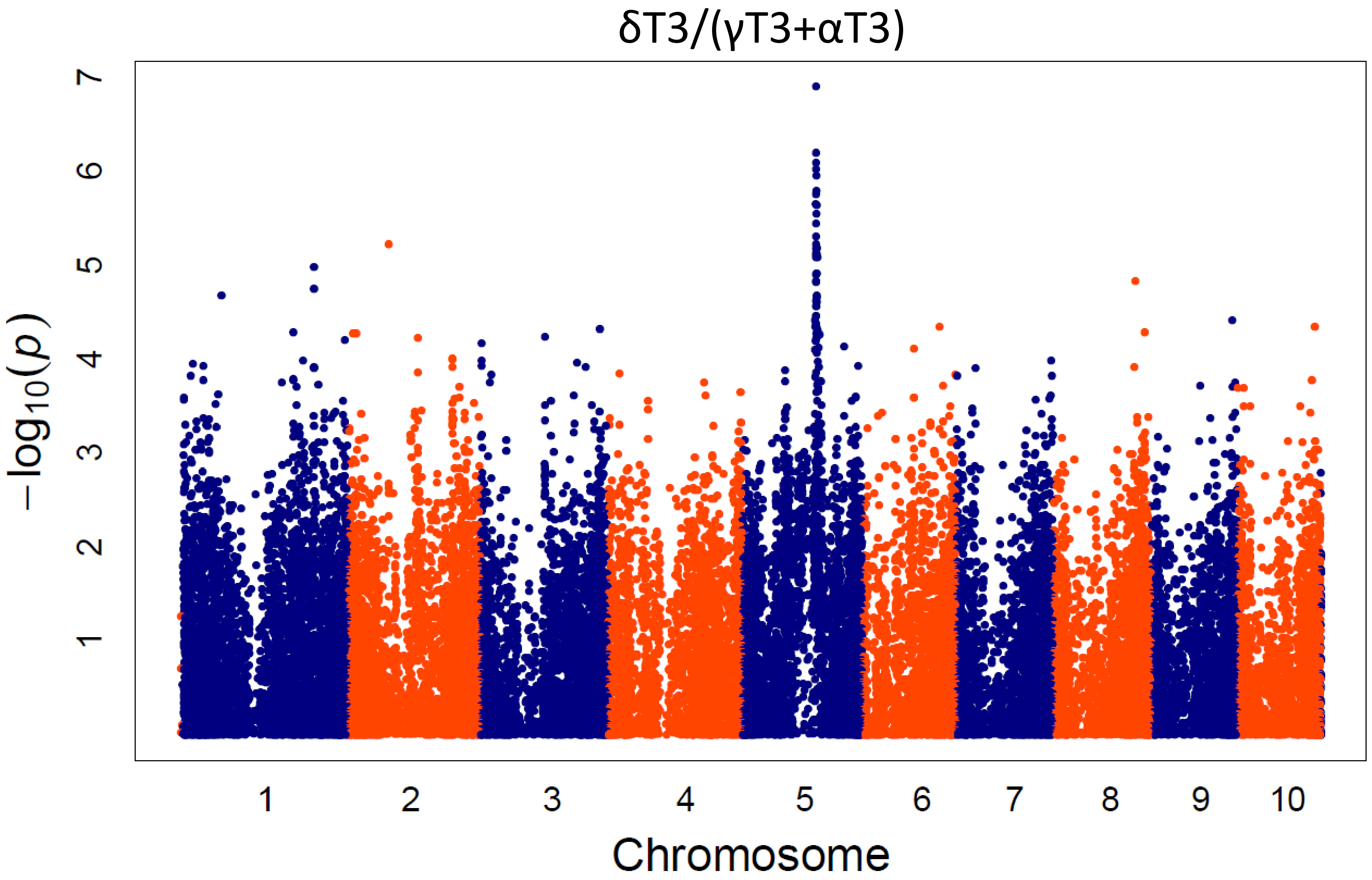
δT3/(γT3+αT3)
−log10(p)
1
2
3
4
5
6
7
1
2
3
4
5
6
7
8
9
10
Chromosome

δT3/γT3

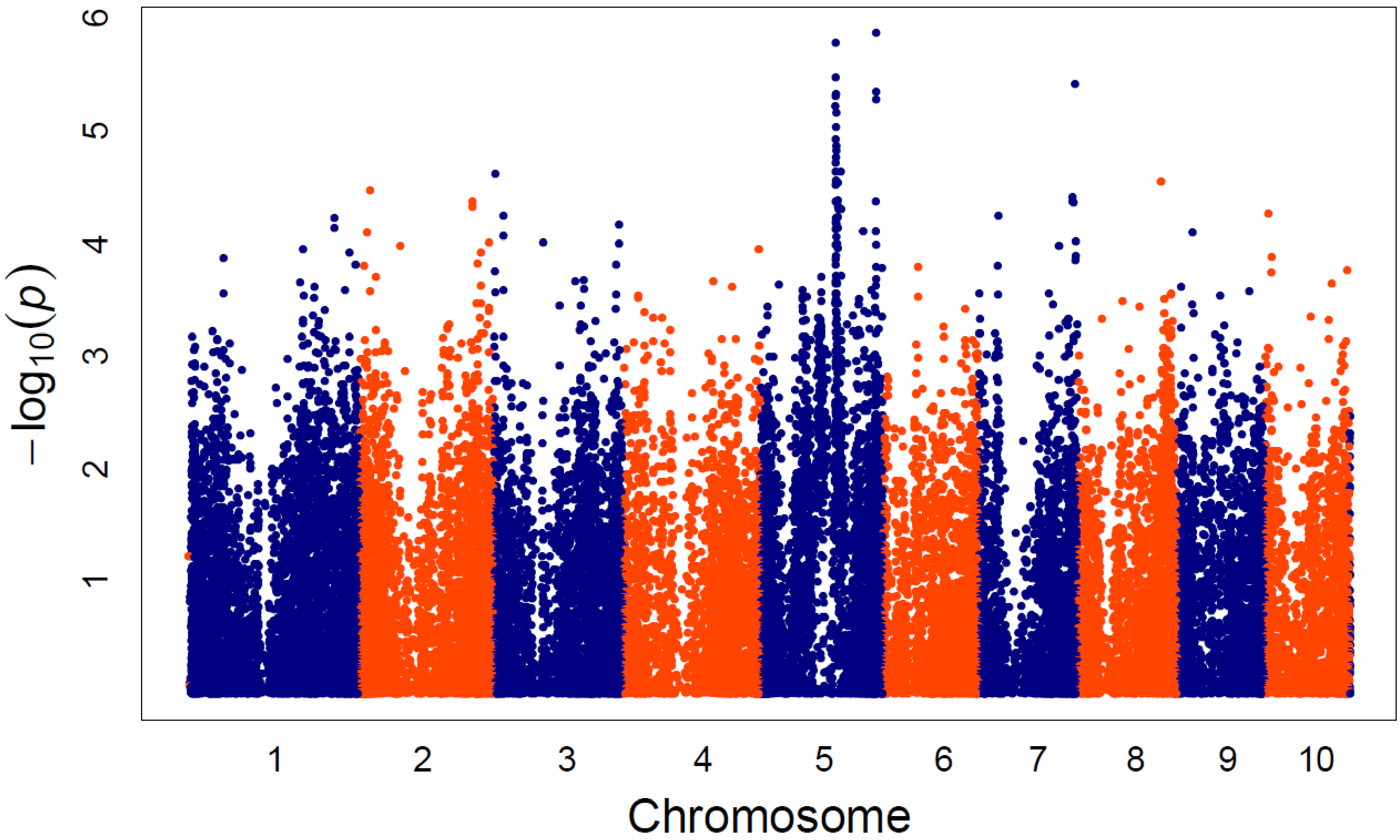

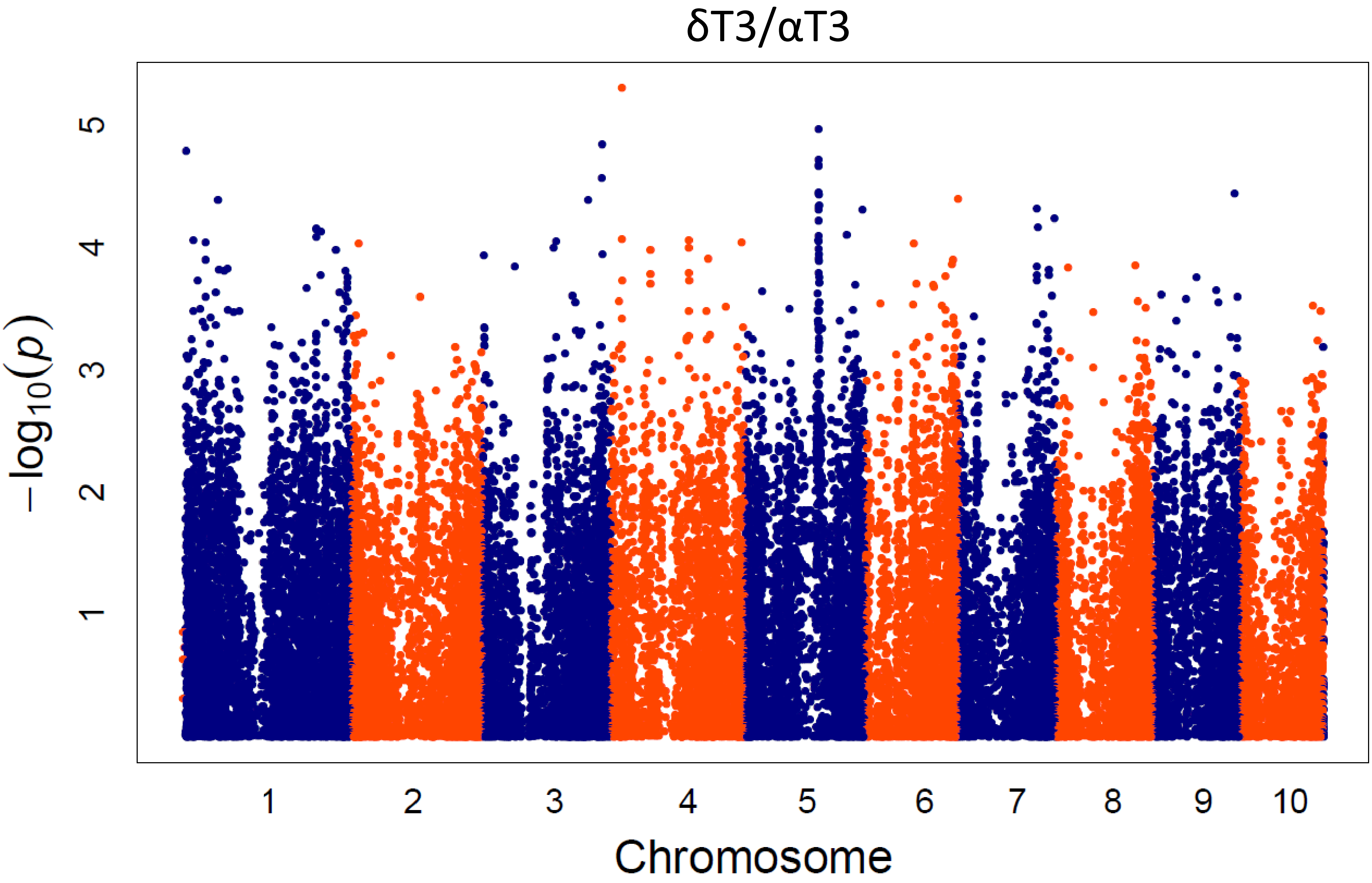
δT3/αT3
$-\log_{10}(p)$
1
2
3
4
5
Chromosome
1
2
3
4
5
6
7
8
9
10

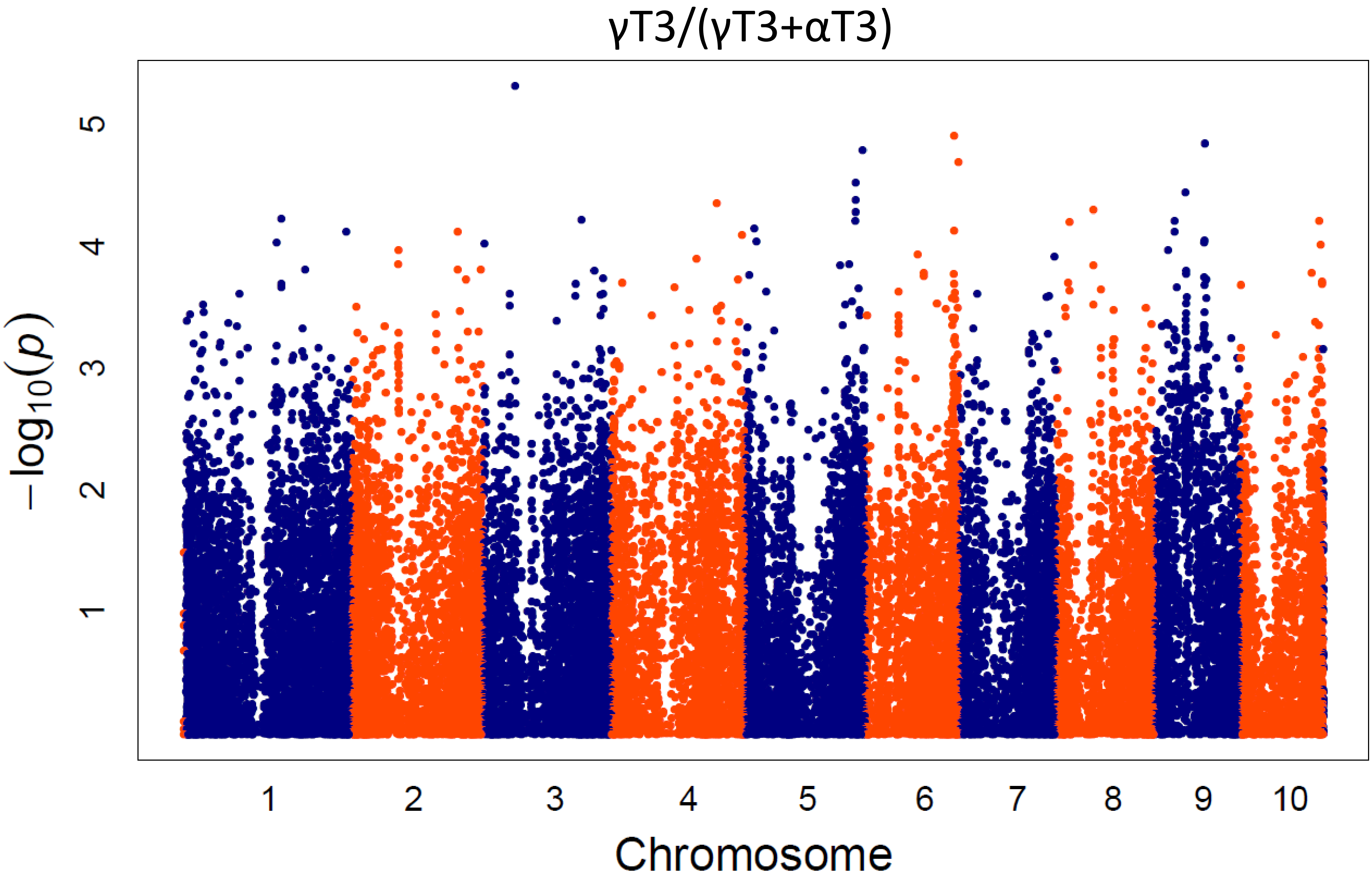
γT3/(γT3+αT3)
−log10(p)
1
2
3
4
5
1
2
3
4
5
6
7
8
9
10
Chromosome

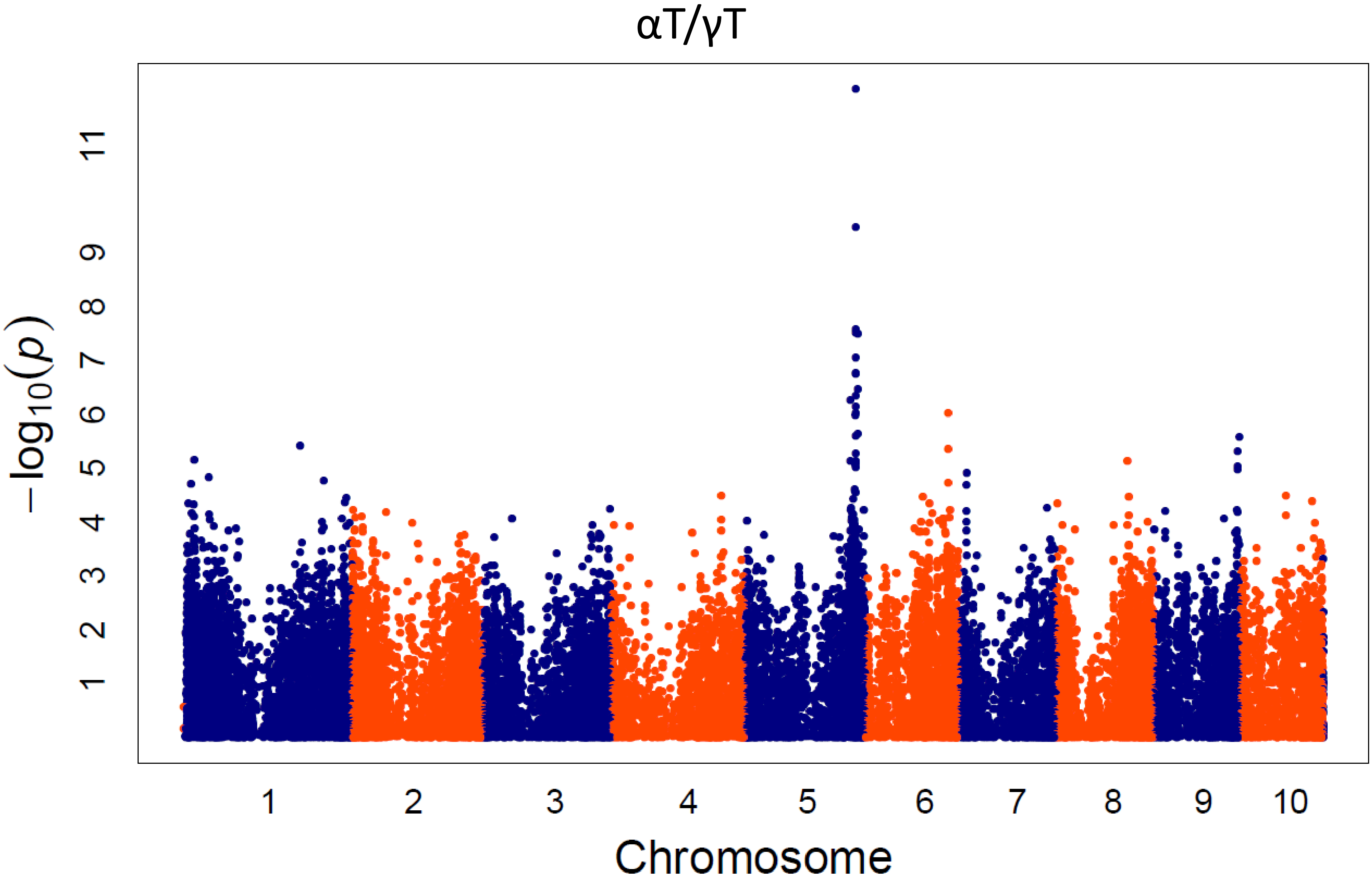
αT/γT
−log10(p)
1
2
3
4
5
6
7
8
9
11
1
2
3
4
5
6
7
8
9
10
Chromosome

αT3/γT3

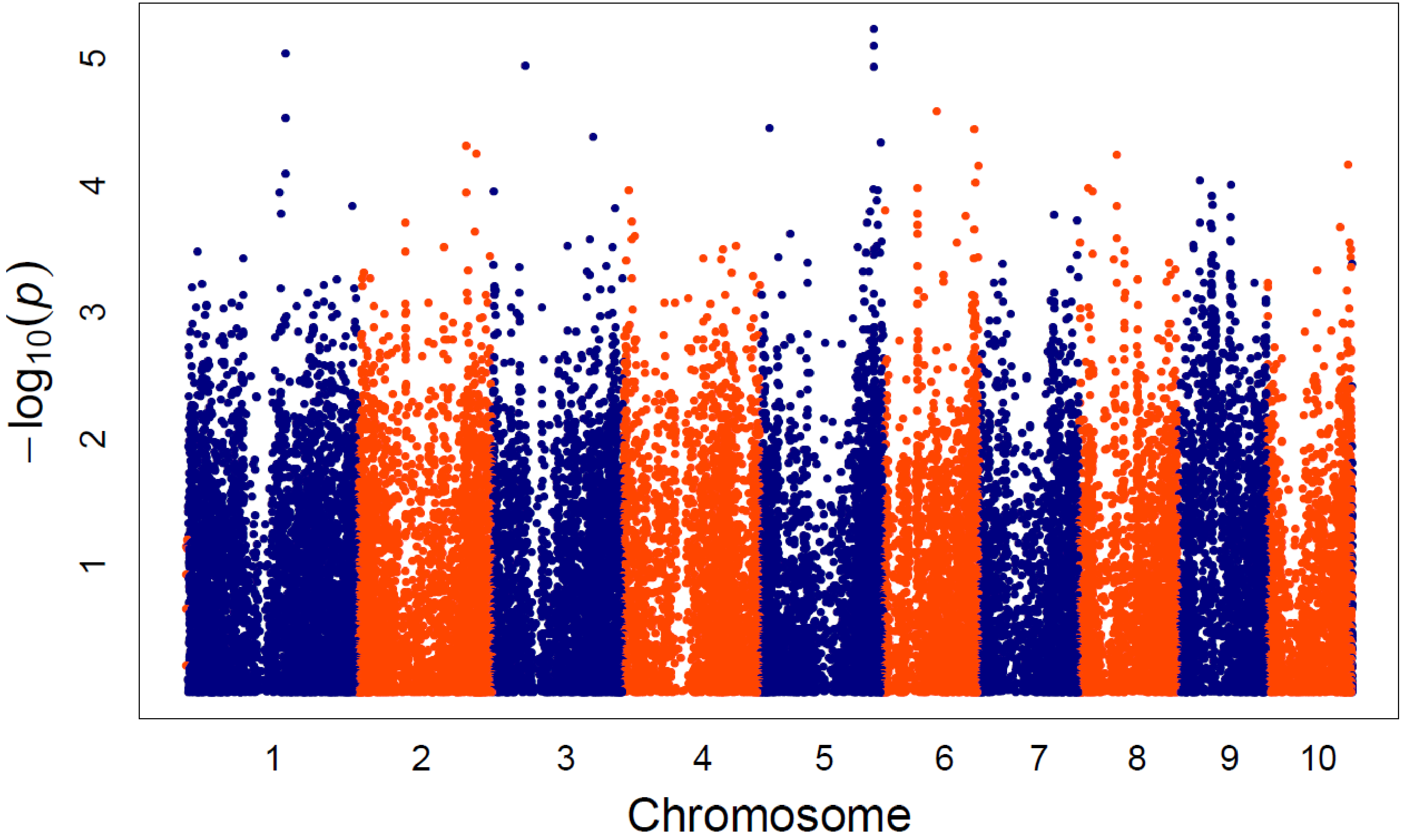

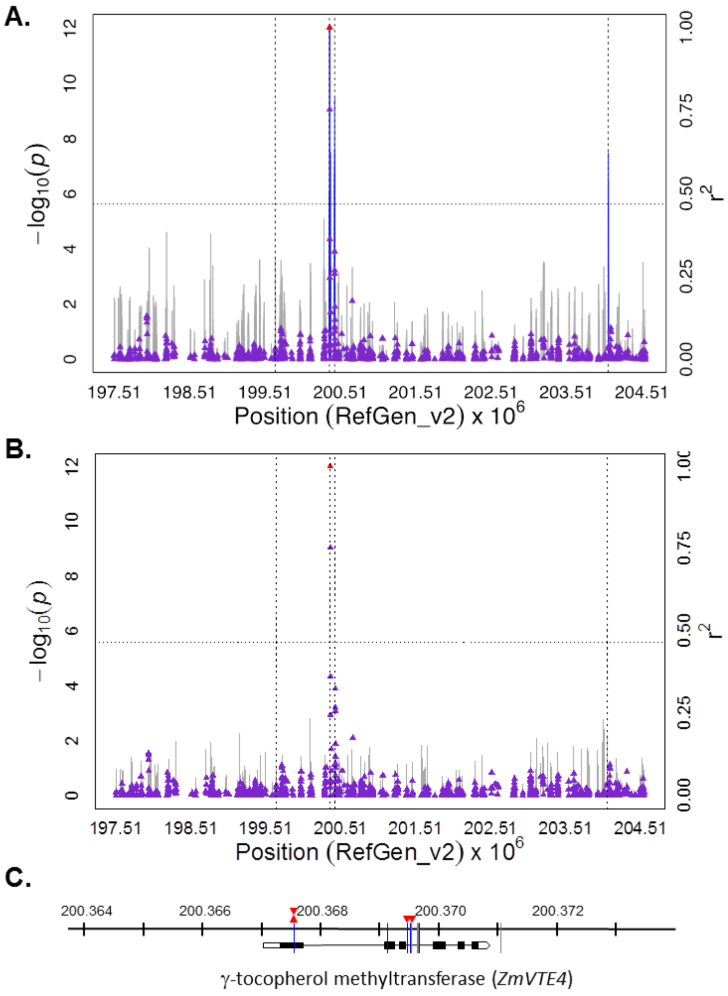

Figure S2 Genome-wide association study (GWAS) for the ratio of α- to γ-tocopherol (αT/γT) in maize grain. (A) Scatter plot of association results from a unified mixed model analysis of αT/γT and linkage disequilibrium (LD) estimates (r^2) across the *ZmVTE4* chromosome region. Negative $\log_{10}$-transformed *P*-values (left y-axis) from a GWAS for αT/γT and r^2 values (right y-axis) are plotted against physical position (B73 RefGen_v2) for a 7 Mb region on chromosome 5 that encompasses *ZmVTE4*. The blue vertical lines are $-\log_{10}$ *P*-values for SNPs that are statistically significant for αT/γT at 5% false discovery rate (FDR), while the gray vertical lines are $-\log_{10}$ *P*-values for SNPs that are non-significant at 5% FDR. Triangles are the r^2 values of each SNP relative to the peak SNP (indicated in red) at 200,367,532 bp. The black horizontal dashed line indicates the $-\log_{10}$ *P*-value of the least statistically significant SNP at 5% FDR. The black vertical dashed lines indicate the positions of four genes (from left to right): a WYRKY transcription factor (GRMZM5G823157), *ZmVTE4* (GRMZM2G035213), a pentatricopeptide repeat-containing protein (GRMZM2G325019), and an amino acid permease (GRMZM2G161641). (B) Scatter plot of association results from a conditional unified mixed model analysis of αT/γT and LD estimates (r^2) across the *ZmVTE4* chromosome region, as in (A). The three SNPs (ss196416269, S5_200369534, and S5_200369481) from the optimal multi-locus mixed model (MLMM) model were included as covariates in the unified mixed model to control for the *ZmVTE4* effect. (C) Gene model diagram for *ZmVTE4* with αT/γT associated SNPs. Blue vertical lines indicate the physical position (RefGen_v2) of SNPs within +/- 3 kb of the open reading frame start or stop position for *ZmVTE4* that are significantly associated with αT/γT at 5% FDR. Significant SNPs at 10% FDR are shown as gray vertical lines. The peak SNP is indicated by a red triangle, while the three SNPs included in the optimal MLMM model are indicated by inverted red triangles.

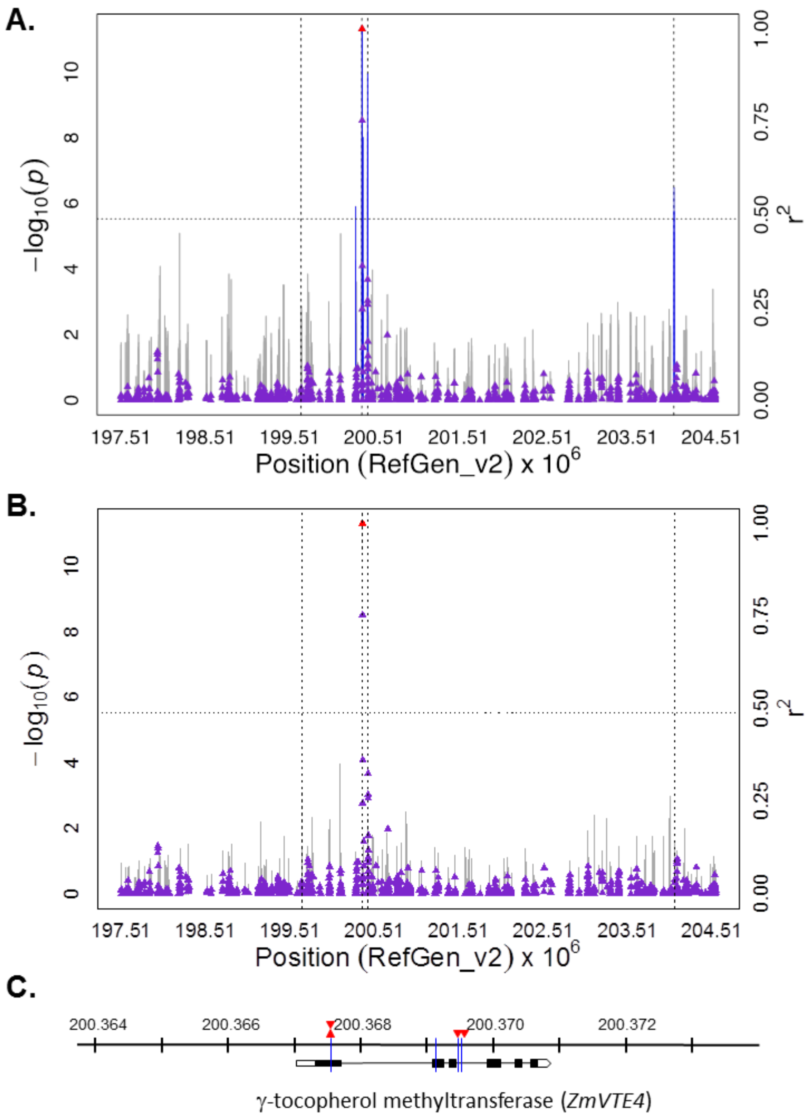

Figure S3 Genome-wide association study (GWAS) for the ratio of γ- to (γ- + α-tocopherols) [γT/(γT+αT)] in maize grain. (A) Scatter plot of association results from a unified mixed model analysis of γT/(γT+αT) and linkage disequilibrium (LD) estimates (r^2) across the *ZmVTE4* chromosome region. Negative $\log_{10}$-transformed *P*-values (left y-axis) from a GWAS for γT/(γT+αT) and r^2 values (right y-axis) are plotted against physical position (B73 RefGen_v2) for a 7 Mb region on chromosome 5 that encompasses *ZmVTE4*. The blue vertical lines are $-\log_{10}$ *P*-values for SNPs that are statistically significant for γT/(γT+αT) at 5% false discovery rate (FDR), while the gray vertical lines are $-\log_{10}$ *P*-values for SNPs that are non-significant at 5% FDR. Triangles are the r^2 values of each SNP relative to the peak SNP (indicated in red) at 200,367,532 bp. The black horizontal dashed line indicates the $-\log_{10}$ *P*-value of the least statistically significant SNP at 5% FDR. The black vertical dashed lines indicate the positions of four genes (from left to right): a WYRKY transcription factor (GRMZM5G823157), *ZmVTE4* (GRMZM2G035213), a pentatricopeptide repeat-containing protein (GRMZM2G325019), and an amino acid permease (GRMZM2G161641). (B) Scatter plot of association results from a conditional unified mixed model analysis of γT/(γT+αT) and LD estimates (r^2) across the *ZmVTE4* chromosome region, as in (A). The three SNPs (ss196416269, S5_200369534, and S5_200369481) from the optimal multi-locus mixed model (MLMM) model were included as covariates in the unified mixed model to control for the *ZmVTE4* effect. (C) Gene model diagram for *ZmVTE4* with γT/(γT+αT) associated SNPs. Blue vertical lines indicate the physical position (RefGen_v2) of SNPs within +/- 3 kb of the open reading frame start or stop position for *ZmVTE4* that are significantly associated with γT/(γT+αT) at 5% FDR. Significant SNPs at 10% FDR are shown as gray vertical lines. The peak SNP is indicated by a red triangle, while the three SNPs included in the optimal MLMM model are indicated by inverted red triangles.

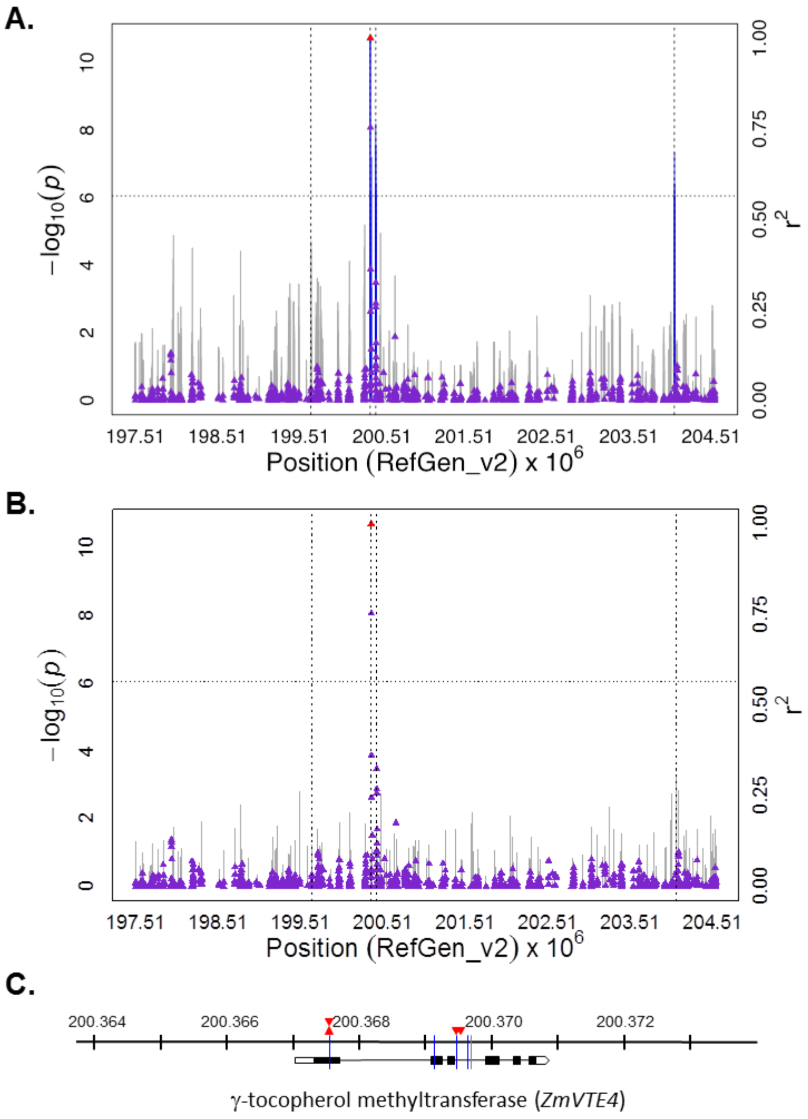

Figure S4 Genome-wide association study (GWAS) for the ratio δ- to α-tocopherol (δT/αT) in maize grain. (A) Scatter plot of association results from a unified mixed model analysis of δT/αT and linkage disequilibrium (LD) estimates (r^2) across the *ZmVTE4* chromosome region. Negative $\log_{10}$-transformed *P*-values (left y-axis) from a GWAS for δT/αT and r^2 values (right y-axis) are plotted against physical position (B73 RefGen_v2) for a 7 Mb region on chromosome 5 that encompasses *ZmVTE4*. The blue vertical lines are $-\log_{10}$ *P*-values for SNPs that are statistically significant for δT/αT at 5% false discovery rate (FDR), while the gray vertical lines are $-\log_{10}$ *P*-values for SNPs that are non-significant at 5% FDR. Triangles are the r^2 values of each SNP relative to the peak SNP (indicated in red) at 200,367,532 bp. The black horizontal dashed line indicates the $-\log_{10}$ *P*-value of the least statistically significant SNP at 5% FDR. The black vertical dashed lines indicate the positions of four genes (from left to right): a WYRKY transcription factor (GRMZM5G823157), *ZmVTE4* (GRMZM2G035213), a pentatricopeptide repeat-containing protein (GRMZM2G325019), and an amino acid permease (GRMZM2G161641). (B) Scatter plot of association results from a conditional unified mixed model analysis of δT/αT and LD estimates (r^2) across the *ZmVTE4* chromosome region, as in (A). The three SNPs (ss196416269, S5_200369534, and S5_200369481) from the optimal multi-locus mixed model (MLMM) model were included as covariates in the unified mixed model to control for the *ZmVTE4* effect. (C) Gene model diagram for *ZmVTE4* with δT/αT associated SNPs. Blue vertical lines indicate the physical position (RefGen_v2) of SNPs within +/- 3 kb of the open reading frame start or stop position for *ZmVTE4* that are significantly associated with δT/αT at 5% FDR. Significant SNPs at 10% FDR are shown as gray vertical lines. The peak SNP is indicated by a red triangle, while the three SNPs included in the optimal MLMM model are indicated by inverted red triangles.

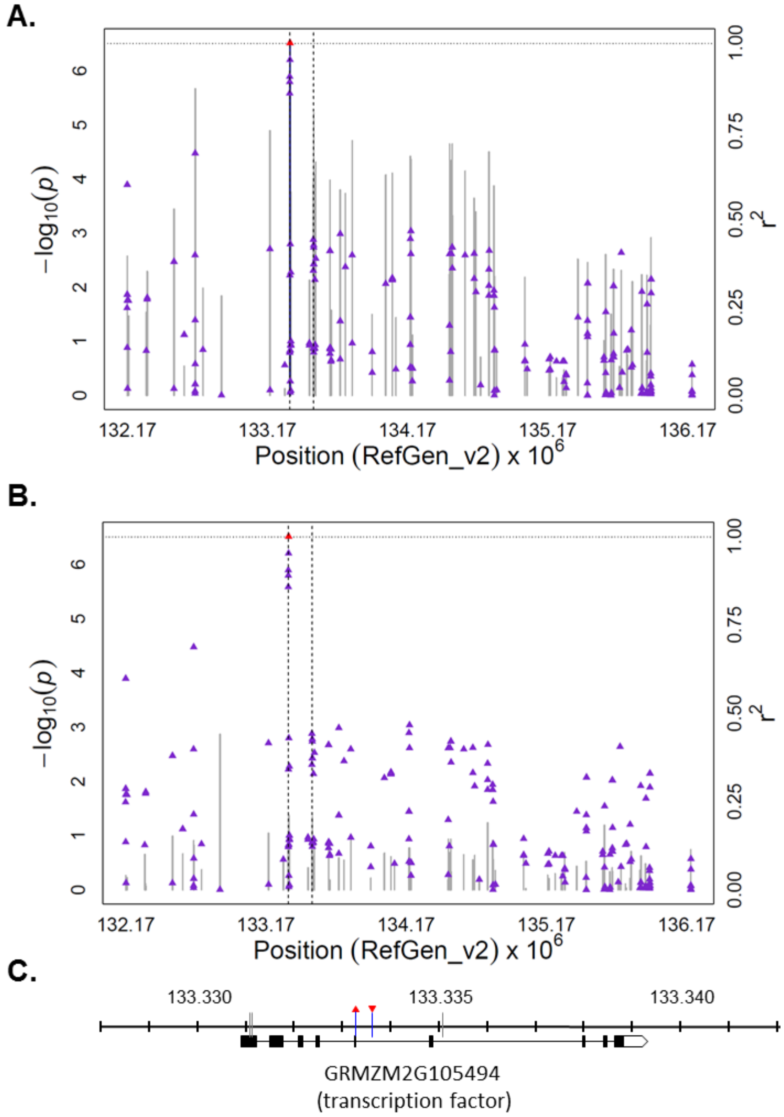

Figure S5 Genome-wide association study (GWAS) for δ-tocotrienol (δT3) in maize grain. (A) Scatter plot of association results from a unified mixed model analysis of δT3 and linkage disequilibrium (LD) estimates (r^2) across the *ZmVTE1* chromosome region. Negative $\log_{10}$-transformed *P*-values (left y-axis) from a GWAS for δT3 and r^2 values (right y-axis) are plotted against physical position (B73 RefGen_v2) for a 4 Mb region on chromosome 5 that encompasses *ZmVTE1*. The blue vertical lines are $-\log_{10}$ *P*-values for SNPs that are statistically significant for δT3 at 5% false discovery rate (FDR), while the gray vertical lines are $-\log_{10}$ *P*-values for SNPs that are non-significant at 5% FDR. Triangles are the r^2 values of each SNP relative to the peak SNP (indicated in red) at 133,333,397 bp. The black horizontal dashed line indicates the $-\log_{10}$ *P*-value of the least statistically significant SNP at 5% FDR. The black vertical dashed lines indicate the positions of two genes (from left to right): a transcription factor (GRMZM2G105494) and *ZmVTE1* (GRMZM2G009785). (B) Scatter plot of association results from a conditional unified mixed model analysis of δT3 and LD estimates (r^2) across the *ZmVTE1* chromosome region, as in (A). The SNP (S5_133333561) from the optimal multi-locus mixed model (MLMM) model was included as a covariate in the unified mixed model to control for the detected effect. (C) Gene model diagram for a transcription factor (GRMZM2G105494) with δT3 associated SNPs. Blue vertical lines indicate the physical position (RefGen_v2) of SNPs within +/- 3 kb of the open reading frame start or stop position for GRMZM2G105494 that are significantly associated with δT3 at 5% FDR. Significant SNPs at 10% FDR are shown as gray vertical lines. The peak SNP is indicated by a red triangle, while the SNP included in the optimal MLMM model is indicated by an inverted red triangle.

Table S1   Best linear unbiased predictors (BLUPs) of the 20 tocochromanol traits used for the genome-wide association study (GWAS) and pathway level analysis.

Sample ID	dT3	gT3	aT3	dT	gT	aT	Total Tocotrienols	Total Tocopherols	Total Tocopherols/Total Tocotrienols	Total Tocochromanols	dT/(gT+aT)	dT/gT	dT/aT	gT/(gT+aT)	dT3/(gT3+aT3)	dT3/gT3	dT3/aT3	gT3/(gT3+aT3)	aT/gT	aT3/gT3
4226	0.4541	18.6571	6.9331	0.8152	38.1068	9.9724	26.1683	48.6365	1.8583	73.1284	0.016	0.0201	0.0835	0.7921	0.0151	0.0225	0.0594	0.738	0.2346	0.3839
4722	0.134	3.7649	5.748	1.935	28.6182	3.7189	11.8841	35.8034	3.9689	47.7134	0.0662	0.0789	0.581	0.8623	0.0111	0.024	0.0247	0.4061	0.1415	1.586
33-16	0.5773	16.3469	9.5772	0.5634	27.2649	5.1656	29.0913	35.2571	0.9613	61.8341	0.0144	0.0196	0.0809	0.7874	0.0227	0.0341	0.0579	0.6458	0.2511	0.5614
38-11	0.2172	5.7519	7.3841	1.0276	39.8059	9.4246	13.7913	49.3912	3.8866	64.3657	0.0215	0.0244	0.1111	0.7949	0.0122	0.0403	0.0298	0.4098	0.2325	1.4282
A188	0.8148	12.8202	5.7998	1.6884	34.3081	9.2174	19.664	45.0175	2.1146	64.2888	0.0388	0.051	0.2274	0.8227	0.0508	0.0688	0.1723	0.6808	0.1877	0.4774
A214N	0.2358	5.4697	11.9109	0.9072	32.3779	9.8742	16.4853	43.6412	2.8475	58.4413	0.0205	0.0274	0.0894	0.7599	0.0075	0.0343	0.0138	0.2644	0.2902	2.7286
A239	0.5886	12.7517	5.3097	2.3019	60.0914	5.492	14.9967	66.0901	3.858	81.4059	0.0373	0.0426	0.5437	0.9132	0.0317	0.0485	0.1121	0.6996	0.0769	0.4562
A441-5	0.1121	3.8338	6.5598	0.6148	20.3641	18.3372	11.2283	41.3533	4.5445	51.6948	0.0126	0.028	0.0606	0.4862	0.0042	0.0064	0.0139	0.2546	1.0311	3.3231
A554	0.4582	6.5114	7.0616	0.9131	34.2571	2.0696	13.6153	36.957	2.8853	51.2735	0.024	0.0257	0.513	0.9442	0.0366	0.0737	0.0722	0.4601	0.0421	1.2275
A556	0.3638	8.9289	7.853	1.2471	39.2386	15.4144	19.567	59.7736	3.5147	74.8736	0.0225	0.0316	0.1055	0.7111	0.0218	0.0375	0.052	0.5509	0.4358	0.8472
A6	0.4918	13.5908	10.6258	0.6025	22.2363	6.9073	23.1235	29.0416	1.2142	51.5464	0.0168	0.0206	0.0647	0.7456	0.0204	0.0342	0.0569	0.5843	0.3149	0.7692
A619	1.5869	25.2378	5.1673	3.3237	83.048	11.0191	32.7949	95.0651	2.5806	125.5316	0.0384	0.0433	0.3601	0.8766	0.0535	0.0668	0.3103	0.8336	0.1204	0.2243
A632	0.1298	3.8057	7.0231	0.389	11.6512	14.1163	10.4281	23.5279	2.1803	36.4646	0.0108	0.022	0.0276	0.4684	0.0093	0.0216	0.0144	0.2806	1.0666	2.6556
A634	0.4042	10.0352	8.8787	0.401	15.6147	12.7774	18.9188	24.8116	1.6794	44.6067	0.0106	0.0186	0.0377	0.5773	0.0144	0.0345	0.0324	0.4739	0.6811	1.1164
A635	0.3402	7.1271	15.2541	0.2899	11.529	16.8475	21.6997	28.7546	1.2643	52.1873	0.0058	0.0154	0.0179	0.3477	0.0133	0.043	0.0202	0.2966	1.7575	2.4342
A641	0.2657	3.5426	9.9825	1.4056	35.2016	12.9695	13.2504	49.4275	3.6863	61.7111	0.0291	0.0406	0.1264	0.7491	0.0185	0.0819	0.0268	0.1985	0.3046	4.2068
A654	0.1253	1.4556	3.1904	0.6463	30.2299	9.4647	6.6717	44.2191	.	52.0527	0.0134	0.0168	0.0509	0.7221	0.0283	0.174	0.03	0.0956	0.3487	11.747
A659	0.1382	5.0723	3.1392	2.114	47.3717	3.5147	5.5522	51.6672	7.8222	58.7328	0.0419	0.0459	0.7572	0.9316	0.0139	0.0172	0.0379	0.5546	0.0605	0.7741
A661	0.4002	22.2418	6.6968	1.2624	66.8914	2.5656	28.628	69.921	2.4014	97.2321	0.0184	0.0171	0.4816	0.9506	0.0153	0.0168	0.0615	0.7578	0.0385	0.3411
A679	0.8713	14.0431	6.9975	1.7351	11.5442	2.2926	22.9346	35.0112	.	56.0546	.	.	0.3354	.	0.0254	0.0477	0.0733	.	0.041	0.8308
A680	1.2302	25.5653	11.5777	1.9069	24.3955	8.5717	40.8025	37.31	0.668	75.8972	0.051	0.0703	0.1559	0.6065	0.0304	0.0451	0.1057	0.6927	0.6281	0.4458
A682	0.2781	6.2745	6.6366	1.7331	33.2254	14.8876	12.2983	54.6563	4.4015	66.0547	0.037	0.0582	0.1221	0.6567	0.0239	0.0401	0.0531	0.4631	0.4855	1.1556
AB28A	0.2791	14.9854	6.753	0.6678	37.9459	1.1497	24.2811	42.2704	1.676	67.198	0.0144	0.0128	0.4892	0.9595	0.0099	0.0247	0.0449	0.7076	0.0265	0.4283
B10	0.1202	4.0845	7.8324	0.9798	23.3321	8.7028	14.821	31.1864	2.6701	45.6193	0.0281	0.0386	0.1085	0.6979	0.0064	0.0163	0.0132	0.2981	0.4166	2.3712
B103	0.9637	8.8489	9.2042	2.7936	49.2085	2.5588	21.4554	54.1561	2.9865	75.5796	0.0565	0.0559	1.4426	0.9428	0.0558	0.119	0.1066	0.4736	0.152	1.155
B104	0.1828	4.1327	7.4623	0.5208	15.1384	12.5802	13.248	28.8287	2.3569	40.6176	0.0154	0.0299	0.0421	0.5457	0.0116	0.0363	0.0175	0.2934	0.7763	2.451
B105	0.4352	14.2637	8.3141	0.9039	21.0734	9.4638	21.5583	33.8603	1.3365	54.3099	0.0251	0.0346	0.0706	0.632	0.0171	0.0278	0.0523	0.62	0.5583	0.6203
B109	1.563	39.0417	3.8248	2.2888	40.298	3.7659	44.6507	45.829	1.1153	90.7088	0.0534	0.0586	0.6381	0.9039	0.0352	0.0378	0.4383	0.9232	0.0863	0.1178
B115	0.3835	7.4624	7.5078	0.8001	22.3506	11.9721	17.1056	35.6844	2.1918	53.4362	0.0207	0.031	0.065	0.6411	0.0245	0.0534	0.0521	0.4935	0.5257	1.0618
B14A	0.1405	7.4241	7.13	0.4249	21.4795	11.2688	13.3457	35.7959	2.1462	49.9697	0.01	0.0183	0.0332	0.5972	0.0082	0.0117	0.0135	0.4458	0.6282	1.2456
B164	0.3232	10.5834	4.5339	1.0965	37.9151	5.48	16.3542	44.1679	3.125	61.735	0.026	0.0281	0.2305	0.8441	0.0226	0.027	0.0891	0.7128	0.1682	0.4117
B2	0.1405	4.1293	6.7759	2.8737	68.9336	4.4758	13.3725	74.8173	7.3336	88.0466	0.0422	0.0428	0.7383	0.9324	0.009	0.0259	0.0149	0.3308	0.0575	2.0072
B37	0.341	8.4968	8.4072	0.6312	22.4373	31.347	11.3013	48.3644	3.1118	63.0313	0.0101	0.0177	0.0216	0.4447	0.0178	0.0431	0.038	0.4725	1.1652	1.1685
B46	0.235	6.9693	9.9775	0.4928	10.8562	10.0047	15.7143	18.9618	1.3117	36.0621	0.0214	0.0405	0.0544	0.5628	0.0101	0.0285	0.0201	0.3834	0.7262	1.6284
B52	0.1822	10.3456	5.1513	0.7288	29.0713	10.1042	15.4803	38.9942	2.33	53.6561	0.0181	0.0231	0.0745	0.6925	0.0132	0.0134	0.0326	0.6136	0.4244	0.6283
B57	0.4154	15.555	6.0185	1.4225	55.5187	14.611	22.0839	72.2512	3.0863	92.8087	0.0205	0.0278	0.1101	0.8033	0.019	0.0252	0.0729	0.7246	0.218	0.4021
B64	0.2245	9.0566	12.2803	1.3679	43.8675	12.5019	21.7446	56.5115	2.4572	78.4463	0.0241	0.032	0.1176	0.7625	0.0094	0.0202	0.0191	0.392	0.288	1.5291
B68	0.2104	12.6293	9.6801	0.8134	37.3095	16.3622	24.4605	53.0783	2.1312	76.9778	0.0144	0.0193	0.0526	0.6833	0.0089	0.0141	0.0189	0.5527	0.4368	0.7931
B73	1.0818	27.3215	12.9806	2.7198	33.016	9.018	41.1952	44.7298	0.851	87.2936	0.0555	0.0772	0.287	0.8075	0.0274	0.0392	0.089	0.688	0.2137	0.4847
B73HTRHM	0.9586	25.9545	10.8946	1.9785	38.7874	9.8504	39.4049	50.4245	1.0682	85.9413	0.0636	0.0777	0.409	0.826	0.0264	0.0407	0.082	0.7007	0.1904	0.4418
B75	0.224	4.4529	4.3168	0.7558	23.9286	6.8931	9.9546	32.4202	3.7467	43.6748	0.0233	0.0289	0.098	0.7507	0.0207	0.0456	0.044	0.4935	0.3075	1.0611
B76	0.537	6.8796	7.1963	0.3843	15.2235	21.7006	14.7164	38.0448	2.4672	52.616	0.0075	0.0166	0.0194	0.3042	0.0431	0.0792	0.0897	0.5045	2.2055	1.0333
B77	0.3486	7.319	5.5158	1.0102	33.7946	7.0553	12.0484	41.7442	3.4233	55.6183	0.0246	0.0265	0.1442	0.8157	0.0233	0.0452	0.0577	0.5786	0.2077	0.7699
B79	0.341	6.9946	4.6331	0.5215	18.9435	12.1656	12.7965	31.6473	2.5146	46.2245	0.0111	0.0148	0.0262	0.5229	0.0292	0.0516	0.0936	0.6393	0.8402	0.5781
B84	0.1853	3.1887	9.7481	1.4581	50.5582	11.728	15.041	65.4826	5.1986	80.8259	0.0235	0.0317	0.1117	0.7288	0.0112	0.0498	0.0139	0.1803	0.3527	4.7231

B97	1.3846	19.0026	5.4041	0.5056	17.5145	9.2571	24.5542	28.1879	1.0432	51.6402	0.0148	0.0237	0.0462	0.5073	0.044	0.0638	0.1832	0.7499	0.9634	0.3364
C103	0.3178	6.7115	4.4437	0.866	23.2497	7.6757	11.3975	31.9571	2.7146	45.1976	0.0251	0.032	0.1054	0.737	0.028	0.0513	0.0779	0.6192	0.3317	0.6641
C123	0.3619	3.911	5.8764	0.5892	15.467	10.5202	10.8585	26.6277	3.3437	37.6317	0.0206	0.0367	0.0582	0.5537	0.0329	0.0959	0.0535	0.3253	0.7565	2.1151
C49A	0.5765	17.5033	11.3217	0.9947	34.2571	3.536	28.3142	40.113	1.2596	66.1746	0.0233	0.0275	0.2113	0.8759	0.0169	0.0298	0.0372	0.5634	0.1268	0.7537
CH701-30	0.4823	20.7876	7.1612	1.6528	38.5132	12.1612	28.1612	56.3594	1.6572	83.9983	0.03	0.0424	0.1018	0.6683	0.016	0.0206	0.0499	0.6984	0.4731	0.4206
CH9	0.123	3.4093	4.2708	0.8306	40.9546	2.0729	7.5798	44.9811	6.5418	51.0361	0.0182	0.0193	0.3826	0.9316	0.0119	0.0247	0.0244	0.3273	0.0593	2.0871
CI.7	0.8769	7.9866	9.2537	0.3473	16.263	14.7189	15.7289	34.6201	1.7126	53.0812	0.0076	0.0145	0.0248	0.5049	0.0591	0.1166	0.1132	0.432	0.9137	1.3153
CI187-2	1.3635	19.8266	7.1989	2.5147	28.8568	7.2441	28.6263	38.5217	1.185	67.9273	0.0729	0.0922	0.4025	0.8125	0.0682	0.0878	0.2877	0.7347	0.2078	0.3788
CI21E	0.2389	5.6234	8.6728	1.6058	35.071	13.4554	13.6213	51.2271	4.2966	66.1966	0.0311	0.0425	0.1021	0.6787	0.0129	0.036	0.0236	0.2938	0.4526	2.3093
CI28A	0.3258	6.9727	4.7706	1.2471	32.1437	5.3032	14.4493	39.4265	3.7924	54.4538	0.0326	0.0398	0.2357	0.8468	0.0275	0.0448	0.0698	0.5915	0.2482	0.7177
CI31A	0.3542	12.3978	5.6555	1.1531	34.7978	4.0635	18.0145	41.6218	1.8957	61.6682	0.0292	0.0338	0.2639	0.8752	0.0178	0.0276	0.0535	0.6829	0.1242	0.4765
CI3A	0.4876	23.3696	9.1374	0.9881	36.1436	7.9503	31.8254	43.5687	1.3539	73.6165	0.0216	0.0356	0.1451	0.8272	0.0144	0.0191	0.0496	0.6987	0.273	0.4566
CI64	0.6221	15.4016	7.3815	0.7351	7.5301	4.6038	24.7055	13.1629	0.4231	39.5203	0.0497	0.0505	0.1127	0.6155	0.0277	0.0399	0.1038	0.7183	0.5756	0.4129
CI66	0.1503	6.0926	9.6398	1.0557	18.7508	7.5118	13.5006	29.152	1.7951	43.3269	0.0369	0.0557	0.1343	0.6901	0.0072	0.0169	0.0152	0.3711	0.4191	1.8166
CI90C	0.198	5.3562	8.6378	2.4601	40.7565	12.804	14.6576	53.7427	4.1517	68.8177	0.0479	0.0627	0.2304	0.767	0.01	0.0295	0.0188	0.3161	0.2793	2.0168
CI91B	0.2011	6.8336	6.685	1.6413	79.3489	1.9116	14.8095	82.1374	6.3372	97.6227	0.0209	0.0241	0.9803	0.9666	0.0123	0.0252	0.0289	0.5013	0.022	0.9828
CM105	0.5133	10.3633	7.3765	0.664	6.1463	4.5215	19.0685	14.2416	0.4848	35.9981	0.07	0.1749	0.1411	0.4477	0.0262	0.0494	0.0644	0.5775	1.1509	0.7456
CM174	0.9212	17.403	7.1224	1.0835	8.1983	4.4936	24.7602	16.5627	0.5529	41.6242	0.0769	0.1733	0.16	0.4531	0.0283	0.044	0.0852	0.6541	1.1453	0.5142
CM37	0.808	13.0596	12.5011	1.6939	55.3318	11.0869	27.4804	68.5497	2.4798	93.7968	0.0261	0.0307	0.144	0.7771	0.0275	0.0575	0.0616	0.5354	0.2666	0.9252
CM7	1.3529	8.3519	8.7124	0.6946	7.1071	7.8763	15.978	15.7076	0.7739	33.4966	0.0369	0.0853	0.0811	0.5473	0.0866	0.2241	0.1651	0.458	0.7561	1.3685
CML103	0.3641	12.4023	8.2772	0.3256	21.7212	11.3368	22.6704	36.2587	1.236	57.1842	0.0067	0.0096	0.0246	0.5929	0.0155	0.0262	0.0416	0.5809	0.6455	0.7421
CML108	0.4114	8.4398	5.16	0.3634	8.7874	6.4102	12.357	15.2248	0.8639	28.3882	0.0095	0.0201	0.0546	0.4639	0.0277	0.0471	0.0667	0.5671	1.1367	0.792
CML11	0.8418	17.8663	.	0.4365	16.0927	10.5589	23.4981	29.3819	0.606	59.4135	0.0124	0.0206	0.0489	0.6233	0.0343	0.0476	0.0975	0.6156	0.5535	0.6393
CML154Q	0.2437	9.2869	5.6063	0.3581	19.5703	12.0756	13.3922	32.8276	1.9425	48.9743	0.0081	0.0149	0.0294	0.5974	0.0134	0.0234	0.0396	0.6175	0.6318	0.6617
CML157Q	0.2555	5.0128	3.7818	0.7296	30.0655	7.6855	6.9432	39.193	4.5382	49.014	0.0172	0.0192	0.0927	0.8007	0.024	0.0555	0.0527	0.5405	0.2214	0.906
CML218	6.0566	30.1625	9.3199	0.4271	15.9071	19.8926	44.2166	38.7718	0.8917	80.6867	0.0097	0.0224	0.0262	0.4282	0.1375	0.1905	0.7029	0.765	1.2397	0.3193
CML220	0.1712	3.8389	8.0674	0.6653	28.9135	24.9823	12.9952	52.4306	4.1812	65.4465	0.0116	0.0202	0.0311	0.5173	0.0117	0.0381	0.0216	0.2776	0.8834	2.8856
CML238	1.0008	16.6081	4.1167	0.4592	12.9166	5.2952	22.214	21.9342	0.6966	45.9695	0.0219	0.0353	0.0866	0.71	0.0524	0.0654	0.3062	0.8303	0.3689	0.2271
CML247	0.4135	7.7052	4.4029	0.7456	18.7962	5.4382	12.6865	25.1522	2.0769	39.7071	0.0305	0.0402	0.1456	0.7802	0.0339	0.0514	0.1037	0.6599	0.2572	0.5581
CML254	0.9755	21.5369	7.6359	2.6617	79.4211	11.8373	32.4395	90.0121	3.1077	116.2607	0.0333	0.0389	0.2949	0.8691	0.0349	0.0476	0.134	0.754	0.1305	0.3822
CML277	.	30.1962	6.4492	2.0763	35.6156	1.0729	42.6136	41.239	0.9284	81.8032	0.0641	0.0699	.	0.9608	.	0.1566	.	0.8303	0.0242	0.2417
CML287	0.1785	6.1294	11.1688	0.6068	17.8747	10.6663	17.8028	30.9125	1.3319	52.9457	0.0175	0.0285	0.0404	0.5053	0.0058	0.0313	0.017	0.3104	0.9206	2.1547
CML311	0.1365	2.7504	3.8513	1.7991	28.1833	12.7589	5.098	42.2106	8.7185	49.0547	0.044	0.0656	0.1796	0.7222	0.0154	0.0421	0.0278	0.3429	0.3525	2.0965
CML314	0.4015	10.7764	8.7076	0.5903	12.9607	8.341	19.1136	22.5234	0.9383	43.1317	0.0247	0.0427	0.0649	0.6024	0.023	0.0361	0.0489	0.5195	0.6159	0.9436
CML322	0.3931	8.9086	4.3801	0.8369	22.8475	2.5409	15.4164	27.4174	1.8619	43.6571	0.0293	0.0405	0.3011	0.8971	0.0301	0.0447	0.1101	0.7153	0.0957	0.4126
CML323	3.1967	17.6413	4.1073	2.0633	45.7305	0.9869	25.3002	50.6693	1.6288	75.8137	0.0405	0.0409	1.1674	0.9584	0.1469	0.173	0.8034	0.8089	0.0312	0.2529
CML332	1.5825	12.1567	6.2136	0.6833	12.1953	5.8845	20.8515	18.5256	0.9105	44.3294	0.0355	0.0541	0.1276	0.6428	0.0901	0.1353	0.2686	0.6653	0.5631	0.5283
CML333	0.2011	6.5617	2.9389	0.8174	23.2489	6.5701	7.6935	32.0034	3.4979	42.5414	0.0245	0.0335	0.1276	0.7829	0.0178	0.0247	0.064	0.6868	0.249	0.4759
CML38	0.7511	19.7594	10.1404	.	44.9995	9.6206	30.8073	58.1651	1.8099	87.7531	0.0756	0.0975	0.484	0.813	0.0262	0.0415	0.0748	0.6596	0.2059	0.5451
CML52	1.1845	30.0782	5.6693	0.8629	19.8668	4.4075	37.1686	26.6067	0.7381	63.333	0.0289	0.0359	0.1779	0.8197	0.0311	0.0427	0.248	0.8459	0.1963	0.2204
CML77	0.9918	14.946	10.5118	0.6263	21.2157	6.9543	25.8986	29.468	0.8793	55.017	0.0187	0.0251	0.0783	0.7163	0.0393	0.0689	0.119	0.6407	0.3712	0.6441
CML91	0.3607	12.642	8.5989	1.3591	42.6558	28.7872	20.3781	73.2041	3.4165	93.4427	0.0199	0.0325	0.0553	0.5616	0.0162	0.025	0.0353	0.5892	0.7427	0.7123
CML92	1.2181	16.1055	12.2142	0.6358	22.3328	10.1888	30.3156	35.4191	1.0035	64.297	0.0157	0.0249	0.046	0.6247	0.0433	0.0711	0.1034	0.579	0.5632	0.7423
CMV3	0.4504	8.8069	7.3462	0.7969	21.2973	4.2781	16.6781	27.1035	1.3262	43.2262	0.0277	0.0286	0.1532	0.8187	0.0277	0.059	0.0579	0.5581	0.2	0.9091
CO106	0.2051	3.1623	5.2938	0.5326	18.9922	5.9383	8.9167	25.4438	2.7316	35.1108	0.0172	0.0237	0.0702	0.6828	0.0201	0.0887	0.0317	0.3136	0.4424	2.6099
CO125	0.1109	4.803	7.6335	1.4452	65.1064	15.2249	13.393	81.669	8.7712	95.118	0.0192	0.0295	0.1289	0.8206	0.0039	0.0046	0.0136	0.3333	0.2785	2.1583
CO255	0.6596	10.619	4.1072	2.8111	35.4609	6.0611	15.3735	45.5774	2.6359	61.8084	0.065	0.0768	0.3995	0.8193	0.0486	0.0654	0.2113	0.7534	0.2018	0.3453
D940Y	0.1011	3.8171	3.2722	0.5581	15.1534	1.2905	7.0597	19.5535	2.7631	28.0473	0.0197	0.0209	0.2551	0.9115	0.0107	0.0237	0.0208	0.5085	0.0769	0.9669

DE_2	0.2665	8.3022	7.8143	1.9487	71.9259	3.0823	14.5853	77.2398	5.4302	88.4138	0.0264	0.0271	0.6895	0.9472	0.0137	0.0266	0.0283	0.4557	0.0412	1.1893
DE_3	0.3207	5.9126	13.1084	0.9937	23.9566	15.933	20.2047	38.0465	2.0899	58.3959	0.0245	0.037	0.0758	0.6576	0.0168	0.0529	0.0279	0.2949	0.4804	2.5358
DE1	0.4208	9.1106	6.2894	1.8737	54.3914	2.2389	17.4129	60.2283	3.543	79.0323	0.0351	0.0346	0.841	0.9475	0.0262	0.0452	0.0677	0.5816	0.0415	0.7338
DE811	0.3511	11.1679	11.7084	2.9428	45.7691	18.0473	24.7956	64.5213	2.5184	88.8366	0.0495	0.0712	0.2027	0.7265	0.0144	0.0288	0.0317	0.465	0.345	1.1746
E2558W	5.309	41.4875	19.1085	2.3895	27.4188	15.6367	67.7217	42.9157	0.7258	106.9698	0.0625	0.1004	0.2119	0.6661	0.1116	0.1704	0.3234	0.6315	0.4655	0.5755
EP1	0.3829	4.6963	3.6466	0.7671	10.5379	5.8372	5.9121	20.5088	2.5127	28.8197	0.0435	0.0856	0.1223	0.5282	0.0522	0.107	0.1035	0.4907	0.8819	1.0779
F2834T	0.506	25.0619	4.7805	0.538	27.0221	11.0737	29.0696	39.7507	1.3758	69.7206	0.0116	0.0179	0.0435	0.6735	0.0148	0.0186	0.0932	0.8262	0.4567	0.2224
F44	0.099	2.1445	4.8304	0.8062	9.735	16.3316	7.242	29.4305	5.1602	38.1195	0.0244	0.0704	0.0457	0.3178	0.0087	0.0246	0.0169	0.1972	2.0298	4.4397
F6	0.8855	10.029	4.1883	1.3913	23.8443	1.5116	13.3665	27.564	2.2329	41.0523	0.0549	0.0604	.	0.9478	0.0686	0.0884	0.2657	0.73	0.0359	0.4068
F7	0.2645	10.2264	3.103	1.8496	50.447	10.8247	12.8557	58.696	4.5718	73.6739	0.0321	0.0385	0.23	0.842	0.0188	0.0242	0.0868	0.7736	0.1688	0.3143
GA209	0.678	11.4542	10.0711	1.8489	31.1966	4.9666	24.8176	39.1703	1.0762	63.1047	0.0527	0.0584	0.3785	0.8505	0.032	0.0583	0.0718	0.5368	0.2451	0.9006
GT112	0.6052	10.8975	8.8387	1.1258	21.2869	8.5093	20.5966	31.1007	1.2652	49.4528	0.0376	0.0494	0.144	0.7238	0.0348	0.0561	0.0811	0.5665	0.3449	0.8266
H105W	0.2324	5.1863	13.5137	0.7521	29.0716	17.7555	17.5431	49.2348	2.4183	68.2539	0.0149	0.0252	0.0468	0.6147	0.0123	0.0397	0.0161	0.2302	0.581	3.4752
H49	1.7174	27.7465	8.7341	1.8925	61.9578	1.535	36.5946	63.3921	1.7335	99.9836	0.0303	0.0308	1.269	0.9633	0.0459	0.062	0.1862	0.7431	0.0241	0.3503
H84	0.1941	5.5064	5.6359	0.4091	16.5522	9.7113	9.4532	30.1359	2.6823	42.2161	0.0097	0.0155	0.0284	0.5334	0.0151	0.0277	0.0323	0.4566	0.8313	1.1972
H91	0.2415	9.5689	10.9547	0.8645	31.1448	9.2106	20.8645	44.5958	1.9523	65.2212	0.0189	0.0252	0.0712	0.7341	0.0105	0.0199	0.0176	0.469	0.3334	1.1536
H95	0.3652	13.0791	6.6808	0.8276	28.4385	8.6008	18.1614	39.5517	1.7367	59.2323	0.0215	0.031	0.0868	0.6604	0.0176	0.0252	0.0494	0.6653	0.4934	0.532
H99	0.255	3.6482	4.9821	1.5022	52.6462	11.8419	7.768	65.0071	7.7402	71.7265	0.0234	0.0279	0.1361	0.7974	0.0189	0.069	0.0284	0.3022	0.2338	2.3331
HI27	0.2888	6.7584	6.937	0.3346	9.5896	7.9743	12.3244	14.4619	1.4184	27.5959	0.0095	0.0156	0.0401	0.6266	0.0198	0.0396	0.0451	0.4977	0.5442	1.113
HP301	0.1707	5.5789	3.2778	0.9117	28.7085	1.1533	8.9327	33.7865	3.811	43.5588	0.0283	0.032	0.8941	0.9519	0.015	0.0324	0.0516	0.6395	0.0342	0.5671
HY	0.259	2.0594	6.8817	1.2416	29.4859	15.2323	10.2586	46.5715	5.5698	58.282	0.0254	0.0391	0.0676	0.5638	0.0223	0.065	0.0236	0.1275	0.7421	7.9665
I137TN	0.366	9.5555	4.2251	0.7827	31.2872	1.5489	13.5898	32.4901	2.1805	46.853	0.0206	0.0216	0.4707	0.9424	0.0263	0.0368	0.0862	0.6774	0.0466	0.4787
I205	0.2713	5.9879	6.0123	0.991	35.2307	1.8073	12.6682	38.9851	3.6647	53.4771	0.0234	0.0257	0.5889	0.9445	0.0205	0.0484	0.0469	0.4833	0.0409	1.0728
I-29	0.2956	5.9294	2.9702	1.1249	25.5307	2.6668	10.4199	30.9622	3.632	44.5219	0.04	0.046	0.3855	0.881	0.0353	0.0458	0.1373	0.7155	0.117	0.4165
IDS28	0.1554	2.3481	5.5719	1.6434	41.299	5.478	10.0499	47.9068	7.7661	56.7707	0.034	0.0373	0.3288	0.8753	0.0134	0.081	0.0196	0.2073	0.1272	4.0939
IDS69	0.1583	3.8097	2.8614	1.6844	24.8501	1.4231	7.093	30.4564	5.183	38.6357	0.0686	0.0756	1.3884	0.934	0.0168	0.0318	0.0416	0.5465	0.0552	0.8211
IDS91	0.112	4.0582	3.6226	1.6852	22.6151	1.6334	8.2443	25.0172	5.0624	36.5891	0.0721	0.0785	2.0407	0.941	0.0114	0.0137	0.0342	0.5716	0.0492	0.7464
K148	0.3139	9.1904	7.0351	1.045	23.0205	8.5773	19.3189	32.7236	1.5127	52.9048	0.0277	0.0406	0.095	0.6105	0.0172	0.0335	0.0327	0.5265	0.6272	0.8936
K4	0.2329	8.6032	6.893	2.7813	43.8975	8.0272	16.2834	57.187	3.44	74.4118	0.0557	0.0686	0.3412	0.8122	0.0145	0.0225	0.0428	0.5765	0.2102	0.7757
K55	0.3638	10.2716	7.5351	1.5442	51.3818	4.2933	20.7369	60.2729	3.1633	78.4911	0.0276	0.0361	0.3004	0.9008	0.0198	0.0317	0.0515	0.5892	0.1955	0.7262
K64	1.0274	43.5797	7.4064	0.8168	17.536	6.8157	49.3226	25.8746	0.4759	75.2812	0.0291	0.04	0.1068	0.6995	0.0178	0.0242	0.1158	0.8559	0.3962	0.2006
KI14	0.4876	11.4098	8.2101	0.3938	15.358	9.1463	22.7268	26.3086	0.7284	48.6893	0.0084	0.0107	0.0546	0.5827	0.0248	0.0401	0.0638	0.5937	0.7125	0.7111
KI21	1.128	14.1977	5.5092	1.6523	26.6319	9.446	20.8578	37.14	1.6468	60.8027	0.0517	0.0702	0.2419	0.7752	0.0702	0.0847	0.3243	0.7418	0.264	0.384
KI3	0.8002	13.9891	4.964	1.1633	40.2998	12.8895	20.2178	57.525	3.0287	80.0516	0.0395	0.06	0.2616	0.8093	0.055	0.0585	0.1242	0.7833	0.1996	0.3234
Ki43	0.2181	1.7898	3.5123	1.3297	28.3492	14.6839	6.6137	45.7324	.	54.2226	0.0304	0.0488	0.0865	0.5876	0.035	.	0.0403	0.2133	0.6647	.
KI44	2.1919	34.8847	9.3501	1.1252	15.7385	5.7244	49.6356	22.767	0.3735	69.434	0.056	0.081	0.2196	0.7163	0.0477	0.0594	0.2366	0.7886	0.4257	0.2958
KY21	0.3954	18.7945	9.679	1.0383	36.3546	6.1403	29.0015	44.2934	1.4327	75.0508	0.0233	0.0267	0.1502	0.8473	0.0117	0.0199	0.0416	0.6705	0.168	0.4961
KY226	0.1131	2.8259	8.3054	0.4511	18.6225	4.4671	9.5943	27.1798	2.0388	35.8248	0.0117	0.0109	0.0446	0.7196	0.0074	0.0234	0.0108	0.1694	0.3628	5.1565
KY228	1.2008	42.0513	9.4743	1.2521	42.3771	2.1251	50.7436	46.416	0.9515	95.4176	0.0272	0.0265	0.6078	0.9439	0.0232	0.0282	0.1213	0.816	0.0445	0.2443
L317	0.5112	4.4227	10.7105	0.9422	29.3623	15.5455	16.6992	43.924	2.8054	59.0074	0.0201	0.0284	0.0625	0.6508	0.0349	0.1353	0.0462	0.2279	0.5009	3.5631
L578	1.469	11.8489	13.9691	1.481	36.4001	7.5506	28.1773	47.2925	1.3867	73.719	0.0347	0.0456	0.2044	0.8147	0.0494	0.1313	0.1534	0.4859	0.2031	1.175
M14	0.506	10.2151	5.4831	0.7922	13.1382	0.701	16.5926	15.9302	0.692	32.1283	0.0517	0.0525	0.976	0.9312	0.029	0.0456	0.0844	0.6392	0.058	0.5677
M37W	0.1896	7.7684	5.5179	0.5877	21.6916	2.8194	12.0082	26.2334	1.6832	39.9696	0.0201	0.026	0.1508	0.8769	0.0084	0.021	0.0208	0.5524	0.1207	0.8275
MEF156-55-2	0.1166	4.8081	7.2454	0.5763	23.3488	5.7042	13.4059	31.6433	2.2122	45.9363	0.0139	0.0203	0.0598	0.7748	0.0104	0.024	0.0134	0.3453	0.265	2.0566
MO17	0.3014	7.9454	6.4246	0.9248	33.6239	12.4102	14.7749	46.9608	3.4238	65.5855	0.0214	0.0275	0.0933	0.6472	0.0192	0.0328	0.046	0.5537	0.5098	0.8294
MO18W	0.9256	14.9933	13.6731	2.527	56.2103	8.2227	32.2127	68.5216	1.9087	97.3023	0.0396	0.0521	0.2774	0.8407	0.0329	0.06	0.0735	0.5361	0.2517	0.9059
MO1W	1.1959	26.2833	8.5473	0.8076	39.2439	1.9602	40.4241	41.7899	0.947	81.8214	0.019	0.0201	0.434	0.9414	0.0356	0.0441	0.1147	0.6753	0.0439	0.5089
MO24W	0.8421	11.0649	15.5362	0.7549	20.5367	15.1767	25.254	39.0569	1.4977	64.0849	0.0182	0.0324	0.0522	0.595	0.0329	0.0743	0.0574	0.4206	0.6255	1.4475

MO44	0.2401	9.4358	7.2764	1.0643	26.3935	17.5998	15.3465	44.2814	2.8345	61.8213	0.0223	0.0403	0.0844	0.573	0.0117	0.0197	0.0293	0.5182	0.7464	0.9657
MO45	0.2403	4.5263	8.0155	0.656	22.2604	18.051	13.3033	42.8837	3.2454	55.1363	0.0144	0.0288	0.0396	0.5379	0.017	0.0499	0.024	0.3263	0.8012	2.1844
MO46	0.7806	9.7667	6.9765	1.163	33.5242	5.8459	18.2676	43.774	2.2286	61.6935	0.0286	0.0365	0.1827	0.8287	0.0473	0.0789	0.1183	0.5817	0.1834	0.7232
MO47	0.3091	15.8466	7.0602	1.7824	47.6434	1.8782	25.0102	50.4351	2.2534	74.8581	0.0367	0.0335	1.1892	0.9551	0.0133	0.0177	0.0415	0.7107	0.0304	0.435
MOG	0.3779	10.3699	10.7603	0.3527	17.0446	20.4391	23.1664	35.5209	1.4189	59.4823	0.0071	0.0155	0.0221	0.4732	0.0203	0.0413	0.0468	0.5199	1.0363	1.1025
MP339	3.1066	33.6948	6.4569	1.3182	31.422	5.8811	45.1901	38.9363	0.7492	83.068	0.0359	0.0477	0.2673	0.8497	0.0773	0.0928	0.5553	0.8525	0.1516	0.1893
MS1334	1.9729	25.9482	6.5941	1.0033	27.1207	2.6967	37.3939	31.607	0.5359	66.4541	0.0313	0.0344	0.2996	0.8869	0.0607	0.0725	0.3797	0.8249	0.2085	0.243
MS153	0.3332	5.5238	6.4681	0.8446	28.4762	2.2546	11.5697	33.1723	2.8745	46.8897	0.0228	0.0251	0.349	0.9213	0.0273	0.0649	0.054	0.4221	0.0671	1.3176
MS71	0.2479	9.2633	6.2609	0.6875	25.9256	8.5217	14.9623	37.9308	2.3937	52.5483	0.0176	0.0222	0.0655	0.7167	0.0164	0.0232	0.0416	0.5429	0.3638	0.8668
MT42	0.4257	18.8154	10.8172	1.3231	32.875	13.6242	28.8354	45.376	1.2189	73.9923	0.0288	0.0391	0.1183	0.7456	0.0159	0.024	0.0504	0.6271	0.3094	0.6585
N192	1.4683	16.4782	9.5819	1.4452	27.7211	11.4481	25.4792	41.2042	1.5449	67.0623	0.0369	0.0543	0.1479	0.6975	0.0545	0.0877	0.1422	0.6164	0.4583	0.649
N28HT	0.2558	5.1771	8.7388	0.7344	23.4731	14.2979	13.5233	37.1491	2.4593	53.0064	0.0175	0.0265	0.0485	0.5636	0.0141	0.0455	0.0253	0.3148	0.7346	2.2806
N6	.	55.2461	5.7461	1.7593	21.2766	2.3713	74.5892	26.3756	0.4725	97.598	0.054	0.0894	0.8153	0.8965	0.2196	0.249	.	0.8895	0.0956	0.1398
N7A	0.2499	10.0203	6.2336	1.1011	34.8555	1.8808	17.9004	38.6506	2.4366	55.139	0.0271	0.0285	0.4867	0.9359	0.0124	0.0204	0.0374	0.654	0.0518	0.5479
NC222	0.1544	3.7449	9.5889	0.4472	11.5527	12.3332	14.2031	24.6023	1.922	44.3318	0.0114	0.0256	0.0569	0.4209	0.0078	0.0334	0.0179	0.1842	1.3952	5.5241
NC230	0.456	12.8871	4.8908	1.7533	36.3215	4.0728	16.8545	40.6529	2.358	61.6736	0.0459	0.0483	0.4541	0.8804	0.0245	0.0329	0.1042	0.7409	0.1187	0.3638
NC232	0.9217	15.4238	4.684	1.1634	24.7322	1.3502	21.2783	26.4105	1.169	48.8737	0.0411	0.0467	1.0658	0.9472	0.0548	0.0674	0.2992	0.8048	0.0402	0.2662
NC236	0.2465	4.1632	7.7913	1.2493	.	5.5756	12.4849	26.337	.	59.54	0.0274	0.03	0.2997	0.9081	0.029	0.0614	0.0456	0.3116	0.0764	2.3011
NC238	0.3907	8.3613	5.5969	0.6688	16.2385	3.0209	14.2769	21.7813	1.4068	37.8873	0.0344	0.0455	0.2178	0.822	0.0256	0.0414	0.059	0.5961	0.1924	0.6842
NC250	0.6636	14.3097	5.2386	1.2417	42.2167	8.9733	19.2245	50.235	2.2894	69.1319	0.0238	0.0252	0.1401	0.8107	0.0316	0.0508	0.1002	0.7173	0.213	0.4226
NC258	0.3921	10.1528	9.1103	1.0275	32.5009	6.6236	20.0732	37.3252	2.3204	57.2272	0.0248	0.029	.	0.793	0.0176	0.033	0.0389	0.5024	0.2599	0.96
NC260	0.1718	5.8329	5.58	1.0134	27.1221	12.3488	10.2007	42.054	3.4254	54.7465	0.0244	0.0369	0.0736	0.6299	0.0115	0.0219	0.0195	0.4781	0.5554	1.1046
NC262	0.4472	9.8124	7.1402	1.1635	32.3918	3.9548	15.2861	41.8051	2.2495	59.0001	0.0286	0.034	0.2654	0.8976	0.0318	0.0448	0.1011	0.6053	0.0887	0.7455
NC264	0.1758	2.0712	5.5275	1.5073	37.7472	8.2806	6.1185	46.3253	7.3683	53.1665	0.0343	0.0443	0.1871	0.8058	0.0115	0.1138	0.0162	0.1164	0.2176	7.8401
NC290A	1.1074	17.0281	5.9082	1.3249	32.745	1.7531	24.9912	36.8352	1.1955	60.6167	0.0393	0.0416	0.7373	0.9303	0.0519	0.0657	0.2531	0.7766	0.059	0.3248
NC294	0.1892	13.6351	7.7229	0.6421	19.8801	8.9866	21.1772	28.3047	1.0537	49.6495	0.0197	0.0285	0.071	0.6969	0.0074	0.012	0.0265	0.6516	0.4047	0.6167
NC296	0.7202	11.5012	.	0.5337	10.1034	22.2939	30.7823	29.9936	0.9826	60.6651	0.0102	0.0221	0.0198	0.333	0.0198	0.0633	0.0343	0.3273	1.8936	2.1467
NC296A	0.7	13.0755	15.5369	0.2622	11.0911	16.6708	27.7605	32.706	0.9679	61.0226	0.0048	0.012	0.0169	0.3383	0.026	0.0512	0.0445	0.4332	1.834	1.3202
NC298	0.9799	24.8476	8.9224	0.7771	25.9309	3.3945	33.8936	29.5777	0.7533	63.0683	0.0256	0.0306	0.2458	0.8801	0.0287	0.0383	0.0984	0.6721	0.1189	0.4812
NC300	0.4185	8.5464	9.948	0.2594	5.2691	11.8138	19.6244	15.9653	0.704	37.9487	0.0062	0.0163	0.0186	0.296	0.0239	0.0542	0.0505	0.4745	2.2133	1.2011
NC302	0.7483	17.7767	12.6369	0.4557	10.1889	10.0502	31.4103	19.8879	0.4398	51.0247	0.016	0.036	0.0379	0.4682	0.031	0.0407	0.0741	0.5421	1.0825	0.8357
NC306	1.3498	45.2225	9.1021	0.9954	37.9497	16.2889	53.7748	49.0972	1.0695	100.2935	0.0204	0.0274	0.0857	0.75	0.0207	0.0304	0.1159	0.8174	0.3062	0.2337
NC310	0.2439	15.7147	9.541	0.8081	27.3102	7.7666	27.0928	37.5051	1.1957	65.5179	0.0199	0.0273	0.0883	0.7545	0.0084	0.0145	0.0271	0.6347	0.3013	0.6017
NC314	0.2115	20.1862	11.8851	0.9652	61.1742	11.3376	35.083	73.477	2.041	104.6474	0.0137	0.0163	0.1012	0.832	0.0067	0.0083	0.0222	0.6487	0.2665	0.5674
NC318	0.1692	1.9271	3.9592	1.4217	37.6755	6.643	6.6347	47.9858	.	55.6224	0.0325	0.0436	0.2026	0.8213	0.028	0.1553	0.0355	0.2041	0.194	4.6817
NC320	0.1718	1.7859	4.5233	1.3537	23.7455	16.291	6.8563	43.9546	9.3411	53.6625	0.0344	0.0647	0.0951	0.5752	0.0197	0.1864	0.0259	0.1221	0.686	7.9042
NC324	0.2249	22.0245	6.5094	0.869	51.7111	7.113	28.2182	59.1922	1.9783	86.2585	0.0147	0.0198	0.1119	0.8746	0.0115	0.0185	0.0313	0.7681	0.1259	0.3392
NC326	0.7851	25.3633	10.1942	2.4838	40.4419	7.541	36.5874	51.129	1.1961	85.5449	0.0526	0.0618	0.3527	0.828	0.0214	0.0301	0.0753	0.7025	0.1888	0.4341
NC328	0.8252	21.8947	9.5829	2.4478	33.2949	11.2683	30.1998	44.8922	1.4554	75.6751	0.0568	0.0696	0.2424	0.7514	0.0237	0.036	0.0752	0.6802	0.3074	0.4797
NC33	0.5519	10.6514	5.5263	1.489	22.7356	1.7463	18.4614	26.9639	1.476	45.9953	0.0598	0.0664	0.9325	0.9231	0.0341	0.0513	0.1107	0.6705	0.0679	0.4787
NC336	1.0057	18.0401	16.1857	0.3702	15.335	8.7663	34.0717	26.5306	0.6125	59.9884	0.0097	0.0175	0.0288	0.543	0.0273	0.0531	0.0561	0.5174	0.7928	0.9415
NC338	1.2674	19.1625	16.8891	1.0298	23.542	10.9033	36.1923	36.6132	0.8784	71.0408	0.0247	0.039	0.0685	0.5199	0.032	0.0619	0.0696	0.5145	0.9066	0.9593
NC340	0.3085	7.048	10.2766	0.243	5.0438	16.1494	18.39	21.4942	0.9907	40.2366	0.0044	0.014	0.0152	0.1959	0.0169	0.0479	0.0317	0.3826	.	1.6589
NC342	0.8949	20.609	5.7298	0.9791	21.49	1.8143	26.2044	26.8033	0.9193	53.3996	0.0381	0.0414	0.3544	0.8633	0.034	0.0398	0.1698	0.7776	0.1431	0.2895
NC344	0.1771	5.5626	6.0946	0.5812	15.2531	9.1458	12.3814	23.6245	1.9939	40.5962	0.0213	0.0298	0.0709	0.6499	0.0135	0.0299	0.0344	0.4695	0.5195	1.5022
NC346	0.3565	10.7087	22.384	0.3097	12.8933	19.9432	33.7826	40.5051	0.9417	74.9715	0.0051	0.0108	0.0159	0.3065	0.0102	0.0305	0.0196	0.311	2.1013	2.2588
NC348	1.5637	26.6501	9.1726	0.612	26.071	2.778	37.6638	30.5882	0.5255	67.6655	0.0186	0.0223	0.1828	0.8823	0.0462	0.061	0.1688	0.7378	0.1155	0.3739
NC350	0.1309	3.4857	5.6387	0.4167	15.4877	16.1188	8.2196	34.4396	3.8975	44.334	0.0098	0.0227	0.0259	0.4209	0.0095	0.0246	0.0161	0.3223	1.3026	2.18

NC352	1.2576	16.3523	15.6841	0.4769	18.0198	10.4232	35.2426	30.9992	0.7163	66.2424	0.013	0.0236	0.0422	0.6394	0.0458	0.0822	0.0938	0.5165	0.5115	1.007
NC354	0.3009	6.2996	11.3148	1.3464	41.5631	15.4486	15.8812	55.4323	3.3678	72.9346	0.0241	0.0322	0.1105	0.7637	0.019	0.0461	0.0349	0.3435	0.2793	2.1135
NC356	0.3976	18.1999	10.5173	0.2166	21.219	14.1596	30.1727	36.4821	0.9431	67.3545	0.0027	0.0012	0.0128	0.5413	0.0131	0.0214	0.0432	0.6462	0.8025	0.5777
NC358	0.8308	9.3537	11.0401	0.481	16.8081	10.2292	23.8009	31.7232	0.9988	55.4652	0.0127	0.025	0.0328	0.504	0.0418	0.0921	0.0753	0.4639	0.9354	1.1699
NC360	0.9541	7.0794	5.1248	0.8662	32.1353	6.2704	14.0349	43.5672	3.5718	55.9151	0.0208	0.025	0.1469	0.8374	0.0975	0.1608	0.2466	0.6098	0.2617	0.6657
NC362	3.3055	26.3773	9.3477	0.9608	31.4923	2.0563	39.5907	35.0622	0.8945	74.3645	0.0283	0.0303	0.5431	0.9364	0.0868	0.1186	0.3238	0.7179	0.0532	0.3985
NC364	2.0695	15.3123	7.1643	0.8147	26.5847	1.2408	23.5582	27.8122	1.4153	51.5775	0.0272	0.0284	0.4763	0.9457	0.0936	0.1272	0.2727	0.6561	0.0411	0.5382
NC366	1.0121	6.7362	5.4958	1.4804	32.3766	1.8639	14.8297	35.0847	2.7453	53.7458	0.0459	0.0484	1.0812	0.9357	0.1034	0.1662	0.2625	0.5901	0.0539	0.7094
NC368	0.3924	22.5871	9.4749	1.0033	41.8262	10.351	31.2752	50.3762	1.5666	82.8117	0.0183	0.0189	0.1077	0.7824	0.0118	0.0155	0.0394	0.6843	0.3302	0.4877
ND246	0.1749	4.5063	4.993	1.2452	40.0682	1.8911	9.4808	41.7204	5.1898	54.3701	0.0297	0.0302	0.6659	0.943	0.014	0.0315	0.0323	0.4669	0.0433	1.1785
OH40B	0.2792	8.6642	5.8399	0.8944	26.3996	10.3274	13.1326	37.6027	2.619	51.4212	0.0221	0.0349	0.0948	0.7425	0.0166	0.0288	0.0401	0.551	0.3182	0.7982
OH43	0.4707	19.6212	7.2497	1.0832	64.9073	19.3493	23.5691	81.0054	2.9428	106.4031	0.0135	0.0173	0.0724	0.7914	0.0151	0.0239	0.0523	0.7029	0.2397	0.4277
OH43E	0.253	9.0263	5.3555	1.8542	85.9447	3.4896	15.4763	89.4645	7.1862	106.5013	0.022	0.021	0.7325	0.9539	0.0151	0.0235	0.0433	0.6221	0.0344	0.6126
OH603	0.3099	8.0695	8.9396	0.3886	15.8154	13.0365	19.236	27.9029	1.3612	47.7095	0.0102	0.0191	0.0306	0.4467	0.0199	0.0383	0.0396	0.4783	1.2085	1.187
OH7B	0.623	19.8456	3.7996	2.5755	42.626	2.1411	22.801	46.6821	2.0672	71.0535	0.0619	0.0718	1.5391	0.9385	0.024	0.0295	0.17	0.844	0.0512	0.2068
OS420	0.2104	4.8516	7.4448	0.9294	45.5779	7.0295	11.0851	52.7001	5.531	66.6585	0.0177	0.0162	0.1319	0.8409	0.0152	0.0364	0.0295	0.362	0.1736	1.7909
PA762	0.3035	14.0818	8.8567	0.7807	49.2339	16.0902	24.6588	63.7125	2.5608	89.9518	0.0116	0.0194	0.0507	0.744	0.0112	0.0212	0.0311	0.6308	0.3188	0.6093
PA875	0.493	4.7792	10.3353	0.9332	20.916	13.7144	16.9399	31.5692	2.0456	48.9594	0.0278	0.0463	0.0819	0.5882	0.0333	0.1141	0.0468	0.2571	0.6776	2.7909
PA880	0.6764	13.1074	6.2587	1.5742	37.4739	1.9103	19.4405	41.2107	2.1258	61.3575	0.0395	0.039	0.869	0.9286	0.0282	0.0455	0.0841	0.6281	0.0621	0.594
PA91	0.288	6.4581	6.0793	0.9424	15.4111	6.8401	11.8444	22.3775	2.1828	36.2386	0.0433	0.0565	0.1462	0.7373	0.0164	0.0397	0.0341	0.4864	0.323	1.0561
R109B	0.2972	9.827	5.9931	1.1861	50.9759	3.1856	18.5943	54.8568	3.7687	73.28	0.0219	0.0245	0.4528	0.9398	0.0172	0.0255	0.0488	0.6263	0.1563	0.6203
R168	0.1925	5.5855	7.6537	0.7519	17.8526	9.7494	15.8675	28.2777	1.9248	45.7183	0.024	0.0385	0.0946	0.6242	0.0118	0.0265	0.0256	0.3838	0.6051	1.7017
R177	0.5209	8.3483	6.2253	0.6931	27.2095	0.9954	13.7947	28.9649	2.3395	44.1079	0.0225	0.024	0.9414	0.962	0.0332	0.0586	0.0715	0.5424	0.0236	0.8506
R229	1.1062	24.6063	11.6693	2.3264	35.4942	9.3703	37.0943	46.0304	1.2828	80.2811	0.0538	0.0649	0.2745	0.7613	0.0239	0.0398	0.0646	0.6303	0.2934	0.5882
R4	0.2804	4.5739	5.4438	0.359	7.2772	4.9325	7.9221	14.4613	1.2377	25.5125	0.0177	0.0386	0.0419	0.4582	0.0259	0.0597	0.0507	0.4139	1.1103	1.3833
SA24	0.3286	7.0132	6.2301	2.2999	44.3934	4.439	14.9319	52.7626	4.4969	69.7074	0.049	0.0533	0.5002	0.8696	0.0169	0.0375	0.0395	0.5326	0.1341	0.9076
SC213R	1.4756	17.1981	5.1353	0.7362	17.3189	4.816	23.1003	21.21	0.8887	48.1746	0.0313	0.0401	0.1964	0.8383	0.0727	0.1078	0.3075	0.7732	0.1712	0.3253
SC357	0.0934	2.5273	4.5242	0.6678	31.6926	8.713	7.7465	41.8646	7.7807	51.4226	0.014	0.0183	0.0578	0.7623	0.0088	0.0334	0.0141	0.2433	0.2856	3.3148
SC55	1.2801	15.1616	5.9268	0.8144	22.2021	1.516	21.0912	26.0737	1.0392	46.6345	0.0323	0.035	0.4657	0.9179	0.0585	0.0811	0.1949	0.6995	0.0741	0.4484
SD40	0.2674	10.2215	5.1464	1.6455	55.1897	1.4101	16.3109	60.8813	3.8399	79.0922	0.0287	0.0302	1.0916	0.9584	0.0132	0.0211	0.0374	0.656	0.0272	0.5184
SD44	0.3829	6.1103	5.2997	1.4574	36.3641	2.0885	12.2319	39.5474	3.3642	51.7073	0.0367	0.0406	0.7027	0.9367	0.0376	0.0644	0.0845	0.5227	0.0563	0.9439
SG1533	0.3773	6.5716	4.7738	0.8299	20.735	1.2848	13.6338	24.1497	1.838	38.2825	0.0366	0.0374	0.7071	0.9376	0.04	0.0582	0.1269	0.6376	0.0515	0.6147
SG18	0.1625	4.5124	4.1876	0.8439	25.0117	1.082	9.5117	27.968	3.05	39.9371	0.0318	0.0354	0.7625	0.9477	0.0161	0.027	0.0299	0.4734	0.0418	1.0706
T232	0.5133	14.7309	6.3838	1.6902	46.6986	2.4834	20.4212	49.0497	2.3663	71.5878	0.034	0.0383	0.6318	0.9374	0.0233	0.0327	0.0733	0.6887	0.0503	0.4813
T234	1.0738	13.5202	5.9292	0.9174	29.2272	6.7683	19.486	36.3679	1.6097	56.1808	0.0252	0.0307	0.1441	0.8003	0.0582	0.0823	0.1923	0.6655	0.2338	0.508
T8	0.2067	16.2355	5.329	0.6804	28.6136	2.3347	19.2249	30.3165	1.3319	50.8912	0.0197	0.0207	0.2995	0.9252	0.0077	0.0107	0.0309	0.7089	0.0634	0.4094
TX303	2.2177	20.1806	3.5128	2.5966	57.274	4.195	25.1812	63.0351	2.5304	87.3946	0.0444	0.0466	0.6632	0.9173	0.0953	0.1059	0.6324	0.8383	0.0739	0.2164
TX601	0.1492	1.8197	4.7485	0.4036	13.5576	3.4795	7.4438	18.8393	3.6974	25.0356	0.0135	0.0184	0.1205	0.8643	0.0225	0.1475	0.0312	0.1323	0.1374	7.2096
TZI11	0.1015	3.504	8.4445	0.2298	6.1082	8.4281	14.7227	21.0862	1.062	37.1537	0.0034	0.0085	0.013	0.1829	0.0058	0.0086	0.0152	0.2886	.	3.2232
TZI16	0.4179	6.8812	9.1776	0.6647	24.6981	7.4678	17.1693	35.7932	1.6762	53.3158	0.0174	0.0244	0.074	0.7524	0.0283	0.0667	0.0554	0.4237	0.2958	1.5407
TZI18	0.1939	9.6529	7.7221	1.558	44.1119	1.601	16.8434	49.081	2.6535	65.7809	0.0362	0.0407	1.3241	0.9567	0.0072	0.0246	0.0232	0.5291	0.031	0.8927
TZI25	0.5638	6.3858	3.7216	0.9652	22.9687	2.9346	13.0279	27.1903	2.8269	41.673	0.0362	0.0388	0.3879	0.897	0.0668	0.1028	0.1885	0.6462	0.1972	0.5738
Tzi9	0.1398	2.6138	3.8224	0.5093	14.1883	13.3753	3.7661	27.7766	5.4232	35.2969	0.0107	0.017	0.0234	0.4666	0.0221	0.0692	0.031	0.2848	1.0883	2.7052
U267Y	0.2636	7.7417	6.3814	0.5702	18.7212	10.1384	17.6728	31.2698	1.7309	50.1748	0.015	0.0221	0.0366	0.5397	0.016	0.0371	0.0452	0.5621	0.8025	0.7875
VA102	0.3737	10.8033	7.7459	0.4418	13.6314	2.0483	20.8884	16.613	0.7019	38.6546	0.0212	0.0237	0.1501	0.8837	0.0198	0.0348	0.0462	0.5921	0.1074	0.7285
VA14	0.0968	6.6787	11.9361	1.6517	43.1489	1.0636	17.4007	46.8799	2.5955	64.2912	0.0374	0.0384	1.9239	0.9635	0.0041	0.0058	0.008	0.3221	0.0245	2.1117
VA17	0.1939	13.436	9.608	1.3324	43.0762	0.9909	22.1726	46.7022	1.9534	69.6559	0.0306	0.0325	.	0.9668	0.0093	0.0208	0.02	0.5873	0.0179	0.7666
VA22	0.1534	6.9851	5.434	1.9516	42.5709	1.6935	13.9153	45.422	3.9781	57.1355	0.0452	0.047	1.4933	0.9501	0.0132	0.0148	0.0326	0.5673	0.0385	0.8079

Va26	0.8667	39.4257	8.0586	1.1221	32.5028	10.8311	48.4856	43.5971	1.1074	89.1993	0.0253	0.0343	0.1354	0.7847	0.0168	0.0209	0.0826	0.7809	0.2443	0.2752
VA35	0.2561	16.3553	6.4915	0.8092	31.7887	2.3153	20.7545	34.548	1.6171	56.0605	0.0214	0.0243	0.4173	0.9337	0.0099	0.0139	0.0357	0.6962	0.0561	0.4327
VA59	0.3463	13.2425	4.9036	0.801	19.3605	1.6699	15.6711	22.5411	1.3365	38.7546	0.0362	0.0377	0.7112	0.9405	0.0167	0.0245	0.0606	0.712	0.0471	0.4172
VA85	0.5852	9.185	10.8716	0.2952	8.5237	7.9454	17.9237	18.2041	0.8438	39.4202	0.009	0.0203	0.0263	0.4002	0.0288	0.065	0.0569	0.4703	1.4108	1.2667
VA99	0.5793	19.4864	7.732	2.0312	41.0777	7.758	28.9297	49.5662	1.7025	76.4231	0.0432	0.0502	0.2897	0.8437	0.0223	0.0285	0.087	0.7381	0.1678	0.3679
VAW6	0.3565	14.2146	7.5641	1.6739	47.3423	7.2165	19.882	54.262	2.691	72.7638	0.0304	0.0364	0.277	0.8738	0.0148	0.0302	0.0441	0.629	0.1245	0.5967
W117HT	0.6935	8.146	4.5446	0.988	35.3791	10.2402	14.8219	46.6836	3.4022	63.0906	0.0214	0.0284	0.103	0.7797	0.0575	0.0887	0.1644	0.6368	0.2534	0.5724
W153R	0.1639	3.3803	5.8748	0.8509	14.8168	9.1622	11.7775	26.204	3.2671	39.6288	0.0317	0.0589	0.1024	0.5532	0.0135	0.0526	0.0252	0.2405	0.7947	3.6317
W182B	0.5514	17.5781	3.9298	0.9792	29.0841	2.2441	22.2219	32.0312	1.4347	53.9263	0.0309	0.0331	0.5005	0.9307	0.0247	0.0298	0.1528	0.8463	0.058	0.2073
W22	0.2482	4.4935	10.5356	1.3632	39.1732	9.8693	14.0728	53.2365	3.574	66.3439	0.0255	0.0317	0.1112	0.7344	0.0135	0.0469	0.0171	0.2146	0.3378	2.5701
W22_R-r:std	0.2481	11.1473	6.8667	0.5215	15.4058	9.4125	18.8691	27.0433	1.1238	46.8828	0.0131	0.0244	0.031	0.5284	0.0167	0.0271	0.0331	0.6037	0.8423	0.6875
W64A	0.2488	6.2839	8.2235	1.0067	27.6679	8.6298	13.6617	36.2387	3.0629	50.4379	0.0266	0.0334	0.1119	0.7521	0.0163	0.0342	0.0272	0.3454	0.3039	1.9041
WD	0.4478	11.6365	3.9546	1.0203	16.9201	2.2051	17.0123	20.8537	1.1679	38.8572	0.0512	0.0571	0.4271	0.8992	0.0317	0.0398	0.1506	0.7733	0.0906	0.3244
WF9	0.7602	9.3178	6.2982	1.247	49.2665	1.4087	14.8626	51.9545	4.0711	67.8038	0.0244	0.0278	0.8857	0.9609	0.0377	0.0715	0.0796	0.5409	0.0268	0.855
YU796_NS	0.2115	8.1464	8.3934	1.2623	40.693	8.227	17.6292	51.5245	2.8553	67.2575	0.0249	0.0298	0.1499	0.8048	0.0105	0.019	0.0255	0.4668	0.2984	1.1907

Table S2 Genomic information for the 60 *a priori* candidate genes. Genes that were identified in the pathway level analysis are highlighted in yellow.

a priori candidate gene pathway	RefGen_v2 Gene ID	RefGen_v2 Annotated Gene Function	RefGen_v2 Chromosome	RefGen_v2 ORF start bp	RefGen_v2 ORF stop bp
Aromatic Head Group	GRMZM2G573867	3-dehydroquinate synthase	2	196,359,627	196,363,692
Aromatic Head Group	GRMZM2G051129	3-dehydroquinate synthase	7	175,831,402	175,835,185
Aromatic Head Group	GRMZM5G877500	3-phosphoshikimate 1-carboxyvinyltransferase	9	22,677,939	22,681,262
Aromatic Head Group	GRMZM2G084942	arogenate dehydrogenase isoform 2	5	59,290,440	59,303,420
Aromatic Head Group	GRMZM2G085117	arogenate dehydrogenase isoform 2	5	59,309,561	59,311,237
Aromatic Head Group	GRMZM2G365961	arogenate dehydrogenase isoform 2	6	85,796,094	85,797,825
Aromatic Head Group	GRMZM2G028369	chorismate mutase	3	194,545,569	194,549,360
Aromatic Head Group	AC198937.4_FG003	chorismate mutase	4	83,615,531	83,617,710
Aromatic Head Group	GRMZM2G179454	chorismate mutase	5	92,183,288	92,186,615
Aromatic Head Group	GRMZM2G124365	chorismate mutase	8	173,103,213	173,107,748
Aromatic Head Group	GRMZM2G116087	chorismate mutase	8	173,111,772	173,115,806
Aromatic Head Group	GRMZM2G121546	chorismate mutase/prephenate dehydratase	7	152,703,579	152,742,017
Aromatic Head Group	GRMZM2G164562	chorismate synthase	1	35,455,268	35,465,683
Aromatic Head Group	GRMZM2G036861	chorismate synthase	9	141,935,141	141,941,423
Aromatic Head Group	GRMZM2G448446	fumarylacetoacetate hydrolase domain-containing protein 1	1	262,231,128	262,234,671
Aromatic Head Group	GRMZM2G156516	fumarylacetoacetate hydrolase domain-containing protein 1	1	295,533,612	295,536,939
Aromatic Head Group	GRMZM2G154093	homogentisate 1,2-dioxygenase	9	28,610,042	28,612,570
Aromatic Head Group	GRMZM2G138624	isochorismatase hydrolase	4	133,923,290	133,925,994
Aromatic Head Group	GRMZM2G128880	isochorismatase hydrolase	5	182,244,213	182,248,069
Aromatic Head Group	GRMZM2G437912	prephenate dehydratase	2	59,037,243	59,039,178
Aromatic Head Group	GRMZM2G466543	prephenate dehydratase	2	165,872,056	165,874,345
Aromatic Head Group	GRMZM2G125923	prephenate dehydratase	10	113,515,151	113,516,898
Aromatic Head Group	GRMZM2G324297	prephenate dehydrogenase	9	61,355,950	61,358,310
Aromatic Head Group	GRMZM2G314652	shikimate biosynthesis protein aroDE	3	71,260,323	71,320,829
Aromatic Head Group	GRMZM5G804881	shikimate dehydrogenase	3	133,182,725	133,187,459
Aromatic Head Group	GRMZM2G014376	shikimate dehydrogenase	10	25,975,417	25,980,229
Aromatic Head Group	GRMZM2G004590	shikimate kinase	2	6,482,881	6,486,234
Aromatic Head Group	GRMZM2G161566	shikimate kinase	4	181,725,008	181,727,763
Aromatic Head Group	GRMZM2G070218	shikimate kinase	5	207,704,956	207,707,881
Aromatic Head Group	GRMZM2G002652	tyrosine transaminase	4	210,690,188	210,693,147
Prenyl Group Synthesis	GRMZM2G027059	4-hydroxy-3-methhylbut-2-enyldiphosphate reductase	1	272,936,836	272,940,502
Prenyl Group Synthesis	GRMZM2G056975	1-deoxy-D-xylulose 5-phosphate reductoisomerase	3	30,226,804	30,233,358
Prenyl Group Synthesis	GRMZM2G036290	1-deoxy-D-xylulose 5-phosphate reductoisomerase	8	8,094,442	8,101,055
Prenyl Group Synthesis	GRMZM2G137151	1-deoxy-D-xylulose 5-phosphate synthase	6	146,378,393	146,382,661
Prenyl Group Synthesis	GRMZM2G493395	1-deoxy-D-xylulose 5-phosphate synthase	7	14,077,852	14,081,075
Prenyl Group Synthesis	GRMZM2G173641	1-deoxy-D-xylulose 5-phosphate synthase 1	9	20,462,059	20,467,072
Prenyl Group Synthesis	GRMZM5G835542	2-C-methyl-D-erythritol 2,4-cyclodiphosphate synthase	4	155,830,779	155,832,786
Prenyl Group Synthesis	AC209374.4_FG002	2-C-methyl-D-erythritol 2,4-cyclodiphosphate synthase	5	196,279,295	196,281,037
Prenyl Group Synthesis	GRMZM5G856881	2-C-methyl-D-erythritol 4-phosphate cytidyltransferase	3	170,115,790	170,118,780
Prenyl Group Synthesis	GRMZM2G172032	2-C-methyl-D-erythritol 4-phosphate cytidyltransferase	8	164,748,939	164,752,371
Prenyl Group Synthesis	GRMZM5G859195	4-diphosphocytidyl-2-C-methyl-D-erythritol kinase	3	187,922,271	187,927,591
Prenyl Group Synthesis	GRMZM2G170734	chlorophyllase-2, chloroplast	7	62,130,993	62,132,323
Prenyl Group Synthesis	GRMZM2G105644	geranylgeranyl hydrogenase	5	206,890,298	206,892,838
Prenyl Group Synthesis	GRMZM2G419111	geranylgeranyl hydrogenase	3	40,062,008	40,064,270
Prenyl Group Synthesis	AC194970.5_FG001	geranylgeranyl pyrophosphate synthase 1	2	207,236,995	207,238,335
Prenyl Group Synthesis	GRMZM2G102550	geranylgeranyl pyrophosphate synthase 2	7	160,531,537	160,533,586
Prenyl Group Synthesis	GRMZM2G058404	geranylgeranyl pyrophosphate synthase 3	8	6,358,798	6,360,117
Prenyl Group Synthesis	GRMZM2G137409	hydroxymethylbutenyl 4-diphosphate synthase	5	182,124,005	182,130,631
Prenyl Group Synthesis	GRMZM2G133082	isopentenyl pyrophosphate isomerase	6	147,131,116	147,136,679
Prenyl Group Synthesis	GRMZM2G108285	isopentenyl pyrophosphate isomerase	7	155,559,747	155,562,921
Prenyl Group Synthesis	GRMZM2G145029	isopentenyl pyrophosphate isomerase	8	104,659,886	104,663,941
Tocochromanol Pathway	GRMZM2G374213	4-hydroxyphenylpyruvate dioxygenase	7	28,816,143	28,818,564
Tocochromanol Pathway	GRMZM2G088396	4-hydroxyphenylpyruvate dioxygenase 1	5	83,859,479	83,861,633
Tocochromanol Pathway	GRMZM2G048472	HGA phytyl transferase	9	107,409,674	107,419,362
Tocochromanol Pathway	GRMZM5G848876	homogentisic acid geranylgeranyl transferase 2	3	6,589,628	6,591,631
Tocochromanol Pathway	GRMZM2G173358	homogentisic acid geranylgeranyl transferase 1	9	92,483,549	92,487,268
Tocochromanol Pathway	GRMZM2G082998	MPBQ/MSBQ methyl transferase	1	173,991,404	173,993,446
Tocochromanol Pathway	GRMZM2G104538	phytol kinase	2	2,495,821	2,497,668
Tocochromanol Pathway	GRMZM2G009785	tocopherol cyclase	5	133,501,928	133,518,495
Tocochromanol Pathway	GRMZM2G035213	γ-tocopherol methyltransferase	5	200,367,029	200,370,851

Table S3 Correlation matrix for untransformed BLUPs of the 20 tocochromanol grain traits. Pearson correlation coefficients are presented in the upper triangle, while the corresponding P-values for the significance of associations (α = 0.05) are displayed below the diagonal.

Trait	δT3	γT3	αT3	δT	γT	αT	Total Tocotrienols	Total Tocopherols	Total Tocopherols/Total Tocotrienols	Total Tocochromanols	δT/(γT+αT)	δT/γT	δT/αT	γT/(γT+αT)	δT3/(γT3+αT3)	δT3/γT3	δT3/αT3	γT3/(γT3+αT3)	αT/γT	αT3/γT3
**δT3**		0.64	0.22	0.16	-0.01	-0.03	0.66	-0.01	-0.37	0.33	0.21	0.21	0.06	0.05	0.81	0.55	0.82	0.44	-0.02	-0.29
**γT3**	0.00		0.22	0.23	0.14	-0.10	0.94	0.10	-0.53	0.58	0.24	0.19	0.08	0.19	0.41	0.12	0.49	0.73	-0.17	-0.49
**αT3**	0.00	0.00		-0.07	-0.07	0.40	0.48	0.08	-0.38	0.31	-0.18	-0.07	-0.27	-0.36	-0.06	-0.02	-0.15	-0.20	0.35	0.03
**δT**	0.01	0.00	0.29		0.68	-0.19	0.20	0.62	0.28	0.60	0.74	0.52	0.49	0.52	0.15	0.06	0.22	0.23	-0.48	-0.14
**γT**	0.91	0.02	0.27	0.00		-0.13	0.10	0.93	0.42	0.79	0.19	-0.03	0.39	0.61	-0.07	-0.15	0.04	0.19	-0.55	-0.12
**αT**	0.62	0.10	0.00	0.00	0.03		0.01	0.20	0.08	0.17	-0.40	-0.17	-0.61	-0.72	-0.17	-0.04	-0.20	-0.37	0.65	0.27
**Total Tocotrienols**	0.00	0.00	0.00	0.00	0.12	0.86		0.10	-0.58	0.61	0.18	0.17	0.00	0.07	0.41	0.16	0.42	0.57	-0.05	-0.41
**Total Tocopherols**	0.85	0.12	0.23	0.00	0.00	0.00	0.11		0.45	0.84	0.07	-0.07	0.18	0.34	-0.12	-0.15	-0.02	0.05	-0.31	-0.02
**Total Tocopherols/Total Tocotirenols**	0.00	0.00	0.00	0.00	0.00	0.20	0.00	0.00		0.05	0.03	-0.02	0.14	0.17	-0.25	-0.02	-0.28	-0.50	-0.15	0.50
**Total Tocochromanols**	0.00	0.00	0.00	0.00	0.00	0.01	0.00	0.00	0.43		0.16	0.04	0.15	0.31	0.13	-0.03	0.20	0.35	-0.27	-0.24
**δT/(γT+αT)**	0.00	0.00	0.00	0.00	0.00	0.00	0.00	0.24	0.64	0.01		0.88	0.53	0.45	0.28	0.17	0.28	0.32	-0.42	-0.22
**δT/γT**	0.00	0.00	0.26	0.00	0.59	0.01	0.01	0.29	0.70	0.53	0.00		0.25	0.10	0.27	0.22	0.20	0.17	-0.09	-0.11
**δT/αT**	0.32	0.23	0.00	0.00	0.00	0.00	0.96	0.00	0.02	0.02	0.00	0.00		0.65	0.16	0.01	0.19	0.27	-0.51	-0.22
**γT/(γT+αT)**	0.46	0.00	0.00	0.00	0.00	0.00	0.29	0.00	0.01	0.00	0.00	0.11	0.00		0.13	-0.01	0.20	0.42	-0.95	-0.28
**δT3/(γT3+αT3)**	0.00	0.00	0.31	0.02	0.26	0.01	0.00	0.06	0.00	0.05	0.00	0.00	0.01	0.04		0.81	0.87	0.39	-0.09	-0.24
**δT3/γT3**	0.00	0.07	0.73	0.33	0.02	0.52	0.01	0.02	0.74	0.61	0.01	0.00	0.84	0.89	0.00		0.54	-0.04	0.03	0.23
**δT3/αT3**	0.00	0.00	0.02	0.00	0.53	0.00	0.00	0.71	0.00	0.00	0.00	0.00	0.00	0.00	0.00	0.00		0.55	-0.15	-0.32
**γT3/(γT3+αT3)**	0.00	0.00	0.00	0.00	0.00	0.00	0.00	0.41	0.00	0.00	0.00	0.01	0.00	0.00	0.00	0.58	0.00		-0.37	-0.81
**αT/γT**	0.70	0.01	0.00	0.00	0.00	0.00	0.41	0.00	0.02	0.00	0.00	0.15	0.00	0.00	0.16	0.68	0.01	0.00		0.24
**αT3/γT3**	0.00	0.00	0.67	0.02	0.05	0.00	0.00	0.74	0.00	0.00	0.00	0.09	0.00	0.00	0.00	0.00	0.00	0.00	0.00	

Table S4 Statistically significant results from the genome-wide association study of 20 tocochromanol grain traits. SNPs that were significantly associated with the indicated trait at 5% FDR are demarcated with boldface font and those significant only at 10% FDR without boldface font.

a priori candidate gene pathway	RefGen_v2 Gene ID	Annotated gene containing associated SNP or gene within 3kb of associated SNP	Trait	SNP ID	SNP Source	Chr	Position in RefGen_v2	*P*-value	FDR-Adjusted *P*-value	Identified in Li et al. (2012)	Minor Allele Frequency (MAF)	Sample Size	MAF Tropical (18% of 252 Lines)	MAF Temperate (82% of 252 Lines)	R^2_{LR} from Model without SNP	R^2_{LR} from Model with SNP	Effect Size	Lambda from Box-Cox Procedure	Back-Transformed Effect Estimates
			αT/γT	S1_16043898	GBS	1	16,043,898	7.25E-06	7.46E-02	-	0.10	246	0.09	0.13	0.24	0.31	-0.05	0.15	-0.29
			δT/αT	S1_42184756	GBS	1	42,184,756	7.43E-06	9.87E-02	-	0.20	246	0.05	0.24	0.28	0.34	0.38	Log	1.26
			αT	S1_69880751	GBS	1	69,880,751	8.96E-06	8.87E-02	-	0.33	251	0.41	0.40	0.25	0.32	-0.19	0.40	-0.41
			αT/γT	S1_207726397	GBS	1	207,726,397	3.89E-06	5.16E-02	-	0.10	246	0.41	0.07	0.24	0.31	-0.07	0.15	-0.38
			αT/γT	S1_207726398	GBS	1	207,726,398	3.89E-06	5.16E-02	-	0.10	246	0.41	0.07	0.24	0.31	0.07	0.15	0.57
			δT3/(γT3+αT3)	S2_71606588	GBS	2	71,606,588	6.14E-06	9.55E-02	-	0.08	248	0.21	0.07	0.10	0.18	0.05	-0.10	-0.39
			αT	S2_196514167	GBS	2	196,514,167	7.19E-06	8.12E-02	-	0.06	251	0.02	0.07	0.25	0.32	-0.36	0.40	-0.67
			αT	S2_219396629	GBS	2	219,396,629	8.33E-06	8.74E-02	-	0.16	251	0.00	0.23	0.25	0.32	-0.27	0.40	-0.54
			αT	S2_219396633	GBS	2	219,396,633	8.33E-06	8.74E-02	-	0.16	251	0.00	0.23	0.25	0.32	0.27	0.40	0.82
			γT/(γT+αT)	ss196506839	55K	3	197,518,747	5.72E-06	9.34E-02	-	0.26	251	0.27	0.26	0.29	0.35	0.09	2.00	0.04
			δT3	S5_132656905	GBS	5	132,656,905	2.15E-06	8.98E-02	-	0.48	247	0.36	0.44	0.12	0.20	0.06	-0.20	-0.25
			δT3/(γT3+αT3)	S5_133189938	GBS	5	133,189,938	2.29E-06	6.85E-02	-	0.41	248	0.19	0.46	0.10	0.18	-0.03	-0.10	0.36
	GRMZM2G105494	transcription factor	δT3	S5_133331096	GBS	5	133,331,096	1.01E-06	7.37E-02	-	0.40	247	0.44	0.41	0.12	0.21	-0.06	-0.20	0.36
	GRMZM2G105494	transcription factor	δT3	S5_133331106	GBS	5	133,331,106	1.01E-06	7.37E-02	-	0.40	247	0.47	0.41	0.12	0.21	0.06	-0.20	-0.25
	**GRMZM2G105494**	**transcription factor**	**δT3**	**S5_133333397**	**GBS**	**5**	**133,333,397**	**3.06E-07**	**4.56E-02**	**-**	**0.43**	**247**	**0.47**	**0.43**	**0.12**	**0.22**	**0.06**	**-0.20**	**-0.25**
	GRMZM2G105494	transcription factor	δT3	ss196465626	55K	5	133,333,397	1.69E-06	8.25E-02	-	0.42	247	0.47	0.42	0.12	0.21	0.06	-0.20	-0.25
	GRMZM2G105494	transcription factor	δT3/(γT3+αT3)	S5_133333397	GBS	5	133,333,397	6.13E-06	9.55E-02	-	0.43	248	0.47	0.43	0.10	0.18	0.03	-0.10	-0.26
	**GRMZM2G105494**	**transcription factor**	**δT3**	**S5_133333561**	**GBS**	**5**	**133,333,561**	**3.12E-07**	**4.56E-02**	**-**	**0.42**	**247**	**0.48**	**0.43**	**0.12**	**0.22**	**0.06**	**-0.20**	**-0.25**
	GRMZM2G105494	transcription factor	δT3/(γT3+αT3)	S5_133333561	GBS	5	133,333,561	8.28E-06	9.55E-02	-	0.42	248	0.48	0.43	0.10	0.17	0.03	-0.10	-0.26
	GRMZM2G105494	transcription factor	δT3	S5_133335078	GBS	5	133,335,078	1.34E-06	7.85E-02	-	0.30	247	0.33	0.29	0.12	0.21	-0.06	-0.20	0.36
Tocochromanol Pathway	GRMZM2G009785	tocopherol cyclase	δT3/(γT3+αT3)	S5_133499169	GBS	5	133,499,169	1.77E-06	6.49E-02	-	0.39	248	0.15	0.44	0.10	0.19	-0.03	-0.10	0.36
Tocochromanol Pathway	GRMZM2G009785	tocopherol cyclase	δT3/(γT3+αT3)	S5_133499269	GBS	5	133,499,269	3.66E-06	8.95E-02	-	0.39	248	0.18	0.46	0.10	0.18	0.03	-0.10	-0.26
**Tocochromanol Pathway**	**GRMZM2G009785**	**tocopherol cyclase**	**δT3/(γT3+αT3)**	**S5_133501858**	**GBS**	**5**	**133,501,858**	**1.29E-07**	**3.78E-02**	**-**	**0.40**	**248**	**0.19**	**0.46**	**0.10**	**0.21**	**0.04**	**-0.10**	**-0.32**
Tocochromanol Pathway	GRMZM2G009785	tocopherol cyclase	δT3/(γT3+αT3)	S5_133501992	GBS	5	133,501,992	9.72E-07	5.53E-02	-	0.40	248	0.14	0.46	0.10	0.19	0.03	-0.10	-0.26
Tocochromanol Pathway	GRMZM2G009785	tocopherol cyclase	δT3/(γT3+αT3)	PZB00969.1	4K	5	133,502,506	8.27E-07	5.53E-02	-	0.41	248	0.14	0.46	0.10	0.19	0.03	-0.10	-0.26
Tocochromanol Pathway	GRMZM2G009785	tocopherol cyclase	δT3/(γT3+αT3)	ss196465634	55K	5	133,510,613	7.54E-06	9.55E-02	-	0.41	248	0.14	0.46	0.10	0.17	-0.03	-0.10	0.36
Tocochromanol Pathway	GRMZM2G009785	tocopherol cyclase	δT3/(γT3+αT3)	PZB02491.1	4K	5	133,517,065	6.69E-06	9.55E-02	-	0.40	248	0.14	0.46	0.10	0.18	-0.03	-0.10	0.36
			δT3/(γT3+αT3)	S5_133618308	GBS	5	133,618,308	7.89E-06	9.55E-02	-	0.40	248	0.10	0.48	0.10	0.17	0.03	-0.10	-0.26
			δT3/(γT3+αT3)	S5_133618309	GBS	5	133,618,309	7.89E-06	9.55E-02	-	0.40	248	0.10	0.48	0.10	0.17	0.03	-0.10	-0.26
			δT3/(γT3+αT3)	S5_133618344	GBS	5	133,618,344	7.89E-06	9.55E-02	-	0.40	248	0.10	0.48	0.10	0.17	0.03	-0.10	-0.26
			δT3/(γT3+αT3)	S5_133691297	GBS	5	133,691,297	5.02E-06	9.55E-02	-	0.37	248	0.10	0.46	0.10	0.18	0.03	-0.10	-0.26
			δT3/(γT3+αT3)	S5_133776316	GBS	5	133,776,316	6.51E-07	5.53E-02	-	0.39	248	0.11	0.46	0.10	0.19	-0.03	-0.10	0.36
			δT3/(γT3+αT3)	S5_134191258	GBS	5	134,191,258	1.66E-06	6.49E-02	-	0.40	248	0.12	0.47	0.10	0.19	0.03	-0.10	-0.26
			δT3/(γT3+αT3)	S5_134195301	GBS	5	134,195,301	2.92E-06	7.77E-02	-	0.39	248	0.14	0.47	0.10	0.18	-0.03	-0.10	0.36
			δT3/(γT3+αT3)	S5_134471200	GBS	5	134,471,200	1.13E-06	5.53E-02	-	0.40	248	0.13	0.46	0.10	0.19	-0.03	-0.10	0.36
			δT3/(γT3+αT3)	S5_134489952	GBS	5	134,489,952	1.13E-06	5.53E-02	-	0.40	248	0.14	0.47	0.10	0.19	-0.03	-0.10	0.36
			δT3/(γT3+αT3)	S5_134751850	GBS	5	134,751,850	2.34E-06	6.85E-02	-	0.43	248	0.21	0.47	0.10	0.18	-0.03	-0.10	0.36
			δT3/(γT3+αT3)	S5_134751883	GBS	5	134,751,883	7.69E-06	9.55E-02	-	0.43	248	0.14	0.45	0.10	0.17	0.03	-0.10	-0.26
			δT3/(γT3+αT3)	S5_134751924	GBS	5	134,751,924	6.83E-06	9.55E-02	-	0.43	248	0.21	0.46	0.10	0.18	-0.03	-0.10	0.36
			δT3/(γT3+αT3)	S5_134787527	GBS	5	134,787,527	6.62E-06	9.55E-02	-	0.33	248	0.08	0.42	0.10	0.18	-0.03	-0.10	0.36
			δT3/(γT3+αT3)	S5_135908766	GBS	5	135,908,766	8.47E-06	9.55E-02	-	0.36	248	0.41	0.39	0.10	0.17	-0.03	-0.10	0.36
			δT3/(γT3+αT3)	S5_135908783	GBS	5	135,908,783	8.47E-06	9.55E-02	-	0.36	248	0.41	0.39	0.10	0.17	-0.03	-0.10	0.36
	**GRMZM2G031952**	**ELMO domain-containing protein 2**	**γT/(γT+αT)**	**S5_190739882**	**GBS**	**5**	**190,739,882**	**1.15E-06**	**2.42E-02**	**-**	**0.24**	**251**	**0.03**	**0.34**	**0.29**	**0.36**	**-0.08**	**2.00**	**-0.04**
	GRMZM2G031952	ELMO domain-containing protein 2	αT/γT	S5_190739882	GBS	5	190,739,882	7.45E-06	7.46E-02	-	0.25	246	0.03	0.34	0.24	0.31	0.04	0.15	0.3
	**GRMZM2G031952**	**ELMO domain-containing protein 2**	**γT/(γT+αT)**	**S5_190739951**	**GBS**	**5**	**190,739,951**	**9.59E-08**	**3.13E-03**	**-**	**0.24**	**251**	**0.05**	**0.30**	**0.29**	**0.37**	**0.09**	**2.00**	**0.04**
	**GRMZM2G031952**	**ELMO domain-containing protein 2**	**αT/γT**	**S5_190739951**	**GBS**	**5**	**190,739,951**	**5.40E-07**	**1.21E-02**	**-**	**0.24**	**246**	**0.05**	**0.30**	**0.24**	**0.33**	**-0.05**	**0.15**	**-0.29**
	**GRMZM2G058943**	**Hypothetical gene of unknown function**	**αT**	**S5_198205644**	**GBS**	**5**	**198,205,644**	**3.56E-06**	**4.98E-02**	**-**	**0.18**	**251**	**0.15**	**0.19**	**0.25**	**0.32**	**-0.25**	**0.40**	**-0.51**
	**GRMZM5G823157**	**WYRKY transciription factor**	**αT**	**S5_199647093**	**GBS**	**5**	**199,647,093**	**3.54E-06**	**4.98E-02**	**-**	**0.24**	**251**	**0.28**	**0.36**	**0.25**	**0.32**	**0.21**	**0.40**	**0.61**
	GRMZM5G823157	WYRKY transciription factor	δT/αT	S5_199647093	GBS	5	199,647,093	4.26E-06	7.78E-02	-	0.23	246	0.28	0.36	0.28	0.34	-0.35	Log	-0.8
		**intergenic region**	**αT/γT**	**S5_199706178**	**GBS**	**5**	**199,706,178**	**1.07E-06**	**1.84E-02**	**-**	**0.14**	**246**	**0.29**	**0.22**	**0.24**	**0.32**	**0.05**	**0.15**	**0.38**
		**intergenic region**	**γT/(γT+αT)**	**S5_199706178**	**GBS**	**5**	**199,706,178**	**1.22E-06**	**2.42E-02**	**-**	**0.15**	**251**	**0.29**	**0.22**	**0.29**	**0.36**	**-0.09**	**2.00**	**-0.05**
		**intergenic region**	**δT/αT**	**S5_199706178**	**GBS**	**5**	**199,706,178**	**1.68E-06**	**3.79E-02**	**-**	**0.15**	**246**	**0.29**	**0.22**	**0.28**	**0.35**	**-0.45**	**Log**	**-0.99**
			αT/γT	S5_199706244	GBS	5	199,706,244	8.86E-06	8.08E-02	-	0.13	246	0.33	0.18	0.24	0.31	-0.05	0.15	-0.29
	**GRMZM2G108996**	**Protein Brevis radix-like 2**	**αT**	**S5_200114137**	**GBS**	**5**	**200,114,137**	**2.63E-07**	**6.44E-03**	**-**	**0.35**	**251**	**0.39**	**0.42**	**0.25**	**0.34**	**-0.22**	**0.40**	**-0.46**
	**GRMZM2G034876**	**growth-regulating factor 1**	**γT/(γT+αT)**	**S5_200293693**	**GBS**	**5**	**200,293,693**	**1.24E-06**	**2.42E-02**	**-**	**0.42**	**251**	**0.34**	**0.37**	**0.29**	**0.36**	**-0.07**	**2.00**	**-0.04**
	**GRMZM2G034876**	**growth-regulating factor 1**	**αT**	**S5_200293693**	**GBS**	**5**	**200,293,693**	**3.33E-06**	**4.98E-02**	**-**	**0.41**	**251**	**0.34**	**0.37**	**0.25**	**0.32**	**0.20**	**0.40**	**0.58**
	GRMZM2G034876	growth-regulating factor 1	αT/γT	S5_200293693	GBS	5	200,293,693	8.10E-06	7.63E-02	-	0.40	246	0.34	0.37	0.24	0.31	0.04	0.15	0.3
			δT/αT	ss196468356	55K	5	200,300,836	6.33E-06	9.33E-02	Yes	0.43	246	0.20	0.49	0.28	0.34	0.33	Log	1.06
		**intergenic region**	**αT**	**S5_200318615**	**GBS**	**5**	**200,318,615**	**1.05E-06**	**2.05E-02**	**-**	**0.22**	**251**	**0.27**	**0.44**	**0.25**	**0.33**	**0.25**	**0.40**	**0.75**
		**intergenic region**	**αT/γT**	**S5_200318615**	**GBS**	**5**	**200,318,615**	**2.50E-06**	**3.82E-02**	**-**	**0.22**	**246**	**0.27**	**0.44**	**0.24**	**0.32**	**0.05**	**0.15**	**0.38**
**Tocochromanol Pathway**	**GRMZM2G035213**	**γ-tocopherol methyltransferase**	**αT**	**ss196416269**	**55K**	**5**	**200,367,532**	**7.36E-14**	**7.21E-09**	**Yes**	**0.20**	**251**	**0.13**	**0.22**	**0.25**	**0.44**	**0.40**	**0.40**	**1.32**
**Tocochromanol Pathway**	**GRMZM2G035213**	**γ-tocopherol methyltransferase**	**αT**	**PZB02283.1**	**4K**	**5**	**200,367,532**	**7.36E-14**	**7.21E-09**	**-**	**0.20**	**251**	**0.14**	**0.22**	**0.25**	**0.44**	**0.40**	**0.40**	**1.32**
**Tocochromanol Pathway**	**GRMZM2G035213**	**γ-tocopherol methyltransferase**	**αT/γT**	**PZB02283.1**	**4K**	**5**	**200,367,532**	**9.21E-13**	**8.96E-08**	**-**	**0.21**	**246**	**0.14**	**0.22**	**0.24**	**0.42**	**0.08**	**0.15**	**0.67**
**Tocochromanol**	**GRMZM2G035213**	**γ-tocopherol**	**αT/γT**	**ss196416269**	**55K**	**5**		**9.21E-13**	**8.96E-08**	**Yes**	**0.21**	**246**	**0.13**	**0.22**	**0.24**	**0.42**	**0.08**	**0.15**	**0.67**

Pathway		methyltransferase					200,367,532												
Tocochromanol Pathway	GRMZM2G035213	γ-tocopherol methyltransferase	γT/(γT+αT)	PZB02283.1	4K	5	200,367,532	4.82E-12	4.72E-07	-	0.20	251	0.14	0.22	0.29	0.44	-0.13	2.00	-0.07
Tocochromanol Pathway	GRMZM2G035213	γ-tocopherol methyltransferase	γT/(γT+αT)	ss196416269	55K	5	200,367,532	4.82E-12	4.72E-07	Yes	0.20	251	0.13	0.22	0.29	0.44	-0.13	2.00	-0.07
Tocochromanol Pathway	GRMZM2G035213	γ-tocopherol methyltransferase	δT/αT	PZB02283.1	4K	5	200,367,532	1.88E-11	1.83E-06	-	0.20	246	0.14	0.22	0.28	0.42	-0.63	Log	-1.27
Tocochromanol Pathway	GRMZM2G035213	γ-tocopherol methyltransferase	δT/αT	ss196416269	55K	5	200,367,532	1.88E-11	1.83E-06	Yes	0.20	246	0.13	0.22	0.28	0.42	-0.63	Log	-1.27
Tocochromanol Pathway	GRMZM2G035213	γ-tocopherol methyltransferase	αT	ss196468362	55K	5	200,369,124	7.36E-14	7.21E-09	Yes	0.20	251	0.13	0.22	0.25	0.44	0.40	0.40	1.32
Tocochromanol Pathway	GRMZM2G035213	γ-tocopherol methyltransferase	αT/γT	ss196468362	55K	5	200,369,124	9.21E-13	8.96E-08	Yes	0.21	246	0.13	0.22	0.24	0.42	0.08	0.15	0.67
Tocochromanol Pathway	GRMZM2G035213	γ-tocopherol methyltransferase	γT/(γT+αT)	ss196468362	55K	5	200,369,124	4.82E-12	4.72E-07	Yes	0.20	251	0.13	0.22	0.29	0.44	-0.13	2.00	-0.07
Tocochromanol Pathway	GRMZM2G035213	γ-tocopherol methyltransferase	δT/αT	ss196468362	55K	5	200,369,124	1.88E-11	1.83E-06	Yes	0.20	246	0.13	0.22	0.28	0.42	-0.63	Log	-1.27
Tocochromanol Pathway	GRMZM2G035213	γ-tocopherol methyltransferase	αT	S5_200369481	GBS	5	200,369,481	1.97E-08	9.61E-04	-	0.11	251	0.36	0.09	0.25	0.35	0.38	0.40	1.24
Tocochromanol Pathway	GRMZM2G035213	γ-tocopherol methyltransferase	αT/γT	S5_200369481	GBS	5	200,369,481	7.24E-07	1.51E-02	-	0.10	246	0.36	0.09	0.24	0.32	0.07	0.15	0.57
Tocochromanol Pathway	GRMZM2G035213	γ-tocopherol methyltransferase	γT/(γT+αT)	S5_200369481	GBS	5	200,369,481	6.15E-09	3.62E-04	-	0.12	251	0.36	0.09	0.29	0.39	-0.14	2.00	-0.07
Tocochromanol Pathway	GRMZM2G035213	γ-tocopherol methyltransferase	δT/αT	S5_200369481	GBS	5	200,369,481	7.21E-07	2.10E-02	-	0.12	246	0.36	0.09	0.28	0.35	-0.56	Log	-1.17
Tocochromanol Pathway	GRMZM2G035213	γ-tocopherol methyltransferase	αT	S5_200369508	GBS	5	200,369,508	6.85E-07	1.44E-02	-	0.18	251	0.11	0.22	0.25	0.33	0.28	0.40	0.85
Tocochromanol Pathway	GRMZM2G035213	γ-tocopherol methyltransferase	αT/γT	S5_200369508	GBS	5	200,369,508	9.69E-07	1.77E-02	-	0.18	246	0.11	0.22	0.24	0.32	0.06	0.15	0.47
Tocochromanol Pathway	GRMZM2G035213	γ-tocopherol methyltransferase	γT/(γT+αT)	S5_200369508	GBS	5	200,369,508	6.57E-07	1.61E-02	-	0.18	251	0.11	0.22	0.29	0.36	-0.10	2.00	-0.05
Tocochromanol Pathway	GRMZM2G035213	γ-tocopherol methyltransferase	αT	S5_200369534	GBS	5	200,369,534	2.59E-06	4.22E-02	-	0.09	251	0.00	0.12	0.25	0.32	-0.34	0.40	-0.65
Tocochromanol Pathway	GRMZM2G035213	γ-tocopherol methyltransferase	αT/γT	S5_200369534	GBS	5	200,369,534	5.45E-06	6.37E-02	-	0.09	246	0.00	0.12	0.24	0.31	-0.07	0.15	-0.38
Tocochromanol Pathway	GRMZM2G035213	γ-tocopherol methyltransferase	αT	S5_200369625	GBS	5	200,369,625	5.00E-08	1.63E-03	-	0.30	251	0.48	0.49	0.25	0.35	-0.24	0.40	-0.5
Tocochromanol Pathway	GRMZM2G035213	γ-tocopherol methyltransferase	δT/αT	S5_200369625	GBS	5	200,369,625	2.02E-06	4.22E-02	-	0.30	246	0.48	0.49	0.28	0.35	0.35	Log	1.14
Tocochromanol Pathway	GRMZM2G035213	γ-tocopherol methyltransferase	αT	S5_200369644	GBS	5	200,369,644	9.36E-06	8.87E-02	-	0.12	251	0.12	0.22	0.25	0.32	-0.26	0.40	-0.53
Tocochromanol Pathway	GRMZM2G035213	γ-tocopherol methyltransferase	αT/γT	S5_200369644	GBS	5	200,369,644	9.97E-06	8.32E-02	-	0.13	246	0.12	0.22	0.24	0.31	-0.05	0.15	-0.29
Tocochromanol Pathway	GRMZM2G035213	γ-tocopherol methyltransferase	αT	S5_200369665	GBS	5	200,369,665	9.36E-06	8.87E-02	-	0.12	251	0.12	0.22	0.25	0.32	0.26	0.40	0.78
Tocochromanol Pathway	GRMZM2G035213	γ-tocopherol methyltransferase	αT/γT	S5_200369665	GBS	5	200,369,665	9.97E-06	8.32E-02	-	0.13	246	0.12	0.22	0.24	0.31	0.05	0.15	0.38
Tocochromanol Pathway	GRMZM2G035213	γ-tocopherol methyltransferase	αT	S5_200369667	GBS	5	200,369,667	1.67E-07	4.47E-03	-	0.10	251	0.00	0.20	0.25	0.34	-0.33	0.40	-0.63
Tocochromanol Pathway	GRMZM2G035213	γ-tocopherol methyltransferase	αT/γT	S5_200369667	GBS	5	200,369,667	1.70E-07	5.22E-03	-	0.11	246	0.00	0.20	0.24	0.33	-0.07	0.15	-0.38
Tocochromanol Pathway	GRMZM2G035213	γ-tocopherol methyltransferase	δT/αT	S5_200369667	GBS	5	200,369,667	7.09E-06	9.87E-02	-	0.10	246	0.00	0.20	0.28	0.34	0.48	Log	1.67
Tocochromanol Pathway	GRMZM2G035213	γ-tocopherol methyltransferase	αT	PZB02424.2	4K	5	200,370,309	4.09E-07	9.25E-03	-	0.17	251	0.09	0.19	0.25	0.33	-0.26	0.40	-0.53
Tocochromanol Pathway	GRMZM2G035213	γ-tocopherol methyltransferase	αT	ss196468368	55K	5	200,371,057	6.73E-06	8.08E-02	-	0.14	251	0.13	0.15	0.25	0.32	-0.27	0.40	-0.54
Tocochromanol Pathway	GRMZM2G035213	γ-tocopherol methyltransferase	αT/γT	ss196468368	55K	5	200,371,057	7.37E-06	7.46E-02	-	0.14	246	0.13	0.15	0.24	0.31	-0.05	0.15	-0.29
	GRMZM2G167431	3-ketodihydrosphingosine reductase	γT/(γT+αT)	S5_200382168	GBS	5	200,382,168	9.59E-09	4.03E-04	-	0.39	251	0.38	0.40	0.29	0.39	0.09	2.00	0.04
	GRMZM2G167431	3-ketodihydrosphingosine reductase	αT	S5_200382168	GBS	5	200,382,168	3.01E-08	1.11E-03	-	0.39	251	0.38	0.40	0.25	0.35	-0.25	0.40	-0.51
	GRMZM2G167431	3-ketodihydrosphingosine reductase	αT/γT	S5_200382168	GBS	5	200,382,168	3.08E-08	1.31E-03	-	0.39	246	0.38	0.40	0.24	0.34	-0.05	0.15	-0.29
	GRMZM2G167431	3-ketodihydrosphingosine reductase	δT/αT	S5_200382168	GBS	5	200,382,168	6.31E-08	2.63E-03	-	0.41	246	0.38	0.40	0.28	0.37	0.41	Log	1.38
	GRMZM2G325001	Mitochondrial uncoupling protein 1	γT/(γT+αT)	S5_200435108	GBS	5	200,435,108	1.13E-10	8.31E-06	-	0.27	251	0.44	0.21	0.29	0.42	0.11	2.00	0.05
	GRMZM2G325001	Mitochondrial uncoupling protein 1	αT/γT	S5_200435108	GBS	5	200,435,108	3.28E-10	2.40E-05	-	0.25	246	0.44	0.21	0.24	0.38	-0.06	0.15	-0.34
	GRMZM2G325001	Mitochondrial uncoupling protein 1	αT	S5_200435108	GBS	5	200,435,108	4.39E-10	3.22E-05	-	0.26	251	0.44	0.21	0.25	0.38	-0.30	0.40	-0.59
	GRMZM2G325001	Mitochondrial uncoupling protein 1	δT/αT	S5_200435108	GBS	5	200,435,108	6.37E-09	4.65E-04	-	0.27	246	0.44	0.21	0.28	0.39	0.47	Log	1.63
	GRMZM2G325001	Mitochondrial uncoupling protein 1	αT	S5_200435117	GBS	5	200,435,117	1.21E-07	3.56E-03	-	0.26	251	0.19	0.28	0.25	0.34	0.25	0.40	0.75
	GRMZM2G325001	Mitochondrial uncoupling protein 1	γT/(γT+αT)	S5_200435117	GBS	5	200,435,117	1.66E-07	4.87E-03	-	0.26	251	0.19	0.28	0.29	0.37	-0.09	2.00	-0.05
	GRMZM2G325001	Mitochondrial uncoupling protein 1	αT/γT	S5_200435117	GBS	5	200,435,117	1.79E-07	5.22E-03	-	0.27	246	0.19	0.28	0.24	0.33	0.05	0.15	0.38
	GRMZM2G325001	Mitochondrial uncoupling protein 1	δT/αT	S5_200435117	GBS	5	200,435,117	7.93E-07	2.10E-02	-	0.25	246	0.19	0.28	0.28	0.35	-0.41	Log	-0.91
	GRMZM2G325019	Pentatricopeptide repeat-containing protein	αT	ss196468352	55K	5	200,435,300	8.32E-10	4.89E-05	Yes	0.40	251	0.39	0.37	0.25	0.38	-0.27	0.40	-0.54
	GRMZM2G325019	Pentatricopeptide repeat-containing protein	γT/(γT+αT)	ss196468352	55K	5	200,435,300	7.67E-09	3.76E-04	Yes	0.40	251	0.39	0.37	0.29	0.39	0.09	2.00	0.04
	GRMZM2G325019	Pentatricopeptide repeat-containing protein	αT/γT	ss196468352	55K	5	200,435,300	2.68E-08	1.31E-03	Yes	0.39	246	0.39	0.37	0.24	0.35	-0.05	0.15	-0.29
	GRMZM2G325019	Pentatricopeptide repeat-containing protein	δT/αT	ss196468352	55K	5	200,435,300	2.87E-08	1.67E-03	Yes	0.41	246	0.39	0.37	0.28	0.38	0.42	Log	1.42
	GRMZM2G325019	Pentatricopeptide repeat-containing protein	αT	S5_200437468	GBS	5	200,437,468	2.29E-08	9.61E-04	-	0.28	251	0.45	0.24	0.25	0.35	-0.26	0.40	-0.53
	GRMZM2G325019	Pentatricopeptide repeat-containing protein	γT/(γT+αT)	S5_200437468	GBS	5	200,437,468	4.13E-08	1.52E-03	-	0.28	251	0.45	0.24	0.29	0.38	0.09	2.00	0.04
	GRMZM2G325019	Pentatricopeptide repeat-containing protein	αT/γT	S5_200437468	GBS	5	200,437,468	9.02E-08	3.29E-03	-	0.27	246	0.45	0.24	0.24	0.34	-0.05	0.15	-0.29
	GRMZM2G325019	Pentatricopeptide repeat-containing protein	δT/αT	S5_200437468	GBS	5	200,437,468	3.72E-07	1.26E-02	-	0.29	246	0.45	0.24	0.28	0.36	0.40	Log	1.34
	GRMZM2G325019	Pentatricopeptide repeat-containing protein	αT/γT	ss196517251	55K	5	200,437,606	4.45E-07	1.08E-02	Yes	0.45	246	0.23	0.40	0.24	0.33	-0.04	0.15	-0.24
	GRMZM2G325019	Pentatricopeptide repeat-containing protein	αT	ss196517251	55K	5	200,437,606	1.38E-06	2.38E-02	Yes	0.46	251	0.23	0.40	0.25	0.33	-0.21	0.40	-0.45
	GRMZM2G325019	Pentatricopeptide repeat-containing protein	γT/(γT+αT)	ss196517251	55K	5	200,437,606	2.99E-06	5.18E-02	Yes	0.46	251	0.23	0.40	0.29	0.35	0.07	2.00	0.03
	GRMZM2G325019	Pentatricopeptide repeat-containing protein	δT/αT	ss196517251	55K	5	200,437,606	5.53E-06	9.33E-02	Yes	0.47	246	0.23	0.40	0.28	0.34	0.35	Log	1.14
	GRMZM2G325038	Transcription factor GT-3b	αT	S5_200438801	GBS	5	200,438,801	4.30E-06	5.74E-02	-	0.10	251	0.06	0.13	0.25	0.32	0.31	0.40	0.96
	GRMZM2G325038	Transcription factor GT-3b	αT/γT	S5_200438801	GBS	5	200,438,801	7.67E-06	7.46E-02	-	0.10	246	0.06	0.13	0.24	0.31	0.06	0.15	0.47
	GRMZM2G161641	amino acid permease	αT/γT	S5_204064933	GBS	5	204,064,933	3.15E-08	1.31E-03	-	0.15	246	0.17	0.15	0.24	0.34	-0.06	0.15	-0.34
	GRMZM2G161641	amino acid permease	γT/(γT+αT)	S5_204064933	GBS	5	204,064,933	3.32E-07	8.87E-03	-	0.15	251	0.17	0.15	0.29	0.36	0.10	2.00	0.05
	GRMZM2G161641	amino acid permease	αT	S5_204064933	GBS	5	204,064,933	1.16E-06	2.13E-02	-	0.15	251	0.17	0.15	0.25	0.33	-0.27	0.40	-0.54
	GRMZM2G161641	amino acid permease	δT/αT	S5_204064933	GBS	5	204,064,933	8.84E-07	2.15E-02	-	0.13	246	0.17	0.15	0.28	0.35	0.48	Log	1.67
	GRMZM2G161641	amino acid permease	αT/γT	S5_204065009	GBS	5	204,065,009	3.42E-07	9.07E-03	-	0.13	246	0.13	0.16	0.24	0.33	-0.06	0.15	-0.34
	GRMZM2G161641	amino acid permease	δT/αT	S5_204065009	GBS	5	204,065,009	3.87E-07	1.26E-02	-	0.13	246	0.13	0.16	0.28	0.36	0.48	Log	1.67
	GRMZM2G161641	amino acid permease	γT/(γT+αT)	S5_204065009	GBS	5	204,065,009	1.74E-06	3.20E-02	-	0.13	251	0.13	0.16	0.29	0.35	0.09	2.00	0.04
	GRMZM2G161641	amino acid permease	αT	S5_204065009	GBS	5	204,065,009	4.64E-06	5.92E-02	-	0.13	251	0.13	0.16	0.25	0.32	-0.26	0.40	-0.53
	GRMZM2G161641	amino acid permease	δT/αT	S5_204065055	GBS	5	204,065,055	4.68E-08	2.28E-03	-	0.16	246	0.15	0.20	0.28	0.37	0.49	Log	1.72
	GRMZM2G161641	amino acid permease	αT/γT	S5_204065055	GBS	5	204,065,055	2.36E-06	3.82E-02	-	0.17	246	0.15	0.20	0.24	0.32	-0.05	0.15	-0.29
		intergenic	αT/γT	S6_149776596	GBS	6	149,776,596	9.71E-07	1.77E-02	-	0.17	246	0.03	0.24	0.24	0.32	-0.05	0.15	-0.29

			αT	S6_149776596	GBS	6	149,776,596	6.87E-06	8.08E-02	-	0.16	251	0.03	0.24	0.25	0.32	-0.23	0.40	-0.48
			αT/γT	S6_149776597	GBS	6	149,776,597	4.37E-06	5.54E-02	-	0.14	246	0.00	0.21	0.24	0.31	0.05	0.15	0.38
			αT	S6_149776597	GBS	6	149,776,597	1.03E-05	9.45E-02	-	0.14	251	0.00	0.21	0.25	0.31	0.23	0.40	0.68
		gene of unknown function	αT/γT	ss196475603	55K	7	13,959,219	1.25E-05	9.86E-02	-	0.30	246	0.37	0.30	0.24	0.31	0.04	0.15	0.3
			αT/γT	ss196486649	55K	8	128,548,850	7.50E-06	7.46E-02	-	0.15	246	0.11	0.16	0.24	0.31	0.06	0.15	0.47
	**GRMZM5G833760**	**phytosulfokine receptor**	**γT3**	**S9_92718671**	**GBS**	**9**	**92,718,671**	**4.48E-07**	**4.40E-02**	**-**	**0.08**	**250**	**0.00**	**0.13**	**0.20**	**0.28**	**-0.05**	**0.10**	**-0.4**
	**GRMZM5G833760**	**phytosulfokine receptor**	**γT3**	**S9_92718674**	**GBS**	**9**	**92,718,674**	**4.48E-07**	**4.40E-02**	**-**	**0.08**	**250**	**0.00**	**0.13**	**0.20**	**0.28**	**-0.05**	**0.10**	**-0.4**
	**GRMZM5G833760**	**phytosulfokine receptor**	**γT3**	**S9_92718709**	**GBS**	**9**	**92,718,709**	**4.48E-07**	**4.40E-02**	**-**	**0.08**	**250**	**0.00**	**0.13**	**0.20**	**0.28**	**-0.05**	**0.10**	**-0.4**
		intergenic region	Total Tocotrienols	S9_107839821	GBS	9	107,839,821	2.00E-07	5.88E-02	-	0.20	250	0.38	0.23	0.20	0.30	-0.05	0.15	-0.29
			αT/γT	S9_151726463	GBS	9	151,726,463	4.81E-06	5.86E-02	-	0.08	246	0.03	0.11	0.24	0.31	0.06	0.15	0.47
			αT/γT	S9_151726511	GBS	9	151,726,511	1.07E-05	8.69E-02	-	0.12	246	0.06	0.14	0.24	0.31	-0.05	0.15	-0.29
			αT/γT	S9_151726870	GBS	9	151,726,870	9.55E-06	8.32E-02	-	0.13	246	0.15	0.13	0.24	0.31	0.05	0.15	0.38
			**αT/γT**	**S9_155585508**	**GBS**	**9**	**155,585,508**	**2.62E-06**	**3.82E-02**	**-**	**0.15**	**246**	**0.27**	**0.13**	**0.24**	**0.32**	**0.05**	**0.15**	**0.38**
			δT/αT	S9_155585508	GBS	9	155,585,508	2.75E-06	5.35E-02	-	0.15	246	0.27	0.13	0.28	0.35	-0.45	Log	-0.99
			δT/αT	S10_129705301	GBS	10	129,705,301	6.39E-06	9.33E-02	-	0.08	246	0.05	0.13	0.28	0.34	-0.64	Log	-1.28
			δT/αT	S10_129705304	GBS	10	129,705,304	6.39E-06	9.33E-02	-	0.08	246	0.05	0.13	0.28	0.34	-0.64	Log	-1.28

Table S5 Multi-locus mixed-model (MLMM) results from an analysis of αT, αT/γT, γT/(γT+αT), δT/αT, δT3/(γT3+αT3), and δT3.

Trait	SNP Added to Model	Chr	Position in RefGen_v2	*P*-value	extBIC	R-square_LR[a]	R-square_LR of All Three SNPs[b]
αT	ss196416269	5	200,367,532	7.12E-16	384.47	0.21	0.45
αT	S5_200369534	5	200,369,534	1.87E-14	353.57	0.19	
αT	S5_200369481	5	200,369,481	3.04E-10	338.67	0.14	
αT/γT	ss196416269	5	200,367,532	5.89E-15	-402.01	0.21	0.40
αT/γT	S5_200369534	5	200,369,534	4.08E-12	-424.67	0.17	
αT/γT	S5_200369481	5	200,369,481	1.92E-07	-427.22	0.09	
γT /(γT+αT)	ss196416269	5	200,367,532	7.40E-14	-113.06	0.19	0.38
γT /(γT+αT)	S5_200369481	5	200,369,481	3.25E-10	-126.83	0.14	
γT /(γT+αT)	S5_200369534	5	200,369,534	2.09E-09	-138.31	0.12	
δT/αT	ss196416269	5	200,367,532	2.29E-12	647.28	0.17	0.33
δT/αT	S5_200369534	5	200,369,534	1.08E-08	630.30	0.11	
δT/αT	S5_200369481	5	200,369,481	4.44E-07	619.70	0.09	
δT3/ (γT3+αT3)	S5_133501858	5	133,501,858	7.16E-08	-485.87	0.10	
δT3	S5_133333561	5	133,333,561	2.04E-07	-170.44	0.09	

[a]Defined as the likelihood-ratio based R^2 statistic that measures the increase in phenotypic variation explained by adding the indicated SNP into the model.Table 1

[b]Defined as the likelihood-ratio based R^2 statistic that compares variation explained by the MLMM model with the three SNPs (indicated in Column A) to the intercept-only model.

Table S6a Haplotype effects of three ZmVTE4 SNPs identified with an optimal multi-locus mixed model (MLMM) for αT/γT.

**Haplotype**	**ss196416269**	**S5_200369481**	**S5_200369534**	**Frequency**	**Mean Value**	**S.D.[a]**
A,C,G	A	C	G	50	0.10	0.09
G,C,G	G	C	G	151	0.41	0.27
A,G,G	A	G	G	1	0.06	-
G,G,G	G	G	G	28	0.95	0.46
G,C,T	G	C	T	22	0.16	0.33
**R^2_{LR}[b]**	51.30%					
**Partial R^2_{LR}[c]**	40.47%					
***P*-value[d]**	5.55E-31					
**Maximum Fold Change[e]**	5.94					

[a]S.D., Standard deviation of the BLUPs for each haplotype.

[b]R^2_{LR}, likelihood-ratio based R^2 statistic, percentage of total phenotypic variation explained by the unified mixed model.

[c]Partial R^2_{LR}, likelihood-ratio based partial R^2 statistic, percentage of total phenotypic variation explained by the haplotypes.

[d]The P-value was from a unified mixed linear model that tested for an association between haplotypes and αT levels.

[e]Fold change was calculated as the ratio between the most favorable (G, G, G) and least favorable (G, C, T) haplotypes for αT levels.

Table S6b Haplotype effects of three ZmVTE4 SNPs identified with an optimal multi-locus mixed model (MLMM) for δT/αT.

Haplotype	ss196416269	S5_200369481	S5_200369534	Frequency	Mean Value	S.D.[a]
A,C,G	A	C	G	50	0.55	0.40
G,C,G	G	C	G	151	0.17	0.15
A,G,G	A	G	G	1	0.76	-
G,G,G	G	G	G	28	0.04	0.04
G,C,T	G	C	T	22	0.69	0.38
**R^2_{LR}[b]**	49.57%					
**Partial R^2_{LR}[c]**	39.96%					
***P*-value[d]**	2.16E-27					
**Maximum Fold Change[e]**	0.06					

[a]S.D., Standard deviation of the BLUPs for each haplotype.

[b]R^2_{LR}, likelihood-ratio based R^2 statistic, percentage of total phenotypic variation explained by the unified mixed model.

[c]Partial R^2_{LR}, likelihood-ratio based partial R^2 statistic, percentage of total phenotypic variation explained by the haplotypes.

[d]The P-value was from a unified mixed linear model that tested for an association between haplotypes and αT levels.

[e]Fold change was calculated as the ratio between the most favorable (G, G, G) and least favorable (G, C, T) haplotypes for αT levels.

Table S6c Haplotype effects of three ZmVTE4 SNPs identified with an optimal multi-locus mixed model (MLMM) for γT/(γT+αT).

Haplotype	ss196416269	S5_200369481	S5_200369534	Frequency	Mean Value	S.D.[a]
A,C,G	A	C	G	50	0.91	0.06
G,C,G	G	C	G	151	0.72	0.13
A,G,G	A	G	G	1	0.93	-
G,G,G	G	G	G	28	0.48	0.14
G,C,T	G	C	T	22	0.89	0.15
**R^2_{LR} [b]**	52.4%					
**Partial R^2_{LR} [c]**	41.3%					
***P*-value[d]**	2.16E-31					
**Maximum Fold Change[e]**	0.54					

[a]S.D., Standard deviation of the BLUPs for each haplotype.

[b]R^2_{LR}, likelihood-ratio based R^2 statistic, percentage of total phenotypic variation explained by the unified mixed model.

[c]Partial R^2_{LR}, likelihood-ratio based partial R^2 statistic, percentage of total phenotypic variation explained by the haplotypes.

[d]The P-value was from a unified mixed linear model that tested for an association between haplotypes and αT levels.

[e]Fold change was calculated as the ratio between the most favorable (G, G, G) and least favorable (G, C, T) haplotypes for αT levels.

Table S7a Statistically significant results from the candidate gene association study of 20 tocochromanol grain traits without and with none of the SNPs identified in the multi-locus mixed-model (MLMM) analysis included as covariates. SNPs that were were significantly associated with the indicated trait at 5% FDR are shown.

a priori candidate gene pathway	RefGen_v2 Gene ID	RefGen_v2 Annotated Gene Function	Trait	SNP ID	SNP Source	Chr	Position	Distance from Gene ORF Start	Distance from Gene ORF Finish	*P*-value	FDR Adjusted *P*-value	Minor Allele Frequency (MAF)	MAF Tropical (18% of 252 Lines)	MAF Temperate (82% of 252 Lines)	Sample Size	R^2_{LR} from Model without SNP	R^2_{LR} from Model with SNP	Effect Size	Lambda from Box-Cox Procedure	Back-Transformed Effect Estimates
Aromatic Head Group	GRMZM2G437912	prephenate dehydratase	Total Tocotrienols	S2_59013838	GBS	2	59,013,838	-23,405	-25,340	1.84E-05	3.76E-02	0.06	0.40	0.09	250	0.20	0.26	0.06	0.15	0.47
Aromatic Head Group	GRMZM2G437912	prephenate dehydratase	Total Tocotrienols	S2_59013840	GBS	2	59,013,840	-23,403	-25,338	1.84E-05	3.76E-02	0.06	0.40	0.09	250	0.20	0.26	-0.06	0.15	-0.34
Aromatic Head Group	GRMZM2G573867	3-dehydroquinate synthase	αT	S2_196293028	GBS	2	196,293,028	-66,599	-70,664	2.50E-04	3.97E-02	0.06	0.00	0.08	251	0.25	0.30	-0.26	0.40	-0.53
Aromatic Head Group	GRMZM2G573867	3-dehydroquinate synthase	αT/γT	S2_196295236	GBS	2	196,295,236	-64,391	-68,456	1.82E-04	4.63E-02	0.06	0.04	0.07	246	0.24	0.29	0.06	0.15	0.47
Aromatic Head Group	GRMZM2G573867	3-dehydroquinate synthase	γT/(γT+αT)	S2_196295236	GBS	2	196,295,236	-64,391	-68,456	2.59E-04	5.11E-02	0.06	0.04	0.07	251	0.29	0.33	-0.10	2.00	-0.05
Aromatic Head Group	GRMZM2G573867	3-dehydroquinate synthase	δT/αT	S2_196295236	GBS	2	196,295,236	-64,391	-68,456	1.36E-04	3.62E-02	0.06	0.04	0.07	246	0.28	0.32	-0.49	Log	-1.05
Aromatic Head Group	GRMZM2G573867	3-dehydroquinate synthase	δT/αT	S2_196387288	GBS	2	196,387,288	27,661	23,596	1.60E-04	3.89E-02	0.06	0.05	0.06	246	0.28	0.32	-0.49	Log	-1.05
Aromatic Head Group	GRMZM2G573867	3-dehydroquinate synthase	αT	S2_196514167	GBS	2	196,514,167	154,540	150,475	7.19E-06	2.20E-03	0.06	0.02	0.07	251	0.25	0.32	-0.36	0.40	-0.67
Aromatic Head Group	GRMZM2G573867	3-dehydroquinate synthase	δT/αT	S2_196514167	GBS	2	196,514,167	154,540	150,475	1.51E-04	3.82E-02	0.06	0.02	0.07	246	0.28	0.32	0.51	Log	1.81
Aromatic Head Group	GRMZM2G138624	isochorismatase hydrolase	αT3	ss196456226	55K	4	134,020,078	96,788	94,084	8.16E-06	2.49E-02	0.45	0.45	0.46	248	0.20	0.27	0.01	0.05	0.22
Aromatic Head Group	GRMZM2G124365	chorismate mutase	αT	S8_173041758	GBS	8	173,041,758	-61,455	-65,990	2.38E-04	3.97E-02	0.12	0.18	0.22	251	0.25	0.30	-0.23	0.40	-0.48
Aromatic Head Group	GRMZM2G124365	chorismate mutase	δT/αT	S8_173070782	GBS	8	173,070,782	-32,431	-36,966	1.37E-04	3.62E-02	0.18	0.04	0.33	246	0.28	0.32	-0.36	Log	-0.82
Aromatic Head Group	GRMZM2G124365	chorismate mutase	Total Tocotrienols	S8_173243067	GBS	8	173,243,067	139,854	135,319	6.12E-05	4.69E-02	0.09	0.20	0.09	250	0.20	0.26	0.05	0.15	0.38
Aromatic Head Group	GRMZM2G124365	chorismate mutase	αT3	S8_173243067	GBS	8	173,243,067	139,854	135,319	3.51E-05	3.57E-02	0.09	0.20	0.09	248	0.20	0.26	0.01	0.05	0.22
Prenyl Group Synthesis	GRMZM2G027059	4-hydroxy-3-methylbut-2-enyldiphosphate reductase	δT3	ss196519818	55K	1	272,871,696	-65,140	-68,806	1.40E-04	4.28E-02	0.32	0.09	0.38	247	0.12	0.17	-0.06	-0.20	0.36
Prenyl Group Synthesis	GRMZM2G027059	4-hydroxy-3-methylbut-2-enyldiphosphate reductase	δT3	ss196519822	55K	1	272,874,639	-62,197	-65,863	1.30E-04	4.18E-02	0.33	0.09	0.38	247	0.12	0.17	-0.06	-0.20	0.36
Prenyl Group Synthesis	GRMZM2G027059	4-hydroxy-3-methylbut-2-enyldiphosphate reductase	αT	S1_273098217	GBS	1	273,098,217	161,381	157,715	3.82E-05	8.98E-03	0.22	0.24	0.24	251	0.25	0.31	-0.20	0.40	-0.43
Prenyl Group Synthesis	GRMZM2G137409	hydroxymethylbutenyl 4-diphosphate synthase	δT3/γT3	S5_182040396	GBS	5	182,040,396	-83,609	-90,235	7.94E-05	3.73E-02	0.29	0.00	0.37	249	0.13	0.18	0.01	0.05	0.22
Prenyl Group Synthesis	AC209374.4_FG002	2-C-methyl-D-erythritol 2,4-cyclodiphosphate synthase	αT/γT	S5_196278264	GBS	5	196,278,264	-1,031	-2,773	9.54E-05	2.64E-02	0.25	0.17	0.30	246	0.24	0.29	0.04	0.15	0.3
Prenyl Group Synthesis	AC209374.4_FG002	2-C-methyl-D-erythritol 2,4-cyclodiphosphate synthase	αT	S5_196278264	GBS	5	196,278,264	-1,031	-2,773	1.31E-04	2.76E-02	0.25	0.17	0.30	251	0.25	0.30	0.19	0.40	0.54
Prenyl Group Synthesis	AC209374.4_FG002	2-C-methyl-D-erythritol 2,4-cyclodiphosphate synthase	γT/(γT+αT)	S5_196278264	GBS	5	196,278,264	-1,031	-2,773	4.51E-05	1.45E-02	0.25	0.17	0.30	251	0.29	0.34	-0.07	2.00	-0.04
Prenyl Group Synthesis	AC209374.4_FG002	2-C-methyl-D-erythritol 2,4-cyclodiphosphate synthase	δT/αT	S5_196278264	GBS	5	196,278,264	-1,031	-2,773	2.72E-05	1.03E-02	0.24	0.17	0.30	246	0.28	0.33	-0.36	Log	-0.82
Prenyl Group Synthesis	AC209374.4_FG002	2-C-methyl-D-erythritol 2,4-cyclodiphosphate synthase	γT/(γT+αT)	S5_196508694	GBS	5	196,508,694	229,399	227,657	2.41E-04	5.08E-02	0.41	0.43	0.45	251	0.29	0.33	0.05	2.00	0.02
Prenyl Group Synthesis	GRMZM2G133082	isopentenyl pyrophosphate isomerase	γT/(γT+αT)	S6_147333828	GBS	6	147,333,828	202,712	197,149	1.43E-04	3.25E-02	0.07	0.22	0.05	251	0.29	0.33	-0.11	2.00	-0.06
Prenyl Group Synthesis	GRMZM2G133082	isopentenyl pyrophosphate isomerase	γT/(γT+αT)	S6_147333833	GBS	6	147,333,833	202,717	197,154	1.43E-04	3.25E-02	0.07	0.22	0.05	251	0.29	0.33	-0.11	2.00	-0.06
Prenyl Group Synthesis	GRMZM2G493395	1-deoxy-D-xylulose 5-phosphate synthase	αT/γT	ss196475603	55K	7	13,959,219	-118,633	-121,856	1.25E-05	3.81E-03	0.30	0.37	0.30	246	0.24	0.31	0.04	0.15	0.3
Prenyl Group Synthesis	GRMZM2G493395	1-deoxy-D-xylulose 5-phosphate synthase	γT/(γT+αT)	ss196475603	55K	7	13,959,219	-118,633	-121,856	4.10E-05	1.45E-02	0.31	0.37	0.30	251	0.29	0.34	-0.07	2.00	-0.04
Prenyl Group Synthesis	GRMZM2G172032	2-C-methyl-D-erythritol 4-phosphate cytidyltransferase	δT3/(γT3+αT3)	S8_164626861	GBS	8	164,626,861	-122,078	-125,510	5.26E-05	1.40E-02	0.14	0.50	0.10	248	0.10	0.16	-0.04	-0.10	0.5
Prenyl Group Synthesis	GRMZM2G172032	2-C-methyl-D-erythritol 4-phosphate cytidyltransferase	δT3	S8_164626861	GBS	8	164,626,861	-122,078	-125,510	1.94E-04	4.60E-02	0.14	0.50	0.10	247	0.12	0.17	-0.07	-0.20	0.44
Tocochromanol Pathway	GRMZM2G009785	tocopherol cyclase	δT3/(γT3+αT3)	S5_133331094	GBS	5	133,331,094	-170,834	-187,401	3.48E-05	1.04E-02	0.41	0.47	0.41	248	0.10	0.16	0.03	-0.10	-0.26
Tocochromanol Pathway	GRMZM2G009785	tocopherol cyclase	δT3/αT3	S5_133331094	GBS	5	133,331,094	-170,834	-187,401	4.92E-05	3.33E-02	0.41	0.47	0.41	246	0.11	0.18	0.05	-0.15	-0.28
Tocochromanol	GRMZM2G009785	tocopherol cyclase	δT3	S5_133331094	GBS	5	133,331,094	-170,834	-187,401	4.29E-06	3.27E-03	0.41	0.47	0.41	247	0.12	0.20	0.06	-0.20	-0.25

Pathway																				
Tocochromanol Pathway	GRMZM2G009785	tocopherol cyclase	δT3/(γT3+αT3)	S5_133331096	GBS	5	133,331,096	-170,832	-187,399	1.54E-05	5.53E-03	0.40	0.44	0.41	248	0.10	0.17	-0.03	-0.10	0.36
Tocochromanol Pathway	GRMZM2G009785	tocopherol cyclase	δT3/αT3	S5_133331096	GBS	5	133,331,096	-170,832	-187,399	1.92E-05	2.63E-02	0.40	0.44	0.41	246	0.11	0.18	-0.05	-0.15	0.41
Tocochromanol Pathway	GRMZM2G009785	tocopherol cyclase	δT3	S5_133331096	GBS	5	133,331,096	-170,832	-187,399	1.01E-06	1.54E-03	0.40	0.44	0.41	247	0.12	0.21	-0.06	-0.20	0.36
Tocochromanol Pathway	GRMZM2G009785	tocopherol cyclase	δT3/(γT3+αT3)	S5_133331106	GBS	5	133,331,106	-170,822	-187,389	1.54E-05	5.53E-03	0.40	0.47	0.41	248	0.10	0.17	0.03	-0.10	-0.26
Tocochromanol Pathway	GRMZM2G009785	tocopherol cyclase	δT3/αT3	S5_133331106	GBS	5	133,331,106	-170,822	-187,389	1.92E-05	2.63E-02	0.40	0.47	0.41	246	0.11	0.18	0.05	-0.15	-0.28
Tocochromanol Pathway	GRMZM2G009785	tocopherol cyclase	δT3	S5_133331106	GBS	5	133,331,106	-170,822	-187,389	1.01E-06	1.54E-03	0.40	0.47	0.41	247	0.12	0.21	0.06	-0.20	-0.25
Tocochromanol Pathway	GRMZM2G009785	tocopherol cyclase	δT3/(γT3+αT3)	ss196465630	55K	5	133,332,323	-169,605	-186,172	6.13E-05	1.56E-02	0.32	0.11	0.36	248	0.10	0.16	-0.03	-0.10	0.36
Tocochromanol Pathway	GRMZM2G009785	tocopherol cyclase	δT3	ss196465630	55K	5	133,332,323	-169,605	-186,172	1.96E-04	4.60E-02	0.32	0.11	0.36	247	0.12	0.17	-0.05	-0.20	0.29
Tocochromanol Pathway	GRMZM2G009785	tocopherol cyclase	δT3/(γT3+αT3)	ss196465628	55K	5	133,333,095	-168,833	-185,400	4.22E-05	1.17E-02	0.42	0.47	0.42	248	0.10	0.16	-0.03	-0.10	0.36
Tocochromanol Pathway	GRMZM2G009785	tocopherol cyclase	δT3/αT3	ss196465628	55K	5	133,333,095	-168,833	-185,400	8.98E-05	4.56E-02	0.42	0.47	0.42	246	0.11	0.17	-0.05	-0.15	0.41
Tocochromanol Pathway	GRMZM2G009785	tocopherol cyclase	δT3	ss196465628	55K	5	133,333,095	-168,833	-185,400	3.88E-06	3.27E-03	0.42	0.47	0.42	247	0.12	0.20	-0.06	-0.20	0.36
Tocochromanol Pathway	GRMZM2G009785	tocopherol cyclase	δT3/(γT3+αT3)	S5_133333397	GBS	5	133,333,397	-168,531	-185,098	6.13E-06	3.89E-03	0.43	0.47	0.43	248	0.10	0.18	0.03	-0.10	-0.26
Tocochromanol Pathway	GRMZM2G009785	tocopherol cyclase	δT3/(γT3+αT3)	ss196465626	55K	5	133,333,397	-168,531	-185,098	2.46E-05	8.34E-03	0.42	0.47	0.42	248	0.10	0.17	0.03	-0.10	-0.26
Tocochromanol Pathway	GRMZM2G009785	tocopherol cyclase	δT3/αT3	S5_133333397	GBS	5	133,333,397	-168,531	-185,098	2.16E-05	2.63E-02	0.43	0.47	0.43	246	0.11	0.18	0.05	-0.15	-0.28
Tocochromanol Pathway	GRMZM2G009785	tocopherol cyclase	δT3/αT3	ss196465626	55K	5	133,333,397	-168,531	-185,098	6.02E-05	3.66E-02	0.42	0.47	0.42	246	0.11	0.17	0.05	-0.15	-0.28
Tocochromanol Pathway	GRMZM2G009785	tocopherol cyclase	δT3	S5_133333397	GBS	5	133,333,397	-168,531	-185,098	3.06E-07	9.53E-04	0.43	0.47	0.43	247	0.12	0.22	0.06	-0.20	-0.25
Tocochromanol Pathway	GRMZM2G009785	tocopherol cyclase	δT3	ss196465626	55K	5	133,333,397	-168,531	-185,098	1.69E-06	1.72E-03	0.42	0.47	0.42	247	0.12	0.21	0.06	-0.20	-0.25
Tocochromanol Pathway	GRMZM2G009785	tocopherol cyclase	δT3/(γT3+αT3)	S5_133333561	GBS	5	133,333,561	-168,367	-184,934	8.28E-06	3.89E-03	0.42	0.48	0.43	248	0.10	0.17	0.03	-0.10	-0.26
Tocochromanol Pathway	GRMZM2G009785	tocopherol cyclase	δT3/αT3	S5_133333561	GBS	5	133,333,561	-168,367	-184,934	2.12E-05	2.63E-02	0.42	0.48	0.43	246	0.11	0.18	0.05	-0.15	-0.28
Tocochromanol Pathway	GRMZM2G009785	tocopherol cyclase	δT3	S5_133333561	GBS	5	133,333,561	-168,367	-184,934	3.12E-07	9.53E-04	0.42	0.48	0.43	247	0.12	0.22	0.06	-0.20	-0.25
Tocochromanol Pathway	GRMZM2G009785	tocopherol cyclase	δT3/(γT3+αT3)	S5_133335078	GBS	5	133,335,078	-166,850	-183,417	1.48E-05	5.53E-03	0.30	0.33	0.29	248	0.10	0.17	-0.03	-0.10	0.36
Tocochromanol Pathway	GRMZM2G009785	tocopherol cyclase	δT3/αT3	S5_133335078	GBS	5	133,335,078	-166,850	-183,417	8.11E-05	4.49E-02	0.30	0.33	0.29	246	0.11	0.17	-0.05	-0.15	0.41
Tocochromanol Pathway	GRMZM2G009785	tocopherol cyclase	δT3	S5_133335078	GBS	5	133,335,078	-166,850	-183,417	1.34E-06	1.64E-03	0.30	0.33	0.29	247	0.12	0.21	-0.06	-0.20	0.36
Tocochromanol Pathway	GRMZM2G009785	tocopherol cyclase	δT3/(γT3+αT3)	S5_133338747	GBS	5	133,338,747	-163,181	-179,748	2.78E-05	8.94E-03	0.37	0.17	0.41	248	0.10	0.16	-0.03	-0.10	0.36
Tocochromanol Pathway	GRMZM2G009785	tocopherol cyclase	δT3/γT3	S5_133338747	GBS	5	133,338,747	-163,181	-179,748	6.52E-05	3.73E-02	0.37	0.17	0.41	249	0.13	0.18	0.01	0.05	0.22
Tocochromanol Pathway	GRMZM2G009785	tocopherol cyclase	δT3	S5_133338747	GBS	5	133,338,747	-163,181	-179,748	1.75E-04	4.60E-02	0.37	0.17	0.41	247	0.12	0.17	-0.05	-0.20	0.29
Tocochromanol Pathway	GRMZM2G009785	tocopherol cyclase	δT/γT	PZA00524.2	4K	5	133,338,936	-162,992	-179,559	1.36E-05	3.93E-02	0.41	0.23	0.39	249	0.13	0.20	-0.02	0.30	-0.07
Tocochromanol Pathway	GRMZM2G009785	tocopherol cyclase	δT/γT	S5_133338936	GBS	5	133,338,936	-162,992	-179,559	1.47E-05	3.93E-02	0.47	0.17	0.40	249	0.13	0.20	0.02	0.30	0.07
Tocochromanol Pathway	GRMZM2G009785	tocopherol cyclase	δT3/(γT3+αT3)	S5_133499169	GBS	5	133,499,169	-2,759	-19,326	1.77E-06	2.71E-03	0.39	0.15	0.44	248	0.10	0.19	-0.03	-0.10	0.36
Tocochromanol Pathway	GRMZM2G009785	tocopherol cyclase	δT3/αT3	S5_133499169	GBS	5	133,499,169	-2,759	-19,326	4.65E-05	3.33E-02	0.39	0.15	0.44	246	0.11	0.18	-0.06	-0.15	0.51
Tocochromanol Pathway	GRMZM2G009785	tocopherol cyclase	δT3/γT3	S5_133499169	GBS	5	133,499,169	-2,759	-19,326	1.04E-04	4.54E-02	0.40	0.15	0.44	249	0.13	0.18	0.01	0.05	0.22
Tocochromanol Pathway	GRMZM2G009785	tocopherol cyclase	δT3	S5_133499169	GBS	5	133,499,169	-2,759	-19,326	1.13E-04	4.07E-02	0.40	0.15	0.44	247	0.12	0.18	-0.05	-0.20	0.29
Tocochromanol Pathway	GRMZM2G009785	tocopherol cyclase	δT3/(γT3+αT3)	S5_133499269	GBS	5	133,499,269	-2,659	-19,226	3.66E-06	3.89E-03	0.39	0.18	0.46	248	0.10	0.18	0.03	-0.10	-0.26
Tocochromanol Pathway	GRMZM2G009785	tocopherol cyclase	δT3	S5_133499269	GBS	5	133,499,269	-2,659	-19,226	1.78E-04	4.60E-02	0.40	0.18	0.46	247	0.12	0.17	0.05	-0.20	-0.22
Tocochromanol Pathway	GRMZM2G009785	tocopherol cyclase	δT3/(γT3+αT3)	S5_133501858	GBS	5	133,501,858	-70	-16,637	1.29E-07	7.88E-04	0.40	0.19	0.46	248	0.10	0.21	0.04	-0.10	-0.32
Tocochromanol Pathway	GRMZM2G009785	tocopherol cyclase	δT3/αT3	S5_133501858	GBS	5	133,501,858	-70	-16,637	1.09E-05	2.63E-02	0.40	0.19	0.46	246	0.11	0.19	0.06	-0.15	-0.32

Tocochromanol Pathway	GRMZM2G009785	tocopherol cyclase	δT3/γT3	S5_133501858	GBS	5	133,501,858	-70	-16,637	1.67E-06	9.96E-03	0.39	0.19	0.46	249	0.13	0.21	-0.01	0.05	-0.18
Tocochromanol Pathway	GRMZM2G009785	tocopherol cyclase	δT3	S5_133501858	GBS	5	133,501,858	-70	-16,637	6.01E-06	4.08E-03	0.40	0.19	0.46	247	0.12	0.20	0.06	-0.20	-0.25
Tocochromanol Pathway	GRMZM2G009785	tocopherol cyclase	δT3/(γT3+αT3)	S5_133501992	GBS	5	133,501,992	64	-16,503	9.72E-07	1.98E-03	0.40	0.14	0.46	248	0.10	0.19	0.03	-0.10	-0.26
Tocochromanol Pathway	GRMZM2G009785	tocopherol cyclase	δT3/αT3	S5_133501992	GBS	5	133,501,992	64	-16,503	3.55E-05	3.17E-02	0.41	0.14	0.46	246	0.11	0.18	0.06	-0.15	-0.32
Tocochromanol Pathway	GRMZM2G009785	tocopherol cyclase	δT3/γT3	S5_133501992	GBS	5	133,501,992	64	-16,503	4.89E-06	9.96E-03	0.40	0.14	0.46	249	0.13	0.20	-0.01	0.05	-0.18
Tocochromanol Pathway	GRMZM2G009785	tocopherol cyclase	δT3	S5_133501992	GBS	5	133,501,992	64	-16,503	1.36E-05	7.57E-03	0.40	0.14	0.46	247	0.12	0.19	0.06	-0.20	-0.25
Tocochromanol Pathway	GRMZM2G009785	tocopherol cyclase	δT3/(γT3+αT3)	PZB00969.1	4K	5	133,502,506	578	-15,989	8.27E-07	1.98E-03	0.41	0.14	0.46	248	0.10	0.19	0.03	-0.10	-0.26
Tocochromanol Pathway	GRMZM2G009785	tocopherol cyclase	δT3/(γT3+αT3)	ss196416168	55K	5	133,502,506	578	-15,989	1.30E-05	5.53E-03	0.40	0.13	0.46	248	0.10	0.17	0.03	-0.10	-0.26
Tocochromanol Pathway	GRMZM2G009785	tocopherol cyclase	δT3/αT3	PZB00969.1	4K	5	133,502,506	578	-15,989	3.64E-05	3.17E-02	0.41	0.14	0.46	246	0.11	0.18	0.06	-0.15	-0.32
Tocochromanol Pathway	GRMZM2G009785	tocopherol cyclase	δT3/γT3	PZB00969.1	4K	5	133,502,506	578	-15,989	3.32E-06	9.96E-03	0.41	0.14	0.46	249	0.13	0.21	-0.01	0.05	-0.18
Tocochromanol Pathway	GRMZM2G009785	tocopherol cyclase	δT3/γT3	ss196416168	55K	5	133,502,506	578	-15,989	4.29E-05	2.91E-02	0.40	0.13	0.46	249	0.13	0.19	-0.01	0.05	-0.18
Tocochromanol Pathway	GRMZM2G009785	tocopherol cyclase	δT3	PZB00969.1	4K	5	133,502,506	578	-15,989	1.17E-05	7.17E-03	0.41	0.14	0.46	247	0.12	0.19	0.06	-0.20	-0.25
Tocochromanol Pathway	GRMZM2G009785	tocopherol cyclase	δT3	ss196416168	55K	5	133,502,506	578	-15,989	1.29E-04	4.18E-02	0.40	0.13	0.46	247	0.12	0.17	0.05	-0.20	-0.22
Tocochromanol Pathway	GRMZM2G009785	tocopherol cyclase	δT3/(γT3+αT3)	ss196465634	55K	5	133,510,613	8,685	-7,882	7.54E-06	3.89E-03	0.41	0.14	0.46	248	0.10	0.17	-0.03	-0.10	0.36
Tocochromanol Pathway	GRMZM2G009785	tocopherol cyclase	δT3/γT3	ss196465634	55K	5	133,510,613	8,685	-7,882	1.92E-05	2.00E-02	0.41	0.14	0.46	249	0.13	0.19	0.01	0.05	0.22
Tocochromanol Pathway	GRMZM2G009785	tocopherol cyclase	δT3	ss196465634	55K	5	133,510,613	8,685	-7,882	8.23E-05	3.86E-02	0.41	0.14	0.46	247	0.12	0.18	-0.05	-0.20	0.29
Tocochromanol Pathway	GRMZM2G009785	tocopherol cyclase	δT3/(γT3+αT3)	PZB02491.1	4K	5	133,517,065	15,137	-1,430	6.69E-06	3.89E-03	0.40	0.14	0.46	248	0.10	0.18	-0.03	-0.10	0.36
Tocochromanol Pathway	GRMZM2G009785	tocopherol cyclase	δT3/γT3	PZB02491.1	4K	5	133,517,065	15,137	-1,430	7.44E-05	3.73E-02	0.40	0.14	0.46	249	0.13	0.18	0.01	0.05	0.22
Tocochromanol Pathway	GRMZM2G009785	tocopherol cyclase	δT3	PZB02491.1	4K	5	133,517,065	15,137	-1,430	4.86E-05	2.48E-02	0.40	0.14	0.46	247	0.12	0.18	-0.05	-0.20	0.29
Tocochromanol Pathway	GRMZM2G009785	tocopherol cyclase	δT3/(γT3+αT3)	S5_133618308	GBS	5	133,618,308	116,380	99,813	7.89E-06	3.89E-03	0.40	0.10	0.48	248	0.10	0.17	0.03	-0.10	-0.26
Tocochromanol Pathway	GRMZM2G009785	tocopherol cyclase	δT3/γT3	S5_133618308	GBS	5	133,618,308	116,380	99,813	2.29E-05	2.00E-02	0.40	0.10	0.48	249	0.13	0.19	-0.01	0.05	-0.18
Tocochromanol Pathway	GRMZM2G009785	tocopherol cyclase	δT3	S5_133618308	GBS	5	133,618,308	116,380	99,813	1.05E-04	4.02E-02	0.40	0.10	0.48	247	0.12	0.18	0.05	-0.20	-0.22
Tocochromanol Pathway	GRMZM2G009785	tocopherol cyclase	δT3/(γT3+αT3)	S5_133618309	GBS	5	133,618,309	116,381	99,814	7.89E-06	3.89E-03	0.40	0.10	0.48	248	0.10	0.17	0.03	-0.10	-0.26
Tocochromanol Pathway	GRMZM2G009785	tocopherol cyclase	δT3/γT3	S5_133618309	GBS	5	133,618,309	116,381	99,814	2.29E-05	2.00E-02	0.40	0.10	0.48	249	0.13	0.19	-0.01	0.05	-0.18
Tocochromanol Pathway	GRMZM2G009785	tocopherol cyclase	δT3	S5_133618309	GBS	5	133,618,309	116,381	99,814	1.05E-04	4.02E-02	0.40	0.10	0.48	247	0.12	0.18	0.05	-0.20	-0.22
Tocochromanol Pathway	GRMZM2G009785	tocopherol cyclase	δT3/(γT3+αT3)	S5_133618344	GBS	5	133,618,344	116,416	99,849	7.89E-06	3.89E-03	0.40	0.10	0.48	248	0.10	0.17	0.03	-0.10	-0.26
Tocochromanol Pathway	GRMZM2G009785	tocopherol cyclase	δT3/γT3	S5_133618344	GBS	5	133,618,344	116,416	99,849	2.29E-05	2.00E-02	0.40	0.10	0.48	249	0.13	0.19	-0.01	0.05	-0.18
Tocochromanol Pathway	GRMZM2G009785	tocopherol cyclase	δT3	S5_133618344	GBS	5	133,618,344	116,416	99,849	1.05E-04	4.02E-02	0.40	0.10	0.48	247	0.12	0.18	0.05	-0.20	-0.22
Tocochromanol Pathway	GRMZM2G009785	tocopherol cyclase	δT/γT	S5_133618788	GBS	5	133,618,788	116,860	100,293	2.56E-05	3.93E-02	0.49	0.11	0.42	249	0.13	0.19	0.02	0.30	0.07
Tocochromanol Pathway	GRMZM2G009785	tocopherol cyclase	δT3/(γT3+αT3)	S5_133618788	GBS	5	133,618,788	116,860	100,293	1.42E-04	3.35E-02	0.49	0.11	0.42	248	0.10	0.15	-0.03	-0.10	0.36
Tocochromanol Pathway	GRMZM2G009785	tocopherol cyclase	δT/γT	S5_133618810	GBS	5	133,618,810	116,882	100,315	2.56E-05	3.93E-02	0.49	0.11	0.42	249	0.13	0.19	0.02	0.30	0.07
Tocochromanol Pathway	GRMZM2G009785	tocopherol cyclase	δT3/(γT3+αT3)	S5_133618810	GBS	5	133,618,810	116,882	100,315	1.42E-04	3.35E-02	0.49	0.11	0.42	248	0.10	0.15	-0.03	-0.10	0.36
Tocochromanol Pathway	GRMZM2G009785	tocopherol cyclase	δT3/(γT3+αT3)	S5_133691297	GBS	5	133,691,297	189,369	172,802	5.02E-06	3.89E-03	0.37	0.10	0.46	248	0.10	0.18	0.03	-0.10	-0.26
Tocochromanol Pathway	GRMZM2G009785	tocopherol cyclase	δT3/γT3	S5_133691297	GBS	5	133,691,297	189,369	172,802	3.04E-05	2.32E-02	0.37	0.10	0.46	249	0.13	0.19	-0.01	0.05	-0.18
Tocochromanol Pathway	GRMZM2G009785	tocopherol cyclase	δT3	S5_133691297	GBS	5	133,691,297	189,369	172,802	1.59E-04	4.60E-02	0.37	0.10	0.46	247	0.12	0.17	0.05	-0.20	-0.22
Tocochromanol Pathway	GRMZM2G009785	tocopherol cyclase	δT3/(γT3+αT3)	ss196465642	55K	5	133,728,050	226,122	209,555	3.56E-05	1.04E-02	0.40	0.11	0.46	248	0.10	0.16	-0.03	-0.10	0.36
Tocochromanol Pathway	GRMZM2G009785	tocopherol cyclase	δT3	ss196465642	55K	5	133,728,050	226,122	209,555	1.85E-04	4.60E-02	0.40	0.11	0.46	247	0.12	0.17	-0.05	-0.20	0.29

Tocochromanol Pathway	GRMZM2G035213	γ-tocopherol methyltransferase	αT	S5_200117524	GBS	5	200,117,524	-249,505	-253,327	3.44E-05	8.42E-03	0.21	0.02	0.27	251	0.25	0.31	0.22	0.40	0.64
Tocochromanol Pathway	GRMZM2G035213	γ-tocopherol methyltransferase	αT	S5_200117526	GBS	5	200,117,526	-249,503	-253,325	1.93E-04	3.58E-02	0.22	0.07	0.27	251	0.25	0.30	0.20	0.40	0.58
Tocochromanol Pathway	GRMZM2G035213	γ-tocopherol methyltransferase	αT	S5_200117632	GBS	5	200,117,632	-249,397	-253,219	2.53E-04	3.97E-02	0.21	0.07	0.26	251	0.25	0.30	-0.19	0.40	-0.41
Tocochromanol Pathway	GRMZM2G035213	γ-tocopherol methyltransferase	αT	S5_200119597	GBS	5	200,119,597	-247,432	-251,254	1.71E-04	3.33E-02	0.33	0.18	0.36	251	0.25	0.30	0.17	0.40	0.48
Tocochromanol Pathway	GRMZM2G035213	γ-tocopherol methyltransferase	αT	S5_200119623	GBS	5	200,119,623	-247,406	-251,228	2.45E-04	3.97E-02	0.35	0.27	0.37	251	0.25	0.30	0.16	0.40	0.45
Tocochromanol Pathway	GRMZM2G035213	γ-tocopherol methyltransferase	γT/(γT+αT)	S5_200130689	GBS	5	200,130,689	-236,340	-240,162	4.45E-05	1.45E-02	0.08	0.33	0.08	251	0.29	0.34	0.12	2.00	0.06
Tocochromanol Pathway	GRMZM2G035213	γ-tocopherol methyltransferase	γT/(γT+αT)	S5_200292465	GBS	5	200,292,465	-74,564	-78,386	5.52E-05	1.69E-02	0.34	0.34	0.28	251	0.29	0.33	-0.06	2.00	-0.03
Tocochromanol Pathway	GRMZM2G035213	γ-tocopherol methyltransferase	αT/γT	S5_200293693	GBS	5	200,293,693	-73,336	-77,158	8.10E-06	2.90E-03	0.40	0.34	0.37	246	0.24	0.31	0.04	0.15	0.3
Tocochromanol Pathway	GRMZM2G035213	γ-tocopherol methyltransferase	αT	S5_200293693	GBS	5	200,293,693	-73,336	-77,158	3.33E-06	1.20E-03	0.41	0.34	0.37	251	0.25	0.32	0.20	0.40	0.58
Tocochromanol Pathway	GRMZM2G035213	γ-tocopherol methyltransferase	γT/(γT+αT)	S5_200293693	GBS	5	200,293,693	-73,336	-77,158	1.24E-06	6.88E-04	0.42	0.34	0.37	251	0.29	0.36	-0.07	2.00	-0.04
Tocochromanol Pathway	GRMZM2G035213	γ-tocopherol methyltransferase	δT/αT	S5_200293693	GBS	5	200,293,693	-73,336	-77,158	3.00E-05	1.07E-02	0.42	0.34	0.37	246	0.28	0.33	-0.31	Log	-0.72
Tocochromanol Pathway	GRMZM2G035213	γ-tocopherol methyltransferase	αT	ss196468356	55K	5	200,300,836	-66,193	-70,015	9.29E-05	2.11E-02	0.43	0.20	0.49	251	0.25	0.30	-0.17	0.40	-0.37
Tocochromanol Pathway	GRMZM2G035213	γ-tocopherol methyltransferase	γT/(γT+αT)	ss196468356	55K	5	200,300,836	-66,193	-70,015	2.50E-04	5.09E-02	0.43	0.20	0.49	251	0.29	0.33	0.06	2.00	0.03
Tocochromanol Pathway	GRMZM2G035213	γ-tocopherol methyltransferase	δT/αT	ss196468356	55K	5	200,300,836	-66,193	-70,015	6.33E-06	3.21E-03	0.43	0.20	0.49	246	0.28	0.34	0.33	Log	1.06
Tocochromanol Pathway	GRMZM2G035213	γ-tocopherol methyltransferase	αT/γT	S5_200318615	GBS	5	200,318,615	-48,414	-52,236	2.50E-06	1.17E-03	0.22	0.27	0.44	246	0.24	0.32	0.05	0.15	0.38
Tocochromanol Pathway	GRMZM2G035213	γ-tocopherol methyltransferase	αT	S5_200318615	GBS	5	200,318,615	-48,414	-52,236	1.05E-06	4.57E-04	0.22	0.27	0.44	251	0.25	0.33	0.25	0.40	0.75
Tocochromanol Pathway	GRMZM2G035213	γ-tocopherol methyltransferase	γT/(γT+αT)	S5_200318615	GBS	5	200,318,615	-48,414	-52,236	2.03E-05	8.29E-03	0.22	0.27	0.44	251	0.29	0.34	-0.08	2.00	-0.04
Tocochromanol Pathway	GRMZM2G035213	γ-tocopherol methyltransferase	δT/αT	S5_200318615	GBS	5	200,318,615	-48,414	-52,236	8.89E-05	2.70E-02	0.21	0.27	0.44	246	0.28	0.33	-0.35	Log	-0.8
Tocochromanol Pathway	GRMZM2G035213	γ-tocopherol methyltransferase	αT/γT	PZB02283.1	4K	5	200,367,532	503	-3,319	9.21E-13	1.87E-09	0.21	0.14	0.22	246	0.24	0.42	0.08	0.15	0.67
Tocochromanol Pathway	GRMZM2G035213	γ-tocopherol methyltransferase	αT/γT	ss196416269	55K	5	200,367,532	503	-3,319	9.21E-13	1.87E-09	0.21	0.13	0.22	246	0.24	0.42	0.08	0.15	0.67
Tocochromanol Pathway	GRMZM2G035213	γ-tocopherol methyltransferase	αT	PZB02283.1	4K	5	200,367,532	503	-3,319	7.36E-14	1.50E-10	0.20	0.14	0.22	251	0.25	0.44	0.40	0.40	1.32
Tocochromanol Pathway	GRMZM2G035213	γ-tocopherol methyltransferase	αT	ss196416269	55K	5	200,367,532	503	-3,319	7.36E-14	1.50E-10	0.20	0.13	0.22	251	0.25	0.44	0.40	0.40	1.32
Tocochromanol Pathway	GRMZM2G035213	γ-tocopherol methyltransferase	γT/(γT+αT)	PZB02283.1	4K	5	200,367,532	503	-3,319	4.82E-12	9.83E-09	0.20	0.14	0.22	251	0.29	0.44	-0.13	2.00	-0.07
Tocochromanol Pathway	GRMZM2G035213	γ-tocopherol methyltransferase	γT/(γT+αT)	ss196416269	55K	5	200,367,532	503	-3,319	4.82E-12	9.83E-09	0.20	0.13	0.22	251	0.29	0.44	-0.13	2.00	-0.07
Tocochromanol Pathway	GRMZM2G035213	γ-tocopherol methyltransferase	δT/αT	PZB02283.1	4K	5	200,367,532	503	-3,319	1.88E-11	3.80E-08	0.20	0.14	0.22	246	0.28	0.42	-0.63	Log	-1.27
Tocochromanol Pathway	GRMZM2G035213	γ-tocopherol methyltransferase	δT/αT	ss196416269	55K	5	200,367,532	503	-3,319	1.88E-11	3.80E-08	0.20	0.13	0.22	246	0.28	0.42	-0.63	Log	-1.27
Tocochromanol Pathway	GRMZM2G035213	γ-tocopherol methyltransferase	αT/γT	ss196468362	55K	5	200,369,124	2,095	-1,727	9.21E-13	1.87E-09	0.21	0.13	0.22	246	0.24	0.42	0.08	0.15	0.67
Tocochromanol Pathway	GRMZM2G035213	γ-tocopherol methyltransferase	αT	ss196468362	55K	5	200,369,124	2,095	-1,727	7.36E-14	1.50E-10	0.20	0.13	0.22	251	0.25	0.44	0.40	0.40	1.32
Tocochromanol Pathway	GRMZM2G035213	γ-tocopherol methyltransferase	γT/(γT+αT)	ss196468362	55K	5	200,369,124	2,095	-1,727	4.82E-12	9.83E-09	0.20	0.13	0.22	251	0.29	0.44	-0.13	2.00	-0.07
Tocochromanol Pathway	GRMZM2G035213	γ-tocopherol methyltransferase	δT/αT	ss196468362	55K	5	200,369,124	2,095	-1,727	1.88E-11	3.80E-08	0.20	0.13	0.22	246	0.28	0.42	-0.63	Log	-1.27
Tocochromanol Pathway	GRMZM2G035213	γ-tocopherol methyltransferase	αT/γT	S5_200369481	GBS	5	200,369,481	2,452	-1,370	7.24E-07	4.01E-04	0.10	0.36	0.09	246	0.24	0.32	0.07	0.15	0.57
Tocochromanol Pathway	GRMZM2G035213	γ-tocopherol methyltransferase	αT	S5_200369481	GBS	5	200,369,481	2,452	-1,370	1.97E-08	2.00E-05	0.11	0.36	0.09	251	0.25	0.35	0.38	0.40	1.24
Tocochromanol Pathway	GRMZM2G035213	γ-tocopherol methyltransferase	γT/(γT+αT)	S5_200369481	GBS	5	200,369,481	2,452	-1,370	6.15E-09	7.53E-06	0.12	0.36	0.09	251	0.29	0.39	-0.14	2.00	-0.07
Tocochromanol Pathway	GRMZM2G035213	γ-tocopherol methyltransferase	δT/αT	S5_200369481	GBS	5	200,369,481	2,452	-1,370	7.21E-07	5.35E-04	0.12	0.36	0.09	246	0.28	0.35	-0.56	Log	-1.17
Tocochromanol Pathway	GRMZM2G035213	γ-tocopherol methyltransferase	αT/γT	S5_200369508	GBS	5	200,369,508	2,479	-1,343	9.69E-07	4.92E-04	0.18	0.11	0.22	246	0.24	0.32	0.06	0.15	0.47
Tocochromanol Pathway	GRMZM2G035213	γ-tocopherol methyltransferase	αT3/γT3	S5_200369508	GBS	5	200,369,508	2,479	-1,343	1.16E-05	2.36E-02	0.17	0.11	0.22	246	0.13	0.20	-0.05	-0.15	0.41
Tocochromanol Pathway	GRMZM2G035213	γ-tocopherol methyltransferase	αT	S5_200369508	GBS	5	200,369,508	2,479	-1,343	6.85E-07	3.22E-04	0.18	0.11	0.22	251	0.25	0.33	0.28	0.40	0.85

Tocochromanol Pathway	GRMZM2G035213	γ-tocopherol methyltransferase	γT/(γT+αT)	S5_200369508	GBS	5	200,369,508	2,479	-1,343	6.57E-07	4.02E-04	0.18	0.11	0.22	251	0.29	0.36	-0.10	2.00	-0.05
Tocochromanol Pathway	GRMZM2G035213	γ-tocopherol methyltransferase	δT/αT	S5_200369508	GBS	5	200,369,508	2,479	-1,343	8.30E-06	3.60E-03	0.17	0.11	0.22	246	0.28	0.34	-0.45	Log	-0.99
Tocochromanol Pathway	GRMZM2G035213	γ-tocopherol methyltransferase	αT/γT	S5_200369534	GBS	5	200,369,534	2,505	-1,317	5.45E-06	2.37E-03	0.09	0.00	0.12	246	0.24	0.31	-0.07	0.15	-0.38
Tocochromanol Pathway	GRMZM2G035213	γ-tocopherol methyltransferase	αT	S5_200369534	GBS	5	200,369,534	2,505	-1,317	2.59E-06	9.88E-04	0.09	0.00	0.12	251	0.25	0.32	-0.34	0.40	-0.65
Tocochromanol Pathway	GRMZM2G035213	γ-tocopherol methyltransferase	γT/(γT+αT)	S5_200369534	GBS	5	200,369,534	2,505	-1,317	6.83E-05	1.99E-02	0.09	0.00	0.12	251	0.29	0.33	0.10	2.00	0.05
Tocochromanol Pathway	GRMZM2G035213	γ-tocopherol methyltransferase	δT/αT	S5_200369534	GBS	5	200,369,534	2,505	-1,317	6.11E-05	1.96E-02	0.08	0.00	0.12	246	0.28	0.33	0.49	Log	1.72
Tocochromanol Pathway	GRMZM2G035213	γ-tocopherol methyltransferase	αT/γT	S5_200369625	GBS	5	200,369,625	2,596	-1,226	2.86E-05	8.28E-03	0.30	0.48	0.49	246	0.24	0.30	-0.04	0.15	-0.24
Tocochromanol Pathway	GRMZM2G035213	γ-tocopherol methyltransferase	αT	S5_200369625	GBS	5	200,369,625	2,596	-1,226	5.00E-08	3.40E-05	0.30	0.48	0.49	251	0.25	0.35	-0.24	0.40	-0.5
Tocochromanol Pathway	GRMZM2G035213	γ-tocopherol methyltransferase	γT/(γT+αT)	S5_200369625	GBS	5	200,369,625	2,596	-1,226	8.13E-06	3.83E-03	0.29	0.48	0.49	251	0.29	0.35	0.07	2.00	0.03
Tocochromanol Pathway	GRMZM2G035213	γ-tocopherol methyltransferase	δT/αT	S5_200369625	GBS	5	200,369,625	2,596	-1,226	2.02E-06	1.23E-03	0.30	0.48	0.49	246	0.28	0.35	0.35	Log	1.14
Tocochromanol Pathway	GRMZM2G035213	γ-tocopherol methyltransferase	αT/γT	S5_200369644	GBS	5	200,369,644	2,615	-1,207	9.97E-06	3.20E-03	0.13	0.12	0.22	246	0.24	0.31	-0.05	0.15	-0.29
Tocochromanol Pathway	GRMZM2G035213	γ-tocopherol methyltransferase	αT	S5_200369644	GBS	5	200,369,644	2,615	-1,207	9.36E-06	2.60E-03	0.12	0.12	0.22	251	0.25	0.32	-0.26	0.40	-0.53
Tocochromanol Pathway	GRMZM2G035213	γ-tocopherol methyltransferase	αT/γT	S5_200369665	GBS	5	200,369,665	2,636	-1,186	9.97E-06	3.20E-03	0.13	0.12	0.22	246	0.24	0.31	0.05	0.15	0.38
Tocochromanol Pathway	GRMZM2G035213	γ-tocopherol methyltransferase	αT	S5_200369665	GBS	5	200,369,665	2,636	-1,186	9.36E-06	2.60E-03	0.12	0.12	0.22	251	0.25	0.32	0.26	0.40	0.78
Tocochromanol Pathway	GRMZM2G035213	γ-tocopherol methyltransferase	αT/γT	S5_200369667	GBS	5	200,369,667	2,638	-1,184	1.70E-07	1.21E-04	0.11	0.00	0.20	246	0.24	0.33	-0.07	0.15	-0.38
Tocochromanol Pathway	GRMZM2G035213	γ-tocopherol methyltransferase	αT	S5_200369667	GBS	5	200,369,667	2,638	-1,184	1.67E-07	9.31E-05	0.10	0.00	0.20	251	0.25	0.34	-0.33	0.40	-0.63
Tocochromanol Pathway	GRMZM2G035213	γ-tocopherol methyltransferase	γT/(γT+αT)	S5_200369667	GBS	5	200,369,667	2,638	-1,184	1.48E-05	6.49E-03	0.10	0.00	0.20	251	0.29	0.34	0.09	2.00	0.04
Tocochromanol Pathway	GRMZM2G035213	γ-tocopherol methyltransferase	δT/αT	S5_200369667	GBS	5	200,369,667	2,638	-1,184	7.09E-06	3.32E-03	0.10	0.00	0.20	246	0.28	0.34	0.48	Log	1.67
Tocochromanol Pathway	GRMZM2G035213	γ-tocopherol methyltransferase	αT	ss196468364	55K	5	200,370,065	3,036	-786	3.24E-05	8.27E-03	0.35	0.39	0.31	251	0.25	0.31	0.18	0.40	0.51
Tocochromanol Pathway	GRMZM2G035213	γ-tocopherol methyltransferase	αT/γT	PZB02424.2	4K	5	200,370,309	3,280	-542	2.60E-04	6.10E-02	0.17	0.09	0.19	246	0.24	0.29	-0.04	0.15	-0.24
Tocochromanol Pathway	GRMZM2G035213	γ-tocopherol methyltransferase	αT	PZB02424.2	4K	5	200,370,309	3,280	-542	4.09E-07	2.09E-04	0.17	0.09	0.19	251	0.25	0.33	-0.26	0.40	-0.53
Tocochromanol Pathway	GRMZM2G035213	γ-tocopherol methyltransferase	γT/(γT+αT)	PZB02424.2	4K	5	200,370,309	3,280	-542	7.64E-04	1.04E-01	0.17	0.09	0.19	251	0.29	0.32	0.06	2.00	0.03
Tocochromanol Pathway	GRMZM2G035213	γ-tocopherol methyltransferase	δT/αT	PZB02424.2	4K	5	200,370,309	3,280	-542	5.35E-03	3.17E-01	0.16	0.09	0.19	246	0.28	0.30	0.25	Log	0.77
Tocochromanol Pathway	GRMZM2G035213	γ-tocopherol methyltransferase	αT/γT	ss196468368	55K	5	200,371,057	4,028	206	7.37E-06	2.90E-03	0.14	0.13	0.15	246	0.24	0.31	-0.05	0.15	-0.29
Tocochromanol Pathway	GRMZM2G035213	γ-tocopherol methyltransferase	αT	ss196468368	55K	5	200,371,057	4,028	206	6.73E-06	2.17E-03	0.14	0.13	0.15	251	0.25	0.32	-0.27	0.40	-0.54
Tocochromanol Pathway	GRMZM2G035213	γ-tocopherol methyltransferase	γT/(γT+αT)	ss196468368	55K	5	200,371,057	4,028	206	2.74E-05	1.05E-02	0.14	0.13	0.15	251	0.29	0.34	0.09	2.00	0.04
Tocochromanol Pathway	GRMZM2G035213	γ-tocopherol methyltransferase	δT/αT	ss196468368	55K	5	200,371,057	4,028	206	5.98E-05	1.96E-02	0.13	0.13	0.15	246	0.28	0.33	0.43	Log	1.46
Tocochromanol Pathway	GRMZM2G035213	γ-tocopherol methyltransferase	γT/(γT+αT)	S5_200382117	GBS	5	200,382,117	15,088	11,266	1.43E-04	3.25E-02	0.06	0.07	0.07	251	0.29	0.33	-0.11	2.00	-0.06
Tocochromanol Pathway	GRMZM2G035213	γ-tocopherol methyltransferase	γT/(γT+αT)	S5_200382141	GBS	5	200,382,141	15,112	11,290	1.43E-04	3.25E-02	0.06	0.07	0.07	251	0.29	0.33	-0.11	2.00	-0.06
Tocochromanol Pathway	GRMZM2G035213	γ-tocopherol methyltransferase	αT/γT	S5_200382168	GBS	5	200,382,168	15,139	11,317	3.08E-08	3.13E-05	0.39	0.38	0.40	246	0.24	0.34	-0.05	0.15	-0.29
Tocochromanol Pathway	GRMZM2G035213	γ-tocopherol methyltransferase	αT	S5_200382168	GBS	5	200,382,168	15,139	11,317	3.01E-08	2.30E-05	0.39	0.38	0.40	251	0.25	0.35	-0.25	0.40	-0.51
Tocochromanol Pathway	GRMZM2G035213	γ-tocopherol methyltransferase	γT/(γT+αT)	S5_200382168	GBS	5	200,382,168	15,139	11,317	9.59E-09	8.38E-06	0.39	0.38	0.40	251	0.29	0.39	0.09	2.00	0.04
Tocochromanol Pathway	GRMZM2G035213	γ-tocopherol methyltransferase	δT/αT	S5_200382168	GBS	5	200,382,168	15,139	11,317	6.31E-08	6.39E-05	0.41	0.38	0.40	246	0.28	0.37	0.41	Log	1.38
Tocochromanol Pathway	GRMZM2G035213	γ-tocopherol methyltransferase	αT/γT	S5_200384686	GBS	5	200,384,686	17,657	13,835	1.72E-04	4.57E-02	0.13	0.31	0.21	246	0.24	0.29	0.04	0.15	0.3
Tocochromanol Pathway	GRMZM2G035213	γ-tocopherol methyltransferase	αT	S5_200384686	GBS	5	200,384,686	17,657	13,835	2.77E-05	7.37E-03	0.14	0.31	0.21	251	0.25	0.31	0.24	0.40	0.71
Tocochromanol Pathway	GRMZM2G035213	γ-tocopherol methyltransferase	γT/(γT+αT)	S5_200384686	GBS	5	200,384,686	17,657	13,835	1.07E-04	2.85E-02	0.14	0.31	0.21	251	0.29	0.33	-0.08	2.00	-0.04
Tocochromanol Pathway	GRMZM2G035213	γ-tocopherol methyltransferase	αT	S5_200435003	GBS	5	200,435,003	67,974	64,152	1.74E-04	3.33E-02	0.11	0.12	0.13	251	0.25	0.30	0.25	0.40	0.75

Tocochromanol Pathway	GRMZM2G035213	γ-tocopherol methyltransferase	αT	S5_200435045	GBS	5	200,435,045	68,016	64,194	1.40E-04	2.85E-02	0.12	0.12	0.13	251	0.25	0.30	-0.24	0.40	-0.5
Tocochromanol Pathway	GRMZM2G035213	γ-tocopherol methyltransferase	αT/γT	S5_200435108	GBS	5	200,435,108	68,079	64,257	3.28E-10	5.00E-07	0.25	0.44	0.21	246	0.24	0.38	-0.06	0.15	-0.34
Tocochromanol Pathway	GRMZM2G035213	γ-tocopherol methyltransferase	αT3	S5_200435108	GBS	5	200,435,108	68,079	64,257	2.65E-05	3.57E-02	0.26	0.44	0.21	248	0.20	0.26	-0.01	0.05	-0.18
Tocochromanol Pathway	GRMZM2G035213	γ-tocopherol methyltransferase	αT	S5_200435108	GBS	5	200,435,108	68,079	64,257	4.39E-10	6.71E-07	0.26	0.44	0.21	251	0.25	0.38	-0.30	0.40	-0.59
Tocochromanol Pathway	GRMZM2G035213	γ-tocopherol methyltransferase	γT/(γT+αT)	S5_200435108	GBS	5	200,435,108	68,079	64,257	1.13E-10	1.73E-07	0.27	0.44	0.21	251	0.29	0.42	0.11	2.00	0.05
Tocochromanol Pathway	GRMZM2G035213	γ-tocopherol methyltransferase	δT/αT	S5_200435108	GBS	5	200,435,108	68,079	64,257	6.37E-09	9.68E-06	0.27	0.44	0.21	246	0.28	0.39	0.47	Log	1.63
Tocochromanol Pathway	GRMZM2G035213	γ-tocopherol methyltransferase	αT/γT	S5_200435117	GBS	5	200,435,117	68,088	64,266	1.79E-07	1.21E-04	0.27	0.19	0.28	246	0.24	0.33	0.05	0.15	0.38
Tocochromanol Pathway	GRMZM2G035213	γ-tocopherol methyltransferase	αT3/γT3	S5_200435117	GBS	5	200,435,117	68,088	64,266	7.91E-06	2.36E-02	0.26	0.19	0.28	246	0.13	0.21	-0.04	-0.15	0.31
Tocochromanol Pathway	GRMZM2G035213	γ-tocopherol methyltransferase	αT3	S5_200435117	GBS	5	200,435,117	68,088	64,266	2.50E-06	1.53E-02	0.26	0.19	0.28	248	0.20	0.27	0.01	0.05	0.22
Tocochromanol Pathway	GRMZM2G035213	γ-tocopherol methyltransferase	αT	S5_200435117	GBS	5	200,435,117	68,088	64,266	1.21E-07	7.41E-05	0.26	0.19	0.28	251	0.25	0.34	0.25	0.40	0.75
Tocochromanol Pathway	GRMZM2G035213	γ-tocopherol methyltransferase	γT/(γT+αT)	S5_200435117	GBS	5	200,435,117	68,088	64,266	1.66E-07	1.13E-04	0.26	0.19	0.28	251	0.29	0.37	-0.09	2.00	-0.05
Tocochromanol Pathway	GRMZM2G035213	γ-tocopherol methyltransferase	δT/αT	S5_200435117	GBS	5	200,435,117	68,088	64,266	7.93E-07	5.35E-04	0.25	0.19	0.28	246	0.28	0.35	-0.41	Log	-0.91
Tocochromanol Pathway	GRMZM2G035213	γ-tocopherol methyltransferase	αT/γT	ss196468352	55K	5	200,435,300	68,271	64,449	2.68E-08	3.13E-05	0.39	0.39	0.37	246	0.24	0.35	-0.05	0.15	-0.29
Tocochromanol Pathway	GRMZM2G035213	γ-tocopherol methyltransferase	αT3/γT3	ss196468352	55K	5	200,435,300	68,271	64,449	5.91E-06	2.36E-02	0.40	0.39	0.37	246	0.13	0.21	0.04	-0.15	-0.23
Tocochromanol Pathway	GRMZM2G035213	γ-tocopherol methyltransferase	αT	ss196468352	55K	5	200,435,300	68,271	64,449	8.32E-10	1.02E-06	0.40	0.39	0.37	251	0.25	0.38	-0.27	0.40	-0.54
Tocochromanol Pathway	GRMZM2G035213	γ-tocopherol methyltransferase	γT/(γT+αT)	ss196468352	55K	5	200,435,300	68,271	64,449	7.67E-09	7.82E-06	0.40	0.39	0.37	251	0.29	0.39	0.09	2.00	0.04
Tocochromanol Pathway	GRMZM2G035213	γ-tocopherol methyltransferase	γT3/(γT3+αT3)	ss196468352	55K	5	200,435,300	68,271	64,449	3.05E-05	4.66E-02	0.40	0.39	0.37	251	0.16	0.22	0.06	1.35	0.04
Tocochromanol Pathway	GRMZM2G035213	γ-tocopherol methyltransferase	δT/αT	ss196468352	55K	5	200,435,300	68,271	64,449	2.87E-08	3.48E-05	0.41	0.39	0.37	246	0.28	0.38	0.42	Log	1.42
Tocochromanol Pathway	GRMZM2G035213	γ-tocopherol methyltransferase	αT/γT	S5_200437468	GBS	5	200,437,468	70,439	66,617	9.02E-08	7.84E-05	0.27	0.45	0.24	246	0.24	0.34	-0.05	0.15	-0.29
Tocochromanol Pathway	GRMZM2G035213	γ-tocopherol methyltransferase	αT	S5_200437468	GBS	5	200,437,468	70,439	66,617	2.29E-08	2.00E-05	0.28	0.45	0.24	251	0.25	0.35	-0.26	0.40	-0.53
Tocochromanol Pathway	GRMZM2G035213	γ-tocopherol methyltransferase	γT/(γT+αT)	S5_200437468	GBS	5	200,437,468	70,439	66,617	4.13E-08	3.16E-05	0.28	0.45	0.24	251	0.29	0.38	0.09	2.00	0.04
Tocochromanol Pathway	GRMZM2G035213	γ-tocopherol methyltransferase	δT/αT	S5_200437468	GBS	5	200,437,468	70,439	66,617	3.72E-07	3.23E-04	0.29	0.45	0.24	246	0.28	0.36	0.40	Log	1.34
Tocochromanol Pathway	GRMZM2G035213	γ-tocopherol methyltransferase	αT/γT	ss196517251	55K	5	200,437,606	70,577	66,755	4.45E-07	2.71E-04	0.45	0.23	0.40	246	0.24	0.33	-0.04	0.15	-0.24
Tocochromanol Pathway	GRMZM2G035213	γ-tocopherol methyltransferase	αT	ss196517251	55K	5	200,437,606	70,577	66,755	1.38E-06	5.62E-04	0.46	0.23	0.40	251	0.25	0.33	-0.21	0.40	-0.45
Tocochromanol Pathway	GRMZM2G035213	γ-tocopherol methyltransferase	γT/(γT+αT)	ss196517251	55K	5	200,437,606	70,577	66,755	2.99E-06	1.53E-03	0.46	0.23	0.40	251	0.29	0.35	0.07	2.00	0.03
Tocochromanol Pathway	GRMZM2G035213	γ-tocopherol methyltransferase	δT/αT	ss196517251	55K	5	200,437,606	70,577	66,755	5.53E-06	3.05E-03	0.47	0.23	0.40	246	0.28	0.34	0.35	Log	1.14
Tocochromanol Pathway	GRMZM2G035213	γ-tocopherol methyltransferase	αT/γT	S5_200438801	GBS	5	200,438,801	71,772	67,950	7.67E-06	2.90E-03	0.10	0.06	0.13	246	0.24	0.31	0.06	0.15	0.47
Tocochromanol Pathway	GRMZM2G035213	γ-tocopherol methyltransferase	αT	S5_200438801	GBS	5	200,438,801	71,772	67,950	4.30E-06	1.46E-03	0.10	0.06	0.13	251	0.25	0.32	0.31	0.40	0.96
Tocochromanol Pathway	GRMZM2G035213	γ-tocopherol methyltransferase	γT/(γT+αT)	S5_200438801	GBS	5	200,438,801	71,772	67,950	2.09E-04	4.58E-02	0.10	0.06	0.13	251	0.29	0.33	-0.09	2.00	-0.05
Tocochromanol Pathway	GRMZM2G035213	γ-tocopherol methyltransferase	αT	ss196512031	55K	5	200,491,310	124,281	120,459	1.09E-04	2.38E-02	0.40	0.35	0.36	251	0.25	0.30	0.17	0.40	0.48
Tocochromanol Pathway	GRMZM2G035213	γ-tocopherol methyltransferase	γT/(γT+αT)	ss196512031	55K	5	200,491,310	124,281	120,459	1.07E-04	2.85E-02	0.40	0.35	0.36	251	0.29	0.33	-0.06	2.00	-0.03
Tocochromanol Pathway	GRMZM2G035213	γ-tocopherol methyltransferase	δT/αT	ss196512031	55K	5	200,491,310	124,281	120,459	1.11E-05	4.51E-03	0.40	0.35	0.36	246	0.28	0.34	-0.33	Log	-0.76
Tocochromanol Pathway	GRMZM2G035213	γ-tocopherol methyltransferase	αT	ss196468372	55K	5	200,598,221	231,192	227,370	2.53E-04	3.97E-02	0.25	0.18	0.27	251	0.25	0.30	-0.19	0.40	-0.41
Tocochromanol Pathway	GRMZM2G173358	homogentisic acid geranylgeranyl transferase	Total Tocotrienols	S9_92346116	GBS	9	92,346,116	-137,433	-141,152	6.37E-06	3.76E-02	0.41	0.43	0.44	250	0.20	0.27	0.03	0.15	0.22
Tocochromanol Pathway	GRMZM2G173358	homogentisic acid geranylgeranyl transferase	γT3	S9_92346116	GBS	9	92,346,116	-137,433	-141,152	3.10E-06	4.76E-03	0.41	0.43	0.44	250	0.20	0.27	0.03	0.10	0.34
Tocochromanol Pathway	GRMZM2G173358	homogentisic acid geranylgeranyl transferase	Total Tocopherols/Total Tocotrienols	S9_92548696	GBS	9	92,548,696	65,147	61,428	1.28E-05	3.91E-02	0.39	0.38	0.40	247	0.19	0.26	-0.01	0.05	-0.20
Tocochromanol	GRMZM2G173358	homogentisic acid	αT3	S9_92553908	GBS	9	92,553,908	70,359	66,640	2.92E-05	3.57E-02	0.43	0.05	0.38	248	0.20	0.26	0.01	0.05	0.22

Pathway		geranylgeranyl transferase																		
Tocochromanol Pathway	GRMZM2G173358	homogentisic acid geranylgeranyl transferase	Total Tocopherols/Total Tocotrienols	S9_92554465	GBS	9	92,554,465	70,916	67,197	2.45E-06	1.49E-02	0.26	0.00	0.32	247	0.19	0.27	-0.01	0.05	-0.24
Tocochromanol Pathway	GRMZM2G173358	homogentisic acid geranylgeranyl transferase	Total Tocotrienols	S9_92718671	GBS	9	92,718,671	235,122	231,403	5.43E-05	4.69E-02	0.08	0.00	0.13	250	0.20	0.26	-0.05	0.15	-0.29
Tocochromanol Pathway	GRMZM2G173358	homogentisic acid geranylgeranyl transferase	γT3/(γT3+αT3)	S9_92718671	GBS	9	92,718,671	235,122	231,403	1.45E-05	2.96E-02	0.08	0.00	0.13	251	0.16	0.23	-0.10	1.35	-0.08
Tocochromanol Pathway	GRMZM2G173358	homogentisic acid geranylgeranyl transferase	γT3	S9_92718671	GBS	9	92,718,671	235,122	231,403	4.48E-07	9.16E-04	0.08	0.00	0.13	250	0.20	0.28	-0.05	0.10	-0.4
Tocochromanol Pathway	GRMZM2G173358	homogentisic acid geranylgeranyl transferase	Total Tocotrienols	S9_92718674	GBS	9	92,718,674	235,125	231,406	5.43E-05	4.69E-02	0.08	0.00	0.13	250	0.20	0.26	-0.05	0.15	-0.29
Tocochromanol Pathway	GRMZM2G173358	homogentisic acid geranylgeranyl transferase	γT3/(γT3+αT3)	S9_92718674	GBS	9	92,718,674	235,125	231,406	1.45E-05	2.96E-02	0.08	0.00	0.13	251	0.16	0.23	-0.10	1.35	-0.08
Tocochromanol Pathway	GRMZM2G173358	homogentisic acid geranylgeranyl transferase	γT3	S9_92718674	GBS	9	92,718,674	235,125	231,406	4.48E-07	9.16E-04	0.08	0.00	0.13	250	0.20	0.28	-0.05	0.10	-0.4
Tocochromanol Pathway	GRMZM2G173358	homogentisic acid geranylgeranyl transferase	Total Tocotrienols	S9_92718709	GBS	9	92,718,709	235,160	231,441	5.43E-05	4.69E-02	0.08	0.00	0.13	250	0.20	0.26	-0.05	0.15	-0.29
Tocochromanol Pathway	GRMZM2G173358	homogentisic acid geranylgeranyl transferase	γT3/(γT3+αT3)	S9_92718709	GBS	9	92,718,709	235,160	231,441	1.45E-05	2.96E-02	0.08	0.00	0.13	251	0.16	0.23	-0.10	1.35	-0.08
Tocochromanol Pathway	GRMZM2G173358	homogentisic acid geranylgeranyl transferase	γT3	S9_92718709	GBS	9	92,718,709	235,160	231,441	4.48E-07	9.16E-04	0.08	0.00	0.13	250	0.20	0.28	-0.05	0.10	-0.4

Table S7b Statistically significant results from the candidate gene association study of 20 tocochromanol grain traits with the three SNPs within ZmVTE4 identified in the multi-locus mixed-model (MLMM) analysis included as covariates. SNPs that were significantly associated with the indicated trait at 5% FDR are shown.

a priori candidate gene pathway	RefGen_v2 Gene ID	RefGen_v2 Annotated Gene Function	Trait	SNP ID	SNP Source	Chr	Position	Distance from Gene ORF Start	Distance from Gene ORF Finish	*P*-value	FDR Adjusted *P*-value	Minor Allele Frequency (MAF)	MAF Tropical (18% of 252 Lines)	MAF Temperate (82% of 252 Lines)	Sample Size	R^2_{LR} from Model without SNP	R^2_{LR} from Model with SNP	Effect Size	Lambda from Box-Cox Procedure	Back-Transformed Effect Estimates
Aromatic Head Group	GRMZM2G437912	prephenate dehydratase	Total Tocotrienols	S2_59013838	GBS	2	59,013,838	-23,405	-25,340	2.16E-05	4.40E-02	0.06	0.40	0.09	250	0.20	0.27	0.06	0.15	0.47
Aromatic Head Group	GRMZM2G437912	prephenate dehydratase	Total Tocotrienols	S2_59013840	GBS	2	59,013,840	-23,403	-25,338	2.16E-05	4.40E-02	0.06	0.40	0.09	250	0.20	0.27	-0.06	0.15	-0.34
Aromatic Head Group	GRMZM2G070218	shikimate kinase	δT/αT	S5_207660287	GBS	5	207,660,287	-44,669	-47,594	7.82E-06	4.75E-02	0.13	0.15	0.16	246	0.56	0.60	-0.36	0.00	-0.82
Prenyl Group Synthesis	GRMZM2G027059	4-hydroxy-3-methylbut-2-enyldiphosphate reductase	δT3	ss196519818	55K	1	272,871,696	-65,140	-68,806	1.49E-04	4.80E-02	0.32	0.09	0.38	247	0.12	0.18	-0.06	-0.20	0.36
Prenyl Group Synthesis	GRMZM2G027059	4-hydroxy-3-methylbut-2-enyldiphosphate reductase	δT3	ss196519822	55K	1	272,874,639	-62,197	-65,863	1.39E-04	4.72E-02	0.33	0.09	0.38	247	0.12	0.18	-0.06	-0.20	0.36
Prenyl Group Synthesis	GRMZM2G172032	2-C-methyl-D-erythritol 4-phosphate cytidyltransferase	δT3/(γT3+αT3)	S8_164626861	GBS	8	164,626,861	-122,078	-125,510	4.09E-05	1.19E-02	0.14	0.50	0.10	248	0.10	0.17	-0.04	-0.10	0.5
Tocochromanol Pathway	GRMZM2G009785	tocopherol cyclase	δT3/(γT3+αT3)	S5_133331094	GBS	5	133,331,094	-170,834	-187,401	3.88E-05	1.19E-02	0.41	0.47	0.41	248	0.10	0.17	0.03	-0.10	-0.26
Tocochromanol Pathway	GRMZM2G009785	tocopherol cyclase	δT3/αT3	S5_133331094	GBS	5	133,331,094	-170,834	-187,401	6.18E-05	3.42E-02	0.41	0.47	0.41	246	0.14	0.20	0.05	-0.15	-0.28
Tocochromanol Pathway	GRMZM2G009785	tocopherol cyclase	δT3	S5_133331094	GBS	5	133,331,094	-170,834	-187,401	5.02E-06	3.83E-03	0.41	0.47	0.41	247	0.12	0.20	0.06	-0.20	-0.25
Tocochromanol Pathway	GRMZM2G009785	tocopherol cyclase	δT3/(γT3+αT3)	S5_133331096	GBS	5	133,331,096	-170,832	-187,399	1.60E-05	6.10E-03	0.40	0.44	0.41	248	0.10	0.17	-0.03	-0.10	0.36
Tocochromanol Pathway	GRMZM2G009785	tocopherol cyclase	δT3/αT3	S5_133331096	GBS	5	133,331,096	-170,832	-187,399	2.10E-05	2.76E-02	0.40	0.44	0.41	246	0.14	0.20	-0.05	-0.15	0.41
Tocochromanol Pathway	GRMZM2G009785	tocopherol cyclase	δT3	S5_133331096	GBS	5	133,331,096	-170,832	-187,399	1.15E-06	1.75E-03	0.40	0.44	0.41	247	0.12	0.21	-0.06	-0.20	0.36
Tocochromanol Pathway	GRMZM2G009785	tocopherol cyclase	δT3/(γT3+αT3)	S5_133331106	GBS	5	133,331,106	-170,822	-187,389	1.60E-05	6.10E-03	0.40	0.47	0.41	248	0.10	0.17	0.03	-0.10	-0.26
Tocochromanol Pathway	GRMZM2G009785	tocopherol cyclase	δT3/αT3	S5_133331106	GBS	5	133,331,106	-170,822	-187,389	2.10E-05	2.76E-02	0.40	0.47	0.41	246	0.14	0.20	0.05	-0.15	-0.28
Tocochromanol Pathway	GRMZM2G009785	tocopherol cyclase	δT3	S5_133331106	GBS	5	133,331,106	-170,822	-187,389	1.15E-06	1.75E-03	0.40	0.47	0.41	247	0.12	0.21	0.06	-0.20	-0.25
Tocochromanol Pathway	GRMZM2G009785	tocopherol cyclase	δT3/(γT3+αT3)	ss196465630	55K	5	133,332,323	-169,605	-186,172	6.91E-05	1.76E-02	0.32	0.11	0.36	248	0.10	0.16	-0.03	-0.10	0.36
Tocochromanol Pathway	GRMZM2G009785	tocopherol cyclase	δT3/(γT3+αT3)	ss196465628	55K	5	133,333,095	-168,833	-185,400	4.48E-05	1.24E-02	0.42	0.47	0.42	248	0.10	0.17	-0.03	-0.10	0.36
Tocochromanol Pathway	GRMZM2G009785	tocopherol cyclase	δT3/αT3	ss196465628	55K	5	133,333,095	-168,833	-185,400	9.53E-05	4.83E-02	0.42	0.47	0.42	246	0.14	0.19	-0.05	-0.15	0.41
Tocochromanol Pathway	GRMZM2G009785	tocopherol cyclase	δT3	ss196465628	55K	5	133,333,095	-168,833	-185,400	4.21E-06	3.67E-03	0.42	0.47	0.42	247	0.12	0.20	-0.06	-0.20	0.36
Tocochromanol Pathway	GRMZM2G009785	tocopherol cyclase	δT3/(γT3+αT3)	S5_133333397	GBS	5	133,333,397	-168,531	-185,098	6.99E-06	4.59E-03	0.43	0.47	0.43	248	0.10	0.18	0.03	-0.10	-0.26
Tocochromanol Pathway	GRMZM2G009785	tocopherol cyclase	δT3/(γT3+αT3)	ss196465626	55K	5	133,333,397	-168,531	-185,098	2.53E-05	8.58E-03	0.42	0.47	0.42	248	0.10	0.17	0.03	-0.10	-0.26
Tocochromanol Pathway	GRMZM2G009785	tocopherol cyclase	δT3/αT3	S5_133333397	GBS	5	133,333,397	-168,531	-185,098	2.27E-05	2.76E-02	0.43	0.47	0.43	246	0.14	0.20	0.05	-0.15	-0.28
Tocochromanol Pathway	GRMZM2G009785	tocopherol cyclase	δT3/αT3	ss196465626	55K	5	133,333,397	-168,531	-185,098	5.49E-05	3.42E-02	0.42	0.47	0.42	246	0.14	0.20	0.05	-0.15	-0.28
Tocochromanol Pathway	GRMZM2G009785	tocopherol cyclase	δT3	S5_133333397	GBS	5	133,333,397	-168,531	-185,098	3.88E-07	1.19E-03	0.43	0.47	0.43	247	0.12	0.22	0.06	-0.20	-0.25
Tocochromanol Pathway	GRMZM2G009785	tocopherol cyclase	δT3	ss196465626	55K	5	133,333,397	-168,531	-185,098	1.85E-06	1.88E-03	0.42	0.47	0.42	247	0.12	0.21	0.06	-0.20	-0.25
Tocochromanol Pathway	GRMZM2G009785	tocopherol cyclase	δT3/(γT3+αT3)	S5_133333561	GBS	5	133,333,561	-168,367	-184,934	8.69E-06	4.59E-03	0.42	0.48	0.43	248	0.10	0.18	0.03	-0.10	-0.26
Tocochromanol Pathway	GRMZM2G009785	tocopherol cyclase	δT3/αT3	S5_133333561	GBS	5	133,333,561	-168,367	-184,934	2.06E-05	2.76E-02	0.42	0.48	0.43	246	0.14	0.20	0.05	-0.15	-0.28
Tocochromanol Pathway	GRMZM2G009785	tocopherol cyclase	δT3	S5_133333561	GBS	5	133,333,561	-168,367	-184,934	3.67E-07	1.19E-03	0.42	0.48	0.43	247	0.12	0.22	0.06	-0.20	-0.25
Tocochromanol Pathway	GRMZM2G009785	tocopherol cyclase	δT3/(γT3+αT3)	S5_133335078	GBS	5	133,335,078	-166,850	-183,417	1.50E-05	6.10E-03	0.30	0.33	0.29	248	0.10	0.17	-0.03	-0.10	0.36
Tocochromanol Pathway	GRMZM2G009785	tocopherol cyclase	δT3/αT3	S5_133335078	GBS	5	133,335,078	-166,850	-183,417	5.98E-05	3.42E-02	0.30	0.33	0.29	246	0.14	0.20	-0.06	-0.15	0.51
Tocochromanol Pathway	GRMZM2G009785	tocopherol cyclase	δT3	S5_133335078	GBS	5	133,335,078	-166,850	-183,417	1.49E-06	1.82E-03	0.30	0.33	0.29	247	0.12	0.21	-0.06	-0.20	0.36
Tocochromanol Pathway	GRMZM2G009785	tocopherol cyclase	δT3/(γT3+αT3)	S5_133338747	GBS	5	133,338,747	-163,181	-179,748	2.95E-05	9.47E-03	0.37	0.17	0.41	248	0.10	0.17	-0.03	-0.10	0.36
Tocochromanol Pathway	GRMZM2G009785	tocopherol cyclase	δT3/γT3	S5_133338747	GBS	5	133,338,747	-163,181	-179,748	4.80E-05	2.93E-02	0.37	0.17	0.41	249	0.13	0.19	0.01	0.05	0.22
Tocochromanol Pathway	GRMZM2G009785	tocopherol cyclase	δT/γT	PZA00524.2	4K	5	133,338,936	-162,992	-179,559	1.64E-05	4.42E-02	0.41	0.23	0.39	249	0.14	0.21	-0.02	0.30	-0.07
Tocochromanol Pathway	GRMZM2G009785	tocopherol cyclase	δT/γT	S5_133338936	GBS	5	133,338,936	-162,992	-179,559	1.80E-05	4.42E-02	0.47	0.17	0.40	249	0.14	0.21	0.02	0.30	0.07
Tocochromanol Pathway	GRMZM2G009785	tocopherol cyclase	δT3/(γT3+αT3)	S5_133499169	GBS	5	133,499,169	-2,759	-19,326	1.98E-06	3.02E-03	0.39	0.15	0.44	248	0.10	0.19	-0.03	-0.10	0.36
Tocochromanol Pathway	GRMZM2G009785	tocopherol cyclase	δT3/αT3	S5_133499169	GBS	5	133,499,169	-2,759	-19,326	6.13E-05	3.42E-02	0.39	0.15	0.44	246	0.14	0.20	-0.05	-0.15	0.41
Tocochromanol Pathway	GRMZM2G009785	tocopherol cyclase	δT3/γT3	S5_133499169	GBS	5	133,499,169	-2,759	-19,326	8.05E-05	4.10E-02	0.39	0.15	0.44	249	0.13	0.19	0.01	0.05	0.22
Tocochromanol Pathway	GRMZM2G009785	tocopherol cyclase	δT3	S5_133499169	GBS	5	133,499,169	-2,759	-19,326	1.31E-04	4.71E-02	0.39	0.15	0.44	247	0.12	0.18	-0.05	-0.20	0.29
Tocochromanol Pathway	GRMZM2G009785	tocopherol cyclase	δT3/(γT3+αT3)	S5_133499269	GBS	5	133,499,269	-2,659	-19,226	4.23E-06	4.59E-03	0.39	0.18	0.46	248	0.10	0.18	0.03	-0.10	-0.26
Tocochromanol Pathway	GRMZM2G009785	tocopherol cyclase	δT3/(γT3+αT3)	S5_133501858	GBS	5	133,501,858	-70	-16,637	1.58E-07	9.62E-04	0.39	0.19	0.46	248	0.10	0.21	0.04	-0.10	-0.32
Tocochromanol Pathway	GRMZM2G009785	tocopherol cyclase	δT3/αT3	S5_133501858	GBS	5	133,501,858	-70	-16,637	1.55E-05	2.76E-02	0.39	0.19	0.46	246	0.14	0.21	0.06	-0.15	-0.32
Tocochromanol Pathway	GRMZM2G009785	tocopherol cyclase	δT3/γT3	S5_133501858	GBS	5	133,501,858	-70	-16,637	1.34E-06	8.18E-03	0.39	0.19	0.46	249	0.13	0.22	-0.01	0.05	-0.18
Tocochromanol Pathway	GRMZM2G009785	tocopherol cyclase	δT3	S5_133501858	GBS	5	133,501,858	-70	-16,637	7.85E-06	5.32E-03	0.39	0.19	0.46	247	0.12	0.20	0.06	-0.20	-0.25
Tocochromanol Pathway	GRMZM2G009785	tocopherol cyclase	δT3/(γT3+αT3)	S5_133501992	GBS	5	133,501,992	64	-16,503	1.37E-06	2.79E-03	0.39	0.14	0.46	248	0.10	0.19	0.03	-0.10	-0.26
Tocochromanol Pathway	GRMZM2G009785	tocopherol cyclase	δT3/αT3	S5_133501992	GBS	5	133,501,992	64	-16,503	5.72E-05	3.42E-02	0.39	0.14	0.46	246	0.14	0.20	0.05	-0.15	-0.28
Tocochromanol Pathway	GRMZM2G009785	tocopherol cyclase	δT3/γT3	S5_133501992	GBS	5	133,501,992	64	-16,503	4.15E-06	8.46E-03	0.39	0.14	0.46	249	0.13	0.21	-0.01	0.05	-0.18
Tocochromanol Pathway	GRMZM2G009785	tocopherol cyclase	δT3	S5_133501992	GBS	5	133,501,992	64	-16,503	1.92E-05	1.06E-02	0.39	0.14	0.46	247	0.12	0.19	0.06	-0.20	-0.25
Tocochromanol Pathway	GRMZM2G009785	tocopherol cyclase	δT3/(γT3+αT3)	PZB00969.1	4K	5	133,502,506	578	-15,989	1.17E-06	2.79E-03	0.40	0.14	0.46	248	0.10	0.19	0.03	-0.10	-0.26
Tocochromanol Pathway	GRMZM2G009785	tocopherol cyclase	δT3/(γT3+αT3)	ss196416168	55K	5	133,502,506	578	-15,989	1.74E-05	6.24E-03	0.40	0.13	0.46	248	0.10	0.17	0.03	-0.10	-0.26
Tocochromanol Pathway	GRMZM2G009785	tocopherol cyclase	δT3/αT3	PZB00969.1	4K	5	133,502,506	578	-15,989	6.09E-05	3.42E-02	0.40	0.14	0.46	246	0.14	0.20	0.05	-0.15	-0.28
Tocochromanol Pathway	GRMZM2G009785	tocopherol cyclase	δT3/γT3	PZB00969.1	4K	5	133,502,506	578	-15,989	2.74E-06	8.36E-03	0.40	0.14	0.46	249	0.13	0.21	-0.01	0.05	-0.18
Tocochromanol Pathway	GRMZM2G009785	tocopherol cyclase	δT3/γT3	ss196416168	55K	5	133,502,506	578	-15,989	3.19E-05	2.17E-02	0.40	0.13	0.46	249	0.13	0.20	-0.01	0.05	-0.18

Tocochromanol Pathway	GRMZM2G009785	tocopherol cyclase	δT3	PZB00969.1	4K	5	133,502,506	578	-15,989	1.65E-05	1.01E-02	0.40	0.14	0.46	247	0.12	0.19	0.06	-0.20	-0.25
Tocochromanol Pathway	GRMZM2G009785	tocopherol cyclase	δT3	ss196416168	55K	5	133,502,506	578	-15,989	1.63E-04	4.97E-02	0.40	0.13	0.46	247	0.12	0.18	0.05	-0.20	-0.22
Tocochromanol Pathway	GRMZM2G009785	tocopherol cyclase	δT3/(γT3+αT3)	ss196465634	55K	5	133,510,613	8,685	-7,882	9.77E-06	4.59E-03	0.41	0.14	0.46	248	0.10	0.18	-0.03	-0.10	0.36
Tocochromanol Pathway	GRMZM2G009785	tocopherol cyclase	δT3/γT3	ss196465634	55K	5	133,510,613	8,685	-7,882	1.39E-05	1.84E-02	0.41	0.14	0.46	249	0.13	0.20	0.01	0.05	0.22
Tocochromanol Pathway	GRMZM2G009785	tocopherol cyclase	δT3	ss196465634	55K	5	133,510,613	8,685	-7,882	1.03E-04	4.71E-02	0.41	0.14	0.46	247	0.12	0.18	-0.05	-0.20	0.29
Tocochromanol Pathway	GRMZM2G009785	tocopherol cyclase	δT3/(γT3+αT3)	PZB02491.1	4K	5	133,517,065	15,137	-1,430	8.41E-06	4.59E-03	0.40	0.14	0.46	248	0.10	0.18	-0.03	-0.10	0.36
Tocochromanol Pathway	GRMZM2G009785	tocopherol cyclase	δT3/γT3	PZB02491.1	4K	5	133,517,065	15,137	-1,430	7.72E-05	4.10E-02	0.40	0.14	0.46	249	0.13	0.19	0.01	0.05	0.22
Tocochromanol Pathway	GRMZM2G009785	tocopherol cyclase	δT3	PZB02491.1	4K	5	133,517,065	15,137	-1,430	6.16E-05	3.13E-02	0.40	0.14	0.46	247	0.12	0.18	-0.05	-0.20	0.29
Tocochromanol Pathway	GRMZM2G009785	tocopherol cyclase	δT3/(γT3+αT3)	S5_133618308	GBS	5	133,618,308	116,380	99,813	9.54E-06	4.59E-03	0.40	0.10	0.48	248	0.10	0.18	0.03	-0.10	-0.26
Tocochromanol Pathway	GRMZM2G009785	tocopherol cyclase	δT3/γT3	S5_133618308	GBS	5	133,618,308	116,380	99,813	2.36E-05	1.84E-02	0.40	0.10	0.48	249	0.13	0.20	-0.01	0.05	-0.18
Tocochromanol Pathway	GRMZM2G009785	tocopherol cyclase	δT3	S5_133618308	GBS	5	133,618,308	116,380	99,813	1.31E-04	4.71E-02	0.40	0.10	0.48	247	0.12	0.18	0.05	-0.20	-0.22
Tocochromanol Pathway	GRMZM2G009785	tocopherol cyclase	δT3/(γT3+αT3)	S5_133618309	GBS	5	133,618,309	116,381	99,814	9.54E-06	4.59E-03	0.40	0.10	0.48	248	0.10	0.18	0.03	-0.10	-0.26
Tocochromanol Pathway	GRMZM2G009785	tocopherol cyclase	δT3/γT3	S5_133618309	GBS	5	133,618,309	116,381	99,814	2.36E-05	1.84E-02	0.40	0.10	0.48	249	0.13	0.20	-0.01	0.05	-0.18
Tocochromanol Pathway	GRMZM2G009785	tocopherol cyclase	δT3	S5_133618309	GBS	5	133,618,309	116,381	99,814	1.31E-04	4.71E-02	0.40	0.10	0.48	247	0.12	0.18	0.05	-0.20	-0.22
Tocochromanol Pathway	GRMZM2G009785	tocopherol cyclase	δT3/(γT3+αT3)	S5_133618344	GBS	5	133,618,344	116,416	99,849	9.54E-06	4.59E-03	0.40	0.10	0.48	248	0.10	0.18	0.03	-0.10	-0.26
Tocochromanol Pathway	GRMZM2G009785	tocopherol cyclase	δT3/γT3	S5_133618344	GBS	5	133,618,344	116,416	99,849	2.36E-05	1.84E-02	0.40	0.10	0.48	249	0.13	0.20	-0.01	0.05	-0.18
Tocochromanol Pathway	GRMZM2G009785	tocopherol cyclase	δT3	S5_133618344	GBS	5	133,618,344	116,416	99,849	1.31E-04	4.71E-02	0.40	0.10	0.48	247	0.12	0.18	0.05	-0.20	-0.22
Tocochromanol Pathway	GRMZM2G009785	tocopherol cyclase	δT/γT	S5_133618788	GBS	5	133,618,788	116,860	100,293	2.89E-05	4.42E-02	0.49	0.11	0.42	249	0.14	0.20	0.02	0.30	0.07
Tocochromanol Pathway	GRMZM2G009785	tocopherol cyclase	δT3/(γT3+αT3)	S5_133618788	GBS	5	133,618,788	116,860	100,293	1.85E-04	4.33E-02	0.49	0.11	0.42	248	0.10	0.16	-0.03	-0.10	0.36
Tocochromanol Pathway	GRMZM2G009785	tocopherol cyclase	δT/γT	S5_133618810	GBS	5	133,618,810	116,882	100,315	2.89E-05	4.42E-02	0.49	0.11	0.42	249	0.14	0.20	0.02	0.30	0.07
Tocochromanol Pathway	GRMZM2G009785	tocopherol cyclase	δT3/(γT3+αT3)	S5_133618810	GBS	5	133,618,810	116,882	100,315	1.85E-04	4.33E-02	0.49	0.11	0.42	248	0.10	0.16	-0.03	-0.10	0.36
Tocochromanol Pathway	GRMZM2G009785	tocopherol cyclase	δT3/(γT3+αT3)	S5_133691297	GBS	5	133,691,297	189,369	172,802	5.54E-06	4.59E-03	0.37	0.10	0.46	248	0.10	0.18	0.03	-0.10	-0.26
Tocochromanol Pathway	GRMZM2G009785	tocopherol cyclase	δT3/γT3	S5_133691297	GBS	5	133,691,297	189,369	172,802	2.41E-05	1.84E-02	0.37	0.10	0.46	249	0.13	0.20	-0.01	0.05	-0.18
Tocochromanol Pathway	GRMZM2G009785	tocopherol cyclase	δT3/(γT3+αT3)	ss196465642	55K	5	133,728,050	226,122	209,555	5.07E-05	1.35E-02	0.40	0.11	0.46	248	0.10	0.17	-0.03	-0.10	0.36
Tocochromanol Pathway	GRMZM2G173358	homogentisic acid geranylgeranyl transferase	Total Tocotrienols	S9_92346116	GBS	9	92,346,116	-137,433	-141,152	7.27E-06	4.40E-02	0.41	0.43	0.44	250	0.20	0.27	0.03	0.15	0.22
Tocochromanol Pathway	GRMZM2G173358	homogentisic acid geranylgeranyl transferase	γT3	S9_92346116	GBS	9	92,346,116	-137,433	-141,152	2.33E-06	3.56E-03	0.41	0.43	0.44	250	0.20	0.28	0.03	0.10	0.34
Tocochromanol Pathway	GRMZM2G173358	homogentisic acid geranylgeranyl transferase	Total Tocopherols/Total Tocotrienols	S9_92548696	GBS	9	92,548,696	65,147	61,428	1.02E-05	3.13E-02	0.39	0.38	0.40	247	0.19	0.26	-0.01	0.05	-0.21
Tocochromanol Pathway	GRMZM2G173358	homogentisic acid geranylgeranyl transferase	Total Tocopherols/Total Tocotrienols	S9_92554465	GBS	9	92,554,465	70,916	67,197	2.75E-06	1.67E-02	0.26	0.00	0.32	247	0.19	0.27	-0.01	0.05	-0.24
Tocochromanol Pathway	GRMZM2G173358	homogentisic acid geranylgeranyl transferase	γT3/(γT3+αT3)	S9_92718671	GBS	9	92,718,671	235,122	231,403	5.56E-06	1.13E-02	0.08	0.00	0.13	251	0.20	0.27	-0.10	1.35	-0.08
Tocochromanol Pathway	GRMZM2G173358	homogentisic acid geranylgeranyl transferase	γT3	S9_92718671	GBS	9	92,718,671	235,122	231,403	4.49E-07	9.16E-04	0.08	0.00	0.13	250	0.20	0.29	-0.05	0.10	-0.4
Tocochromanol Pathway	GRMZM2G173358	homogentisic acid geranylgeranyl transferase	γT3/(γT3+αT3)	S9_92718674	GBS	9	92,718,674	235,125	231,406	5.56E-06	1.13E-02	0.08	0.00	0.13	251	0.20	0.27	-0.10	1.35	-0.08
Tocochromanol Pathway	GRMZM2G173358	homogentisic acid geranylgeranyl transferase	γT3	S9_92718674	GBS	9	92,718,674	235,125	231,406	4.49E-07	9.16E-04	0.08	0.00	0.13	250	0.20	0.29	-0.05	0.10	-0.4
Tocochromanol Pathway	GRMZM2G173358	homogentisic acid geranylgeranyl transferase	γT3/(γT3+αT3)	S9_92718709	GBS	9	92,718,709	235,160	231,441	5.56E-06	1.13E-02	0.08	0.00	0.13	251	0.20	0.27	-0.10	1.35	-0.08
Tocochromanol Pathway	GRMZM2G173358	homogentisic acid geranylgeranyl transferase	γT3	S9_92718709	GBS	9	92,718,709	235,160	231,441	4.49E-07	9.16E-04	0.08	0.00	0.13	250	0.20	0.29	-0.05	0.10	-0.4

Table S7c Statistically significant results from the candidate gene association study of 20 tocochromanol grain traits with the two SNPs in the region including ZmVTE1 identified in the multi-locus mixed-model (MLMM) analysis included as covariates. SNPs that were significantly associated with the indicated trait at 5% FDR are shown.

a priori candidate gene pathway	RefGen_v2 Gene ID	RefGen_v2 Annotated Gene Function	Trait	SNP ID	SNP Source	Chr	Position	Distance from Gene ORF Start	Distance from Gene ORF Finish	*P*-value	FDR Adjusted *P*-value	Minor Allele Frequency (MAF)	MAF Tropical (18% of 252 Lines)	MAF Temperate (82% of 252 Lines)	Sample Size	R^2_{LR} from Model without SNP	R^2_{LR} from Model with SNP	Effect Size	Lambda from Box-Cox Procedure	Back-Transformed Effect Estimates
Aromatic Head Group	GRMZM2G437912	prephenate dehydratase	γT3	S2_59013838	GBS	2	59,013,838	-23,405	-25,340	3.44E-05	3.01E-02	0.06	0.40	0.09	250	0.23	0.28	0.04	0.10	0.47
Aromatic Head Group	GRMZM2G437912	prephenate dehydratase	Total Tocotrienols	S2_59013838	GBS	2	59,013,838	-23,405	-25,340	1.45E-05	2.96E-02	0.06	0.40	0.09	250	0.23	0.29	0.06	0.15	0.43
Aromatic Head Group	GRMZM2G437912	prephenate dehydratase	Total Tocotrienols	S2_59013840	GBS	2	59,013,840	-23,403	-25,338	1.45E-05	2.96E-02	0.06	0.40	0.09	250	0.23	0.29	-0.06	0.15	-0.32
Aromatic Head Group	GRMZM2G437912	prephenate dehydratase	γT3	S2_59013840	GBS	2	59,013,840	-23,403	-25,338	3.44E-05	3.01E-02	0.06	0.40	0.09	250	0.23	0.28	-0.04	0.10	-0.33
Aromatic Head Group	GRMZM2G573867	3-dehydroquinate synthase	δT/αT	S2_196295236	GBS	2	196,295,236	-64,391	-68,456	1.71E-04	4.51E-02	0.06	0.04	0.07	246	0.28	0.33	-0.48	0.00	-0.83
Aromatic Head Group	GRMZM2G573867	3-dehydroquinate synthase	αT	S2_196514167	GBS	2	196,514,167	154,540	150,475	9.47E-06	2.90E-03	0.06	0.02	0.07	251	0.25	0.32	-0.36	0.40	-0.67
Aromatic Head Group	GRMZM2G138624	isochorismatase hydrolase	αT3	ss196456226	55K	4	134,020,078	96,788	94,084	1.27E-05	2.70E-02	0.45	0.45	0.46	248	0.21	0.28	0.01	0.05	0.14
Aromatic Head Group	GRMZM2G124365	chorismate mutase	αT	S8_173041758	GBS	8	173,041,758	-61,455	-65,990	2.70E-04	4.59E-02	0.12	0.18	0.22	251	0.25	0.30	-0.23	0.40	-0.49
Aromatic Head Group	GRMZM2G124365	chorismate mutase	δT/αT	S8_173070782	GBS	8	173,070,782	-32,431	-36,966	1.50E-04	4.16E-02	0.18	0.04	0.33	246	0.28	0.33	-0.36	0.00	-1.05
Aromatic Head Group	GRMZM2G124365	chorismate mutase	Total Tocotrienols	S8_173243067	GBS	8	173,243,067	139,854	135,319	5.41E-05	4.95E-02	0.09	0.20	0.09	250	0.23	0.28	0.05	0.15	-0.3
Aromatic Head Group	GRMZM2G124365	chorismate mutase	αT3	S8_173243067	GBS	8	173,243,067	139,854	135,319	4.36E-05	4.88E-02	0.09	0.20	0.09	248	0.21	0.27	0.01	0.05	0.2
Prenyl Group Synthesis	GRMZM2G027059	4-hydroxy-3-methylbut-2-enyldiphosphate reductase	αT	S1_273098217	GBS	1	273,098,217	161,381	157,715	3.50E-05	8.56E-03	0.22	0.24	0.24	251	0.25	0.31	-0.21	0.40	-0.44
Prenyl Group Synthesis	AC209374.4_FG002	2-C-methyl-D-erythritol 2,4-cyclodiphosphate synthase	αT/γT	S5_196278264	GBS	5	196,278,264	-1,031	-2,773	1.29E-04	3.57E-02	0.25	0.17	0.30	246	0.25	0.29	0.04	0.15	0.29
Prenyl Group Synthesis	AC209374.4_FG002	2-C-methyl-D-erythritol 2,4-cyclodiphosphate synthase	αT	S5_196278264	GBS	5	196,278,264	-1,031	-2,773	1.36E-04	2.87E-02	0.25	0.17	0.30	251	0.25	0.30	0.19	0.40	0.55
Prenyl Group Synthesis	AC209374.4_FG002	2-C-methyl-D-erythritol 2,4-cyclodiphosphate synthase	γT/(γT+αT)	S5_196278264	GBS	5	196,278,264	-1,031	-2,773	6.18E-05	2.10E-02	0.25	0.17	0.30	251	0.29	0.34	-0.07	2.00	-0.04
Prenyl Group Synthesis	AC209374.4_FG002	2-C-methyl-D-erythritol 2,4-cyclodiphosphate synthase	δT/αT	S5_196278264	GBS	5	196,278,264	-1,031	-2,773	2.46E-05	8.78E-03	0.25	0.17	0.30	246	0.28	0.34	-0.36	0.00	-0.84
Prenyl Group Synthesis	GRMZM2G133082	isopentenyl pyrophosphate isomerase	γT/(γT+αT)	S6_147333828	GBS	6	147,333,828	202,712	197,149	2.19E-04	4.79E-02	0.07	0.22	0.05	251	0.29	0.33	-0.11	2.00	-0.06
Prenyl Group Synthesis	GRMZM2G133082	isopentenyl pyrophosphate isomerase	γT/(γT+αT)	S6_147333833	GBS	6	147,333,833	202,717	197,154	2.19E-04	4.79E-02	0.07	0.22	0.05	251	0.29	0.33	-0.11	2.00	-0.06
Prenyl Group Synthesis	GRMZM2G493395	1-deoxy-D-xylulose 5-phosphate synthase	αT/γT	ss196475603	55K	7	13,959,219	-118,633	-121,856	1.58E-05	4.81E-03	0.30	0.37	0.30	246	0.25	0.31	0.04	0.15	0.3
Prenyl Group Synthesis	GRMZM2G493395	1-deoxy-D-xylulose 5-phosphate synthase	γT/(γT+αT)	ss196475603	55K	7	13,959,219	-118,633	-121,856	4.39E-05	1.58E-02	0.30	0.37	0.30	251	0.29	0.34	-0.07	2.00	-0.03
Tocochromanol Pathway	GRMZM2G035213	γ-tocopherol methyltransferase	αT	S5_200117524	GBS	5	200,117,524	-249,505	-253,327	3.88E-05	9.14E-03	0.21	0.02	0.27	251	0.25	0.31	0.22	0.40	0.66
Tocochromanol Pathway	GRMZM2G035213	γ-tocopherol methyltransferase	αT	S5_200117526	GBS	5	200,117,526	-249,503	-253,325	2.14E-04	3.97E-02	0.22	0.07	0.27	251	0.25	0.30	0.20	0.40	0.73
Tocochromanol Pathway	GRMZM2G035213	γ-tocopherol methyltransferase	αT	S5_200119597	GBS	5	200,119,597	-247,432	-251,254	1.95E-04	3.73E-02	0.33	0.18	0.36	251	0.25	0.30	0.17	0.40	0.44
Tocochromanol Pathway	GRMZM2G035213	γ-tocopherol methyltransferase	αT	S5_200119623	GBS	5	200,119,623	-247,406	-251,228	2.80E-04	4.64E-02	0.35	0.27	0.37	251	0.25	0.30	0.16	0.40	-0.47
Tocochromanol Pathway	GRMZM2G035213	γ-tocopherol methyltransferase	γT/(γT+αT)	S5_200130689	GBS	5	200,130,689	-236,340	-240,162	7.59E-05	2.21E-02	0.07	0.33	0.08	251	0.29	0.34	0.12	2.00	0.06
Tocochromanol Pathway	GRMZM2G035213	γ-tocopherol methyltransferase	δT/αT	S5_200130689	GBS	5	200,130,689	-236,340	-240,162	1.82E-04	4.61E-02	0.07	0.33	0.08	246	0.28	0.33	0.54	0.00	-0.83
Tocochromanol Pathway	GRMZM2G035213	γ-tocopherol methyltransferase	γT/(γT+αT)	S5_200292465	GBS	5	200,292,465	-74,564	-78,386	7.49E-05	2.21E-02	0.34	0.34	0.28	251	0.29	0.34	-0.06	2.00	-0.03
Tocochromanol Pathway	GRMZM2G035213	γ-tocopherol methyltransferase	αT/γT	S5_200293693	GBS	5	200,293,693	-73,336	-77,158	1.02E-05	3.64E-03	0.41	0.34	0.37	246	0.25	0.31	0.04	0.15	0.29
Tocochromanol Pathway	GRMZM2G035213	γ-tocopherol methyltransferase	αT	S5_200293693	GBS	5	200,293,693	-73,336	-77,158	3.70E-06	1.33E-03	0.41	0.34	0.37	251	0.25	0.32	0.20	0.40	0.58
Tocochromanol Pathway	GRMZM2G035213	γ-tocopherol methyltransferase	γT/(γT+αT)	S5_200293693	GBS	5	200,293,693	-73,336	-77,158	1.59E-06	8.86E-04	0.41	0.34	0.37	251	0.29	0.36	-0.07	2.00	-0.04
Tocochromanol Pathway	GRMZM2G035213	γ-tocopherol methyltransferase	δT/αT	S5_200293693	GBS	5	200,293,693	-73,336	-77,158	2.21E-05	8.39E-03	0.41	0.34	0.37	246	0.28	0.34	-0.31	0.00	-0.73
Tocochromanol Pathway	GRMZM2G035213	γ-tocopherol methyltransferase	αT	ss196468356	55K	5	200,300,836	-66,193	-70,015	9.23E-05	2.09E-02	0.43	0.20	0.49	251	0.25	0.30	-0.17	0.40	-0.37
Tocochromanol Pathway	GRMZM2G035213	γ-tocopherol methyltransferase	δT/αT	ss196468356	55K	5	200,300,836	-66,193	-70,015	4.99E-06	2.53E-03	0.43	0.20	0.49	246	0.28	0.35	0.34	0.00	1.09
Tocochromanol Pathway	GRMZM2G035213	γ-tocopherol methyltransferase	αT/γT	S5_200318615	GBS	5	200,318,615	-48,414	-52,236	3.21E-06	1.51E-03	0.22	0.27	0.44	246	0.25	0.32	0.05	0.15	0.37
Tocochromanol Pathway	GRMZM2G035213	γ-tocopherol methyltransferase	αT	S5_200318615	GBS	5	200,318,615	-48,414	-52,236	1.42E-06	5.81E-04	0.22	0.27	0.44	251	0.25	0.33	0.25	0.40	0.74
Tocochromanol Pathway	GRMZM2G035213	γ-tocopherol methyltransferase	γT/(γT+αT)	S5_200318615	GBS	5	200,318,615	-48,414	-52,236	2.36E-05	9.65E-03	0.22	0.27	0.44	251	0.29	0.34	-0.08	2.00	-0.04
Tocochromanol Pathway	GRMZM2G035213	γ-tocopherol methyltransferase	δT/αT	S5_200318615	GBS	5	200,318,615	-48,414	-52,236	9.52E-05	2.89E-02	0.22	0.27	0.44	246	0.28	0.33	-0.34	0.00	-0.8
Tocochromanol Pathway	GRMZM2G035213	γ-tocopherol methyltransferase	αT/γT	PZB02283.1	4K	5	200,367,532	503	-3,319	1.04E-12	2.11E-09	0.20	0.14	0.22	246	0.25	0.42	0.08	0.15	0.64
Tocochromanol Pathway	GRMZM2G035213	γ-tocopherol methyltransferase	αT/γT	ss196416269	55K	5	200,367,532	503	-3,319	1.04E-12	2.11E-09	0.20	0.13	0.22	246	0.25	0.42	0.08	0.15	0.64
Tocochromanol Pathway	GRMZM2G035213	γ-tocopherol methyltransferase	αT	PZB02283.1	4K	5	200,367,532	503	-3,319	1.01E-13	2.07E-10	0.20	0.14	0.22	251	0.25	0.44	0.40	0.40	1.31
Tocochromanol Pathway	GRMZM2G035213	γ-tocopherol methyltransferase	αT	ss196416269	55K	5	200,367,532	503	-3,319	1.01E-13	2.07E-10	0.20	0.13	0.22	251	0.25	0.44	0.40	0.40	1.31
Tocochromanol Pathway	GRMZM2G035213	γ-tocopherol methyltransferase	γT/(γT+αT)	PZB02283.1	4K	5	200,367,532	503	-3,319	4.98E-12	1.02E-08	0.20	0.14	0.22	251	0.29	0.44	-0.13	2.00	-0.07
Tocochromanol Pathway	GRMZM2G035213	γ-tocopherol methyltransferase	γT/(γT+αT)	ss196416269	55K	5	200,367,532	503	-3,319	4.98E-12	1.02E-08	0.20	0.13	0.22	251	0.29	0.44	-0.13	2.00	-0.07
Tocochromanol Pathway	GRMZM2G035213	γ-tocopherol methyltransferase	δT/αT	PZB02283.1	4K	5	200,367,532	503	-3,319	2.46E-11	4.98E-08	0.20	0.14	0.22	246	0.28	0.43	-0.63	0.00	-1.27
Tocochromanol Pathway	GRMZM2G035213	γ-tocopherol methyltransferase	δT/αT	ss196416269	55K	5	200,367,532	503	-3,319	2.46E-11	4.98E-08	0.20	0.13	0.22	246	0.28	0.43	-0.63	0.00	-1.27
Tocochromanol Pathway	GRMZM2G035213	γ-tocopherol methyltransferase	αT/γT	ss196468362	55K	5	200,369,124	2,095	-1,727	1.04E-12	2.11E-09	0.20	0.13	0.22	246	0.25	0.42	0.08	0.15	0.64
Tocochromanol Pathway	GRMZM2G035213	γ-tocopherol methyltransferase	αT	ss196468362	55K	5	200,369,124	2,095	-1,727	1.01E-13	2.07E-10	0.20	0.13	0.22	251	0.25	0.44	0.40	0.40	1.31
Tocochromanol Pathway	GRMZM2G035213	γ-tocopherol methyltransferase	γT/(γT+αT)	ss196468362	55K	5	200,369,124	2,095	-1,727	4.98E-12	1.02E-08	0.20	0.13	0.22	251	0.29	0.44	-0.13	2.00	-0.07
Tocochromanol Pathway	GRMZM2G035213	γ-tocopherol methyltransferase	δT/αT	ss196468362	55K	5	200,369,124	2,095	-1,727	2.46E-11	4.98E-08	0.20	0.13	0.22	246	0.28	0.43	-0.63	0.00	-1.27
Tocochromanol Pathway	GRMZM2G035213	γ-tocopherol methyltransferase	αT/γT	S5_200369481	GBS	5	200,369,481	2,452	-1,370	5.14E-07	2.85E-04	0.11	0.36	0.09	246	0.25	0.33	0.07	0.15	0.57

Tocochromanol Pathway	GRMZM2G035213	γ-tocopherol methyltransferase	αT	S5_200369481	GBS	5	200,369,481	2,452	-1,370	1.81E-08	1.58E-05	0.11	0.36	0.09	251	0.25	0.36	0.38	0.40	1.23
Tocochromanol Pathway	GRMZM2G035213	γ-tocopherol methyltransferase	γT/(γT+αT)	S5_200369481	GBS	5	200,369,481	2,452	-1,370	5.10E-09	6.24E-06	0.11	0.36	0.09	251	0.29	0.40	-0.14	2.00	-0.07
Tocochromanol Pathway	GRMZM2G035213	γ-tocopherol methyltransferase	δT/(γT+αT)	S5_200369481	GBS	5	200,369,481	2,452	-1,370	7.53E-06	4.61E-02	0.11	0.36	0.09	251	0.21	0.27	-0.03	0.35	-0.07
Tocochromanol Pathway	GRMZM2G035213	γ-tocopherol methyltransferase	δT/αT	S5_200369481	GBS	5	200,369,481	2,452	-1,370	5.29E-07	4.02E-04	0.11	0.36	0.09	246	0.28	0.36	-0.57	0.00	-1.18
Tocochromanol Pathway	GRMZM2G035213	γ-tocopherol methyltransferase	αT/γT	S5_200369508	GBS	5	200,369,508	2,479	-1,343	7.52E-07	3.82E-04	0.18	0.11	0.22	246	0.25	0.33	0.06	0.15	0.44
Tocochromanol Pathway	GRMZM2G035213	γ-tocopherol methyltransferase	αT3/γT3	S5_200369508	GBS	5	200,369,508	2,479	-1,343	1.30E-05	2.64E-02	0.18	0.11	0.22	246	0.15	0.21	-0.05	-0.15	0.39
Tocochromanol Pathway	GRMZM2G035213	γ-tocopherol methyltransferase	αT	S5_200369508	GBS	5	200,369,508	2,479	-1,343	6.77E-07	3.19E-04	0.18	0.11	0.22	251	0.25	0.33	0.28	0.40	0.85
Tocochromanol Pathway	GRMZM2G035213	γ-tocopherol methyltransferase	γT/(γT+αT)	S5_200369508	GBS	5	200,369,508	2,479	-1,343	5.11E-07	3.13E-04	0.18	0.11	0.22	251	0.29	0.37	-0.10	2.00	-0.05
Tocochromanol Pathway	GRMZM2G035213	γ-tocopherol methyltransferase	γT3/(γT3+αT3)	S5_200369508	GBS	5	200,369,508	2,479	-1,343	5.08E-05	4.44E-02	0.18	0.11	0.22	251	0.18	0.24	-0.07	1.35	-0.05
Tocochromanol Pathway	GRMZM2G035213	γ-tocopherol methyltransferase	δT/αT	S5_200369508	GBS	5	200,369,508	2,479	-1,343	8.74E-06	3.79E-03	0.18	0.11	0.22	246	0.28	0.35	-0.44	0.00	-0.98
Tocochromanol Pathway	GRMZM2G035213	γ-tocopherol methyltransferase	αT/γT	S5_200369534	GBS	5	200,369,534	2,505	-1,317	5.38E-06	2.34E-03	0.08	0.00	0.12	246	0.25	0.31	-0.07	0.15	-0.36
Tocochromanol Pathway	GRMZM2G035213	γ-tocopherol methyltransferase	αT	S5_200369534	GBS	5	200,369,534	2,505	-1,317	2.74E-06	1.05E-03	0.08	0.00	0.12	251	0.25	0.32	-0.34	0.40	-0.64
Tocochromanol Pathway	GRMZM2G035213	γ-tocopherol methyltransferase	γT/(γT+αT)	S5_200369534	GBS	5	200,369,534	2,505	-1,317	6.52E-05	2.10E-02	0.08	0.00	0.12	251	0.29	0.34	0.10	2.00	0.05
Tocochromanol Pathway	GRMZM2G035213	γ-tocopherol methyltransferase	δT/αT	S5_200369534	GBS	5	200,369,534	2,505	-1,317	6.74E-05	2.16E-02	0.08	0.00	0.12	246	0.28	0.33	0.49	0.00	1.74
Tocochromanol Pathway	GRMZM2G035213	γ-tocopherol methyltransferase	αT/γT	S5_200369625	GBS	5	200,369,625	2,596	-1,226	2.92E-05	8.47E-03	0.30	0.48	0.49	246	0.25	0.30	-0.04	0.15	-0.23
Tocochromanol Pathway	GRMZM2G035213	γ-tocopherol methyltransferase	αT	S5_200369625	GBS	5	200,369,625	2,596	-1,226	6.30E-08	4.28E-05	0.30	0.48	0.49	251	0.25	0.35	-0.24	0.40	-0.49
Tocochromanol Pathway	GRMZM2G035213	γ-tocopherol methyltransferase	γT/(γT+αT)	S5_200369625	GBS	5	200,369,625	2,596	-1,226	7.75E-06	3.65E-03	0.30	0.48	0.49	251	0.29	0.35	0.07	2.00	0.03
Tocochromanol Pathway	GRMZM2G035213	γ-tocopherol methyltransferase	δT/αT	S5_200369625	GBS	5	200,369,625	2,596	-1,226	2.40E-06	1.46E-03	0.30	0.48	0.49	246	0.28	0.35	0.35	0.00	1.14
Tocochromanol Pathway	GRMZM2G035213	γ-tocopherol methyltransferase	αT/γT	S5_200369644	GBS	5	200,369,644	2,615	-1,207	1.40E-05	4.50E-03	0.13	0.12	0.22	246	0.25	0.31	-0.05	0.15	-0.29
Tocochromanol Pathway	GRMZM2G035213	γ-tocopherol methyltransferase	αT	S5_200369644	GBS	5	200,369,644	2,615	-1,207	1.20E-05	3.33E-03	0.13	0.12	0.22	251	0.25	0.31	-0.25	0.40	-0.52
Tocochromanol Pathway	GRMZM2G035213	γ-tocopherol methyltransferase	αT/γT	S5_200369665	GBS	5	200,369,665	2,636	-1,186	1.40E-05	4.50E-03	0.12	0.12	0.22	246	0.25	0.31	0.05	0.15	0.39
Tocochromanol Pathway	GRMZM2G035213	γ-tocopherol methyltransferase	αT	S5_200369665	GBS	5	200,369,665	2,636	-1,186	1.20E-05	3.33E-03	0.12	0.12	0.22	251	0.25	0.31	0.25	0.40	0.76
Tocochromanol Pathway	GRMZM2G035213	γ-tocopherol methyltransferase	αT/γT	S5_200369667	GBS	5	200,369,667	2,638	-1,184	1.90E-07	1.44E-04	0.10	0.00	0.20	246	0.25	0.34	-0.07	0.15	-0.36
Tocochromanol Pathway	GRMZM2G035213	γ-tocopherol methyltransferase	αT	S5_200369667	GBS	5	200,369,667	2,638	-1,184	2.13E-07	1.19E-04	0.10	0.00	0.20	251	0.25	0.34	-0.33	0.40	-0.63
Tocochromanol Pathway	GRMZM2G035213	γ-tocopherol methyltransferase	γT/(γT+αT)	S5_200369667	GBS	5	200,369,667	2,638	-1,184	1.48E-05	6.46E-03	0.10	0.00	0.20	251	0.29	0.35	0.09	2.00	0.05
Tocochromanol Pathway	GRMZM2G035213	γ-tocopherol methyltransferase	δT/αT	S5_200369667	GBS	5	200,369,667	2,638	-1,184	7.99E-06	3.74E-03	0.10	0.00	0.20	246	0.28	0.35	0.48	0.00	1.67
Tocochromanol Pathway	GRMZM2G035213	γ-tocopherol methyltransferase	αT	ss196468364	55K	5	200,370,065	3,036	-786	3.26E-05	8.38E-03	0.35	0.39	0.31	251	0.25	0.31	0.18	0.40	0.51
Tocochromanol Pathway	GRMZM2G035213	γ-tocopherol methyltransferase	αT	PZB02424.2	4K	5	200,370,309	3,280	-542	4.68E-07	2.39E-04	0.17	0.09	0.19	251	0.25	0.34	-0.26	0.40	-0.54
Tocochromanol Pathway	GRMZM2G035213	γ-tocopherol methyltransferase	αT/γT	ss196468368	55K	5	200,371,057	4,028	206	7.53E-06	2.87E-03	0.13	0.13	0.15	246	0.25	0.31	-0.05	0.15	-0.31
Tocochromanol Pathway	GRMZM2G035213	γ-tocopherol methyltransferase	αT	ss196468368	55K	5	200,371,057	4,028	206	7.87E-06	2.53E-03	0.13	0.13	0.15	251	0.25	0.32	-0.27	0.40	-0.55
Tocochromanol Pathway	GRMZM2G035213	γ-tocopherol methyltransferase	γT/(γT+αT)	ss196468368	55K	5	200,371,057	4,028	206	2.65E-05	1.01E-02	0.13	0.13	0.15	251	0.29	0.34	0.09	2.00	0.04
Tocochromanol Pathway	GRMZM2G035213	γ-tocopherol methyltransferase	δT/αT	ss196468368	55K	5	200,371,057	4,028	206	6.12E-05	2.07E-02	0.13	0.13	0.15	246	0.28	0.33	0.43	0.00	1.45
Tocochromanol Pathway	GRMZM2G035213	γ-tocopherol methyltransferase	γT/(γT+αT)	S5_200382117	GBS	5	200,382,117	15,088	11,266	1.39E-04	3.56E-02	0.06	0.07	0.07	251	0.29	0.33	-0.11	2.00	-0.06
Tocochromanol Pathway	GRMZM2G035213	γ-tocopherol methyltransferase	γT/(γT+αT)	S5_200382141	GBS	5	200,382,141	15,112	11,290	1.39E-04	3.56E-02	0.06	0.07	0.07	251	0.29	0.33	-0.11	2.00	-0.06
Tocochromanol Pathway	GRMZM2G035213	γ-tocopherol methyltransferase	αT/γT	S5_200382168	GBS	5	200,382,168	15,139	11,317	2.81E-08	2.86E-05	0.41	0.38	0.40	246	0.25	0.35	-0.05	0.15	-0.29
Tocochromanol Pathway	GRMZM2G035213	γ-tocopherol methyltransferase	αT	S5_200382168	GBS	5	200,382,168	15,139	11,317	3.30E-08	2.52E-05	0.41	0.38	0.40	251	0.25	0.35	-0.25	0.40	-0.51
Tocochromanol Pathway	GRMZM2G035213	γ-tocopherol methyltransferase	γT/(γT+αT)	S5_200382168	GBS	5	200,382,168	15,139	11,317	7.60E-09	6.65E-06	0.41	0.38	0.40	251	0.29	0.39	0.09	2.00	0.05
Tocochromanol Pathway	GRMZM2G035213	γ-tocopherol methyltransferase	δT/αT	S5_200382168	GBS	5	200,382,168	15,139	11,317	5.45E-08	5.52E-05	0.41	0.38	0.40	246	0.28	0.38	0.42	0.00	1.4
Tocochromanol Pathway	GRMZM2G035213	γ-tocopherol methyltransferase	αT	S5_200384686	GBS	5	200,384,686	17,657	13,835	3.29E-05	8.38E-03	0.14	0.31	0.21	251	0.25	0.31	0.24	0.40	0.7
Tocochromanol Pathway	GRMZM2G035213	γ-tocopherol methyltransferase	γT/(γT+αT)	S5_200384686	GBS	5	200,384,686	17,657	13,835	1.48E-04	3.61E-02	0.14	0.31	0.21	251	0.29	0.33	-0.07	2.00	-0.04
Tocochromanol Pathway	GRMZM2G035213	γ-tocopherol methyltransferase	αT	S5_200435003	GBS	5	200,435,003	67,974	64,152	1.92E-04	3.73E-02	0.11	0.12	0.13	251	0.25	0.30	0.25	0.40	0.47
Tocochromanol Pathway	GRMZM2G035213	γ-tocopherol methyltransferase	αT	S5_200435045	GBS	5	200,435,045	68,016	64,194	1.50E-04	3.06E-02	0.12	0.12	0.13	251	0.25	0.30	-0.24	0.40	0.56
Tocochromanol Pathway	GRMZM2G035213	γ-tocopherol methyltransferase	αT/γT	S5_200435108	GBS	5	200,435,108	68,079	64,257	3.12E-10	4.75E-07	0.27	0.44	0.21	246	0.25	0.38	-0.06	0.15	-0.35
Tocochromanol Pathway	GRMZM2G035213	γ-tocopherol methyltransferase	αT3	S5_200435108	GBS	5	200,435,108	68,079	64,257	4.80E-05	4.88E-02	0.27	0.44	0.21	248	0.21	0.27	-0.01	0.05	0.13
Tocochromanol Pathway	GRMZM2G035213	γ-tocopherol methyltransferase	αT	S5_200435108	GBS	5	200,435,108	68,079	64,257	3.30E-10	5.04E-07	0.27	0.44	0.21	251	0.25	0.38	-0.31	0.40	-0.6
Tocochromanol Pathway	GRMZM2G035213	γ-tocopherol methyltransferase	γT/(γT+αT)	S5_200435108	GBS	5	200,435,108	68,079	64,257	1.15E-10	1.76E-07	0.27	0.44	0.21	251	0.29	0.42	0.11	2.00	0.05
Tocochromanol Pathway	GRMZM2G035213	γ-tocopherol methyltransferase	δT/αT	S5_200435108	GBS	5	200,435,108	68,079	64,257	1.95E-09	2.97E-06	0.27	0.44	0.21	246	0.28	0.40	0.50	0.00	1.75
Tocochromanol Pathway	GRMZM2G035213	γ-tocopherol methyltransferase	αT/γT	S5_200435117	GBS	5	200,435,117	68,088	64,266	2.42E-07	1.64E-04	0.26	0.19	0.28	246	0.25	0.33	0.05	0.15	0.38
Tocochromanol Pathway	GRMZM2G035213	γ-tocopherol methyltransferase	αT3/γT3	S5_200435117	GBS	5	200,435,117	68,088	64,266	9.48E-06	2.64E-02	0.26	0.19	0.28	246	0.15	0.22	-0.04	-0.15	0.32
Tocochromanol Pathway	GRMZM2G035213	γ-tocopherol methyltransferase	αT3	S5_200435117	GBS	5	200,435,117	68,088	64,266	1.60E-06	9.78E-03	0.26	0.19	0.28	248	0.21	0.29	0.01	0.05	0.16
Tocochromanol Pathway	GRMZM2G035213	γ-tocopherol methyltransferase	αT	S5_200435117	GBS	5	200,435,117	68,088	64,266	1.52E-07	9.30E-05	0.26	0.19	0.28	251	0.25	0.34	0.25	0.40	0.75
Tocochromanol Pathway	GRMZM2G035213	γ-tocopherol methyltransferase	γT/(γT+αT)	S5_200435117	GBS	5	200,435,117	68,088	64,266	2.25E-07	1.53E-04	0.26	0.19	0.28	251	0.29	0.37	-0.09	2.00	-0.05
Tocochromanol Pathway	GRMZM2G035213	γ-tocopherol methyltransferase	γT3/(γT3+αT3)	S5_200435117	GBS	5	200,435,117	68,088	64,266	4.56E-05	4.44E-02	0.26	0.19	0.28	251	0.18	0.24	-0.06	1.35	-0.04
Tocochromanol Pathway	GRMZM2G035213	γ-tocopherol methyltransferase	δT/αT	S5_200435117	GBS	5	200,435,117	68,088	64,266	6.96E-07	4.70E-04	0.26	0.19	0.28	246	0.28	0.36	-0.41	0.00	-0.92
Tocochromanol Pathway	GRMZM2G035213	γ-tocopherol methyltransferase	αT/γT	ss196468352	55K	5	200,435,300	68,271	64,449	2.56E-08	2.86E-05	0.41	0.39	0.37	246	0.25	0.35	-0.05	0.15	-0.29
Tocochromanol Pathway	GRMZM2G035213	γ-tocopherol methyltransferase	αT3/γT3	ss196468352	55K	5	200,435,300	68,271	64,449	6.70E-06	2.64E-02	0.41	0.39	0.37	246	0.15	0.22	0.04	-0.15	-0.22
Tocochromanol Pathway	GRMZM2G035213	γ-tocopherol methyltransferase	αT	ss196468352	55K	5	200,435,300	68,271	64,449	9.58E-10	1.17E-06	0.41	0.39	0.37	251	0.25	0.38	-0.27	0.40	-0.55
Tocochromanol Pathway	GRMZM2G035213	γ-tocopherol methyltransferase	γT/(γT+αT)	ss196468352	55K	5	200,435,300	68,271	64,449	6.65E-09	6.65E-06	0.41	0.39	0.37	251	0.29	0.39	0.09	2.00	0.05
Tocochromanol Pathway	GRMZM2G035213	γ-tocopherol methyltransferase	γT3/(γT3+αT3)	ss196468352	55K	5	200,435,300	68,271	64,449	2.81E-05	3.69E-02	0.41	0.39	0.37	251	0.18	0.24	0.06	1.35	0.04

Tocochromanol Pathway	GRMZM2G035213	γ-tocopherol methyltransferase	δT/αT	ss196468352	55K	5	200,435,300	68,271	64,449	2.92E-08	3.55E-05	0.41	0.39	0.37	246	0.28	0.38	0.42	0.00	1.42
Tocochromanol Pathway	GRMZM2G035213	γ-tocopherol methyltransferase	αT/γT	S5_200437468	GBS	5	200,437,468	70,439	66,617	7.35E-08	6.39E-05	0.29	0.45	0.24	246	0.25	0.34	-0.05	0.15	-0.3
Tocochromanol Pathway	GRMZM2G035213	γ-tocopherol methyltransferase	αT	S5_200437468	GBS	5	200,437,468	70,439	66,617	1.73E-08	1.58E-05	0.29	0.45	0.24	251	0.25	0.36	-0.27	0.40	-0.54
Tocochromanol Pathway	GRMZM2G035213	γ-tocopherol methyltransferase	γT/(γT+αT)	S5_200437468	GBS	5	200,437,468	70,439	66,617	3.36E-08	2.57E-05	0.29	0.45	0.24	251	0.29	0.38	0.09	2.00	0.05
Tocochromanol Pathway	GRMZM2G035213	γ-tocopherol methyltransferase	δT/αT	S5_200437468	GBS	5	200,437,468	70,439	66,617	1.49E-07	1.29E-04	0.29	0.45	0.24	246	0.28	0.37	0.42	0.00	1.42
Tocochromanol Pathway	GRMZM2G035213	γ-tocopherol methyltransferase	αT/γT	ss196517251	55K	5	200,437,606	70,577	66,755	4.82E-07	2.85E-04	0.47	0.23	0.40	246	0.25	0.33	-0.05	0.15	-0.26
Tocochromanol Pathway	GRMZM2G035213	γ-tocopherol methyltransferase	αT	ss196517251	55K	5	200,437,606	70,577	66,755	1.33E-06	5.80E-04	0.47	0.23	0.40	251	0.25	0.33	-0.21	0.40	-0.45
Tocochromanol Pathway	GRMZM2G035213	γ-tocopherol methyltransferase	γT/(γT+αT)	ss196517251	55K	5	200,437,606	70,577	66,755	3.40E-06	1.73E-03	0.47	0.23	0.40	251	0.29	0.36	0.07	2.00	0.04
Tocochromanol Pathway	GRMZM2G035213	γ-tocopherol methyltransferase	δT/αT	ss196517251	55K	5	200,437,606	70,577	66,755	3.35E-06	1.85E-03	0.47	0.23	0.40	246	0.28	0.35	0.35	0.00	1.16
Tocochromanol Pathway	GRMZM2G035213	γ-tocopherol methyltransferase	αT/γT	S5_200438801	GBS	5	200,438,801	71,772	67,950	6.92E-06	2.81E-03	0.10	0.06	0.13	246	0.25	0.31	0.06	0.15	0.48
Tocochromanol Pathway	GRMZM2G035213	γ-tocopherol methyltransferase	αT	S5_200438801	GBS	5	200,438,801	71,772	67,950	4.48E-06	1.52E-03	0.10	0.06	0.13	251	0.25	0.32	0.31	0.40	0.95
Tocochromanol Pathway	GRMZM2G035213	γ-tocopherol methyltransferase	γT/(γT+αT)	S5_200438801	GBS	5	200,438,801	71,772	67,950	1.81E-04	4.27E-02	0.10	0.06	0.13	251	0.29	0.33	-0.09	2.00	-0.04
Tocochromanol Pathway	GRMZM2G035213	γ-tocopherol methyltransferase	αT	ss196512031	55K	5	200,491,310	124,281	120,459	1.11E-04	2.43E-02	0.40	0.35	0.36	251	0.25	0.30	0.17	0.40	0.48
Tocochromanol Pathway	GRMZM2G035213	γ-tocopherol methyltransferase	γT/(γT+αT)	ss196512031	55K	5	200,491,310	124,281	120,459	1.08E-04	3.00E-02	0.40	0.35	0.36	251	0.29	0.34	-0.06	2.00	-0.03
Tocochromanol Pathway	GRMZM2G035213	γ-tocopherol methyltransferase	δT/αT	ss196512031	55K	5	200,491,310	124,281	120,459	1.13E-05	4.57E-03	0.40	0.35	0.36	246	0.28	0.34	-0.33	0.00	-0.77
Tocochromanol Pathway	GRMZM2G035213	γ-tocopherol methyltransferase	αT	ss196468372	55K	5	200,598,221	231,192	227,370	2.59E-04	4.59E-02	0.25	0.18	0.27	251	0.25	0.30	-0.19	0.40	-0.4
Tocochromanol Pathway	GRMZM2G173358	homogentisic acid geranylgeranyl transferase	γT3/(γT3+αT3)	S9_92313446	GBS	9	92,313,446	-170,103	-173,822	3.01E-05	3.69E-02	0.45	0.27	0.49	251	0.18	0.24	-0.06	1.35	-0.04
Tocochromanol Pathway	GRMZM2G173358	homogentisic acid geranylgeranyl transferase	γT3	S9_92313446	GBS	9	92,313,446	-170,103	-173,822	1.24E-05	1.52E-02	0.45	0.27	0.49	250	0.23	0.29	-0.03	0.10	-0.23
Tocochromanol Pathway	GRMZM2G173358	homogentisic acid geranylgeranyl transferase	Total Tocotrienols	S9_92346116	GBS	9	92,346,116	-137,433	-141,152	1.48E-06	9.08E-03	0.41	0.43	0.44	250	0.23	0.30	0.04	0.15	0.27
Tocochromanol Pathway	GRMZM2G173358	homogentisic acid geranylgeranyl transferase	γT3	S9_92346116	GBS	9	92,346,116	-137,433	-141,152	6.82E-07	1.05E-03	0.41	0.43	0.44	250	0.23	0.31	0.03	0.10	0.34
Tocochromanol Pathway	GRMZM2G173358	homogentisic acid geranylgeranyl transferase	Total Tocopherols/Total Tocotrienols	S9_92548696	GBS	9	92,548,696	65,147	61,428	5.84E-06	1.78E-02	0.39	0.38	0.40	247	0.21	0.28	-0.01	0.05	-0.21
Tocochromanol Pathway	GRMZM2G173358	homogentisic acid geranylgeranyl transferase	γT3	S9_92548696	GBS	9	92,548,696	65,147	61,428	5.89E-05	4.52E-02	0.40	0.38	0.40	250	0.23	0.28	0.03	0.10	0.30
Tocochromanol Pathway	GRMZM2G173358	homogentisic acid geranylgeranyl transferase	Total Tocopherols/Total Tocotrienols	S9_92554465	GBS	9	92,554,465	70,916	67,197	1.52E-06	9.26E-03	0.26	0.00	0.32	247	0.21	0.29	-0.01	0.05	-0.25
Tocochromanol Pathway	GRMZM2G173358	homogentisic acid geranylgeranyl transferase	αT3	S9_92553908	GBS	9	92,553,908	70,359	66,640	1.33E-05	2.70E-02	0.43	0.05	0.38	248	0.21	0.28	0.01	0.05	-0.12
Tocochromanol Pathway	GRMZM2G173358	homogentisic acid geranylgeranyl transferase	Total Tocotrienols	S9_92718671	GBS	9	92,718,671	235,122	231,403	6.46E-05	4.95E-02	0.08	0.00	0.13	250	0.23	0.28	-0.05	0.15	-0.3
Tocochromanol Pathway	GRMZM2G173358	homogentisic acid geranylgeranyl transferase	γT3/(γT3+αT3)	S9_92718671	GBS	9	92,718,671	235,122	231,403	2.36E-05	3.69E-02	0.08	0.00	0.13	251	0.18	0.24	-0.10	1.35	-0.07
Tocochromanol Pathway	GRMZM2G173358	homogentisic acid geranylgeranyl transferase	γT3	S9_92718671	GBS	9	92,718,671	235,122	231,403	6.20E-07	1.05E-03	0.08	0.00	0.13	250	0.23	0.31	-0.05	0.10	-0.41
Tocochromanol Pathway	GRMZM2G173358	homogentisic acid geranylgeranyl transferase	Total Tocotrienols	S9_92718674	GBS	9	92,718,674	235,125	231,406	6.46E-05	4.95E-02	0.08	0.00	0.13	250	0.23	0.28	-0.05	0.15	0.36
Tocochromanol Pathway	GRMZM2G173358	homogentisic acid geranylgeranyl transferase	γT3/(γT3+αT3)	S9_92718674	GBS	9	92,718,674	235,125	231,406	2.36E-05	3.69E-02	0.08	0.00	0.13	251	0.18	0.24	-0.10	1.35	-0.07
Tocochromanol Pathway	GRMZM2G173358	homogentisic acid geranylgeranyl transferase	γT3	S9_92718674	GBS	9	92,718,674	235,125	231,406	6.20E-07	1.05E-03	0.08	0.00	0.13	250	0.23	0.31	-0.05	0.10	-0.41
Tocochromanol Pathway	GRMZM2G173358	homogentisic acid geranylgeranyl transferase	Total Tocotrienols	S9_92718709	GBS	9	92,718,709	235,160	231,441	6.46E-05	4.95E-02	0.08	0.00	0.13	250	0.23	0.28	-0.05	0.15	0.36
Tocochromanol Pathway	GRMZM2G173358	homogentisic acid geranylgeranyl transferase	γT3/(γT3+αT3)	S9_92718709	GBS	9	92,718,709	235,160	231,441	2.36E-05	3.69E-02	0.08	0.00	0.13	251	0.18	0.24	-0.10	1.35	-0.07
Tocochromanol Pathway	GRMZM2G173358	homogentisic acid geranylgeranyl transferase	γT3	S9_92718709	GBS	9	92,718,709	235,160	231,441	6.20E-07	1.05E-03	0.08	0.00	0.13	250	0.23	0.31	-0.05	0.10	-0.41

Table S7d Statistically significant results from the candidate gene association study of 20 tocochromanol grain traits with all five SNPs identified in the multi-locus mixed-model (MLMM) analysis included as covariates. SNPs that were significantly associated with the indicated trait at 5% FDR are shown.

a priori candidate gene pathway	RefGen_v2 Gene ID	RefGen_v2 Annotated Gene Function	Trait	SNP ID	SNP Source	Chr	Position	Distance from Gene ORF Start	Distance from Gene ORF Finish	*P*-value	FDR Adjusted *P*-value	Minor Allele Frequency (MAF)	MAF Tropical (18% of 252 Lines)	MAF Temperate (82% of 252 Lines)	Sample Size	R^2_{LR} from Model without SNP	R^2_{LR} from Model with SNP	Effect Size	Lambda from Box-Cox Procedure	Back-Transformed Effect Estimates
Aromatic Head Group	GRMZM2G437912	prephenate dehydratase	Total Tocotrienols	S2_59013838	GBS	2	59,013,838	-23,405	-25,340	1.62E-05	3.25E-02	0.06	0.40	0.09	250	0.23	0.29	0.06	0.15	0.47
Aromatic Head Group	GRMZM2G437912	prephenate dehydratase	Total Tocotrienols	S2_59013840	GBS	2	59,013,840	-23,403	-25,338	1.62E-05	3.25E-02	0.06	0.40	0.09	250	0.23	0.29	-0.06	0.15	-0.34
Tocochromanol Pathway	GRMZM2G173358	homogentisic acid geranylgeranyl transferase	Total Tocotrienols	S9_92346116	GBS	9	92,346,116	-137,433	-141,152	1.75E-06	1.05E-02	0.41	0.43	0.44	250	0.23	0.31	0.04	0.15	0.30
Tocochromanol Pathway	GRMZM2G173358	homogentisic acid geranylgeranyl transferase	γT3/(γT3+αT3)	S9_92718671	GBS	9	92,718,671	235,122	231,403	8.05E-06	1.61E-02	0.08	0.00	0.13	251	0.22	0.29	-0.10	1.35	-0.08
Tocochromanol Pathway	GRMZM2G173358	homogentisic acid geranylgeranyl transferase	γT3/(γT3+αT3)	S9_92718674	GBS	9	92,718,674	235,125	231,406	8.05E-06	1.61E-02	0.08	0.00	0.13	251	0.22	0.29	-0.10	1.35	-0.08
Tocochromanol Pathway	GRMZM2G173358	homogentisic acid geranylgeranyl transferase	γT3/(γT3+αT3)	S9_92718709	GBS	9	92,718,709	235,160	231,441	8.05E-06	1.61E-02	0.08	0.00	0.13	251	0.22	0.29	-0.10	1.35	-0.08
Aromatic Head Group	GRMZM2G437912	prephenate dehydratase	γT3	S2_59013838	GBS	2	59,013,838	-23,405	-25,340	4.99E-05	3.76E-02	0.06	0.40	0.09	250	0.23	0.29	0.04	0.10	0.48
Aromatic Head Group	GRMZM2G437912	prephenate dehydratase	γT3	S2_59013840	GBS	2	59,013,840	-23,403	-25,338	4.99E-05	3.76E-02	0.06	0.40	0.09	250	0.23	0.29	-0.04	0.10	-0.34
Tocochromanol Pathway	GRMZM2G173358	homogentisic acid geranylgeranyl transferase	γT3	S9_92313446	GBS	9	92,313,446	-170,103	-173,822	1.29E-05	1.56E-02	0.45	0.27	0.49	250	0.23	0.29	-0.03	0.10	-0.26
Tocochromanol Pathway	GRMZM2G173358	homogentisic acid geranylgeranyl transferase	γT3	S9_92346116	GBS	9	92,346,116	-137,433	-141,152	4.41E-07	8.50E-04	0.41	0.43	0.44	250	0.23	0.32	0.03	0.10	0.34
Tocochromanol Pathway	GRMZM2G173358	homogentisic acid geranylgeranyl transferase	Total Tocopherols/Total Tocotrienols	S9_92548696	GBS	9	92,548,696	65,147	61,428	1.11E-05	3.33E-02	0.39	0.38	0.40	247	0.22	0.29	-0.01	0.05	-0.21
Tocochromanol Pathway	GRMZM2G173358	homogentisic acid geranylgeranyl transferase	γT3	S9_92548696	GBS	9	92,548,696	65,147	61,428	4.13E-05	3.76E-02	0.40	0.38	0.40	250	0.23	0.29	0.03	0.10	0.34
Tocochromanol Pathway	GRMZM2G173358	homogentisic acid geranylgeranyl transferase	Total Tocopherols/Total Tocotrienols	S9_92554465	GBS	9	92,554,465	70,916	67,197	3.14E-06	1.88E-02	0.26	0.00	0.32	247	0.22	0.30	-0.01	0.05	-0.24
Tocochromanol Pathway	GRMZM2G173358	homogentisic acid geranylgeranyl transferase	γT3	S9_92718671	GBS	9	92,718,671	235,122	231,403	5.65E-07	8.50E-04	0.08	0.00	0.13	250	0.23	0.32	-0.05	0.10	-0.40
Tocochromanol Pathway	GRMZM2G173358	homogentisic acid geranylgeranyl transferase	γT3	S9_92718674	GBS	9	92,718,674	235,125	231,406	5.65E-07	8.50E-04	0.08	0.00	0.13	250	0.23	0.32	-0.05	0.10	-0.40
Tocochromanol Pathway	GRMZM2G173358	homogentisic acid geranylgeranyl transferase	γT3	S9_92718709	GBS	9	92,718,709	235,160	231,441	5.65E-07	8.50E-04	0.08	0.00	0.13	250	0.23	0.32	-0.05	0.10	-0.40
Tocochromanol Pathway	GRMZM2G173358	homogentisic acid geranylgeranyl transferase	γT3	ss196492047	55K	9	92,344,750	-138,799	-142,518	5.80E-05	3.88E-02	0.40	0.50	0.40	250	0.23	0.29	-0.02	0.10	-0.18
