# Supplementary material for: Genome-Wide Association Study and Pathway-Level Analysis of Tocochromanol Levels in Maize Grain
Source: G3 (Bethesda). 2013 Aug 1;3(8):1287–99. doi: 10.1534/g3.113.006148 (PMC3737168; doi:10.1534/g3.113.006148)
Supplement: Supporting Information [file supp_g3.113.006148_FigureS1.pdf]

Figure S1 Genome wide association study of 20 tocochromanol grain traits. Manhattan Plots (Gibson, 2010) of association results from a unified mixed model analysis of each tocochromanol grain trait. Negative $\log_{10}$-transformed *P*-values (y-axis) from a GWAS are plotted against physical position (B73 RefGen_v2) on each of 10 chromosomes. Chromosomes are alternatingly colored.

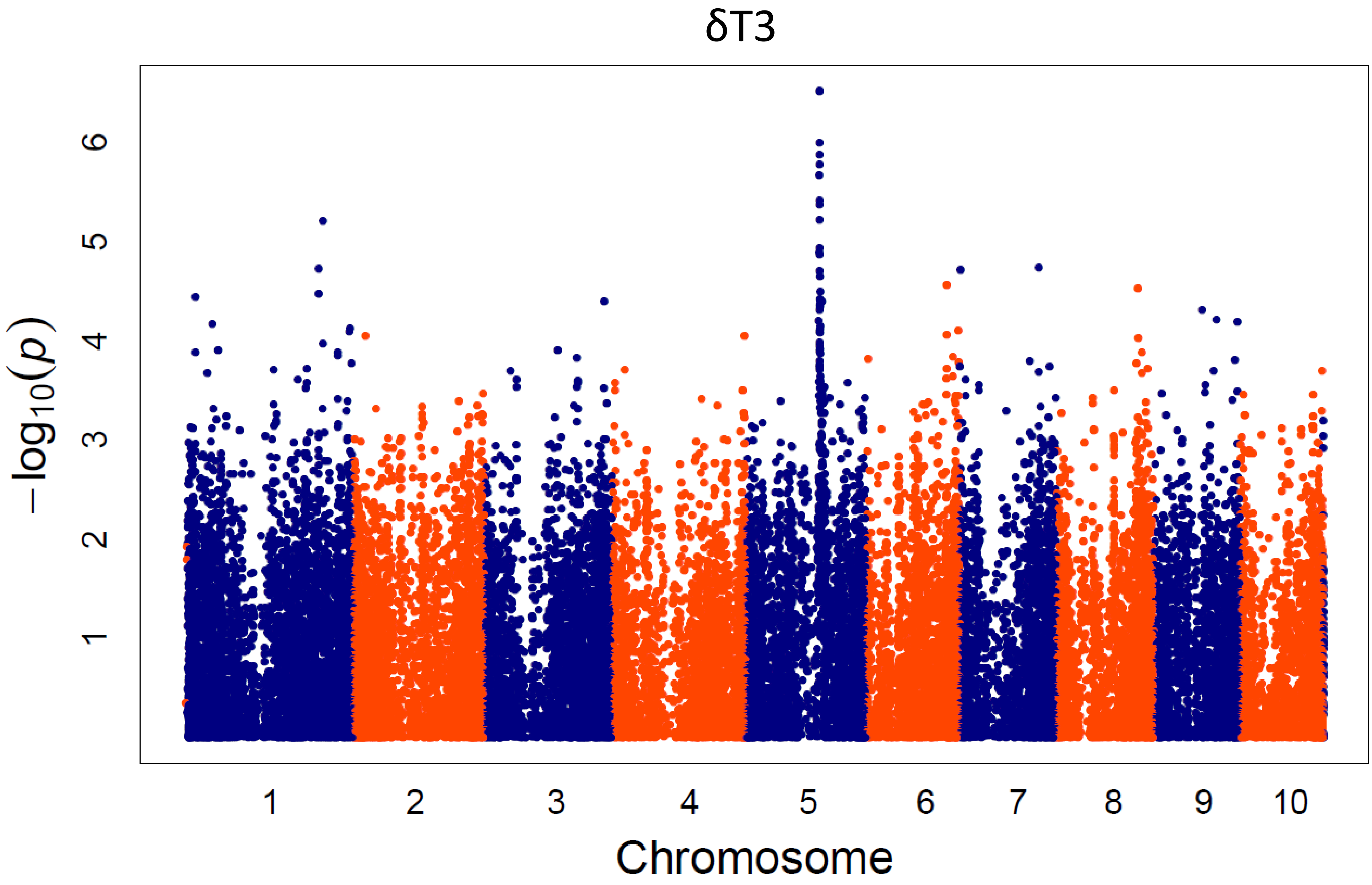
δT3
6
5
4
3
2
1
−log10(p)
1
2
3
4
5
6
7
8
9
10
Chromosome

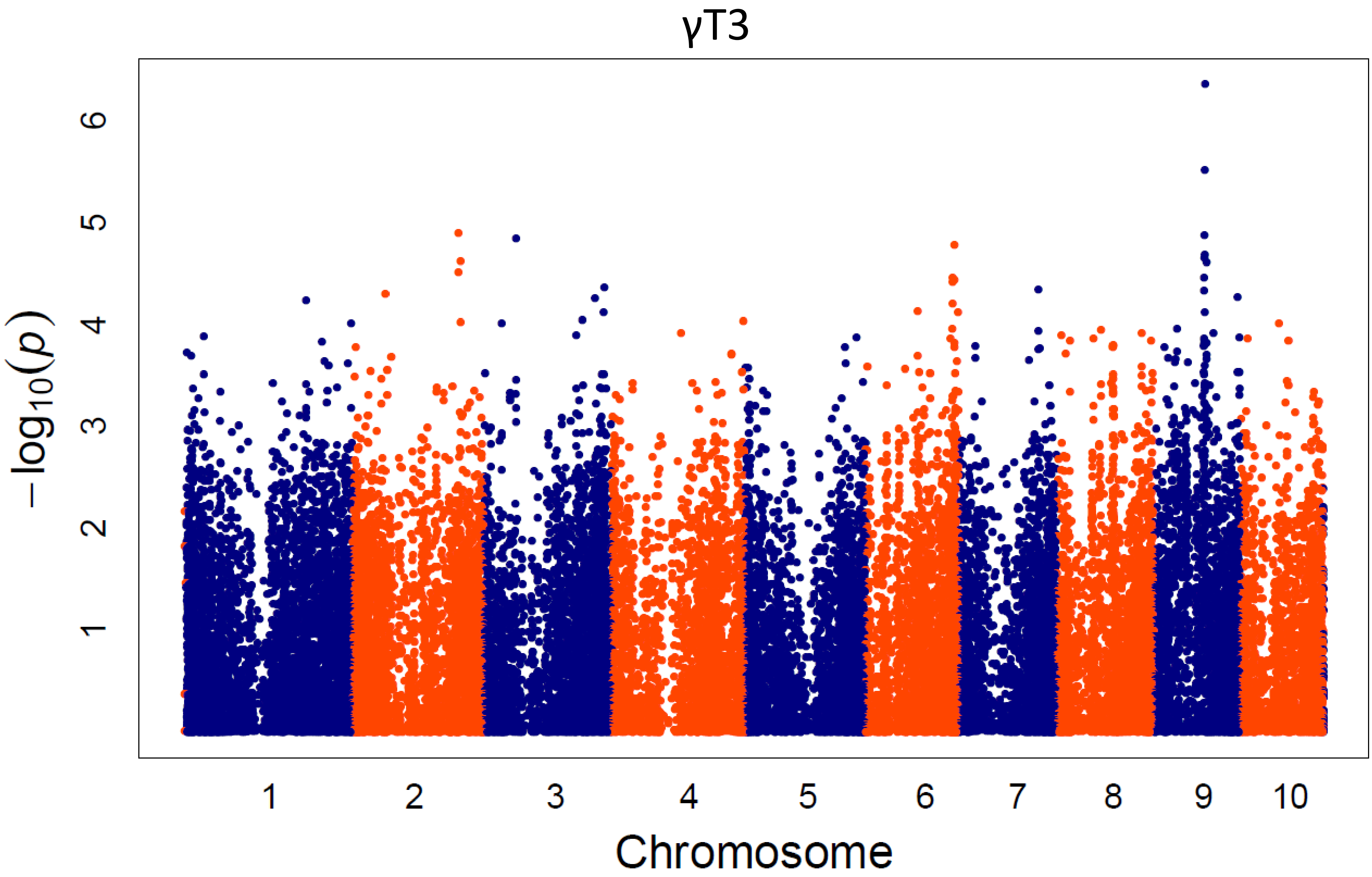
γT3
−log10(p)
1
2
3
4
5
6
1
2
3
4
5
6
7
8
9
10
Chromosome

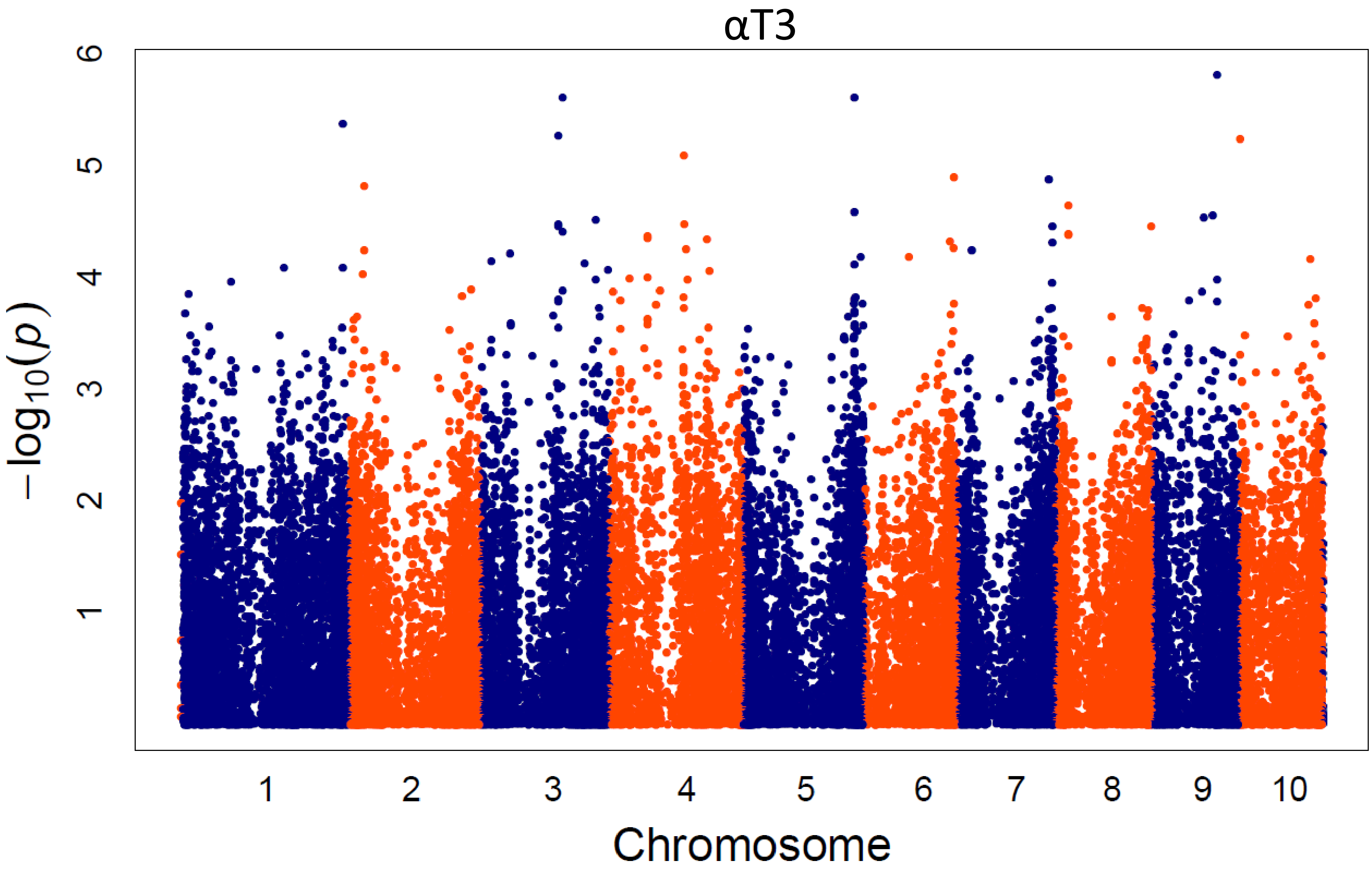

αT3
−log10(p)
6
5
4
3
2
1
1
2
3
4
5
6
7
8
9
10
Chromosome

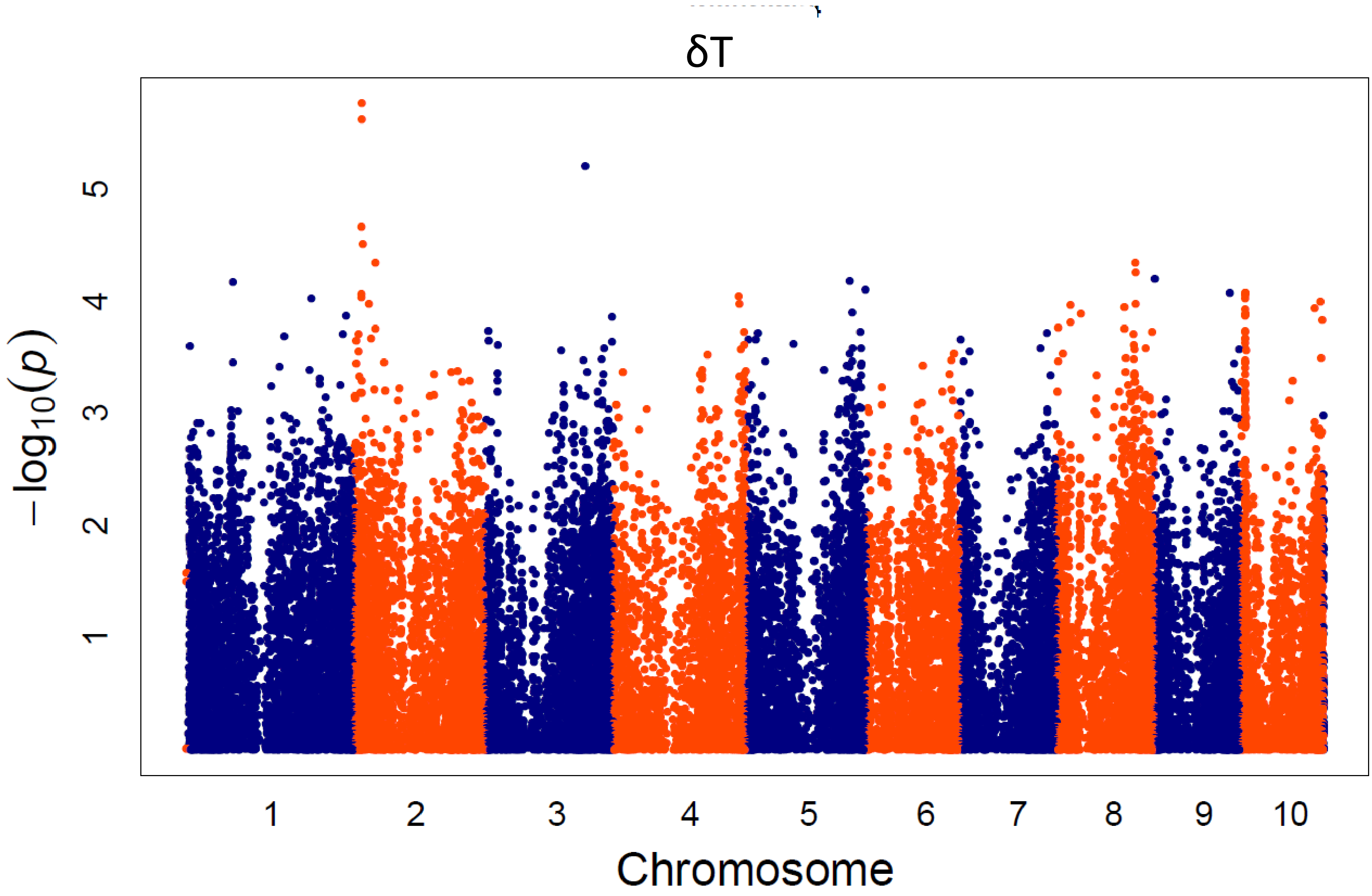
δT
−log10(p)
1
2
3
4
5
1
2
3
4
5
6
7
8
9
10
Chromosome

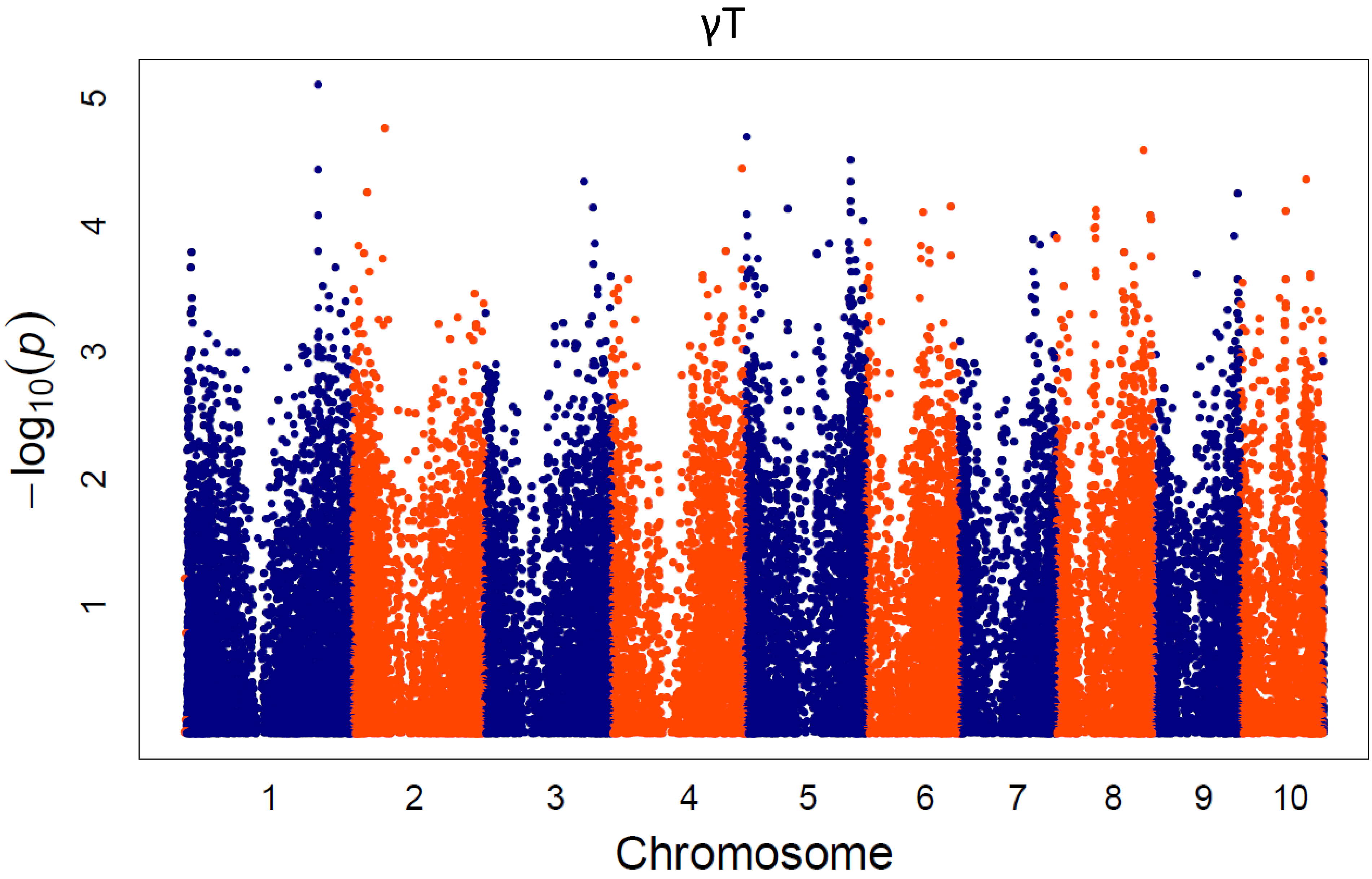
γT
5
4
3
2
1
−log10(p)
1
2
3
4
5
6
7
8
9
10
Chromosome

αT

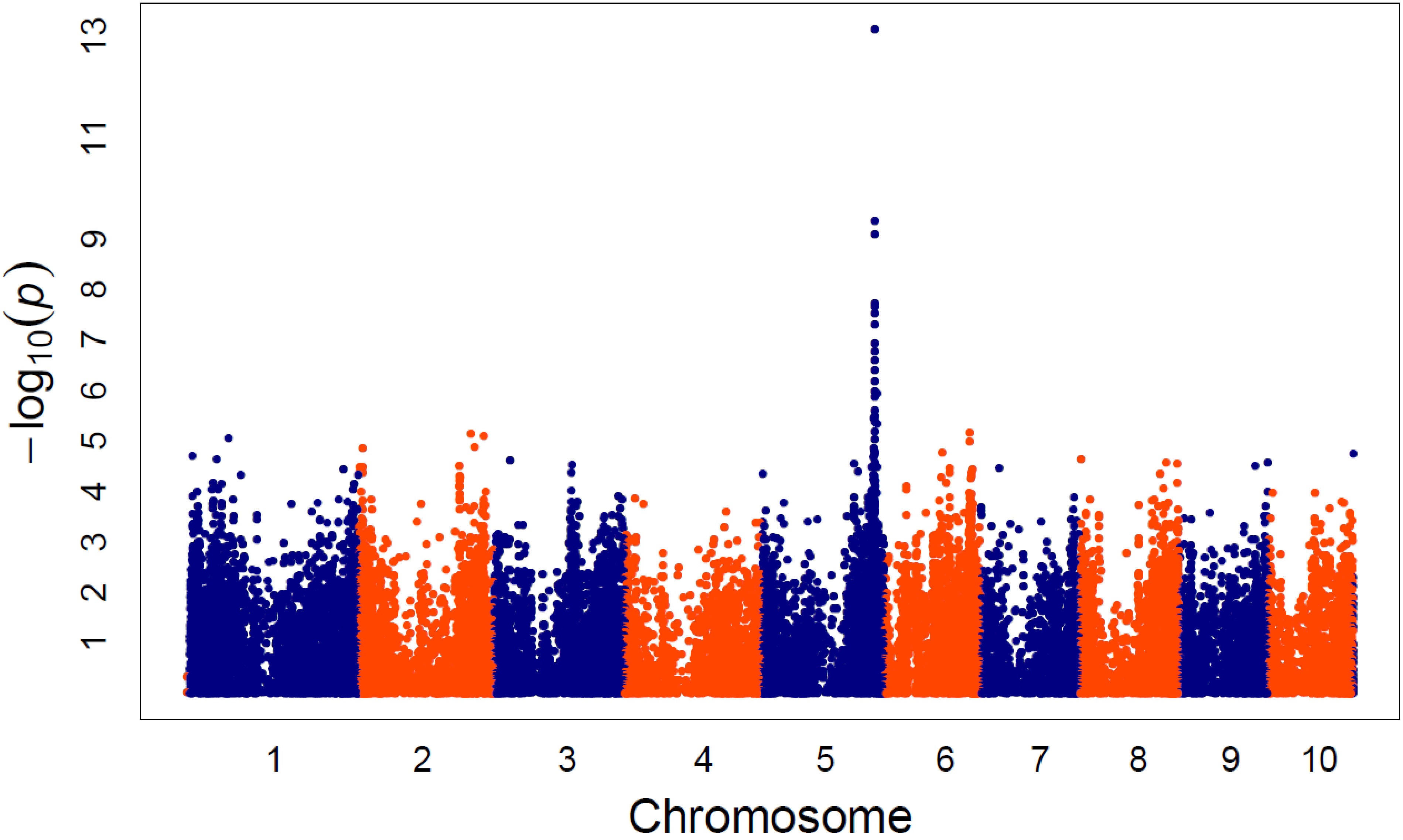

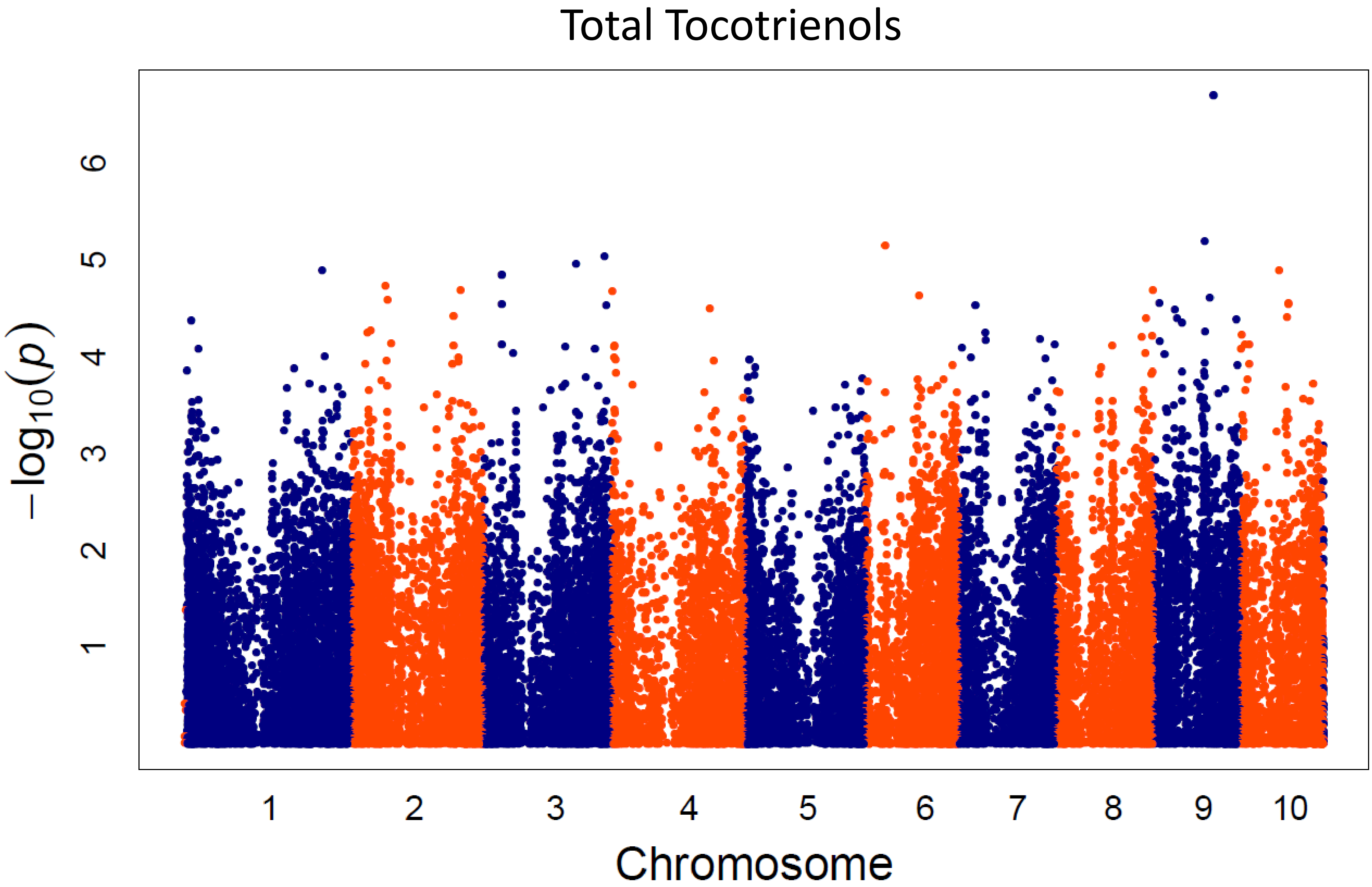
Total Tocotrienols
−log10(p)
1
2
3
4
5
6
1
2
3
4
5
6
7
8
9
10
Chromosome

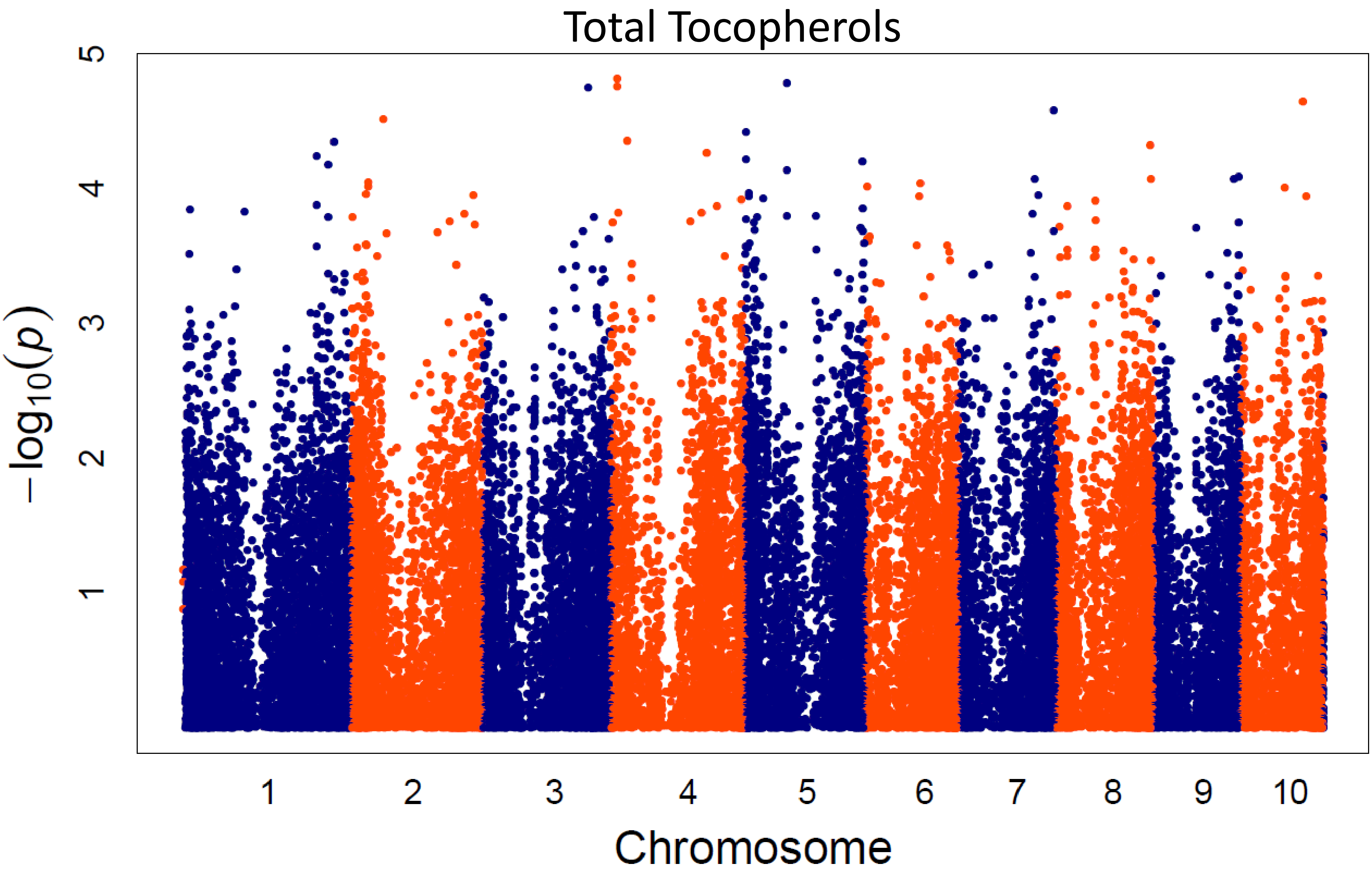

Total Tocopherols
5
4
3
2
1
$-\log_{10}(p)$
1
2
3
4
5
6
7
8
9
10
Chromosome

Total Tocopherols/Total Tocotrienols

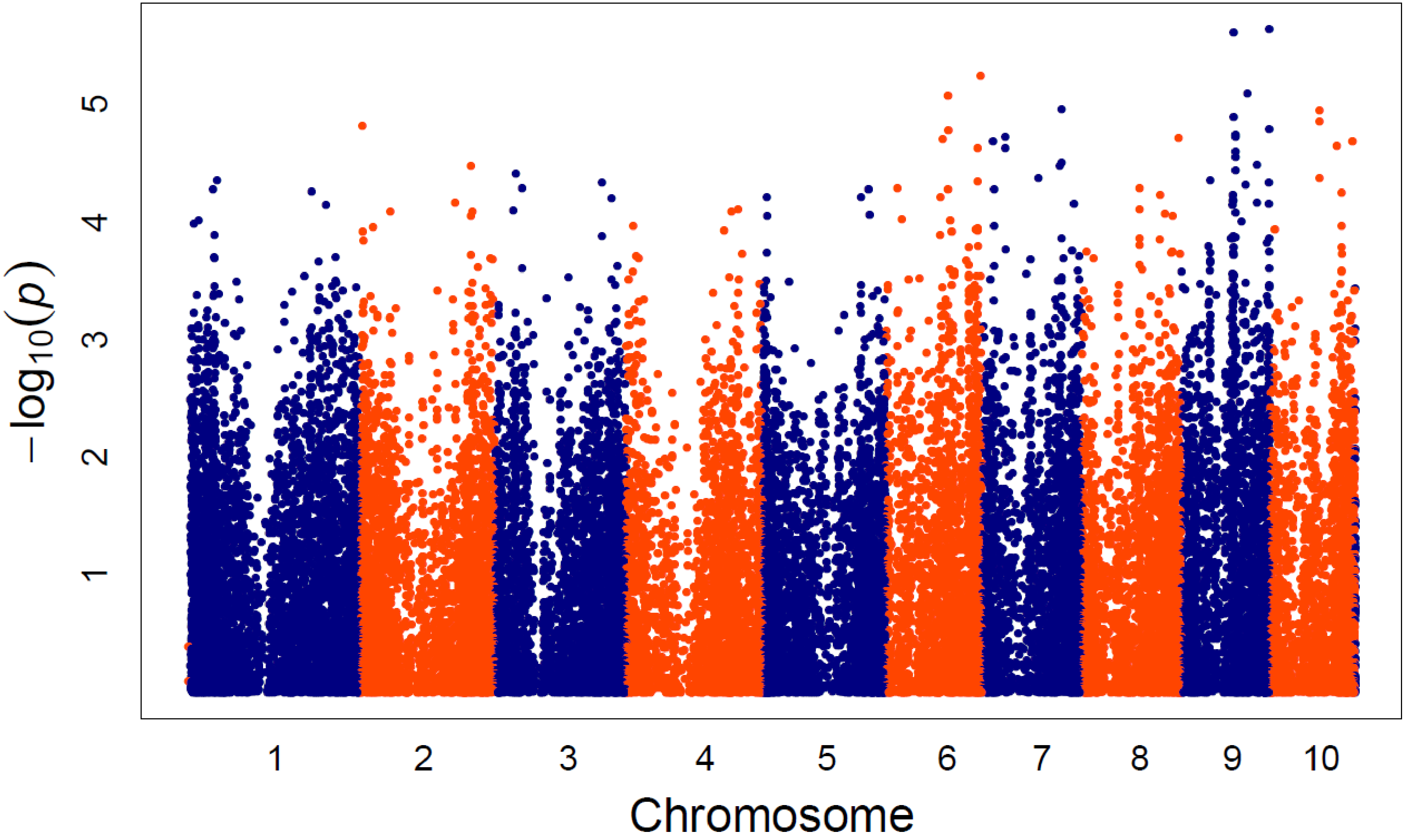

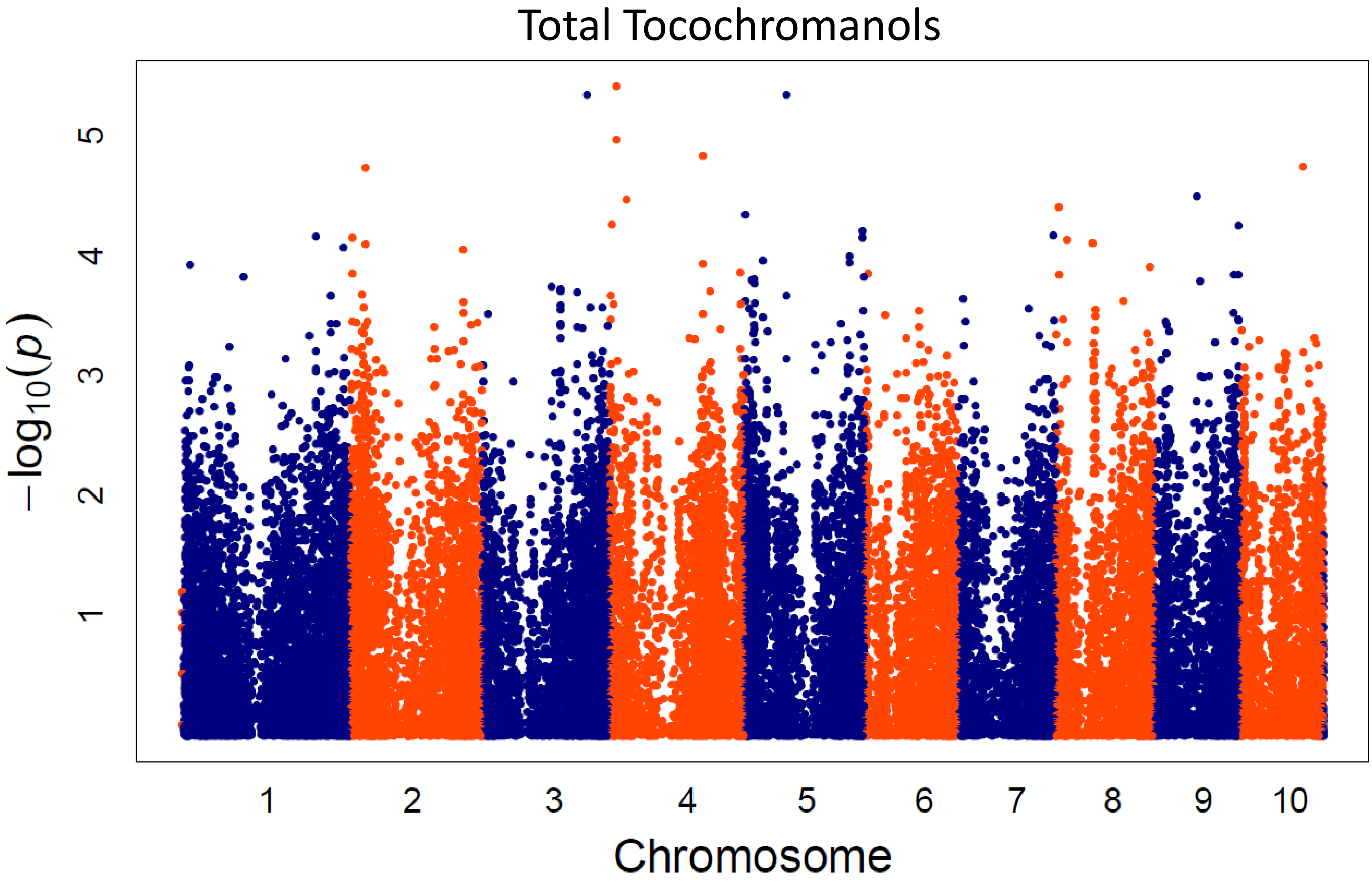
Total Tocochromanols
−log10(p)
1
2
3
4
5
1
2
3
4
5
6
7
8
9
10
Chromosome

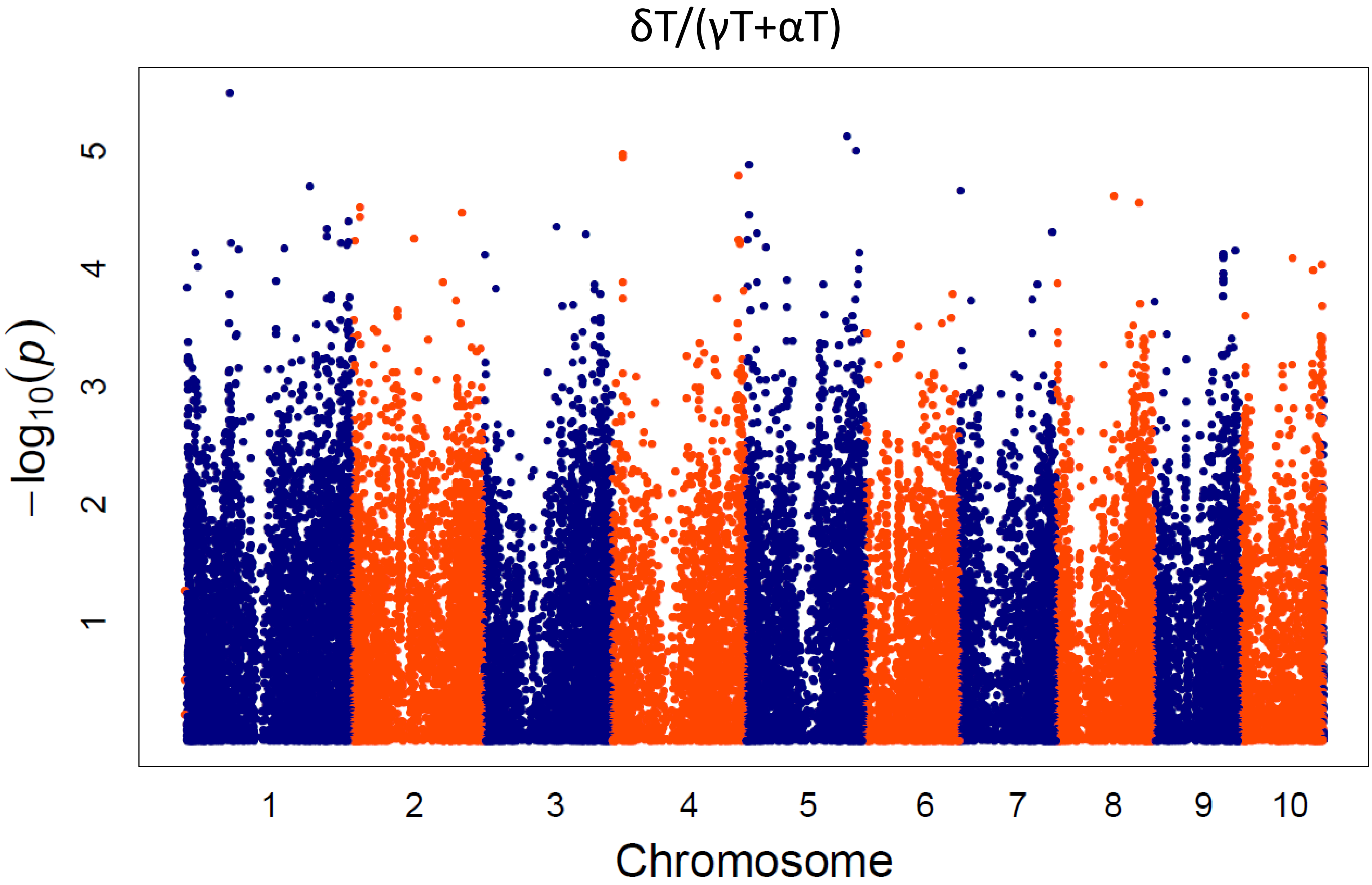
δT/(γT+αT)
−log10(p)
1
2
3
4
5
1
2
3
4
5
6
7
8
9
10
Chromosome

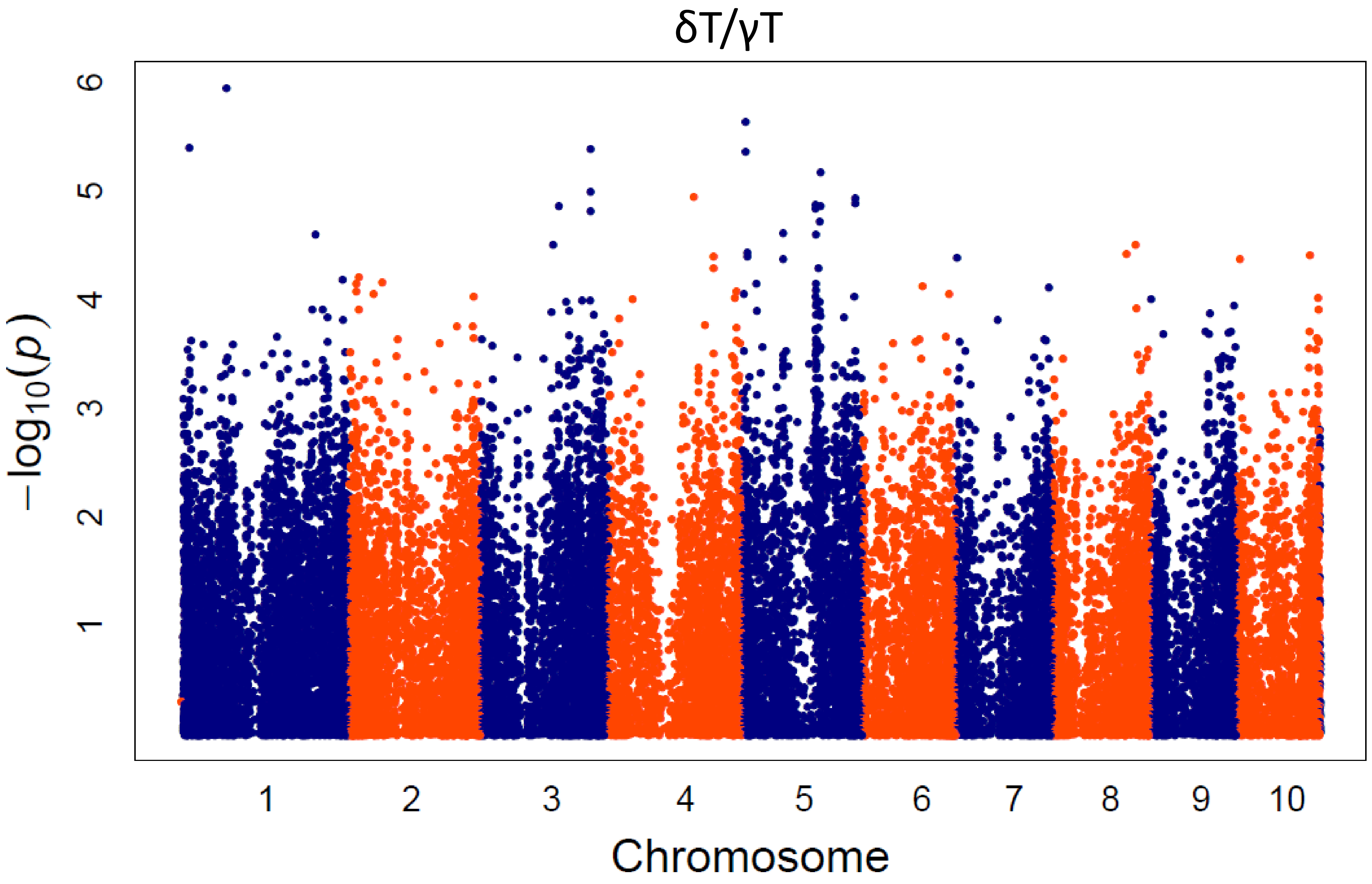
δT/γT
6
5
4
3
2
1
−log10(p)
1
2
3
4
5
6
7
8
9
10
Chromosome

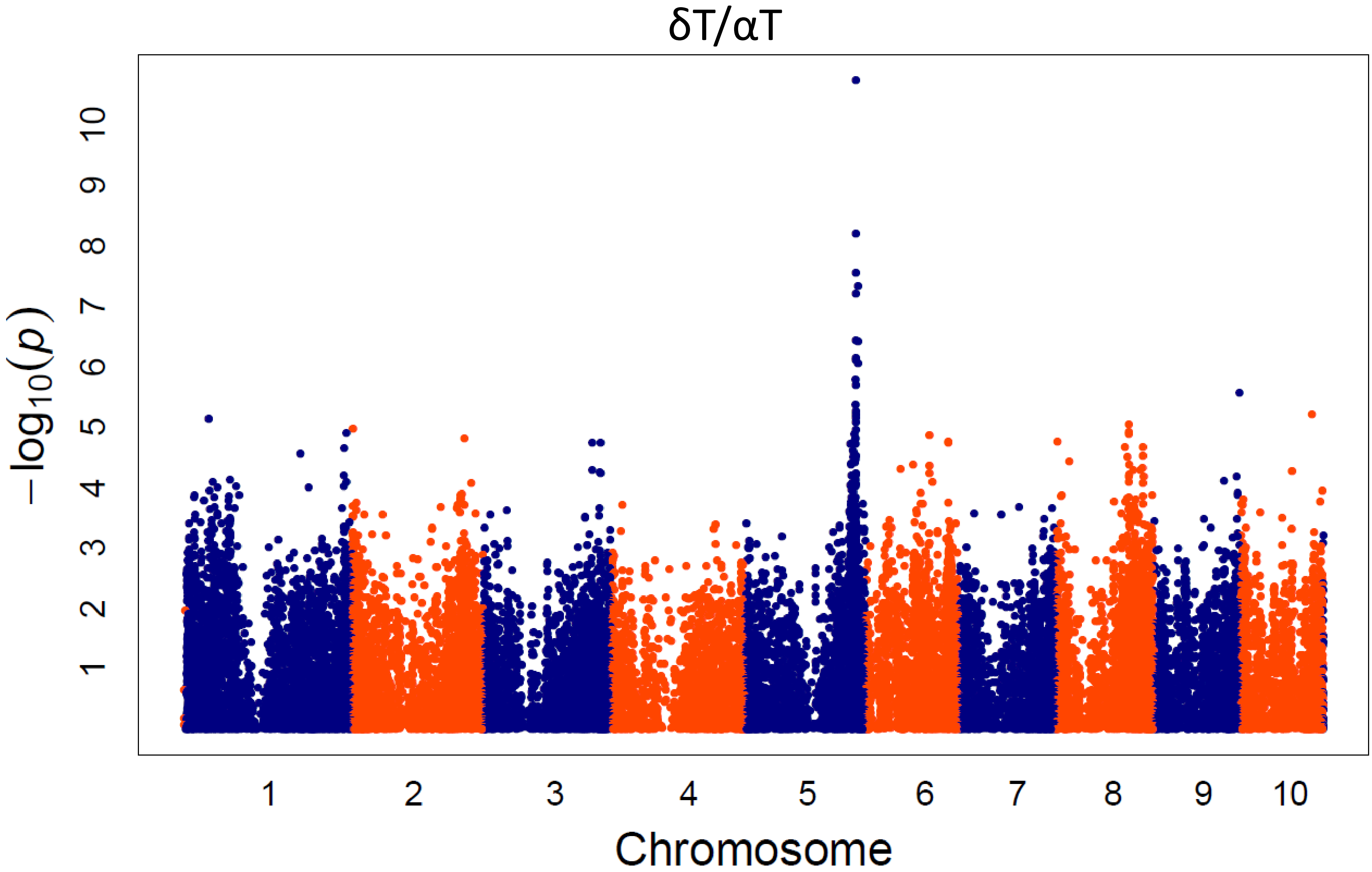
δT/αT
−log10(p)
1
2
3
4
5
6
7
8
9
10
1
2
3
4
5
6
7
8
9
10
Chromosome

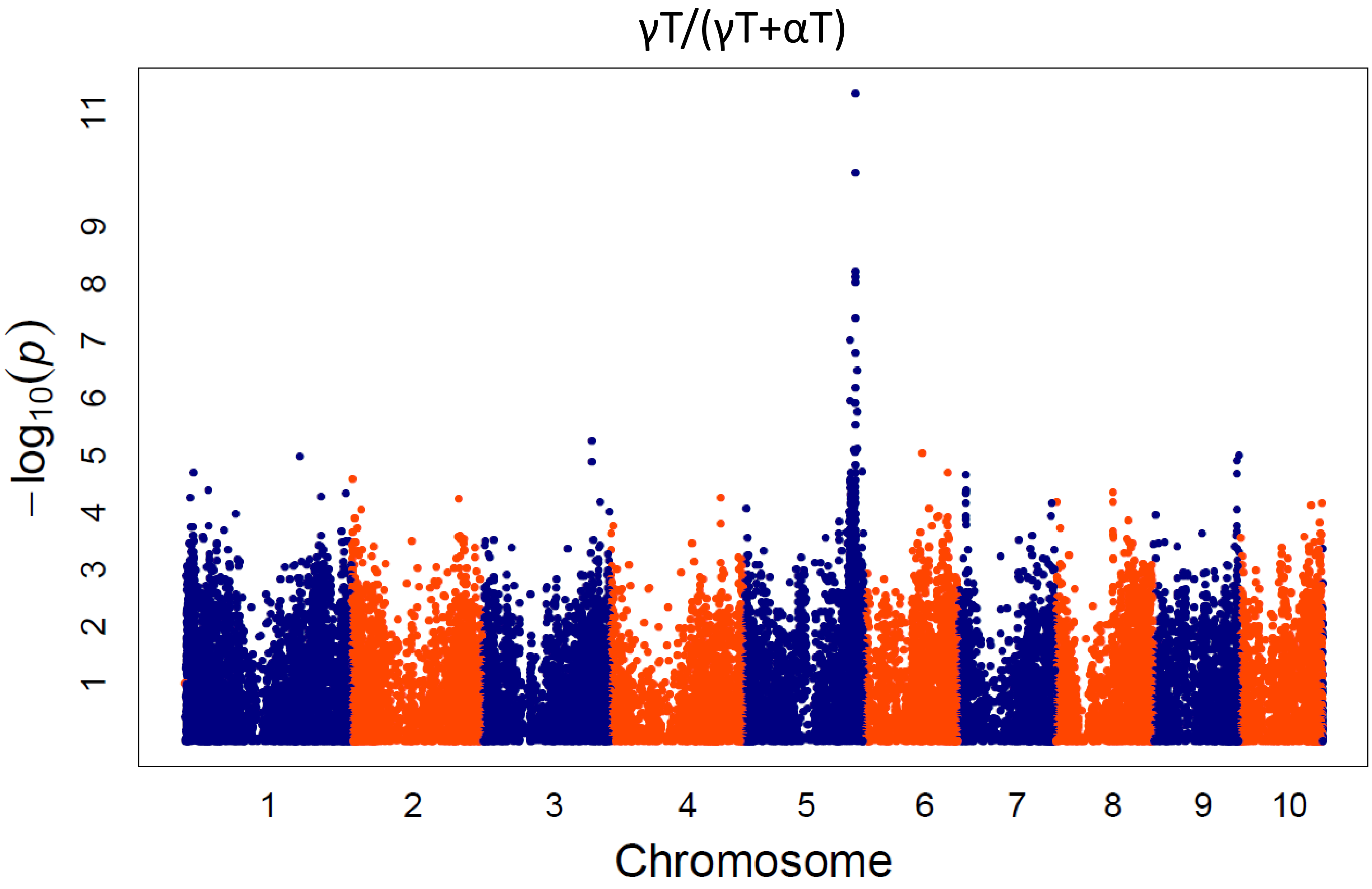
γT/(γT+αT)
−log10(p)
1
2
3
4
5
6
7
8
9
11
1
2
3
4
5
6
7
8
9
10
Chromosome

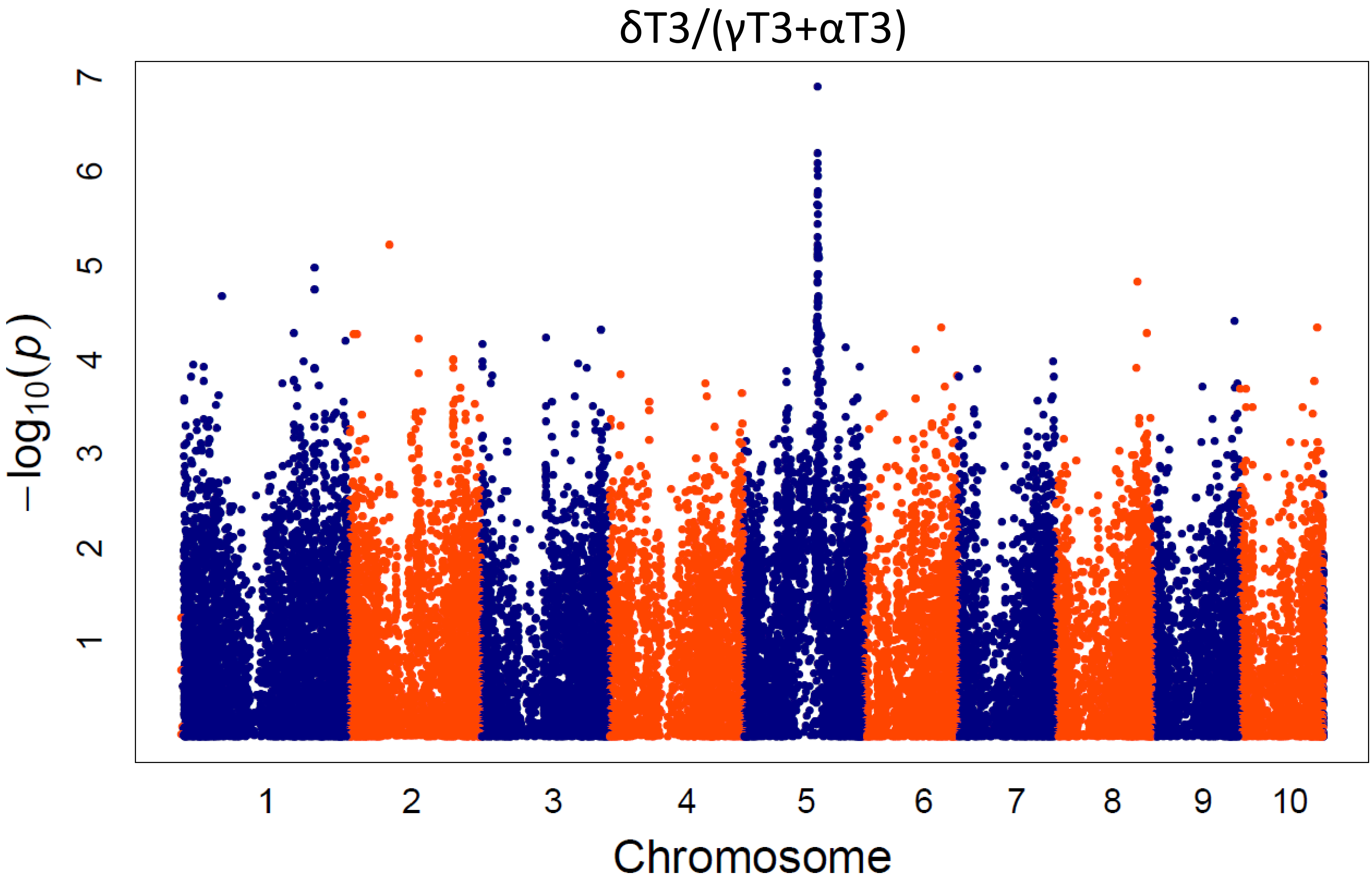
δT3/(γT3+αT3)
−log10(p)
1
2
3
4
5
6
7
1
2
3
4
5
6
7
8
9
10
Chromosome

δT3/γT3

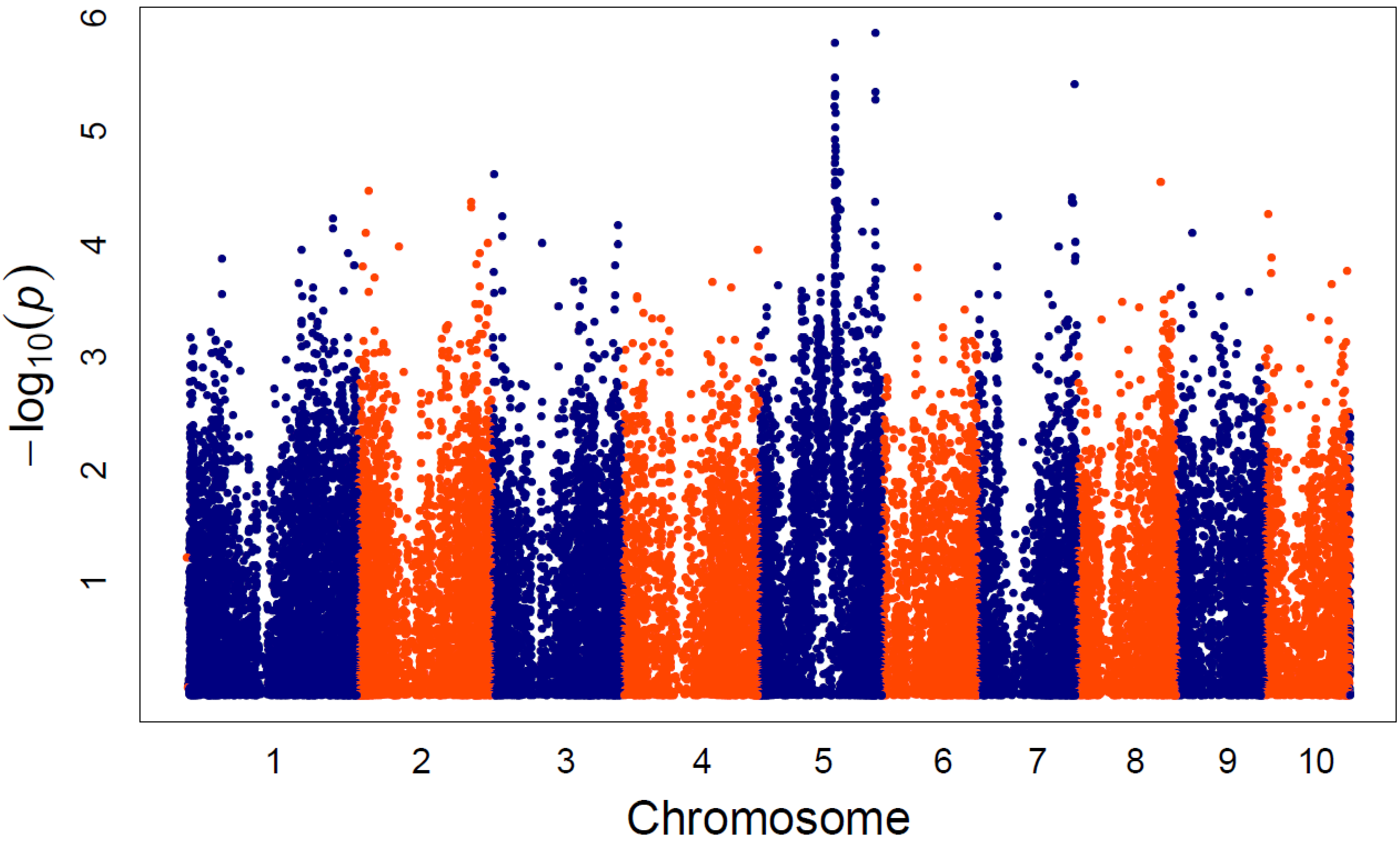

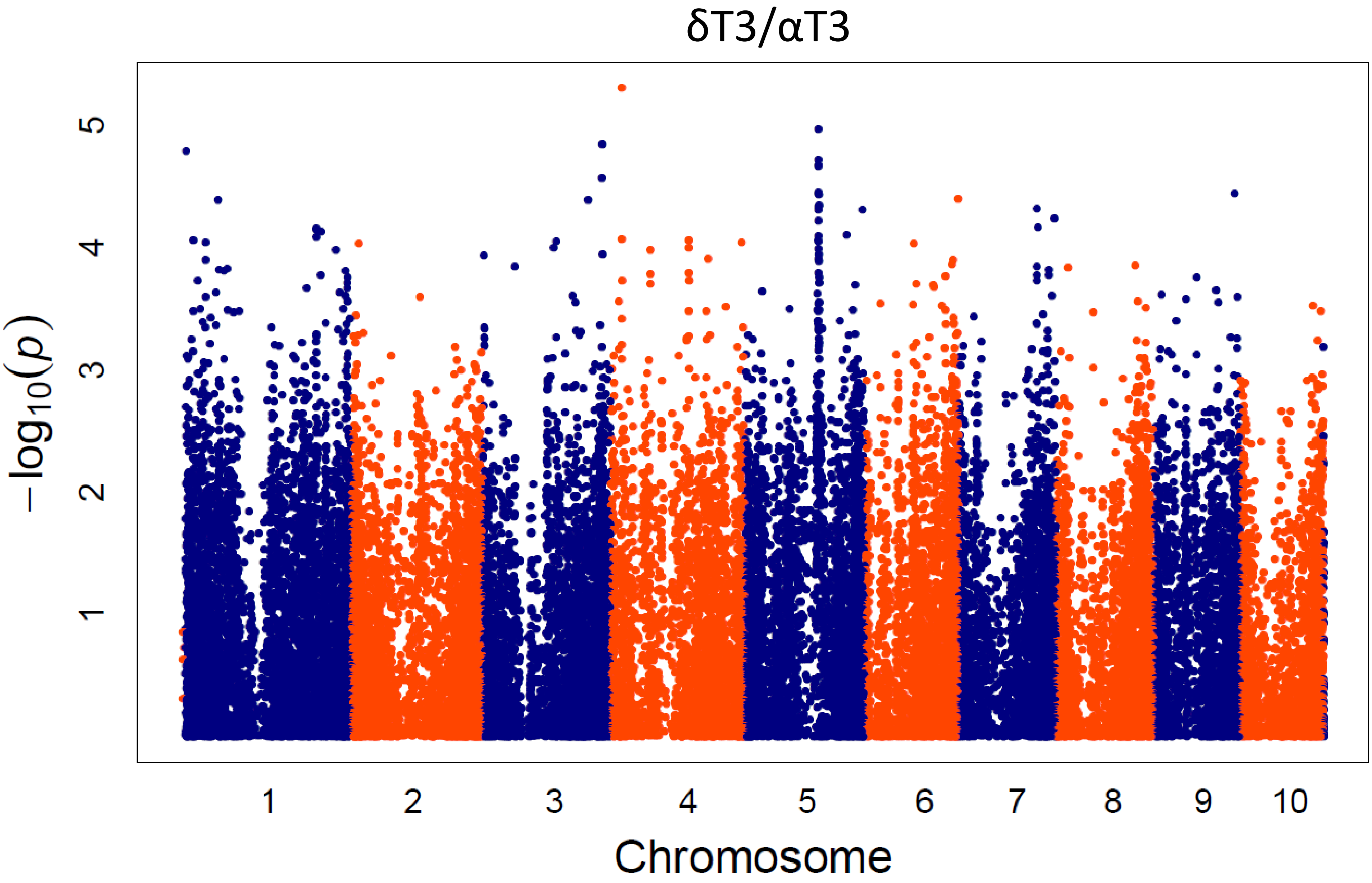
δT3/αT3
−log10(p)
5
4
3
2
1
1
2
3
4
5
6
7
8
9
10
Chromosome

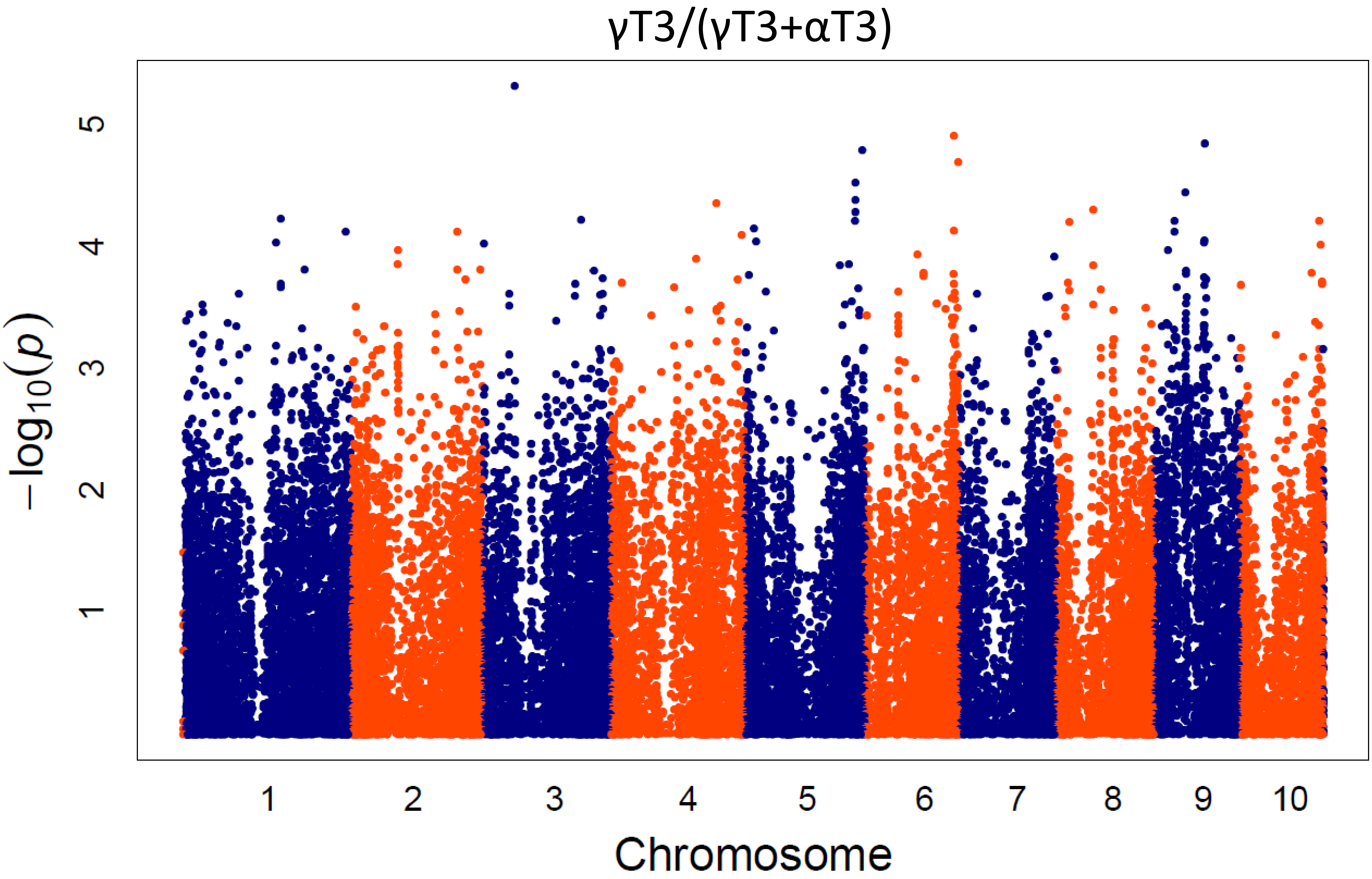
γT3/(γT3+αT3)
−log10(p)
5
4
3
2
1
1
2
3
4
5
6
7
8
9
10
Chromosome

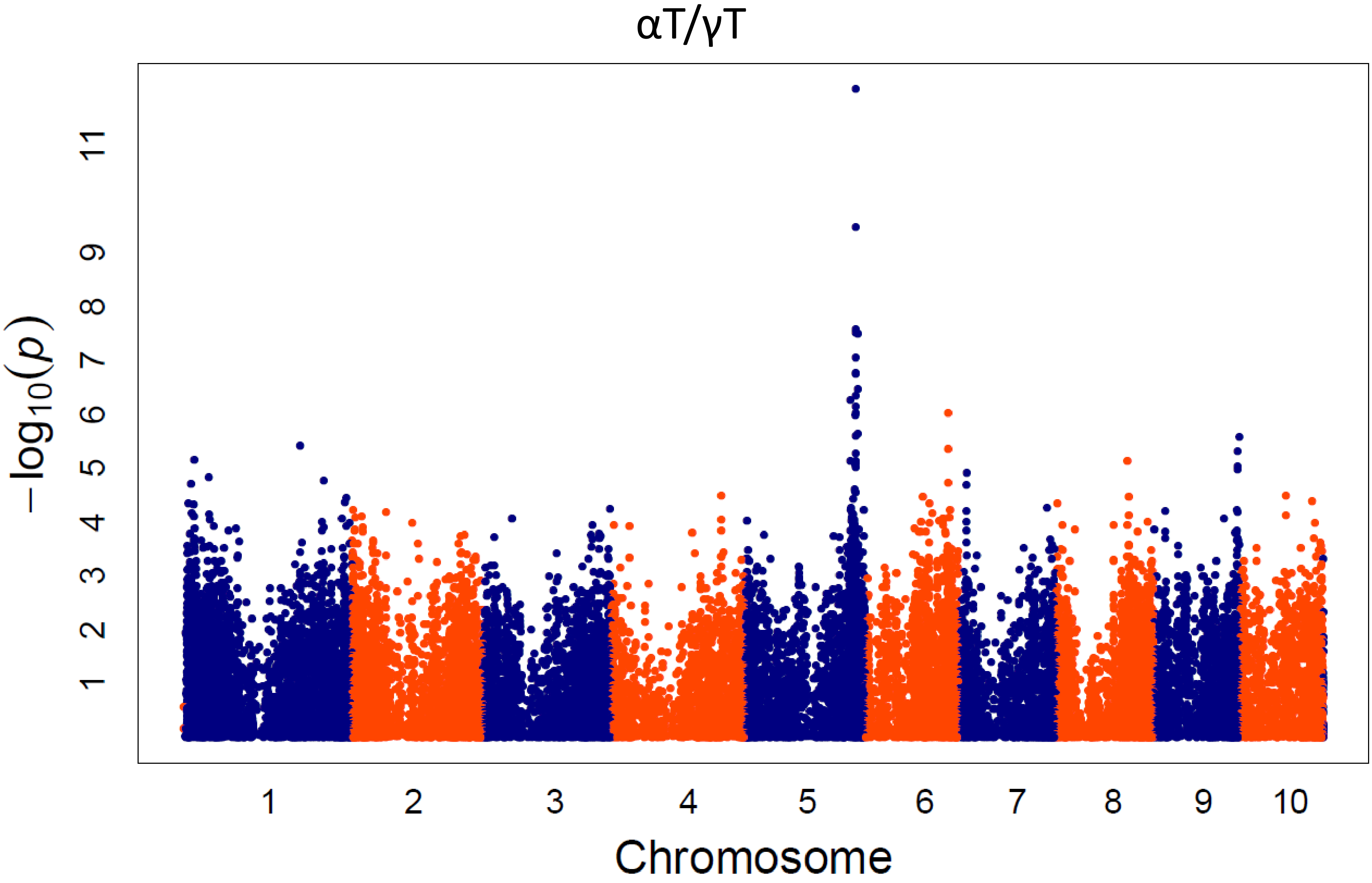
αT/γT
−log10(p)
1
2
3
4
5
6
7
8
9
11
1
2
3
4
5
6
7
8
9
10
Chromosome

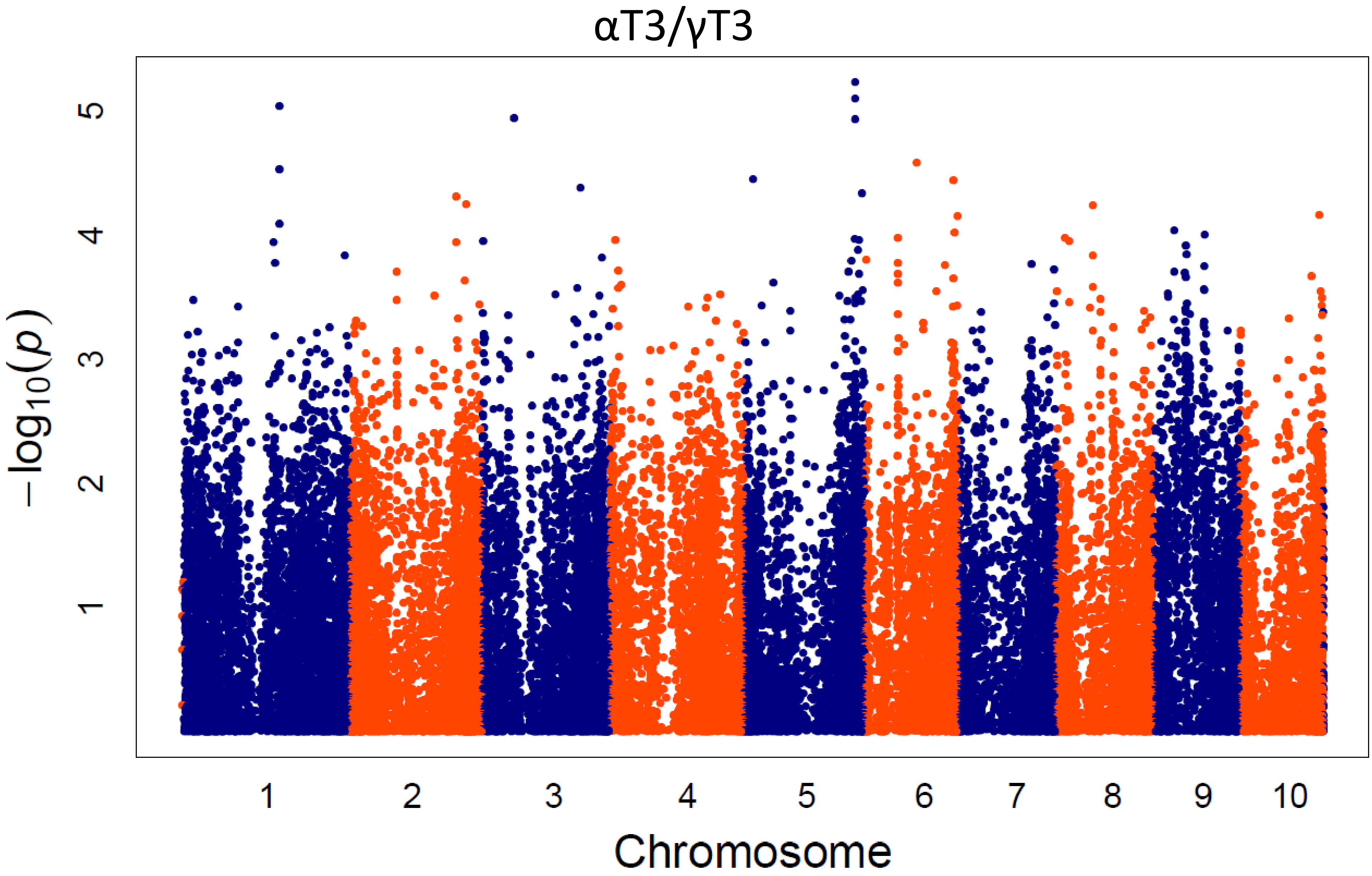
αT3/γT3
−log10(p)
5
4
3
2
1
1
2
3
4
5
6
7
8
9
10
Chromosome
