# Supplementary material for: Genome-Wide Association Study and Pathway-Level Analysis of Tocochromanol Levels in Maize Grain
Source: G3 (Bethesda). 2013 Aug 1;3(8):1287–99. doi: 10.1534/g3.113.006148 (PMC3737168; doi:10.1534/g3.113.006148)
Supplement: Supporting Information [file supp_g3.113.006148_FigureS3.pdf]

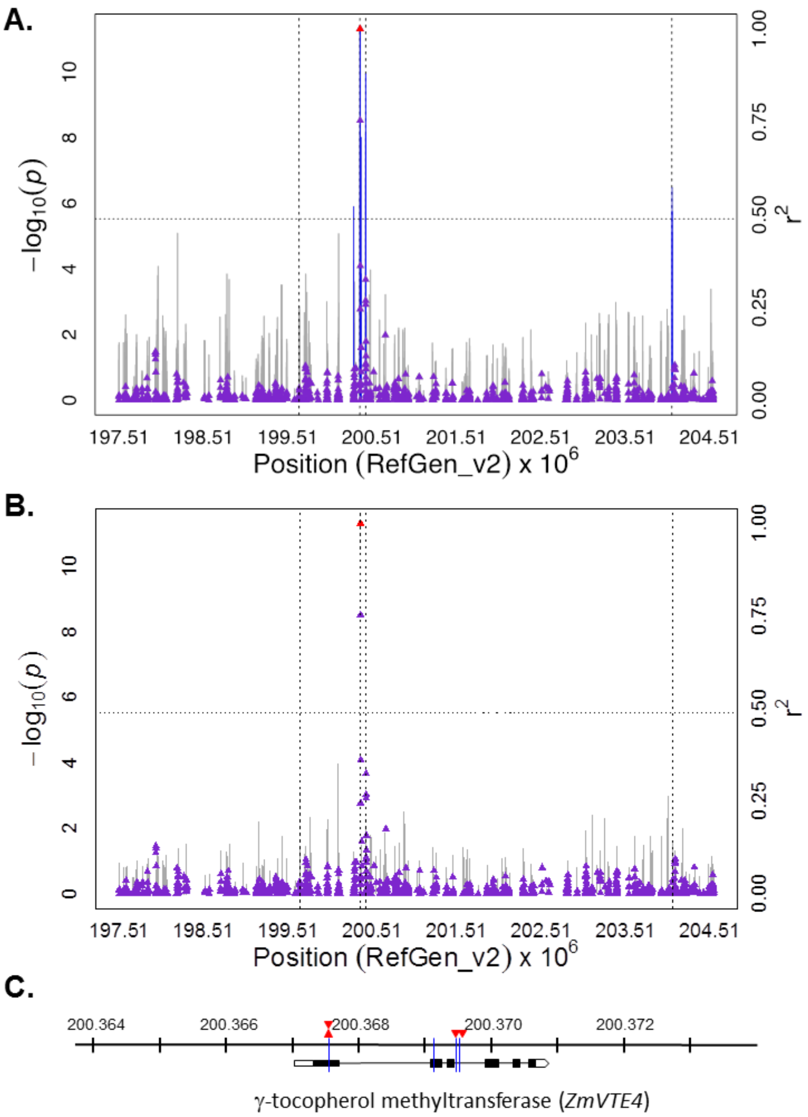

Figure S3 Genome-wide association study (GWAS) for the ratio of γ- to (γ- + α-tocopherols) [γT/(γT+αT)] in maize grain. (A) Scatter plot of association results from a unified mixed model analysis of γT/(γT+αT) and linkage disequilibrium (LD) estimates (r^2) across the *ZmVTE4* chromosome region. Negative $\log_{10}$-transformed *P*-values (left y-axis) from a GWAS for γT/(γT+αT) and r^2 values (right y-axis) are plotted against physical position (B73 RefGen_v2) for a 7 Mb region on chromosome 5 that encompasses *ZmVTE4*. The blue vertical lines are $-\log_{10}$ *P*-values for SNPs that are statistically significant for γT/(γT+αT) at 5% false discovery rate (FDR), while the gray vertical lines are $-\log_{10}$ *P*-values for SNPs that are non-significant at 5% FDR. Triangles are the r^2 values of each SNP relative to the peak SNP (indicated in red) at 200,367,532 bp. The black horizontal dashed line indicates the $-\log_{10}$ *P*-value of the least statistically significant SNP at 5% FDR. The black vertical dashed lines indicate the positions of four genes (from left to right): a WYRKY transcription factor (GRMZM5G823157), *ZmVTE4* (GRMZM2G035213), a pentatricopeptide repeat-containing protein (GRMZM2G325019), and an amino acid permease (GRMZM2G161641). (B) Scatter plot of association results from a conditional unified mixed model analysis of γT/(γT+αT) and LD estimates (r^2) across the *ZmVTE4* chromosome region, as in (A). The three SNPs (ss196416269, S5_200369534, and S5_200369481) from the optimal multi-locus mixed model (MLMM) model were included as covariates in the unified mixed model to control for the *ZmVTE4* effect. (C) Gene model diagram for *ZmVTE4* with γT/(γT+αT) associated SNPs. Blue vertical lines indicate the physical position (RefGen_v2) of SNPs within +/- 3 kb of the open reading frame start or stop position for *ZmVTE4* that are significantly associated with γT/(γT+αT) at 5% FDR. Significant SNPs at 10% FDR are shown as gray vertical lines. The peak SNP is indicated by a red triangle, while the three SNPs included in the optimal MLMM model are indicated by inverted red triangles.
