# Supplementary material for: Genome-Wide Association Study and Pathway-Level Analysis of Tocochromanol Levels in Maize Grain
Source: G3 (Bethesda). 2013 Aug 1;3(8):1287–99. doi: 10.1534/g3.113.006148 (PMC3737168; doi:10.1534/g3.113.006148)
Supplement: Supporting Information [file supp_g3.113.006148_FigureS5.pdf]

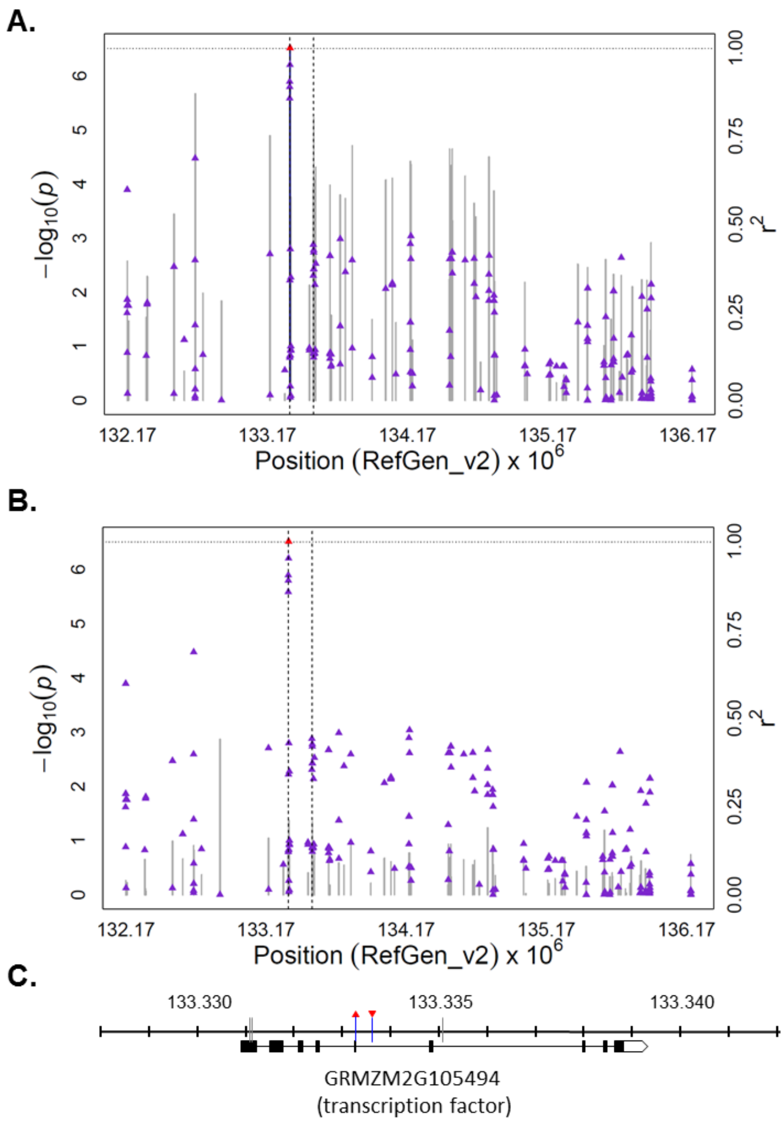

Figure S5 Genome-wide association study (GWAS) for δ-tocotrienol (δT3) in maize grain. (A) Scatter plot of association results from a unified mixed model analysis of δT3 and linkage disequilibrium (LD) estimates (r^2) across the *ZmVTE1* chromosome region. Negative $\log_{10}$-transformed *P*-values (left y-axis) from a GWAS for δT3 and r^2 values (right y-axis) are plotted against physical position (B73 RefGen_v2) for a 4 Mb region on chromosome 5 that encompasses *ZmVTE1*. The blue vertical lines are –$\log_{10}$ *P*-values for SNPs that are statistically significant for δT3 at 5% false discovery rate (FDR), while the gray vertical lines are –$\log_{10}$ *P*-values for SNPs that are non-significant at 5% FDR. Triangles are the r^2 values of each SNP relative to the peak SNP (indicated in red) at 133,333,397 bp. The black horizontal dashed line indicates the –$\log_{10}$ *P*-value of the least statistically significant SNP at 5% FDR. The black vertical dashed lines indicate the positions of two genes (from left to right): a transcription factor (GRMZM2G105494) and *ZmVTE1* (GRMZM2G009785). (B) Scatter plot of association results from a conditional unified mixed model analysis of δT3 and LD estimates (r^2) across the *ZmVTE1* chromosome region, as in (A). The SNP (S5_133333561) from the optimal multi-locus mixed model (MLMM) model was included as a covariate in the unified mixed model to control for the detected effect. (C) Gene model diagram for a transcription factor (GRMZM2G105494) with δT3 associated SNPs. Blue vertical lines indicate the physical position (RefGen_v2) of SNPs within +/- 3 kb of the open reading frame start or stop position for GRMZM2G105494 that are significantly associated with δT3 at 5% FDR. Significant SNPs at 10% FDR are shown as gray vertical lines. The peak SNP is indicated by a red triangle, while the SNP included in the optimal MLMM model is indicated by an inverted red triangle.
