# Supplementary material for: Genome-Wide Association Study and Pathway-Level Analysis of Tocochromanol Levels in Maize Grain
Source: G3 (Bethesda). 2013 Aug 1;3(8):1287–99. doi: 10.1534/g3.113.006148 (PMC3737168; doi:10.1534/g3.113.006148)
Supplement: Supporting Information [file supp_g3.113.006148_TableS2.pdf]

Table S2 Genomic information for the 60 *a priori* candidate genes. Genes that were identified in the pathway level analysis are highlighted in yellow.

a priori candidate gene pathway	RefGen_v2 Gene ID	RefGen_v2 Annotated Gene Function	RefGen_v2 Chromosome	RefGen_v2 ORF start bp	RefGen_v2 ORF stop bp
Aromatic Head Group	GRMZM2G573867	3-dehydroquinate synthase	2	196,359,627	196,363,692
Aromatic Head Group	GRMZM2G051129	3-dehydroquinate synthase	7	175,831,402	175,835,185
Aromatic Head Group	GRMZM5G877500	3-phosphoshikimate 1-carboxyvinyltransferase	9	22,677,939	22,681,262
Aromatic Head Group	GRMZM2G084942	arogenate dehydrogenase isoform 2	5	59,290,440	59,303,420
Aromatic Head Group	GRMZM2G085117	arogenate dehydrogenase isoform 2	5	59,309,561	59,311,237
Aromatic Head Group	GRMZM2G365961	arogenate dehydrogenase isoform 2	6	85,796,094	85,797,825
Aromatic Head Group	GRMZM2G028369	chorismate mutase	3	194,545,569	194,549,360
Aromatic Head Group	AC198937.4_FG003	chorismate mutase	4	83,615,531	83,617,710
Aromatic Head Group	GRMZM2G179454	chorismate mutase	5	92,183,288	92,186,615
Aromatic Head Group	GRMZM2G124365	chorismate mutase	8	173,103,213	173,107,748
Aromatic Head Group	GRMZM2G116087	chorismate mutase	8	173,111,772	173,115,806
Aromatic Head Group	GRMZM2G121546	chorismate mutase/prephenate dehydratase	7	152,703,579	152,742,017
Aromatic Head Group	GRMZM2G164562	chorismate synthase	1	35,455,268	35,465,683
Aromatic Head Group	GRMZM2G036861	chorismate synthase	9	141,935,141	141,941,423
Aromatic Head Group	GRMZM2G448446	fumarylacetoacetate hydrolase domain-containing protein 1	1	262,231,128	262,234,671
Aromatic Head Group	GRMZM2G156516	fumarylacetoacetate hydrolase domain-containing protein 1	1	295,533,612	295,536,939
Aromatic Head Group	GRMZM2G154093	homogentisate 1,2-dioxygenase	9	28,610,042	28,612,570
Aromatic Head Group	GRMZM2G138624	isochorismatase hydrolase	4	133,923,290	133,925,994
Aromatic Head Group	GRMZM2G128880	isochorismatase hydrolase	5	182,244,213	182,248,069
Aromatic Head Group	GRMZM2G437912	prephenate dehydratase	2	59,037,243	59,039,178
Aromatic Head Group	GRMZM2G466543	prephenate dehydratase	2	165,872,056	165,874,345
Aromatic Head Group	GRMZM2G125923	prephenate dehydratase	10	113,515,151	113,516,898
Aromatic Head Group	GRMZM2G324297	prephenate dehydrogenase	9	61,355,950	61,358,310
Aromatic Head Group	GRMZM2G314652	shikimate biosynthesis protein aroDE	3	71,260,323	71,320,829
Aromatic Head Group	GRMZM5G804881	shikimate dehydrogenase	3	133,182,725	133,187,459
Aromatic Head Group	GRMZM2G014376	shikimate dehydrogenase	10	25,975,417	25,980,229
Aromatic Head Group	GRMZM2G004590	shikimate kinase	2	6,482,881	6,486,234
Aromatic Head Group	GRMZM2G161566	shikimate kinase	4	181,725,008	181,727,763
Aromatic Head Group	GRMZM2G070218	shikimate kinase	5	207,704,956	207,707,881
Aromatic Head Group	GRMZM2G002652	tyrosine transaminase	4	210,690,188	210,693,147
Prenyl Group Synthesis	GRMZM2G027059	4-hydroxy-3-methhylbut-2-enyldiphosphate reductase	1	272,936,836	272,940,502
Prenyl Group Synthesis	GRMZM2G056975	1-deoxy-D-xylulose 5-phosphate reductoisomerase	3	30,226,804	30,233,358
Prenyl Group Synthesis	GRMZM2G036290	1-deoxy-D-xylulose 5-phosphate reductoisomerase	8	8,094,442	8,101,055
Prenyl Group Synthesis	GRMZM2G137151	1-deoxy-D-xylulose 5-phosphate synthase	6	146,378,393	146,382,661
Prenyl Group Synthesis	GRMZM2G493395	1-deoxy-D-xylulose 5-phosphate synthase	7	14,077,852	14,081,075
Prenyl Group Synthesis	GRMZM2G173641	1-deoxy-D-xylulose 5-phosphate synthase 1	9	20,462,059	20,467,072
Prenyl Group Synthesis	GRMZM5G835542	2-C-methyl-D-erythritol 2,4-cyclodiphosphate synthase	4	155,830,779	155,832,786
Prenyl Group Synthesis	AC209374.4_FG002	2-C-methyl-D-erythritol 2,4-cyclodiphosphate synthase	5	196,279,295	196,281,037
Prenyl Group Synthesis	GRMZM5G856881	2-C-methyl-D-erythritol 4-phosphate cytidyltransferase	3	170,115,790	170,118,780
Prenyl Group Synthesis	GRMZM2G172032	2-C-methyl-D-erythritol 4-phosphate cytidyltransferase	8	164,748,939	164,752,371
Prenyl Group Synthesis	GRMZM5G859195	4-diphosphocytidyl-2-C-methyl-D-erythritol kinase	3	187,922,271	187,927,591
Prenyl Group Synthesis	GRMZM2G170734	chlorophyllase-2, chloroplast	7	62,130,993	62,132,323
Prenyl Group Synthesis	GRMZM2G105644	geranylgeranyl hydrogenase	5	206,890,298	206,892,838
Prenyl Group Synthesis	GRMZM2G419111	geranylgeranyl hydrogenase	3	40,062,008	40,064,270
Prenyl Group Synthesis	AC194970.5_FG001	geranylgeranyl pyrophosphate synthase 1	2	207,236,995	207,238,335
Prenyl Group Synthesis	GRMZM2G102550	geranylgeranyl pyrophosphate synthase 2	7	160,531,537	160,533,586
Prenyl Group Synthesis	GRMZM2G058404	geranylgeranyl pyrophosphate synthase 3	8	6,358,798	6,360,117
Prenyl Group Synthesis	GRMZM2G137409	hydroxymethylbutenyl 4-diphosphate synthase	5	182,124,005	182,130,631
Prenyl Group Synthesis	GRMZM2G133082	isopentenyl pyrophosphate isomerase	6	147,131,116	147,136,679
Prenyl Group Synthesis	GRMZM2G108285	isopentenyl pyrophosphate isomerase	7	155,559,747	155,562,921
Prenyl Group Synthesis	GRMZM2G145029	isopentenyl pyrophosphate isomerase	8	104,659,886	104,663,941
Tocochromanol Pathway	GRMZM2G374213	4-hydroxyphenylpyruvate dioxygenase	7	28,816,143	28,818,564
Tocochromanol Pathway	GRMZM2G088396	4-hydroxyphenylpyruvate dioxygenase 1	5	83,859,479	83,861,633
Tocochromanol Pathway	GRMZM2G048472	HGA phytyl transferase	9	107,409,674	107,419,362
Tocochromanol Pathway	GRMZM5G848876	homogentisic acid geranylgeranyl transferase 2	3	6,589,628	6,591,631
Tocochromanol Pathway	GRMZM2G173358	homogentisic acid geranylgeranyl transferase 1	9	92,483,549	92,487,268
Tocochromanol Pathway	GRMZM2G082998	MPBQ/MSBQ methyl transferase	1	173,991,404	173,993,446
Tocochromanol Pathway	GRMZM2G104538	phytol kinase	2	2,495,821	2,497,668
Tocochromanol Pathway	GRMZM2G009785	tocopherol cyclase	5	133,501,928	133,518,495
Tocochromanol Pathway	GRMZM2G035213	γ-tocopherol methyltransferase	5	200,367,029	200,370,851
