# Supplementary material for: Genome-Wide Association Study and Pathway-Level Analysis of Tocochromanol Levels in Maize Grain
Source: G3 (Bethesda). 2013 Aug 1;3(8):1287–99. doi: 10.1534/g3.113.006148 (PMC3737168; doi:10.1534/g3.113.006148)
Supplement: Supporting Information [file supp_g3.113.006148_TableS3.pdf]

Table S3 Correlation matrix for untransformed BLUPs of the 20 tocochromanol grain traits. Pearson correlation coefficients are presented in the upper triangle, while the corresponding P-values for the significance of associations ($\alpha = 0.05$) are displayed below the diagonal.

Trait	δT3	γT3	αT3	δT	γT	αT	Total Tocotrienols	Total Tocopherols	Total Tocopherols/Total Tocotrienols	Total Tocochromanols	δT/(γT+αT)	δT/γT	δT/αT	γT/(γT+αT)	δT3/(γT3+αT3)	δT3/γT3	δT3/αT3	γT3/(γT3+αT3)	αT/γT	αT3/γT3
**δT3**		0.64	0.22	0.16	-0.01	-0.03	0.66	-0.01	-0.37	0.33	0.21	0.21	0.06	0.05	0.81	0.55	0.82	0.44	-0.02	-0.29
**γT3**	0.00		0.22	0.23	0.14	-0.10	0.94	0.10	-0.53	0.58	0.24	0.19	0.08	0.19	0.41	0.12	0.49	0.73	-0.17	-0.49
**αT3**	0.00	0.00		-0.07	-0.07	0.40	0.48	0.08	-0.38	0.31	-0.18	-0.07	-0.27	-0.36	-0.06	-0.02	-0.15	-0.20	0.35	0.03
**δT**	0.01	0.00	0.29		0.68	-0.19	0.20	0.62	0.28	0.60	0.74	0.52	0.49	0.52	0.15	0.06	0.22	0.23	-0.48	-0.14
**γT**	0.91	0.02	0.27	0.00		-0.13	0.10	0.93	0.42	0.79	0.19	-0.03	0.39	0.61	-0.07	-0.15	0.04	0.19	-0.55	-0.12
**αT**	0.62	0.10	0.00	0.00	0.03		0.01	0.20	0.08	0.17	-0.40	-0.17	-0.61	-0.72	-0.17	-0.04	-0.20	-0.37	0.65	0.27
**Total Tocotrienols**	0.00	0.00	0.00	0.00	0.12	0.86		0.10	-0.58	0.61	0.18	0.17	0.00	0.07	0.41	0.16	0.42	0.57	-0.05	-0.41
**Total Tocopherols**	0.85	0.12	0.23	0.00	0.00	0.00	0.11		0.45	0.84	0.07	-0.07	0.18	0.34	-0.12	-0.15	-0.02	0.05	-0.31	-0.02
**Total Tocopherols/Total Tocotirenols**	0.00	0.00	0.00	0.00	0.00	0.20	0.00	0.00		0.05	0.03	-0.02	0.14	0.17	-0.25	-0.02	-0.28	-0.50	-0.15	0.50
**Total Tocochromanols**	0.00	0.00	0.00	0.00	0.00	0.01	0.00	0.00	0.43		0.16	0.04	0.15	0.31	0.13	-0.03	0.20	0.35	-0.27	-0.24
**δT/(γT+αT)**	0.00	0.00	0.00	0.00	0.00	0.00	0.00	0.24	0.64	0.01		0.88	0.53	0.45	0.28	0.17	0.28	0.32	-0.42	-0.22
**δT/γT**	0.00	0.00	0.26	0.00	0.59	0.01	0.01	0.29	0.70	0.53	0.00		0.25	0.10	0.27	0.22	0.20	0.17	-0.09	-0.11
**δT/αT**	0.32	0.23	0.00	0.00	0.00	0.00	0.96	0.00	0.02	0.02	0.00	0.00		0.65	0.16	0.01	0.19	0.27	-0.51	-0.22
**γT/(γT+αT)**	0.46	0.00	0.00	0.00	0.00	0.00	0.29	0.00	0.01	0.00	0.00	0.11	0.00		0.13	-0.01	0.20	0.42	-0.95	-0.28
**δT3/(γT3+αT3)**	0.00	0.00	0.31	0.02	0.26	0.01	0.00	0.06	0.00	0.05	0.00	0.00	0.01	0.04		0.81	0.87	0.39	-0.09	-0.24
**δT3/γT3**	0.00	0.07	0.73	0.33	0.02	0.52	0.01	0.02	0.74	0.61	0.01	0.00	0.84	0.89	0.00		0.54	-0.04	0.03	0.23
**δT3/αT3**	0.00	0.00	0.02	0.00	0.53	0.00	0.00	0.71	0.00	0.00	0.00	0.00	0.00	0.00	0.00	0.00		0.55	-0.15	-0.32
**γT3/(γT3+αT3)**	0.00	0.00	0.00	0.00	0.00	0.00	0.00	0.41	0.00	0.00	0.00	0.01	0.00	0.00	0.00	0.58	0.00		-0.37	-0.81
**αT/γT**	0.70	0.01	0.00	0.00	0.00	0.00	0.41	0.00	0.02	0.00	0.00	0.15	0.00	0.00	0.16	0.68	0.01	0.00		0.24
**αT3/γT3**	0.00	0.00	0.67	0.02	0.05	0.00	0.00	0.74	0.00	0.00	0.00	0.09	0.00	0.00	0.00	0.00	0.00	0.00	0.00	
