# Supplementary material for: Genome-Wide Association Study and Pathway-Level Analysis of Tocochromanol Levels in Maize Grain
Source: G3 (Bethesda). 2013 Aug 1;3(8):1287–99. doi: 10.1534/g3.113.006148 (PMC3737168; doi:10.1534/g3.113.006148)
Supplement: Supporting Information [file supp_g3.113.006148_TableS4.pdf]

Table S4 Statistically significant results from the genome-wide association study of 20 tocochromanol grain traits. SNPs that were significantly associated with the indicated trait at 5% FDR are demarcated with boldface font and those significant only at 10% FDR without boldface font.

a priori candidate gene pathway	RefGen_v2 Gene ID	Annotated gene containing associated SNP or gene within 3kb of associated SNP	Trait	SNP ID	SNP Source	Chr	Position in RefGen_v2	*P*-value	FDR-Adjusted *P*-value	Identified in Li et al. (2012)	Minor Allele Frequency (MAF)	Sample Size	MAF Tropical (18% of 252 Lines)	MAF Temperate (82% of 252 Lines)	R^2_{LR} from Model without SNP	R^2_{LR} from Model with SNP	Effect Size	Lambda from Box-Cox Procedure	Back-Transformed Effect Estimates
			αT/γT	S1_16043898	GBS	1	16,043,898	7.25E-06	7.46E-02	-	0.10	246	0.09	0.13	0.24	0.31	-0.05	0.15	-0.29
			δT/αT	S1_42184756	GBS	1	42,184,756	7.43E-06	9.87E-02	-	0.20	246	0.05	0.24	0.28	0.34	0.38	Log	1.26
			αT	S1_69880751	GBS	1	69,880,751	8.96E-06	8.87E-02	-	0.33	251	0.41	0.40	0.25	0.32	-0.19	0.40	-0.41
			αT/γT	S1_207726397	GBS	1	207,726,397	3.89E-06	5.16E-02	-	0.10	246	0.41	0.07	0.24	0.31	-0.07	0.15	-0.38
			αT/γT	S1_207726398	GBS	1	207,726,398	3.89E-06	5.16E-02	-	0.10	246	0.41	0.07	0.24	0.31	0.07	0.15	0.57
			δT3/(γT3+αT3)	S2_71606588	GBS	2	71,606,588	6.14E-06	9.55E-02	-	0.08	248	0.21	0.07	0.10	0.18	0.05	-0.10	-0.39
			αT	S2_196514167	GBS	2	196,514,167	7.19E-06	8.12E-02	-	0.06	251	0.02	0.07	0.25	0.32	-0.36	0.40	-0.67
			αT	S2_219396629	GBS	2	219,396,629	8.33E-06	8.74E-02	-	0.16	251	0.00	0.23	0.25	0.32	-0.27	0.40	-0.54
			αT	S2_219396633	GBS	2	219,396,633	8.33E-06	8.74E-02	-	0.16	251	0.00	0.23	0.25	0.32	0.27	0.40	0.82
			γT/(γT+αT)	ss196506839	55K	3	197,518,747	5.72E-06	9.34E-02	-	0.26	251	0.27	0.26	0.29	0.35	0.09	2.00	0.04
			δT3	S5_132656905	GBS	5	132,656,905	2.15E-06	8.98E-02	-	0.48	247	0.36	0.44	0.12	0.20	0.06	-0.20	-0.25
			δT3/(γT3+αT3)	S5_133189938	GBS	5	133,189,938	2.29E-06	6.85E-02	-	0.41	248	0.19	0.46	0.10	0.18	-0.03	-0.10	0.36
	GRMZM2G105494	transcription factor	δT3	S5_133331096	GBS	5	133,331,096	1.01E-06	7.37E-02	-	0.40	247	0.44	0.41	0.12	0.21	-0.06	-0.20	0.36
	GRMZM2G105494	transcription factor	δT3	S5_133331106	GBS	5	133,331,106	1.01E-06	7.37E-02	-	0.40	247	0.47	0.41	0.12	0.21	0.06	-0.20	-0.25
	**GRMZM2G105494**	**transcription factor**	**δT3**	**S5_133333397**	**GBS**	**5**	**133,333,397**	**3.06E-07**	**4.56E-02**	**-**	**0.43**	**247**	**0.47**	**0.43**	**0.12**	**0.22**	**0.06**	**-0.20**	**-0.25**
	GRMZM2G105494	transcription factor	δT3	ss196465626	55K	5	133,333,397	1.69E-06	8.25E-02	-	0.42	247	0.47	0.42	0.12	0.21	0.06	-0.20	-0.25
	GRMZM2G105494	transcription factor	δT3/(γT3+αT3)	S5_133333397	GBS	5	133,333,397	6.13E-06	9.55E-02	-	0.43	248	0.47	0.43	0.10	0.18	0.03	-0.10	-0.26
	**GRMZM2G105494**	**transcription factor**	**δT3**	**S5_133333561**	**GBS**	**5**	**133,333,561**	**3.12E-07**	**4.56E-02**	**-**	**0.42**	**247**	**0.48**	**0.43**	**0.12**	**0.22**	**0.06**	**-0.20**	**-0.25**
	GRMZM2G105494	transcription factor	δT3/(γT3+αT3)	S5_133333561	GBS	5	133,333,561	8.28E-06	9.55E-02	-	0.42	248	0.48	0.43	0.10	0.17	0.03	-0.10	-0.26
	GRMZM2G105494	transcription factor	δT3	S5_133335078	GBS	5	133,335,078	1.34E-06	7.85E-02	-	0.30	247	0.33	0.29	0.12	0.21	-0.06	-0.20	0.36
Tocochromanol Pathway	GRMZM2G009785	tocopherol cyclase	δT3/(γT3+αT3)	S5_133499169	GBS	5	133,499,169	1.77E-06	6.49E-02	-	0.39	248	0.15	0.44	0.10	0.19	-0.03	-0.10	0.36
Tocochromanol Pathway	GRMZM2G009785	tocopherol cyclase	δT3/(γT3+αT3)	S5_133499269	GBS	5	133,499,269	3.66E-06	8.95E-02	-	0.39	248	0.18	0.46	0.10	0.18	0.03	-0.10	-0.26
**Tocochromanol Pathway**	**GRMZM2G009785**	**tocopherol cyclase**	**δT3/(γT3+αT3)**	**S5_133501858**	**GBS**	**5**	**133,501,858**	**1.29E-07**	**3.78E-02**	**-**	**0.40**	**248**	**0.19**	**0.46**	**0.10**	**0.21**	**0.04**	**-0.10**	**-0.32**
Tocochromanol Pathway	GRMZM2G009785	tocopherol cyclase	δT3/(γT3+αT3)	S5_133501992	GBS	5	133,501,992	9.72E-07	5.53E-02	-	0.40	248	0.14	0.46	0.10	0.19	0.03	-0.10	-0.26
Tocochromanol Pathway	GRMZM2G009785	tocopherol cyclase	δT3/(γT3+αT3)	PZB00969.1	4K	5	133,502,506	8.27E-07	5.53E-02	-	0.41	248	0.14	0.46	0.10	0.19	0.03	-0.10	-0.26
Tocochromanol Pathway	GRMZM2G009785	tocopherol cyclase	δT3/(γT3+αT3)	ss196465634	55K	5	133,510,613	7.54E-06	9.55E-02	-	0.41	248	0.14	0.46	0.10	0.17	-0.03	-0.10	0.36
Tocochromanol Pathway	GRMZM2G009785	tocopherol cyclase	δT3/(γT3+αT3)	PZB02491.1	4K	5	133,517,065	6.69E-06	9.55E-02	-	0.40	248	0.14	0.46	0.10	0.18	-0.03	-0.10	0.36
			δT3/(γT3+αT3)	S5_133618308	GBS	5	133,618,308	7.89E-06	9.55E-02	-	0.40	248	0.10	0.48	0.10	0.17	0.03	-0.10	-0.26
			δT3/(γT3+αT3)	S5_133618309	GBS	5	133,618,309	7.89E-06	9.55E-02	-	0.40	248	0.10	0.48	0.10	0.17	0.03	-0.10	-0.26
			δT3/(γT3+αT3)	S5_133618344	GBS	5	133,618,344	7.89E-06	9.55E-02	-	0.40	248	0.10	0.48	0.10	0.17	0.03	-0.10	-0.26
			δT3/(γT3+αT3)	S5_133691297	GBS	5	133,691,297	5.02E-06	9.55E-02	-	0.37	248	0.10	0.46	0.10	0.18	0.03	-0.10	-0.26
			δT3/(γT3+αT3)	S5_133776316	GBS	5	133,776,316	6.51E-07	5.53E-02	-	0.39	248	0.11	0.46	0.10	0.19	-0.03	-0.10	0.36
			δT3/(γT3+αT3)	S5_134191258	GBS	5	134,191,258	1.66E-06	6.49E-02	-	0.40	248	0.12	0.47	0.10	0.19	0.03	-0.10	-0.26
			δT3/(γT3+αT3)	S5_134195301	GBS	5	134,195,301	2.92E-06	7.77E-02	-	0.39	248	0.14	0.47	0.10	0.18	-0.03	-0.10	0.36
			δT3/(γT3+αT3)	S5_134471200	GBS	5	134,471,200	1.13E-06	5.53E-02	-	0.40	248	0.13	0.46	0.10	0.19	-0.03	-0.10	0.36
			δT3/(γT3+αT3)	S5_134489952	GBS	5	134,489,952	1.13E-06	5.53E-02	-	0.40	248	0.14	0.47	0.10	0.19	-0.03	-0.10	0.36
			δT3/(γT3+αT3)	S5_134751850	GBS	5	134,751,850	2.34E-06	6.85E-02	-	0.43	248	0.21	0.47	0.10	0.18	-0.03	-0.10	0.36
			δT3/(γT3+αT3)	S5_134751883	GBS	5	134,751,883	7.69E-06	9.55E-02	-	0.43	248	0.14	0.45	0.10	0.17	0.03	-0.10	-0.26
			δT3/(γT3+αT3)	S5_134751924	GBS	5	134,751,924	6.83E-06	9.55E-02	-	0.43	248	0.21	0.46	0.10	0.18	-0.03	-0.10	0.36
			δT3/(γT3+αT3)	S5_134787527	GBS	5	134,787,527	6.62E-06	9.55E-02	-	0.33	248	0.08	0.42	0.10	0.18	-0.03	-0.10	0.36
			δT3/(γT3+αT3)	S5_135908766	GBS	5	135,908,766	8.47E-06	9.55E-02	-	0.36	248	0.41	0.39	0.10	0.17	-0.03	-0.10	0.36
			δT3/(γT3+αT3)	S5_135908783	GBS	5	135,908,783	8.47E-06	9.55E-02	-	0.36	248	0.41	0.39	0.10	0.17	-0.03	-0.10	0.36
	**GRMZM2G031952**	**ELMO domain-containing protein 2**	**γT/(γT+αT)**	**S5_190739882**	**GBS**	**5**	**190,739,882**	**1.15E-06**	**2.42E-02**	**-**	**0.24**	**251**	**0.03**	**0.34**	**0.29**	**0.36**	**-0.08**	**2.00**	**-0.04**
	GRMZM2G031952	ELMO domain-containing protein 2	αT/γT	S5_190739882	GBS	5	190,739,882	7.45E-06	7.46E-02	-	0.25	246	0.03	0.34	0.24	0.31	0.04	0.15	0.3
	**GRMZM2G031952**	**ELMO domain-containing protein 2**	**γT/(γT+αT)**	**S5_190739951**	**GBS**	**5**	**190,739,951**	**9.59E-08**	**3.13E-03**	**-**	**0.24**	**251**	**0.05**	**0.30**	**0.29**	**0.37**	**0.09**	**2.00**	**0.04**
	**GRMZM2G031952**	**ELMO domain-containing protein 2**	**αT/γT**	**S5_190739951**	**GBS**	**5**	**190,739,951**	**5.40E-07**	**1.21E-02**	**-**	**0.24**	**246**	**0.05**	**0.30**	**0.24**	**0.33**	**-0.05**	**0.15**	**-0.29**
	**GRMZM2G058943**	**Hypothetical gene of unknown function**	**αT**	**S5_198205644**	**GBS**	**5**	**198,205,644**	**3.56E-06**	**4.98E-02**	**-**	**0.18**	**251**	**0.15**	**0.19**	**0.25**	**0.32**	**-0.25**	**0.40**	**-0.51**
	**GRMZM5G823157**	**WYRKY transciription factor**	**αT**	**S5_199647093**	**GBS**	**5**	**199,647,093**	**3.54E-06**	**4.98E-02**	**-**	**0.24**	**251**	**0.28**	**0.36**	**0.25**	**0.32**	**0.21**	**0.40**	**0.61**
	GRMZM5G823157	WYRKY transciription factor	δT/αT	S5_199647093	GBS	5	199,647,093	4.26E-06	7.78E-02	-	0.23	246	0.28	0.36	0.28	0.34	-0.35	Log	-0.8
		**intergenic region**	**αT/γT**	**S5_199706178**	**GBS**	**5**	**199,706,178**	**1.07E-06**	**1.84E-02**	**-**	**0.14**	**246**	**0.29**	**0.22**	**0.24**	**0.32**	**0.05**	**0.15**	**0.38**
		**intergenic region**	**γT/(γT+αT)**	**S5_199706178**	**GBS**	**5**	**199,706,178**	**1.22E-06**	**2.42E-02**	**-**	**0.15**	**251**	**0.29**	**0.22**	**0.29**	**0.36**	**-0.09**	**2.00**	**-0.05**
		**intergenic region**	**δT/αT**	**S5_199706178**	**GBS**	**5**	**199,706,178**	**1.68E-06**	**3.79E-02**	**-**	**0.15**	**246**	**0.29**	**0.22**	**0.28**	**0.35**	**-0.45**	**Log**	**-0.99**
			αT/γT	S5_199706244	GBS	5	199,706,244	8.86E-06	8.08E-02	-	0.13	246	0.33	0.18	0.24	0.31	-0.05	0.15	-0.29
	**GRMZM2G108996**	**Protein Brevis radix-like 2**	**αT**	**S5_200114137**	**GBS**	**5**	**200,114,137**	**2.63E-07**	**6.44E-03**	**-**	**0.35**	**251**	**0.39**	**0.42**	**0.25**	**0.34**	**-0.22**	**0.40**	**-0.46**
	**GRMZM2G034876**	**growth-regulating factor 1**	**γT/(γT+αT)**	**S5_200293693**	**GBS**	**5**	**200,293,693**	**1.24E-06**	**2.42E-02**	**-**	**0.42**	**251**	**0.34**	**0.37**	**0.29**	**0.36**	**-0.07**	**2.00**	**-0.04**
	**GRMZM2G034876**	**growth-regulating factor 1**	**αT**	**S5_200293693**	**GBS**	**5**	**200,293,693**	**3.33E-06**	**4.98E-02**	**-**	**0.41**	**251**	**0.34**	**0.37**	**0.25**	**0.32**	**0.20**	**0.40**	**0.58**
	GRMZM2G034876	growth-regulating factor 1	αT/γT	S5_200293693	GBS	5	200,293,693	8.10E-06	7.63E-02	-	0.40	246	0.34	0.37	0.24	0.31	0.04	0.15	0.3
			δT/αT	ss196468356	55K	5	200,300,836	6.33E-06	9.33E-02	Yes	0.43	246	0.20	0.49	0.28	0.34	0.33	Log	1.06
		**intergenic region**	**αT**	**S5_200318615**	**GBS**	**5**	**200,318,615**	**1.05E-06**	**2.05E-02**	**-**	**0.22**	**251**	**0.27**	**0.44**	**0.25**	**0.33**	**0.25**	**0.40**	**0.75**
		**intergenic region**	**αT/γT**	**S5_200318615**	**GBS**	**5**	**200,318,615**	**2.50E-06**	**3.82E-02**	**-**	**0.22**	**246**	**0.27**	**0.44**	**0.24**	**0.32**	**0.05**	**0.15**	**0.38**
**Tocochromanol Pathway**	**GRMZM2G035213**	**γ-tocopherol methyltransferase**	**αT**	**ss196416269**	**55K**	**5**	**200,367,532**	**7.36E-14**	**7.21E-09**	**Yes**	**0.20**	**251**	**0.13**	**0.22**	**0.25**	**0.44**	**0.40**	**0.40**	**1.32**
**Tocochromanol Pathway**	**GRMZM2G035213**	**γ-tocopherol methyltransferase**	**αT**	**PZB02283.1**	**4K**	**5**	**200,367,532**	**7.36E-14**	**7.21E-09**	**-**	**0.20**	**251**	**0.14**	**0.22**	**0.25**	**0.44**	**0.40**	**0.40**	**1.32**
**Tocochromanol Pathway**	**GRMZM2G035213**	**γ-tocopherol methyltransferase**	**αT/γT**	**PZB02283.1**	**4K**	**5**	**200,367,532**	**9.21E-13**	**8.96E-08**	**-**	**0.21**	**246**	**0.14**	**0.22**	**0.24**	**0.42**	**0.08**	**0.15**	**0.67**
**Tocochromanol**	**GRMZM2G035213**	**γ-tocopherol**	**αT/γT**	**ss196416269**	**55K**	**5**		**9.21E-13**	**8.96E-08**	**Yes**	**0.21**	**246**	**0.13**	**0.22**	**0.24**	**0.42**	**0.08**	**0.15**	**0.67**

Pathway		methyltransferase					200,367,532												
Tocochromanol Pathway	GRMZM2G035213	γ-tocopherol methyltransferase	γT/(γT+αT)	PZB02283.1	4K	5	200,367,532	4.82E-12	4.72E-07	-	0.20	251	0.14	0.22	0.29	0.44	-0.13	2.00	-0.07
Tocochromanol Pathway	GRMZM2G035213	γ-tocopherol methyltransferase	γT/(γT+αT)	ss196416269	55K	5	200,367,532	4.82E-12	4.72E-07	Yes	0.20	251	0.13	0.22	0.29	0.44	-0.13	2.00	-0.07
Tocochromanol Pathway	GRMZM2G035213	γ-tocopherol methyltransferase	δT/αT	PZB02283.1	4K	5	200,367,532	1.88E-11	1.83E-06	-	0.20	246	0.14	0.22	0.28	0.42	-0.63	Log	-1.27
Tocochromanol Pathway	GRMZM2G035213	γ-tocopherol methyltransferase	δT/αT	ss196416269	55K	5	200,367,532	1.88E-11	1.83E-06	Yes	0.20	246	0.13	0.22	0.28	0.42	-0.63	Log	-1.27
Tocochromanol Pathway	GRMZM2G035213	γ-tocopherol methyltransferase	αT	ss196468362	55K	5	200,369,124	7.36E-14	7.21E-09	Yes	0.20	251	0.13	0.22	0.25	0.44	0.40	0.40	1.32
Tocochromanol Pathway	GRMZM2G035213	γ-tocopherol methyltransferase	αT/γT	ss196468362	55K	5	200,369,124	9.21E-13	8.96E-08	Yes	0.21	246	0.13	0.22	0.24	0.42	0.08	0.15	0.67
Tocochromanol Pathway	GRMZM2G035213	γ-tocopherol methyltransferase	γT/(γT+αT)	ss196468362	55K	5	200,369,124	4.82E-12	4.72E-07	Yes	0.20	251	0.13	0.22	0.29	0.44	-0.13	2.00	-0.07
Tocochromanol Pathway	GRMZM2G035213	γ-tocopherol methyltransferase	δT/αT	ss196468362	55K	5	200,369,124	1.88E-11	1.83E-06	Yes	0.20	246	0.13	0.22	0.28	0.42	-0.63	Log	-1.27
Tocochromanol Pathway	GRMZM2G035213	γ-tocopherol methyltransferase	αT	S5_200369481	GBS	5	200,369,481	1.97E-08	9.61E-04	-	0.11	251	0.36	0.09	0.25	0.35	0.38	0.40	1.24
Tocochromanol Pathway	GRMZM2G035213	γ-tocopherol methyltransferase	αT/γT	S5_200369481	GBS	5	200,369,481	7.24E-07	1.51E-02	-	0.10	246	0.36	0.09	0.24	0.32	0.07	0.15	0.57
Tocochromanol Pathway	GRMZM2G035213	γ-tocopherol methyltransferase	γT/(γT+αT)	S5_200369481	GBS	5	200,369,481	6.15E-09	3.62E-04	-	0.12	251	0.36	0.09	0.29	0.39	-0.14	2.00	-0.07
Tocochromanol Pathway	GRMZM2G035213	γ-tocopherol methyltransferase	δT/αT	S5_200369481	GBS	5	200,369,481	7.21E-07	2.10E-02	-	0.12	246	0.36	0.09	0.28	0.35	-0.56	Log	-1.17
Tocochromanol Pathway	GRMZM2G035213	γ-tocopherol methyltransferase	αT	S5_200369508	GBS	5	200,369,508	6.85E-07	1.44E-02	-	0.18	251	0.11	0.22	0.25	0.33	0.28	0.40	0.85
Tocochromanol Pathway	GRMZM2G035213	γ-tocopherol methyltransferase	αT/γT	S5_200369508	GBS	5	200,369,508	9.69E-07	1.77E-02	-	0.18	246	0.11	0.22	0.24	0.32	0.06	0.15	0.47
Tocochromanol Pathway	GRMZM2G035213	γ-tocopherol methyltransferase	γT/(γT+αT)	S5_200369508	GBS	5	200,369,508	6.57E-07	1.61E-02	-	0.18	251	0.11	0.22	0.29	0.36	-0.10	2.00	-0.05
Tocochromanol Pathway	GRMZM2G035213	γ-tocopherol methyltransferase	αT	S5_200369534	GBS	5	200,369,534	2.59E-06	4.22E-02	-	0.09	251	0.00	0.12	0.25	0.32	-0.34	0.40	-0.65
Tocochromanol Pathway	GRMZM2G035213	γ-tocopherol methyltransferase	αT/γT	S5_200369534	GBS	5	200,369,534	5.45E-06	6.37E-02	-	0.09	246	0.00	0.12	0.24	0.31	-0.07	0.15	-0.38
Tocochromanol Pathway	GRMZM2G035213	γ-tocopherol methyltransferase	αT	S5_200369625	GBS	5	200,369,625	5.00E-08	1.63E-03	-	0.30	251	0.48	0.49	0.25	0.35	-0.24	0.40	-0.5
Tocochromanol Pathway	GRMZM2G035213	γ-tocopherol methyltransferase	δT/αT	S5_200369625	GBS	5	200,369,625	2.02E-06	4.22E-02	-	0.30	246	0.48	0.49	0.28	0.35	0.35	Log	1.14
Tocochromanol Pathway	GRMZM2G035213	γ-tocopherol methyltransferase	αT	S5_200369644	GBS	5	200,369,644	9.36E-06	8.87E-02	-	0.12	251	0.12	0.22	0.25	0.32	-0.26	0.40	-0.53
Tocochromanol Pathway	GRMZM2G035213	γ-tocopherol methyltransferase	αT/γT	S5_200369644	GBS	5	200,369,644	9.97E-06	8.32E-02	-	0.13	246	0.12	0.22	0.24	0.31	-0.05	0.15	-0.29
Tocochromanol Pathway	GRMZM2G035213	γ-tocopherol methyltransferase	αT	S5_200369665	GBS	5	200,369,665	9.36E-06	8.87E-02	-	0.12	251	0.12	0.22	0.25	0.32	0.26	0.40	0.78
Tocochromanol Pathway	GRMZM2G035213	γ-tocopherol methyltransferase	αT/γT	S5_200369665	GBS	5	200,369,665	9.97E-06	8.32E-02	-	0.13	246	0.12	0.22	0.24	0.31	0.05	0.15	0.38
Tocochromanol Pathway	GRMZM2G035213	γ-tocopherol methyltransferase	αT	S5_200369667	GBS	5	200,369,667	1.67E-07	4.47E-03	-	0.10	251	0.00	0.20	0.25	0.34	-0.33	0.40	-0.63
Tocochromanol Pathway	GRMZM2G035213	γ-tocopherol methyltransferase	αT/γT	S5_200369667	GBS	5	200,369,667	1.70E-07	5.22E-03	-	0.11	246	0.00	0.20	0.24	0.33	-0.07	0.15	-0.38
Tocochromanol Pathway	GRMZM2G035213	γ-tocopherol methyltransferase	δT/αT	S5_200369667	GBS	5	200,369,667	7.09E-06	9.87E-02	-	0.10	246	0.00	0.20	0.28	0.34	0.48	Log	1.67
Tocochromanol Pathway	GRMZM2G035213	γ-tocopherol methyltransferase	αT	PZB02424.2	4K	5	200,370,309	4.09E-07	9.25E-03	-	0.17	251	0.09	0.19	0.25	0.33	-0.26	0.40	-0.53
Tocochromanol Pathway	GRMZM2G035213	γ-tocopherol methyltransferase	αT	ss196468368	55K	5	200,371,057	6.73E-06	8.08E-02	-	0.14	251	0.13	0.15	0.25	0.32	-0.27	0.40	-0.54
Tocochromanol Pathway	GRMZM2G035213	γ-tocopherol methyltransferase	αT/γT	ss196468368	55K	5	200,371,057	7.37E-06	7.46E-02	-	0.14	246	0.13	0.15	0.24	0.31	-0.05	0.15	-0.29
	GRMZM2G167431	3-ketodihydrosphingosine reductase	γT/(γT+αT)	S5_200382168	GBS	5	200,382,168	9.59E-09	4.03E-04	-	0.39	251	0.38	0.40	0.29	0.39	0.09	2.00	0.04
	GRMZM2G167431	3-ketodihydrosphingosine reductase	αT	S5_200382168	GBS	5	200,382,168	3.01E-08	1.11E-03	-	0.39	251	0.38	0.40	0.25	0.35	-0.25	0.40	-0.51
	GRMZM2G167431	3-ketodihydrosphingosine reductase	αT/γT	S5_200382168	GBS	5	200,382,168	3.08E-08	1.31E-03	-	0.39	246	0.38	0.40	0.24	0.34	-0.05	0.15	-0.29
	GRMZM2G167431	3-ketodihydrosphingosine reductase	δT/αT	S5_200382168	GBS	5	200,382,168	6.31E-08	2.63E-03	-	0.41	246	0.38	0.40	0.28	0.37	0.41	Log	1.38
	GRMZM2G325001	Mitochondrial uncoupling protein 1	γT/(γT+αT)	S5_200435108	GBS	5	200,435,108	1.13E-10	8.31E-06	-	0.27	251	0.44	0.21	0.29	0.42	0.11	2.00	0.05
	GRMZM2G325001	Mitochondrial uncoupling protein 1	αT/γT	S5_200435108	GBS	5	200,435,108	3.28E-10	2.40E-05	-	0.25	246	0.44	0.21	0.24	0.38	-0.06	0.15	-0.34
	GRMZM2G325001	Mitochondrial uncoupling protein 1	αT	S5_200435108	GBS	5	200,435,108	4.39E-10	3.22E-05	-	0.26	251	0.44	0.21	0.25	0.38	-0.30	0.40	-0.59
	GRMZM2G325001	Mitochondrial uncoupling protein 1	δT/αT	S5_200435108	GBS	5	200,435,108	6.37E-09	4.65E-04	-	0.27	246	0.44	0.21	0.28	0.39	0.47	Log	1.63
	GRMZM2G325001	Mitochondrial uncoupling protein 1	αT	S5_200435117	GBS	5	200,435,117	1.21E-07	3.56E-03	-	0.26	251	0.19	0.28	0.25	0.34	0.25	0.40	0.75
	GRMZM2G325001	Mitochondrial uncoupling protein 1	γT/(γT+αT)	S5_200435117	GBS	5	200,435,117	1.66E-07	4.87E-03	-	0.26	251	0.19	0.28	0.29	0.37	-0.09	2.00	-0.05
	GRMZM2G325001	Mitochondrial uncoupling protein 1	αT/γT	S5_200435117	GBS	5	200,435,117	1.79E-07	5.22E-03	-	0.27	246	0.19	0.28	0.24	0.33	0.05	0.15	0.38
	GRMZM2G325001	Mitochondrial uncoupling protein 1	δT/αT	S5_200435117	GBS	5	200,435,117	7.93E-07	2.10E-02	-	0.25	246	0.19	0.28	0.28	0.35	-0.41	Log	-0.91
	GRMZM2G325019	Pentatricopeptide repeat-containing protein	αT	ss196468352	55K	5	200,435,300	8.32E-10	4.89E-05	Yes	0.40	251	0.39	0.37	0.25	0.38	-0.27	0.40	-0.54
	GRMZM2G325019	Pentatricopeptide repeat-containing protein	γT/(γT+αT)	ss196468352	55K	5	200,435,300	7.67E-09	3.76E-04	Yes	0.40	251	0.39	0.37	0.29	0.39	0.09	2.00	0.04
	GRMZM2G325019	Pentatricopeptide repeat-containing protein	αT/γT	ss196468352	55K	5	200,435,300	2.68E-08	1.31E-03	Yes	0.39	246	0.39	0.37	0.24	0.35	-0.05	0.15	-0.29
	GRMZM2G325019	Pentatricopeptide repeat-containing protein	δT/αT	ss196468352	55K	5	200,435,300	2.87E-08	1.67E-03	Yes	0.41	246	0.39	0.37	0.28	0.38	0.42	Log	1.42
	GRMZM2G325019	Pentatricopeptide repeat-containing protein	αT	S5_200437468	GBS	5	200,437,468	2.29E-08	9.61E-04	-	0.28	251	0.45	0.24	0.25	0.35	-0.26	0.40	-0.53
	GRMZM2G325019	Pentatricopeptide repeat-containing protein	γT/(γT+αT)	S5_200437468	GBS	5	200,437,468	4.13E-08	1.52E-03	-	0.28	251	0.45	0.24	0.29	0.38	0.09	2.00	0.04
	GRMZM2G325019	Pentatricopeptide repeat-containing protein	αT/γT	S5_200437468	GBS	5	200,437,468	9.02E-08	3.29E-03	-	0.27	246	0.45	0.24	0.24	0.34	-0.05	0.15	-0.29
	GRMZM2G325019	Pentatricopeptide repeat-containing protein	δT/αT	S5_200437468	GBS	5	200,437,468	3.72E-07	1.26E-02	-	0.29	246	0.45	0.24	0.28	0.36	0.40	Log	1.34
	GRMZM2G325019	Pentatricopeptide repeat-containing protein	αT/γT	ss196517251	55K	5	200,437,606	4.45E-07	1.08E-02	Yes	0.45	246	0.23	0.40	0.24	0.33	-0.04	0.15	-0.24
	GRMZM2G325019	Pentatricopeptide repeat-containing protein	αT	ss196517251	55K	5	200,437,606	1.38E-06	2.38E-02	Yes	0.46	251	0.23	0.40	0.25	0.33	-0.21	0.40	-0.45
	GRMZM2G325019	Pentatricopeptide repeat-containing protein	γT/(γT+αT)	ss196517251	55K	5	200,437,606	2.99E-06	5.18E-02	Yes	0.46	251	0.23	0.40	0.29	0.35	0.07	2.00	0.03
	GRMZM2G325019	Pentatricopeptide repeat-containing protein	δT/αT	ss196517251	55K	5	200,437,606	5.53E-06	9.33E-02	Yes	0.47	246	0.23	0.40	0.28	0.34	0.35	Log	1.14
	GRMZM2G325038	Transcription factor GT-3b	αT	S5_200438801	GBS	5	200,438,801	4.30E-06	5.74E-02	-	0.10	251	0.06	0.13	0.25	0.32	0.31	0.40	0.96
	GRMZM2G325038	Transcription factor GT-3b	αT/γT	S5_200438801	GBS	5	200,438,801	7.67E-06	7.46E-02	-	0.10	246	0.06	0.13	0.24	0.31	0.06	0.15	0.47
	GRMZM2G161641	amino acid permease	αT/γT	S5_204064933	GBS	5	204,064,933	3.15E-08	1.31E-03	-	0.15	246	0.17	0.15	0.24	0.34	-0.06	0.15	-0.34
	GRMZM2G161641	amino acid permease	γT/(γT+αT)	S5_204064933	GBS	5	204,064,933	3.32E-07	8.87E-03	-	0.15	251	0.17	0.15	0.29	0.36	0.10	2.00	0.05
	GRMZM2G161641	amino acid permease	αT	S5_204064933	GBS	5	204,064,933	1.16E-06	2.13E-02	-	0.15	251	0.17	0.15	0.25	0.33	-0.27	0.40	-0.54
	GRMZM2G161641	amino acid permease	δT/αT	S5_204064933	GBS	5	204,064,933	8.84E-07	2.15E-02	-	0.13	246	0.17	0.15	0.28	0.35	0.48	Log	1.67
	GRMZM2G161641	amino acid permease	αT/γT	S5_204065009	GBS	5	204,065,009	3.42E-07	9.07E-03	-	0.13	246	0.13	0.16	0.24	0.33	-0.06	0.15	-0.34
	GRMZM2G161641	amino acid permease	δT/αT	S5_204065009	GBS	5	204,065,009	3.87E-07	1.26E-02	-	0.13	246	0.13	0.16	0.28	0.36	0.48	Log	1.67
	GRMZM2G161641	amino acid permease	γT/(γT+αT)	S5_204065009	GBS	5	204,065,009	1.74E-06	3.20E-02	-	0.13	251	0.13	0.16	0.29	0.35	0.09	2.00	0.04
	GRMZM2G161641	amino acid permease	αT	S5_204065009	GBS	5	204,065,009	4.64E-06	5.92E-02	-	0.13	251	0.13	0.16	0.25	0.32	-0.26	0.40	-0.53
	GRMZM2G161641	amino acid permease	δT/αT	S5_204065055	GBS	5	204,065,055	4.68E-08	2.28E-03	-	0.16	246	0.15	0.20	0.28	0.37	0.49	Log	1.72
	GRMZM2G161641	amino acid permease	αT/γT	S5_204065055	GBS	5	204,065,055	2.36E-06	3.82E-02	-	0.17	246	0.15	0.20	0.24	0.32	-0.05	0.15	-0.29
		intergenic	αT/γT	S6_149776596	GBS	6	149,776,596	9.71E-07	1.77E-02	-	0.17	246	0.03	0.24	0.24	0.32	-0.05	0.15	-0.29

			αT	S6_149776596	GBS	6	149,776,596	6.87E-06	8.08E-02	-	0.16	251	0.03	0.24	0.25	0.32	-0.23	0.40	-0.48
			αT/γT	S6_149776597	GBS	6	149,776,597	4.37E-06	5.54E-02	-	0.14	246	0.00	0.21	0.24	0.31	0.05	0.15	0.38
			αT	S6_149776597	GBS	6	149,776,597	1.03E-05	9.45E-02	-	0.14	251	0.00	0.21	0.25	0.31	0.23	0.40	0.68
		gene of unknown function	αT/γT	ss196475603	55K	7	13,959,219	1.25E-05	9.86E-02	-	0.30	246	0.37	0.30	0.24	0.31	0.04	0.15	0.3
			αT/γT	ss196486649	55K	8	128,548,850	7.50E-06	7.46E-02	-	0.15	246	0.11	0.16	0.24	0.31	0.06	0.15	0.47
	**GRMZM5G833760**	**phytosulfokine receptor**	**γT3**	**S9_92718671**	**GBS**	**9**	**92,718,671**	**4.48E-07**	**4.40E-02**	**-**	**0.08**	**250**	**0.00**	**0.13**	**0.20**	**0.28**	**-0.05**	**0.10**	**-0.4**
	**GRMZM5G833760**	**phytosulfokine receptor**	**γT3**	**S9_92718674**	**GBS**	**9**	**92,718,674**	**4.48E-07**	**4.40E-02**	**-**	**0.08**	**250**	**0.00**	**0.13**	**0.20**	**0.28**	**-0.05**	**0.10**	**-0.4**
	**GRMZM5G833760**	**phytosulfokine receptor**	**γT3**	**S9_92718709**	**GBS**	**9**	**92,718,709**	**4.48E-07**	**4.40E-02**	**-**	**0.08**	**250**	**0.00**	**0.13**	**0.20**	**0.28**	**-0.05**	**0.10**	**-0.4**
		intergenic region	Total Tocotrienols	S9_107839821	GBS	9	107,839,821	2.00E-07	5.88E-02	-	0.20	250	0.38	0.23	0.20	0.30	-0.05	0.15	-0.29
			αT/γT	S9_151726463	GBS	9	151,726,463	4.81E-06	5.86E-02	-	0.08	246	0.03	0.11	0.24	0.31	0.06	0.15	0.47
			αT/γT	S9_151726511	GBS	9	151,726,511	1.07E-05	8.69E-02	-	0.12	246	0.06	0.14	0.24	0.31	-0.05	0.15	-0.29
			αT/γT	S9_151726870	GBS	9	151,726,870	9.55E-06	8.32E-02	-	0.13	246	0.15	0.13	0.24	0.31	0.05	0.15	0.38
			**αT/γT**	**S9_155585508**	**GBS**	**9**	**155,585,508**	**2.62E-06**	**3.82E-02**	**-**	**0.15**	**246**	**0.27**	**0.13**	**0.24**	**0.32**	**0.05**	**0.15**	**0.38**
			δT/αT	S9_155585508	GBS	9	155,585,508	2.75E-06	5.35E-02	-	0.15	246	0.27	0.13	0.28	0.35	-0.45	Log	-0.99
			δT/αT	S10_129705301	GBS	10	129,705,301	6.39E-06	9.33E-02	-	0.08	246	0.05	0.13	0.28	0.34	-0.64	Log	-1.28
			δT/αT	S10_129705304	GBS	10	129,705,304	6.39E-06	9.33E-02	-	0.08	246	0.05	0.13	0.28	0.34	-0.64	Log	-1.28
