# Supplementary material for: Genome-Wide Association Study and Pathway-Level Analysis of Tocochromanol Levels in Maize Grain
Source: G3 (Bethesda). 2013 Aug 1;3(8):1287–99. doi: 10.1534/g3.113.006148 (PMC3737168; doi:10.1534/g3.113.006148)
Supplement: Supporting Information [file supp_g3.113.006148_TableS5.pdf]

**Table S5 Multi-locus mixed-model (MLMM) results from an analysis of αT, αT/γT, γT/(γT+αT), δT/αT, δT3/(γT3+αT3), and δT3.**

| Trait | SNP Added to Model | Chr | Position in RefGen_v2 | *P*-value | extBIC | R-square_LR[a] | R-square_LR of All Three SNPs[b] |
|---|---|---|---|---|---|---|---|
| αT | ss196416269 | 5 | 200,367,532 | 7.12E-16 | 384.47 | 0.21 | 0.45 |
| αT | S5_200369534 | 5 | 200,369,534 | 1.87E-14 | 353.57 | 0.19 | |
| αT | S5_200369481 | 5 | 200,369,481 | 3.04E-10 | 338.67 | 0.14 | |
| | | | | | | | |
| αT/γT | ss196416269 | 5 | 200,367,532 | 5.89E-15 | -402.01 | 0.21 | 0.40 |
| αT/γT | S5_200369534 | 5 | 200,369,534 | 4.08E-12 | -424.67 | 0.17 | |
| αT/γT | S5_200369481 | 5 | 200,369,481 | 1.92E-07 | -427.22 | 0.09 | |
| | | | | | | | |
| γT /(γT+αT) | ss196416269 | 5 | 200,367,532 | 7.40E-14 | -113.06 | 0.19 | 0.38 |
| γT /(γT+αT) | S5_200369481 | 5 | 200,369,481 | 3.25E-10 | -126.83 | 0.14 | |
| γT /(γT+αT) | S5_200369534 | 5 | 200,369,534 | 2.09E-09 | -138.31 | 0.12 | |
| | | | | | | | |
| δT/αT | ss196416269 | 5 | 200,367,532 | 2.29E-12 | 647.28 | 0.17 | 0.33 |
| δT/αT | S5_200369534 | 5 | 200,369,534 | 1.08E-08 | 630.30 | 0.11 | |
| δT/αT | S5_200369481 | 5 | 200,369,481 | 4.44E-07 | 619.70 | 0.09 | |
| | | | | | | | |
| δT3/ (γT3+αT3) | S5_133501858 | 5 | 133,501,858 | 7.16E-08 | -485.87 | 0.10 | |
| | | | | | | | |
| δT3 | S5_133333561 | 5 | 133,333,561 | 2.04E-07 | -170.44 | 0.09 | |

[a]Defined as the likelihood-ratio based $R^2$ statistic that measures the increase in phenotypic variation explained by adding the indicated SNP into the model.Table 1
[b]Defined as the likelihood-ratio based $R^2$ statistic that compares variation explained by the MLMM model with the three SNPs (indicated in Column A) to the intercept-only model.
