# Supplementary material for: Genome-Wide Association Study and Pathway-Level Analysis of Tocochromanol Levels in Maize Grain
Source: G3 (Bethesda). 2013 Aug 1;3(8):1287–99. doi: 10.1534/g3.113.006148 (PMC3737168; doi:10.1534/g3.113.006148)
Supplement: Supporting Information [file supp_g3.113.006148_TableS6.pdf]

Table S6a Haplotype effects of three ZmVTE4 SNPs identified with an optimal multi-locus mixed model (MLMM) for αT/γT.

Haplotype	ss196416269	S5_200369481	S5_200369534	Frequency	Mean Value	S.D.[a]
A,C,G	A	C	G	50	0.10	0.09
G,C,G	G	C	G	151	0.41	0.27
A,G,G	A	G	G	1	0.06	-
G,G,G	G	G	G	28	0.95	0.46
G,C,T	G	C	T	22	0.16	0.33
**R^2_{LR}[b]**	51.30%					
**Partial R^2_{LR}[c]**	40.47%					
***P*-value[d]**	5.55E-31					
**Maximum Fold Change[e]**	5.94					

[a]S.D., Standard deviation of the BLUPs for each haplotype.

[b]R^2_{LR}, likelihood-ratio based R^2 statistic, percentage of total phenotypic variation explained by the unified mixed model.

[c]Partial R^2_{LR}, likelihood-ratio based partial R^2 statistic, percentage of total phenotypic variation explained by the haplotypes.

[d]The P-value was from a unified mixed linear model that tested for an association between haplotypes and αT levels.

[e]Fold change was calculated as the ratio between the most favorable (G, G, G) and least favorable (G, C, T) haplotypes for αT levels.

Table S6b Haplotype effects of three ZmVTE4 SNPs identified with an optimal multi-locus mixed model (MLMM) for δT/αT.

**Haplotype**	**ss196416269**	**S5_200369481**	**S5_200369534**	**Frequency**	**Mean Value**	**S.D.**[a]
A,C,G	A	C	G	50	0.55	0.40
G,C,G	G	C	G	151	0.17	0.15
A,G,G	A	G	G	1	0.76	-
G,G,G	G	G	G	28	0.04	0.04
G,C,T	G	C	T	22	0.69	0.38
**R^2_{LR}**[b]	49.57%					
**Partial R^2_{LR}**[c]	39.96%					
***P*-value**[d]	2.16E-27					
**Maximum Fold Change**[e]	0.06					

[a]S.D., Standard deviation of the BLUPs for each haplotype.

[b]R^2_{LR}, likelihood-ratio based R^2 statistic, percentage of total phenotypic variation explained by the unified mixed model.

[c]Partial R^2_{LR}, likelihood-ratio based partial R^2 statistic, percentage of total phenotypic variation explained by the haplotypes.

[d]The P-value was from a unified mixed linear model that tested for an association between haplotypes and αT levels.

[e]Fold change was calculated as the ratio between the most favorable (G, G, G) and least favorable (G, C, T) haplotypes for αT levels.

Table S6c Haplotype effects of three ZmVTE4 SNPs identified with an optimal multi-locus mixed model (MLMM) for γT/(γT+αT).

Haplotype	ss196416269	S5_200369481	S5_200369534	Frequency	Mean Value	S.D.[a]
A,C,G	A	C	G	50	0.91	0.06
G,C,G	G	C	G	151	0.72	0.13
A,G,G	A	G	G	1	0.93	-
G,G,G	G	G	G	28	0.48	0.14
G,C,T	G	C	T	22	0.89	0.15
**R^2_{LR} [b]**	52.4%					
**Partial R^2_{LR} [c]**	41.3%					
***P*-value[d]**	2.16E-31					
**Maximum Fold Change[e]**	0.54					

[a]S.D., Standard deviation of the BLUPs for each haplotype.

[b]R^2_{LR}, likelihood-ratio based R^2 statistic, percentage of total phenotypic variation explained by the unified mixed model.

[c]Partial R^2_{LR}, likelihood-ratio based partial R^2 statistic, percentage of total phenotypic variation explained by the haplotypes.

[d]The P-value was from a unified mixed linear model that tested for an association between haplotypes and αT levels.

[e]Fold change was calculated as the ratio between the most favorable (G, G, G) and least favorable (G, C, T) haplotypes for αT levels.
