# Supplementary material for: Genome-Wide Association Study and Pathway-Level Analysis of Tocochromanol Levels in Maize Grain
Source: G3 (Bethesda). 2013 Aug 1;3(8):1287–99. doi: 10.1534/g3.113.006148 (PMC3737168; doi:10.1534/g3.113.006148)
Supplement: Supporting Information [file supp_g3.113.006148_TableS7.pdf]

Table S7a Statistically significant results from the candidate gene association study of 20 tocochromanol grain traits without and with none of the SNPs identified in the multi-locus mixed-model (MLMM) analysis included as covariates. SNPs that were were significantly associated with the indicated trait at 5% FDR are shown.

a priori candidate gene pathway	RefGen_v2 Gene ID	RefGen_v2 Annotated Gene Function	Trait	SNP ID	SNP Source	Chr	Position	Distance from Gene ORF Start	Distance from Gene ORF Finish	*P*-value	FDR Adjusted *P*-value	Minor Allele Frequency (MAF)	MAF Tropical (18% of 252 Lines)	MAF Temperate (82% of 252 Lines)	Sample Size	R^2_{LR} from Model without SNP	R^2_{LR} from Model with SNP	Effect Size	Lambda from Box-Cox Procedure	Back-Transformed Effect Estimates
Aromatic Head Group	GRMZM2G437912	prephenate dehydratase	Total Tocotrienols	S2_59013838	GBS	2	59,013,838	-23,405	-25,340	1.84E-05	3.76E-02	0.06	0.40	0.09	250	0.20	0.26	0.06	0.15	0.47
Aromatic Head Group	GRMZM2G437912	prephenate dehydratase	Total Tocotrienols	S2_59013840	GBS	2	59,013,840	-23,403	-25,338	1.84E-05	3.76E-02	0.06	0.40	0.09	250	0.20	0.26	-0.06	0.15	-0.34
Aromatic Head Group	GRMZM2G573867	3-dehydroquinate synthase	αT	S2_196293028	GBS	2	196,293,028	-66,599	-70,664	2.50E-04	3.97E-02	0.06	0.00	0.08	251	0.25	0.30	-0.26	0.40	-0.53
Aromatic Head Group	GRMZM2G573867	3-dehydroquinate synthase	αT/γT	S2_196295236	GBS	2	196,295,236	-64,391	-68,456	1.82E-04	4.63E-02	0.06	0.04	0.07	246	0.24	0.29	0.06	0.15	0.47
Aromatic Head Group	GRMZM2G573867	3-dehydroquinate synthase	γT/(γT+αT)	S2_196295236	GBS	2	196,295,236	-64,391	-68,456	2.59E-04	5.11E-02	0.06	0.04	0.07	251	0.29	0.33	-0.10	2.00	-0.05
Aromatic Head Group	GRMZM2G573867	3-dehydroquinate synthase	δT/αT	S2_196295236	GBS	2	196,295,236	-64,391	-68,456	1.36E-04	3.62E-02	0.06	0.04	0.07	246	0.28	0.32	-0.49	Log	-1.05
Aromatic Head Group	GRMZM2G573867	3-dehydroquinate synthase	δT/αT	S2_196387288	GBS	2	196,387,288	27,661	23,596	1.60E-04	3.89E-02	0.06	0.05	0.06	246	0.28	0.32	-0.49	Log	-1.05
Aromatic Head Group	GRMZM2G573867	3-dehydroquinate synthase	αT	S2_196514167	GBS	2	196,514,167	154,540	150,475	7.19E-06	2.20E-03	0.06	0.02	0.07	251	0.25	0.32	-0.36	0.40	-0.67
Aromatic Head Group	GRMZM2G573867	3-dehydroquinate synthase	δT/αT	S2_196514167	GBS	2	196,514,167	154,540	150,475	1.51E-04	3.82E-02	0.06	0.02	0.07	246	0.28	0.32	0.51	Log	1.81
Aromatic Head Group	GRMZM2G138624	isochorismatase hydrolase	αT3	ss196456226	55K	4	134,020,078	96,788	94,084	8.16E-06	2.49E-02	0.45	0.45	0.46	248	0.20	0.27	0.01	0.05	0.22
Aromatic Head Group	GRMZM2G124365	chorismate mutase	αT	S8_173041758	GBS	8	173,041,758	-61,455	-65,990	2.38E-04	3.97E-02	0.12	0.18	0.22	251	0.25	0.30	-0.23	0.40	-0.48
Aromatic Head Group	GRMZM2G124365	chorismate mutase	δT/αT	S8_173070782	GBS	8	173,070,782	-32,431	-36,966	1.37E-04	3.62E-02	0.18	0.04	0.33	246	0.28	0.32	-0.36	Log	-0.82
Aromatic Head Group	GRMZM2G124365	chorismate mutase	Total Tocotrienols	S8_173243067	GBS	8	173,243,067	139,854	135,319	6.12E-05	4.69E-02	0.09	0.20	0.09	250	0.20	0.26	0.05	0.15	0.38
Aromatic Head Group	GRMZM2G124365	chorismate mutase	αT3	S8_173243067	GBS	8	173,243,067	139,854	135,319	3.51E-05	3.57E-02	0.09	0.20	0.09	248	0.20	0.26	0.01	0.05	0.22
Prenyl Group Synthesis	GRMZM2G027059	4-hydroxy-3-methylbut-2-enyldiphosphate reductase	δT3	ss196519818	55K	1	272,871,696	-65,140	-68,806	1.40E-04	4.28E-02	0.32	0.09	0.38	247	0.12	0.17	-0.06	-0.20	0.36
Prenyl Group Synthesis	GRMZM2G027059	4-hydroxy-3-methylbut-2-enyldiphosphate reductase	δT3	ss196519822	55K	1	272,874,639	-62,197	-65,863	1.30E-04	4.18E-02	0.33	0.09	0.38	247	0.12	0.17	-0.06	-0.20	0.36
Prenyl Group Synthesis	GRMZM2G027059	4-hydroxy-3-methylbut-2-enyldiphosphate reductase	αT	S1_273098217	GBS	1	273,098,217	161,381	157,715	3.82E-05	8.98E-03	0.22	0.24	0.24	251	0.25	0.31	-0.20	0.40	-0.43
Prenyl Group Synthesis	GRMZM2G137409	hydroxymethylbutenyl 4-diphosphate synthase	δT3/γT3	S5_182040396	GBS	5	182,040,396	-83,609	-90,235	7.94E-05	3.73E-02	0.29	0.00	0.37	249	0.13	0.18	0.01	0.05	0.22
Prenyl Group Synthesis	AC209374.4_FG002	2-C-methyl-D-erythritol 2,4-cyclodiphosphate synthase	αT/γT	S5_196278264	GBS	5	196,278,264	-1,031	-2,773	9.54E-05	2.64E-02	0.25	0.17	0.30	246	0.24	0.29	0.04	0.15	0.3
Prenyl Group Synthesis	AC209374.4_FG002	2-C-methyl-D-erythritol 2,4-cyclodiphosphate synthase	αT	S5_196278264	GBS	5	196,278,264	-1,031	-2,773	1.31E-04	2.76E-02	0.25	0.17	0.30	251	0.25	0.30	0.19	0.40	0.54
Prenyl Group Synthesis	AC209374.4_FG002	2-C-methyl-D-erythritol 2,4-cyclodiphosphate synthase	γT/(γT+αT)	S5_196278264	GBS	5	196,278,264	-1,031	-2,773	4.51E-05	1.45E-02	0.25	0.17	0.30	251	0.29	0.34	-0.07	2.00	-0.04
Prenyl Group Synthesis	AC209374.4_FG002	2-C-methyl-D-erythritol 2,4-cyclodiphosphate synthase	δT/αT	S5_196278264	GBS	5	196,278,264	-1,031	-2,773	2.72E-05	1.03E-02	0.24	0.17	0.30	246	0.28	0.33	-0.36	Log	-0.82
Prenyl Group Synthesis	AC209374.4_FG002	2-C-methyl-D-erythritol 2,4-cyclodiphosphate synthase	γT/(γT+αT)	S5_196508694	GBS	5	196,508,694	229,399	227,657	2.41E-04	5.08E-02	0.41	0.43	0.45	251	0.29	0.33	0.05	2.00	0.02
Prenyl Group Synthesis	GRMZM2G133082	isopentenyl pyrophosphate isomerase	γT/(γT+αT)	S6_147333828	GBS	6	147,333,828	202,712	197,149	1.43E-04	3.25E-02	0.07	0.22	0.05	251	0.29	0.33	-0.11	2.00	-0.06
Prenyl Group Synthesis	GRMZM2G133082	isopentenyl pyrophosphate isomerase	γT/(γT+αT)	S6_147333833	GBS	6	147,333,833	202,717	197,154	1.43E-04	3.25E-02	0.07	0.22	0.05	251	0.29	0.33	-0.11	2.00	-0.06
Prenyl Group Synthesis	GRMZM2G493395	1-deoxy-D-xylulose 5-phosphate synthase	αT/γT	ss196475603	55K	7	13,959,219	-118,633	-121,856	1.25E-05	3.81E-03	0.30	0.37	0.30	246	0.24	0.31	0.04	0.15	0.3
Prenyl Group Synthesis	GRMZM2G493395	1-deoxy-D-xylulose 5-phosphate synthase	γT/(γT+αT)	ss196475603	55K	7	13,959,219	-118,633	-121,856	4.10E-05	1.45E-02	0.31	0.37	0.30	251	0.29	0.34	-0.07	2.00	-0.04
Prenyl Group Synthesis	GRMZM2G172032	2-C-methyl-D-erythritol 4-phosphate cytidyltransferase	δT3/(γT3+αT3)	S8_164626861	GBS	8	164,626,861	-122,078	-125,510	5.26E-05	1.40E-02	0.14	0.50	0.10	248	0.10	0.16	-0.04	-0.10	0.5
Prenyl Group Synthesis	GRMZM2G172032	2-C-methyl-D-erythritol 4-phosphate cytidyltransferase	δT3	S8_164626861	GBS	8	164,626,861	-122,078	-125,510	1.94E-04	4.60E-02	0.14	0.50	0.10	247	0.12	0.17	-0.07	-0.20	0.44
Tocochromanol Pathway	GRMZM2G009785	tocopherol cyclase	δT3/(γT3+αT3)	S5_133331094	GBS	5	133,331,094	-170,834	-187,401	3.48E-05	1.04E-02	0.41	0.47	0.41	248	0.10	0.16	0.03	-0.10	-0.26
Tocochromanol Pathway	GRMZM2G009785	tocopherol cyclase	δT3/αT3	S5_133331094	GBS	5	133,331,094	-170,834	-187,401	4.92E-05	3.33E-02	0.41	0.47	0.41	246	0.11	0.18	0.05	-0.15	-0.28
Tocochromanol	GRMZM2G009785	tocopherol cyclase	δT3	S5_133331094	GBS	5	133,331,094	-170,834	-187,401	4.29E-06	3.27E-03	0.41	0.47	0.41	247	0.12	0.20	0.06	-0.20	-0.25

Pathway																				
Tocochromanol Pathway	GRMZM2G009785	tocopherol cyclase	δT3/(γT3+αT3)	S5_133331096	GBS	5	133,331,096	-170,832	-187,399	1.54E-05	5.53E-03	0.40	0.44	0.41	248	0.10	0.17	-0.03	-0.10	0.36
Tocochromanol Pathway	GRMZM2G009785	tocopherol cyclase	δT3/αT3	S5_133331096	GBS	5	133,331,096	-170,832	-187,399	1.92E-05	2.63E-02	0.40	0.44	0.41	246	0.11	0.18	-0.05	-0.15	0.41
Tocochromanol Pathway	GRMZM2G009785	tocopherol cyclase	δT3	S5_133331096	GBS	5	133,331,096	-170,832	-187,399	1.01E-06	1.54E-03	0.40	0.44	0.41	247	0.12	0.21	-0.06	-0.20	0.36
Tocochromanol Pathway	GRMZM2G009785	tocopherol cyclase	δT3/(γT3+αT3)	S5_133331106	GBS	5	133,331,106	-170,822	-187,389	1.54E-05	5.53E-03	0.40	0.47	0.41	248	0.10	0.17	0.03	-0.10	-0.26
Tocochromanol Pathway	GRMZM2G009785	tocopherol cyclase	δT3/αT3	S5_133331106	GBS	5	133,331,106	-170,822	-187,389	1.92E-05	2.63E-02	0.40	0.47	0.41	246	0.11	0.18	0.05	-0.15	-0.28
Tocochromanol Pathway	GRMZM2G009785	tocopherol cyclase	δT3	S5_133331106	GBS	5	133,331,106	-170,822	-187,389	1.01E-06	1.54E-03	0.40	0.47	0.41	247	0.12	0.21	0.06	-0.20	-0.25
Tocochromanol Pathway	GRMZM2G009785	tocopherol cyclase	δT3/(γT3+αT3)	ss196465630	55K	5	133,332,323	-169,605	-186,172	6.13E-05	1.56E-02	0.32	0.11	0.36	248	0.10	0.16	-0.03	-0.10	0.36
Tocochromanol Pathway	GRMZM2G009785	tocopherol cyclase	δT3	ss196465630	55K	5	133,332,323	-169,605	-186,172	1.96E-04	4.60E-02	0.32	0.11	0.36	247	0.12	0.17	-0.05	-0.20	0.29
Tocochromanol Pathway	GRMZM2G009785	tocopherol cyclase	δT3/(γT3+αT3)	ss196465628	55K	5	133,333,095	-168,833	-185,400	4.22E-05	1.17E-02	0.42	0.47	0.42	248	0.10	0.16	-0.03	-0.10	0.36
Tocochromanol Pathway	GRMZM2G009785	tocopherol cyclase	δT3/αT3	ss196465628	55K	5	133,333,095	-168,833	-185,400	8.98E-05	4.56E-02	0.42	0.47	0.42	246	0.11	0.17	-0.05	-0.15	0.41
Tocochromanol Pathway	GRMZM2G009785	tocopherol cyclase	δT3	ss196465628	55K	5	133,333,095	-168,833	-185,400	3.88E-06	3.27E-03	0.42	0.47	0.42	247	0.12	0.20	-0.06	-0.20	0.36
Tocochromanol Pathway	GRMZM2G009785	tocopherol cyclase	δT3/(γT3+αT3)	S5_133333397	GBS	5	133,333,397	-168,531	-185,098	6.13E-06	3.89E-03	0.43	0.47	0.43	248	0.10	0.18	0.03	-0.10	-0.26
Tocochromanol Pathway	GRMZM2G009785	tocopherol cyclase	δT3/(γT3+αT3)	ss196465626	55K	5	133,333,397	-168,531	-185,098	2.46E-05	8.34E-03	0.42	0.47	0.42	248	0.10	0.17	0.03	-0.10	-0.26
Tocochromanol Pathway	GRMZM2G009785	tocopherol cyclase	δT3/αT3	S5_133333397	GBS	5	133,333,397	-168,531	-185,098	2.16E-05	2.63E-02	0.43	0.47	0.43	246	0.11	0.18	0.05	-0.15	-0.28
Tocochromanol Pathway	GRMZM2G009785	tocopherol cyclase	δT3/αT3	ss196465626	55K	5	133,333,397	-168,531	-185,098	6.02E-05	3.66E-02	0.42	0.47	0.42	246	0.11	0.17	0.05	-0.15	-0.28
Tocochromanol Pathway	GRMZM2G009785	tocopherol cyclase	δT3	S5_133333397	GBS	5	133,333,397	-168,531	-185,098	3.06E-07	9.53E-04	0.43	0.47	0.43	247	0.12	0.22	0.06	-0.20	-0.25
Tocochromanol Pathway	GRMZM2G009785	tocopherol cyclase	δT3	ss196465626	55K	5	133,333,397	-168,531	-185,098	1.69E-06	1.72E-03	0.42	0.47	0.42	247	0.12	0.21	0.06	-0.20	-0.25
Tocochromanol Pathway	GRMZM2G009785	tocopherol cyclase	δT3/(γT3+αT3)	S5_133333561	GBS	5	133,333,561	-168,367	-184,934	8.28E-06	3.89E-03	0.42	0.48	0.43	248	0.10	0.17	0.03	-0.10	-0.26
Tocochromanol Pathway	GRMZM2G009785	tocopherol cyclase	δT3/αT3	S5_133333561	GBS	5	133,333,561	-168,367	-184,934	2.12E-05	2.63E-02	0.42	0.48	0.43	246	0.11	0.18	0.05	-0.15	-0.28
Tocochromanol Pathway	GRMZM2G009785	tocopherol cyclase	δT3	S5_133333561	GBS	5	133,333,561	-168,367	-184,934	3.12E-07	9.53E-04	0.42	0.48	0.43	247	0.12	0.22	0.06	-0.20	-0.25
Tocochromanol Pathway	GRMZM2G009785	tocopherol cyclase	δT3/(γT3+αT3)	S5_133335078	GBS	5	133,335,078	-166,850	-183,417	1.48E-05	5.53E-03	0.30	0.33	0.29	248	0.10	0.17	-0.03	-0.10	0.36
Tocochromanol Pathway	GRMZM2G009785	tocopherol cyclase	δT3/αT3	S5_133335078	GBS	5	133,335,078	-166,850	-183,417	8.11E-05	4.49E-02	0.30	0.33	0.29	246	0.11	0.17	-0.05	-0.15	0.41
Tocochromanol Pathway	GRMZM2G009785	tocopherol cyclase	δT3	S5_133335078	GBS	5	133,335,078	-166,850	-183,417	1.34E-06	1.64E-03	0.30	0.33	0.29	247	0.12	0.21	-0.06	-0.20	0.36
Tocochromanol Pathway	GRMZM2G009785	tocopherol cyclase	δT3/(γT3+αT3)	S5_133338747	GBS	5	133,338,747	-163,181	-179,748	2.78E-05	8.94E-03	0.37	0.17	0.41	248	0.10	0.16	-0.03	-0.10	0.36
Tocochromanol Pathway	GRMZM2G009785	tocopherol cyclase	δT3/γT3	S5_133338747	GBS	5	133,338,747	-163,181	-179,748	6.52E-05	3.73E-02	0.37	0.17	0.41	249	0.13	0.18	0.01	0.05	0.22
Tocochromanol Pathway	GRMZM2G009785	tocopherol cyclase	δT3	S5_133338747	GBS	5	133,338,747	-163,181	-179,748	1.75E-04	4.60E-02	0.37	0.17	0.41	247	0.12	0.17	-0.05	-0.20	0.29
Tocochromanol Pathway	GRMZM2G009785	tocopherol cyclase	δT/γT	PZA00524.2	4K	5	133,338,936	-162,992	-179,559	1.36E-05	3.93E-02	0.41	0.23	0.39	249	0.13	0.20	-0.02	0.30	-0.07
Tocochromanol Pathway	GRMZM2G009785	tocopherol cyclase	δT/γT	S5_133338936	GBS	5	133,338,936	-162,992	-179,559	1.47E-05	3.93E-02	0.47	0.17	0.40	249	0.13	0.20	0.02	0.30	0.07
Tocochromanol Pathway	GRMZM2G009785	tocopherol cyclase	δT3/(γT3+αT3)	S5_133499169	GBS	5	133,499,169	-2,759	-19,326	1.77E-06	2.71E-03	0.39	0.15	0.44	248	0.10	0.19	-0.03	-0.10	0.36
Tocochromanol Pathway	GRMZM2G009785	tocopherol cyclase	δT3/αT3	S5_133499169	GBS	5	133,499,169	-2,759	-19,326	4.65E-05	3.33E-02	0.39	0.15	0.44	246	0.11	0.18	-0.06	-0.15	0.51
Tocochromanol Pathway	GRMZM2G009785	tocopherol cyclase	δT3/γT3	S5_133499169	GBS	5	133,499,169	-2,759	-19,326	1.04E-04	4.54E-02	0.40	0.15	0.44	249	0.13	0.18	0.01	0.05	0.22
Tocochromanol Pathway	GRMZM2G009785	tocopherol cyclase	δT3	S5_133499169	GBS	5	133,499,169	-2,759	-19,326	1.13E-04	4.07E-02	0.40	0.15	0.44	247	0.12	0.18	-0.05	-0.20	0.29
Tocochromanol Pathway	GRMZM2G009785	tocopherol cyclase	δT3/(γT3+αT3)	S5_133499269	GBS	5	133,499,269	-2,659	-19,226	3.66E-06	3.89E-03	0.39	0.18	0.46	248	0.10	0.18	0.03	-0.10	-0.26
Tocochromanol Pathway	GRMZM2G009785	tocopherol cyclase	δT3	S5_133499269	GBS	5	133,499,269	-2,659	-19,226	1.78E-04	4.60E-02	0.40	0.18	0.46	247	0.12	0.17	0.05	-0.20	-0.22
Tocochromanol Pathway	GRMZM2G009785	tocopherol cyclase	δT3/(γT3+αT3)	S5_133501858	GBS	5	133,501,858	-70	-16,637	1.29E-07	7.88E-04	0.40	0.19	0.46	248	0.10	0.21	0.04	-0.10	-0.32
Tocochromanol Pathway	GRMZM2G009785	tocopherol cyclase	δT3/αT3	S5_133501858	GBS	5	133,501,858	-70	-16,637	1.09E-05	2.63E-02	0.40	0.19	0.46	246	0.11	0.19	0.06	-0.15	-0.32

Tocochromanol Pathway	GRMZM2G009785	tocopherol cyclase	δT3/γT3	S5_133501858	GBS	5	133,501,858	-70	-16,637	1.67E-06	9.96E-03	0.39	0.19	0.46	249	0.13	0.21	-0.01	0.05	-0.18
Tocochromanol Pathway	GRMZM2G009785	tocopherol cyclase	δT3	S5_133501858	GBS	5	133,501,858	-70	-16,637	6.01E-06	4.08E-03	0.40	0.19	0.46	247	0.12	0.20	0.06	-0.20	-0.25
Tocochromanol Pathway	GRMZM2G009785	tocopherol cyclase	δT3/(γT3+αT3)	S5_133501992	GBS	5	133,501,992	64	-16,503	9.72E-07	1.98E-03	0.40	0.14	0.46	248	0.10	0.19	0.03	-0.10	-0.26
Tocochromanol Pathway	GRMZM2G009785	tocopherol cyclase	δT3/αT3	S5_133501992	GBS	5	133,501,992	64	-16,503	3.55E-05	3.17E-02	0.41	0.14	0.46	246	0.11	0.18	0.06	-0.15	-0.32
Tocochromanol Pathway	GRMZM2G009785	tocopherol cyclase	δT3/γT3	S5_133501992	GBS	5	133,501,992	64	-16,503	4.89E-06	9.96E-03	0.40	0.14	0.46	249	0.13	0.20	-0.01	0.05	-0.18
Tocochromanol Pathway	GRMZM2G009785	tocopherol cyclase	δT3	S5_133501992	GBS	5	133,501,992	64	-16,503	1.36E-05	7.57E-03	0.40	0.14	0.46	247	0.12	0.19	0.06	-0.20	-0.25
Tocochromanol Pathway	GRMZM2G009785	tocopherol cyclase	δT3/(γT3+αT3)	PZB00969.1	4K	5	133,502,506	578	-15,989	8.27E-07	1.98E-03	0.41	0.14	0.46	248	0.10	0.19	0.03	-0.10	-0.26
Tocochromanol Pathway	GRMZM2G009785	tocopherol cyclase	δT3/(γT3+αT3)	ss196416168	55K	5	133,502,506	578	-15,989	1.30E-05	5.53E-03	0.40	0.13	0.46	248	0.10	0.17	0.03	-0.10	-0.26
Tocochromanol Pathway	GRMZM2G009785	tocopherol cyclase	δT3/αT3	PZB00969.1	4K	5	133,502,506	578	-15,989	3.64E-05	3.17E-02	0.41	0.14	0.46	246	0.11	0.18	0.06	-0.15	-0.32
Tocochromanol Pathway	GRMZM2G009785	tocopherol cyclase	δT3/γT3	PZB00969.1	4K	5	133,502,506	578	-15,989	3.32E-06	9.96E-03	0.41	0.14	0.46	249	0.13	0.21	-0.01	0.05	-0.18
Tocochromanol Pathway	GRMZM2G009785	tocopherol cyclase	δT3/γT3	ss196416168	55K	5	133,502,506	578	-15,989	4.29E-05	2.91E-02	0.40	0.13	0.46	249	0.13	0.19	-0.01	0.05	-0.18
Tocochromanol Pathway	GRMZM2G009785	tocopherol cyclase	δT3	PZB00969.1	4K	5	133,502,506	578	-15,989	1.17E-05	7.17E-03	0.41	0.14	0.46	247	0.12	0.19	0.06	-0.20	-0.25
Tocochromanol Pathway	GRMZM2G009785	tocopherol cyclase	δT3	ss196416168	55K	5	133,502,506	578	-15,989	1.29E-04	4.18E-02	0.40	0.13	0.46	247	0.12	0.17	0.05	-0.20	-0.22
Tocochromanol Pathway	GRMZM2G009785	tocopherol cyclase	δT3/(γT3+αT3)	ss196465634	55K	5	133,510,613	8,685	-7,882	7.54E-06	3.89E-03	0.41	0.14	0.46	248	0.10	0.17	-0.03	-0.10	0.36
Tocochromanol Pathway	GRMZM2G009785	tocopherol cyclase	δT3/γT3	ss196465634	55K	5	133,510,613	8,685	-7,882	1.92E-05	2.00E-02	0.41	0.14	0.46	249	0.13	0.19	0.01	0.05	0.22
Tocochromanol Pathway	GRMZM2G009785	tocopherol cyclase	δT3	ss196465634	55K	5	133,510,613	8,685	-7,882	8.23E-05	3.86E-02	0.41	0.14	0.46	247	0.12	0.18	-0.05	-0.20	0.29
Tocochromanol Pathway	GRMZM2G009785	tocopherol cyclase	δT3/(γT3+αT3)	PZB02491.1	4K	5	133,517,065	15,137	-1,430	6.69E-06	3.89E-03	0.40	0.14	0.46	248	0.10	0.18	-0.03	-0.10	0.36
Tocochromanol Pathway	GRMZM2G009785	tocopherol cyclase	δT3/γT3	PZB02491.1	4K	5	133,517,065	15,137	-1,430	7.44E-05	3.73E-02	0.40	0.14	0.46	249	0.13	0.18	0.01	0.05	0.22
Tocochromanol Pathway	GRMZM2G009785	tocopherol cyclase	δT3	PZB02491.1	4K	5	133,517,065	15,137	-1,430	4.86E-05	2.48E-02	0.40	0.14	0.46	247	0.12	0.18	-0.05	-0.20	0.29
Tocochromanol Pathway	GRMZM2G009785	tocopherol cyclase	δT3/(γT3+αT3)	S5_133618308	GBS	5	133,618,308	116,380	99,813	7.89E-06	3.89E-03	0.40	0.10	0.48	248	0.10	0.17	0.03	-0.10	-0.26
Tocochromanol Pathway	GRMZM2G009785	tocopherol cyclase	δT3/γT3	S5_133618308	GBS	5	133,618,308	116,380	99,813	2.29E-05	2.00E-02	0.40	0.10	0.48	249	0.13	0.19	-0.01	0.05	-0.18
Tocochromanol Pathway	GRMZM2G009785	tocopherol cyclase	δT3	S5_133618308	GBS	5	133,618,308	116,380	99,813	1.05E-04	4.02E-02	0.40	0.10	0.48	247	0.12	0.18	0.05	-0.20	-0.22
Tocochromanol Pathway	GRMZM2G009785	tocopherol cyclase	δT3/(γT3+αT3)	S5_133618309	GBS	5	133,618,309	116,381	99,814	7.89E-06	3.89E-03	0.40	0.10	0.48	248	0.10	0.17	0.03	-0.10	-0.26
Tocochromanol Pathway	GRMZM2G009785	tocopherol cyclase	δT3/γT3	S5_133618309	GBS	5	133,618,309	116,381	99,814	2.29E-05	2.00E-02	0.40	0.10	0.48	249	0.13	0.19	-0.01	0.05	-0.18
Tocochromanol Pathway	GRMZM2G009785	tocopherol cyclase	δT3	S5_133618309	GBS	5	133,618,309	116,381	99,814	1.05E-04	4.02E-02	0.40	0.10	0.48	247	0.12	0.18	0.05	-0.20	-0.22
Tocochromanol Pathway	GRMZM2G009785	tocopherol cyclase	δT3/(γT3+αT3)	S5_133618344	GBS	5	133,618,344	116,416	99,849	7.89E-06	3.89E-03	0.40	0.10	0.48	248	0.10	0.17	0.03	-0.10	-0.26
Tocochromanol Pathway	GRMZM2G009785	tocopherol cyclase	δT3/γT3	S5_133618344	GBS	5	133,618,344	116,416	99,849	2.29E-05	2.00E-02	0.40	0.10	0.48	249	0.13	0.19	-0.01	0.05	-0.18
Tocochromanol Pathway	GRMZM2G009785	tocopherol cyclase	δT3	S5_133618344	GBS	5	133,618,344	116,416	99,849	1.05E-04	4.02E-02	0.40	0.10	0.48	247	0.12	0.18	0.05	-0.20	-0.22
Tocochromanol Pathway	GRMZM2G009785	tocopherol cyclase	δT/γT	S5_133618788	GBS	5	133,618,788	116,860	100,293	2.56E-05	3.93E-02	0.49	0.11	0.42	249	0.13	0.19	0.02	0.30	0.07
Tocochromanol Pathway	GRMZM2G009785	tocopherol cyclase	δT3/(γT3+αT3)	S5_133618788	GBS	5	133,618,788	116,860	100,293	1.42E-04	3.35E-02	0.49	0.11	0.42	248	0.10	0.15	-0.03	-0.10	0.36
Tocochromanol Pathway	GRMZM2G009785	tocopherol cyclase	δT/γT	S5_133618810	GBS	5	133,618,810	116,882	100,315	2.56E-05	3.93E-02	0.49	0.11	0.42	249	0.13	0.19	0.02	0.30	0.07
Tocochromanol Pathway	GRMZM2G009785	tocopherol cyclase	δT3/(γT3+αT3)	S5_133618810	GBS	5	133,618,810	116,882	100,315	1.42E-04	3.35E-02	0.49	0.11	0.42	248	0.10	0.15	-0.03	-0.10	0.36
Tocochromanol Pathway	GRMZM2G009785	tocopherol cyclase	δT3/(γT3+αT3)	S5_133691297	GBS	5	133,691,297	189,369	172,802	5.02E-06	3.89E-03	0.37	0.10	0.46	248	0.10	0.18	0.03	-0.10	-0.26
Tocochromanol Pathway	GRMZM2G009785	tocopherol cyclase	δT3/γT3	S5_133691297	GBS	5	133,691,297	189,369	172,802	3.04E-05	2.32E-02	0.37	0.10	0.46	249	0.13	0.19	-0.01	0.05	-0.18
Tocochromanol Pathway	GRMZM2G009785	tocopherol cyclase	δT3	S5_133691297	GBS	5	133,691,297	189,369	172,802	1.59E-04	4.60E-02	0.37	0.10	0.46	247	0.12	0.17	0.05	-0.20	-0.22
Tocochromanol Pathway	GRMZM2G009785	tocopherol cyclase	δT3/(γT3+αT3)	ss196465642	55K	5	133,728,050	226,122	209,555	3.56E-05	1.04E-02	0.40	0.11	0.46	248	0.10	0.16	-0.03	-0.10	0.36
Tocochromanol Pathway	GRMZM2G009785	tocopherol cyclase	δT3	ss196465642	55K	5	133,728,050	226,122	209,555	1.85E-04	4.60E-02	0.40	0.11	0.46	247	0.12	0.17	-0.05	-0.20	0.29

Tocochromanol Pathway	GRMZM2G035213	γ-tocopherol methyltransferase	αT	S5_200117524	GBS	5	200,117,524	-249,505	-253,327	3.44E-05	8.42E-03	0.21	0.02	0.27	251	0.25	0.31	0.22	0.40	0.64
Tocochromanol Pathway	GRMZM2G035213	γ-tocopherol methyltransferase	αT	S5_200117526	GBS	5	200,117,526	-249,503	-253,325	1.93E-04	3.58E-02	0.22	0.07	0.27	251	0.25	0.30	0.20	0.40	0.58
Tocochromanol Pathway	GRMZM2G035213	γ-tocopherol methyltransferase	αT	S5_200117632	GBS	5	200,117,632	-249,397	-253,219	2.53E-04	3.97E-02	0.21	0.07	0.26	251	0.25	0.30	-0.19	0.40	-0.41
Tocochromanol Pathway	GRMZM2G035213	γ-tocopherol methyltransferase	αT	S5_200119597	GBS	5	200,119,597	-247,432	-251,254	1.71E-04	3.33E-02	0.33	0.18	0.36	251	0.25	0.30	0.17	0.40	0.48
Tocochromanol Pathway	GRMZM2G035213	γ-tocopherol methyltransferase	αT	S5_200119623	GBS	5	200,119,623	-247,406	-251,228	2.45E-04	3.97E-02	0.35	0.27	0.37	251	0.25	0.30	0.16	0.40	0.45
Tocochromanol Pathway	GRMZM2G035213	γ-tocopherol methyltransferase	γT/(γT+αT)	S5_200130689	GBS	5	200,130,689	-236,340	-240,162	4.45E-05	1.45E-02	0.08	0.33	0.08	251	0.29	0.34	0.12	2.00	0.06
Tocochromanol Pathway	GRMZM2G035213	γ-tocopherol methyltransferase	γT/(γT+αT)	S5_200292465	GBS	5	200,292,465	-74,564	-78,386	5.52E-05	1.69E-02	0.34	0.34	0.28	251	0.29	0.33	-0.06	2.00	-0.03
Tocochromanol Pathway	GRMZM2G035213	γ-tocopherol methyltransferase	αT/γT	S5_200293693	GBS	5	200,293,693	-73,336	-77,158	8.10E-06	2.90E-03	0.40	0.34	0.37	246	0.24	0.31	0.04	0.15	0.3
Tocochromanol Pathway	GRMZM2G035213	γ-tocopherol methyltransferase	αT	S5_200293693	GBS	5	200,293,693	-73,336	-77,158	3.33E-06	1.20E-03	0.41	0.34	0.37	251	0.25	0.32	0.20	0.40	0.58
Tocochromanol Pathway	GRMZM2G035213	γ-tocopherol methyltransferase	γT/(γT+αT)	S5_200293693	GBS	5	200,293,693	-73,336	-77,158	1.24E-06	6.88E-04	0.42	0.34	0.37	251	0.29	0.36	-0.07	2.00	-0.04
Tocochromanol Pathway	GRMZM2G035213	γ-tocopherol methyltransferase	δT/αT	S5_200293693	GBS	5	200,293,693	-73,336	-77,158	3.00E-05	1.07E-02	0.42	0.34	0.37	246	0.28	0.33	-0.31	Log	-0.72
Tocochromanol Pathway	GRMZM2G035213	γ-tocopherol methyltransferase	αT	ss196468356	55K	5	200,300,836	-66,193	-70,015	9.29E-05	2.11E-02	0.43	0.20	0.49	251	0.25	0.30	-0.17	0.40	-0.37
Tocochromanol Pathway	GRMZM2G035213	γ-tocopherol methyltransferase	γT/(γT+αT)	ss196468356	55K	5	200,300,836	-66,193	-70,015	2.50E-04	5.09E-02	0.43	0.20	0.49	251	0.29	0.33	0.06	2.00	0.03
Tocochromanol Pathway	GRMZM2G035213	γ-tocopherol methyltransferase	δT/αT	ss196468356	55K	5	200,300,836	-66,193	-70,015	6.33E-06	3.21E-03	0.43	0.20	0.49	246	0.28	0.34	0.33	Log	1.06
Tocochromanol Pathway	GRMZM2G035213	γ-tocopherol methyltransferase	αT/γT	S5_200318615	GBS	5	200,318,615	-48,414	-52,236	2.50E-06	1.17E-03	0.22	0.27	0.44	246	0.24	0.32	0.05	0.15	0.38
Tocochromanol Pathway	GRMZM2G035213	γ-tocopherol methyltransferase	αT	S5_200318615	GBS	5	200,318,615	-48,414	-52,236	1.05E-06	4.57E-04	0.22	0.27	0.44	251	0.25	0.33	0.25	0.40	0.75
Tocochromanol Pathway	GRMZM2G035213	γ-tocopherol methyltransferase	γT/(γT+αT)	S5_200318615	GBS	5	200,318,615	-48,414	-52,236	2.03E-05	8.29E-03	0.22	0.27	0.44	251	0.29	0.34	-0.08	2.00	-0.04
Tocochromanol Pathway	GRMZM2G035213	γ-tocopherol methyltransferase	δT/αT	S5_200318615	GBS	5	200,318,615	-48,414	-52,236	8.89E-05	2.70E-02	0.21	0.27	0.44	246	0.28	0.33	-0.35	Log	-0.8
Tocochromanol Pathway	GRMZM2G035213	γ-tocopherol methyltransferase	αT/γT	PZB02283.1	4K	5	200,367,532	503	-3,319	9.21E-13	1.87E-09	0.21	0.14	0.22	246	0.24	0.42	0.08	0.15	0.67
Tocochromanol Pathway	GRMZM2G035213	γ-tocopherol methyltransferase	αT/γT	ss196416269	55K	5	200,367,532	503	-3,319	9.21E-13	1.87E-09	0.21	0.13	0.22	246	0.24	0.42	0.08	0.15	0.67
Tocochromanol Pathway	GRMZM2G035213	γ-tocopherol methyltransferase	αT	PZB02283.1	4K	5	200,367,532	503	-3,319	7.36E-14	1.50E-10	0.20	0.14	0.22	251	0.25	0.44	0.40	0.40	1.32
Tocochromanol Pathway	GRMZM2G035213	γ-tocopherol methyltransferase	αT	ss196416269	55K	5	200,367,532	503	-3,319	7.36E-14	1.50E-10	0.20	0.13	0.22	251	0.25	0.44	0.40	0.40	1.32
Tocochromanol Pathway	GRMZM2G035213	γ-tocopherol methyltransferase	γT/(γT+αT)	PZB02283.1	4K	5	200,367,532	503	-3,319	4.82E-12	9.83E-09	0.20	0.14	0.22	251	0.29	0.44	-0.13	2.00	-0.07
Tocochromanol Pathway	GRMZM2G035213	γ-tocopherol methyltransferase	γT/(γT+αT)	ss196416269	55K	5	200,367,532	503	-3,319	4.82E-12	9.83E-09	0.20	0.13	0.22	251	0.29	0.44	-0.13	2.00	-0.07
Tocochromanol Pathway	GRMZM2G035213	γ-tocopherol methyltransferase	δT/αT	PZB02283.1	4K	5	200,367,532	503	-3,319	1.88E-11	3.80E-08	0.20	0.14	0.22	246	0.28	0.42	-0.63	Log	-1.27
Tocochromanol Pathway	GRMZM2G035213	γ-tocopherol methyltransferase	δT/αT	ss196416269	55K	5	200,367,532	503	-3,319	1.88E-11	3.80E-08	0.20	0.13	0.22	246	0.28	0.42	-0.63	Log	-1.27
Tocochromanol Pathway	GRMZM2G035213	γ-tocopherol methyltransferase	αT/γT	ss196468362	55K	5	200,369,124	2,095	-1,727	9.21E-13	1.87E-09	0.21	0.13	0.22	246	0.24	0.42	0.08	0.15	0.67
Tocochromanol Pathway	GRMZM2G035213	γ-tocopherol methyltransferase	αT	ss196468362	55K	5	200,369,124	2,095	-1,727	7.36E-14	1.50E-10	0.20	0.13	0.22	251	0.25	0.44	0.40	0.40	1.32
Tocochromanol Pathway	GRMZM2G035213	γ-tocopherol methyltransferase	γT/(γT+αT)	ss196468362	55K	5	200,369,124	2,095	-1,727	4.82E-12	9.83E-09	0.20	0.13	0.22	251	0.29	0.44	-0.13	2.00	-0.07
Tocochromanol Pathway	GRMZM2G035213	γ-tocopherol methyltransferase	δT/αT	ss196468362	55K	5	200,369,124	2,095	-1,727	1.88E-11	3.80E-08	0.20	0.13	0.22	246	0.28	0.42	-0.63	Log	-1.27
Tocochromanol Pathway	GRMZM2G035213	γ-tocopherol methyltransferase	αT/γT	S5_200369481	GBS	5	200,369,481	2,452	-1,370	7.24E-07	4.01E-04	0.10	0.36	0.09	246	0.24	0.32	0.07	0.15	0.57
Tocochromanol Pathway	GRMZM2G035213	γ-tocopherol methyltransferase	αT	S5_200369481	GBS	5	200,369,481	2,452	-1,370	1.97E-08	2.00E-05	0.11	0.36	0.09	251	0.25	0.35	0.38	0.40	1.24
Tocochromanol Pathway	GRMZM2G035213	γ-tocopherol methyltransferase	γT/(γT+αT)	S5_200369481	GBS	5	200,369,481	2,452	-1,370	6.15E-09	7.53E-06	0.12	0.36	0.09	251	0.29	0.39	-0.14	2.00	-0.07
Tocochromanol Pathway	GRMZM2G035213	γ-tocopherol methyltransferase	δT/αT	S5_200369481	GBS	5	200,369,481	2,452	-1,370	7.21E-07	5.35E-04	0.12	0.36	0.09	246	0.28	0.35	-0.56	Log	-1.17
Tocochromanol Pathway	GRMZM2G035213	γ-tocopherol methyltransferase	αT/γT	S5_200369508	GBS	5	200,369,508	2,479	-1,343	9.69E-07	4.92E-04	0.18	0.11	0.22	246	0.24	0.32	0.06	0.15	0.47
Tocochromanol Pathway	GRMZM2G035213	γ-tocopherol methyltransferase	αT3/γT3	S5_200369508	GBS	5	200,369,508	2,479	-1,343	1.16E-05	2.36E-02	0.17	0.11	0.22	246	0.13	0.20	-0.05	-0.15	0.41
Tocochromanol Pathway	GRMZM2G035213	γ-tocopherol methyltransferase	αT	S5_200369508	GBS	5	200,369,508	2,479	-1,343	6.85E-07	3.22E-04	0.18	0.11	0.22	251	0.25	0.33	0.28	0.40	0.85

Tocochromanol Pathway	GRMZM2G035213	γ-tocopherol methyltransferase	γT/(γT+αT)	S5_200369508	GBS	5	200,369,508	2,479	-1,343	6.57E-07	4.02E-04	0.18	0.11	0.22	251	0.29	0.36	-0.10	2.00	-0.05
Tocochromanol Pathway	GRMZM2G035213	γ-tocopherol methyltransferase	δT/αT	S5_200369508	GBS	5	200,369,508	2,479	-1,343	8.30E-06	3.60E-03	0.17	0.11	0.22	246	0.28	0.34	-0.45	Log	-0.99
Tocochromanol Pathway	GRMZM2G035213	γ-tocopherol methyltransferase	αT/γT	S5_200369534	GBS	5	200,369,534	2,505	-1,317	5.45E-06	2.37E-03	0.09	0.00	0.12	246	0.24	0.31	-0.07	0.15	-0.38
Tocochromanol Pathway	GRMZM2G035213	γ-tocopherol methyltransferase	αT	S5_200369534	GBS	5	200,369,534	2,505	-1,317	2.59E-06	9.88E-04	0.09	0.00	0.12	251	0.25	0.32	-0.34	0.40	-0.65
Tocochromanol Pathway	GRMZM2G035213	γ-tocopherol methyltransferase	γT/(γT+αT)	S5_200369534	GBS	5	200,369,534	2,505	-1,317	6.83E-05	1.99E-02	0.09	0.00	0.12	251	0.29	0.33	0.10	2.00	0.05
Tocochromanol Pathway	GRMZM2G035213	γ-tocopherol methyltransferase	δT/αT	S5_200369534	GBS	5	200,369,534	2,505	-1,317	6.11E-05	1.96E-02	0.08	0.00	0.12	246	0.28	0.33	0.49	Log	1.72
Tocochromanol Pathway	GRMZM2G035213	γ-tocopherol methyltransferase	αT/γT	S5_200369625	GBS	5	200,369,625	2,596	-1,226	2.86E-05	8.28E-03	0.30	0.48	0.49	246	0.24	0.30	-0.04	0.15	-0.24
Tocochromanol Pathway	GRMZM2G035213	γ-tocopherol methyltransferase	αT	S5_200369625	GBS	5	200,369,625	2,596	-1,226	5.00E-08	3.40E-05	0.30	0.48	0.49	251	0.25	0.35	-0.24	0.40	-0.5
Tocochromanol Pathway	GRMZM2G035213	γ-tocopherol methyltransferase	γT/(γT+αT)	S5_200369625	GBS	5	200,369,625	2,596	-1,226	8.13E-06	3.83E-03	0.29	0.48	0.49	251	0.29	0.35	0.07	2.00	0.03
Tocochromanol Pathway	GRMZM2G035213	γ-tocopherol methyltransferase	δT/αT	S5_200369625	GBS	5	200,369,625	2,596	-1,226	2.02E-06	1.23E-03	0.30	0.48	0.49	246	0.28	0.35	0.35	Log	1.14
Tocochromanol Pathway	GRMZM2G035213	γ-tocopherol methyltransferase	αT/γT	S5_200369644	GBS	5	200,369,644	2,615	-1,207	9.97E-06	3.20E-03	0.13	0.12	0.22	246	0.24	0.31	-0.05	0.15	-0.29
Tocochromanol Pathway	GRMZM2G035213	γ-tocopherol methyltransferase	αT	S5_200369644	GBS	5	200,369,644	2,615	-1,207	9.36E-06	2.60E-03	0.12	0.12	0.22	251	0.25	0.32	-0.26	0.40	-0.53
Tocochromanol Pathway	GRMZM2G035213	γ-tocopherol methyltransferase	αT/γT	S5_200369665	GBS	5	200,369,665	2,636	-1,186	9.97E-06	3.20E-03	0.13	0.12	0.22	246	0.24	0.31	0.05	0.15	0.38
Tocochromanol Pathway	GRMZM2G035213	γ-tocopherol methyltransferase	αT	S5_200369665	GBS	5	200,369,665	2,636	-1,186	9.36E-06	2.60E-03	0.12	0.12	0.22	251	0.25	0.32	0.26	0.40	0.78
Tocochromanol Pathway	GRMZM2G035213	γ-tocopherol methyltransferase	αT/γT	S5_200369667	GBS	5	200,369,667	2,638	-1,184	1.70E-07	1.21E-04	0.11	0.00	0.20	246	0.24	0.33	-0.07	0.15	-0.38
Tocochromanol Pathway	GRMZM2G035213	γ-tocopherol methyltransferase	αT	S5_200369667	GBS	5	200,369,667	2,638	-1,184	1.67E-07	9.31E-05	0.10	0.00	0.20	251	0.25	0.34	-0.33	0.40	-0.63
Tocochromanol Pathway	GRMZM2G035213	γ-tocopherol methyltransferase	γT/(γT+αT)	S5_200369667	GBS	5	200,369,667	2,638	-1,184	1.48E-05	6.49E-03	0.10	0.00	0.20	251	0.29	0.34	0.09	2.00	0.04
Tocochromanol Pathway	GRMZM2G035213	γ-tocopherol methyltransferase	δT/αT	S5_200369667	GBS	5	200,369,667	2,638	-1,184	7.09E-06	3.32E-03	0.10	0.00	0.20	246	0.28	0.34	0.48	Log	1.67
Tocochromanol Pathway	GRMZM2G035213	γ-tocopherol methyltransferase	αT	ss196468364	55K	5	200,370,065	3,036	-786	3.24E-05	8.27E-03	0.35	0.39	0.31	251	0.25	0.31	0.18	0.40	0.51
Tocochromanol Pathway	GRMZM2G035213	γ-tocopherol methyltransferase	αT/γT	PZB02424.2	4K	5	200,370,309	3,280	-542	2.60E-04	6.10E-02	0.17	0.09	0.19	246	0.24	0.29	-0.04	0.15	-0.24
Tocochromanol Pathway	GRMZM2G035213	γ-tocopherol methyltransferase	αT	PZB02424.2	4K	5	200,370,309	3,280	-542	4.09E-07	2.09E-04	0.17	0.09	0.19	251	0.25	0.33	-0.26	0.40	-0.53
Tocochromanol Pathway	GRMZM2G035213	γ-tocopherol methyltransferase	γT/(γT+αT)	PZB02424.2	4K	5	200,370,309	3,280	-542	7.64E-04	1.04E-01	0.17	0.09	0.19	251	0.29	0.32	0.06	2.00	0.03
Tocochromanol Pathway	GRMZM2G035213	γ-tocopherol methyltransferase	δT/αT	PZB02424.2	4K	5	200,370,309	3,280	-542	5.35E-03	3.17E-01	0.16	0.09	0.19	246	0.28	0.30	0.25	Log	0.77
Tocochromanol Pathway	GRMZM2G035213	γ-tocopherol methyltransferase	αT/γT	ss196468368	55K	5	200,371,057	4,028	206	7.37E-06	2.90E-03	0.14	0.13	0.15	246	0.24	0.31	-0.05	0.15	-0.29
Tocochromanol Pathway	GRMZM2G035213	γ-tocopherol methyltransferase	αT	ss196468368	55K	5	200,371,057	4,028	206	6.73E-06	2.17E-03	0.14	0.13	0.15	251	0.25	0.32	-0.27	0.40	-0.54
Tocochromanol Pathway	GRMZM2G035213	γ-tocopherol methyltransferase	γT/(γT+αT)	ss196468368	55K	5	200,371,057	4,028	206	2.74E-05	1.05E-02	0.14	0.13	0.15	251	0.29	0.34	0.09	2.00	0.04
Tocochromanol Pathway	GRMZM2G035213	γ-tocopherol methyltransferase	δT/αT	ss196468368	55K	5	200,371,057	4,028	206	5.98E-05	1.96E-02	0.13	0.13	0.15	246	0.28	0.33	0.43	Log	1.46
Tocochromanol Pathway	GRMZM2G035213	γ-tocopherol methyltransferase	γT/(γT+αT)	S5_200382117	GBS	5	200,382,117	15,088	11,266	1.43E-04	3.25E-02	0.06	0.07	0.07	251	0.29	0.33	-0.11	2.00	-0.06
Tocochromanol Pathway	GRMZM2G035213	γ-tocopherol methyltransferase	γT/(γT+αT)	S5_200382141	GBS	5	200,382,141	15,112	11,290	1.43E-04	3.25E-02	0.06	0.07	0.07	251	0.29	0.33	-0.11	2.00	-0.06
Tocochromanol Pathway	GRMZM2G035213	γ-tocopherol methyltransferase	αT/γT	S5_200382168	GBS	5	200,382,168	15,139	11,317	3.08E-08	3.13E-05	0.39	0.38	0.40	246	0.24	0.34	-0.05	0.15	-0.29
Tocochromanol Pathway	GRMZM2G035213	γ-tocopherol methyltransferase	αT	S5_200382168	GBS	5	200,382,168	15,139	11,317	3.01E-08	2.30E-05	0.39	0.38	0.40	251	0.25	0.35	-0.25	0.40	-0.51
Tocochromanol Pathway	GRMZM2G035213	γ-tocopherol methyltransferase	γT/(γT+αT)	S5_200382168	GBS	5	200,382,168	15,139	11,317	9.59E-09	8.38E-06	0.39	0.38	0.40	251	0.29	0.39	0.09	2.00	0.04
Tocochromanol Pathway	GRMZM2G035213	γ-tocopherol methyltransferase	δT/αT	S5_200382168	GBS	5	200,382,168	15,139	11,317	6.31E-08	6.39E-05	0.41	0.38	0.40	246	0.28	0.37	0.41	Log	1.38
Tocochromanol Pathway	GRMZM2G035213	γ-tocopherol methyltransferase	αT/γT	S5_200384686	GBS	5	200,384,686	17,657	13,835	1.72E-04	4.57E-02	0.13	0.31	0.21	246	0.24	0.29	0.04	0.15	0.3
Tocochromanol Pathway	GRMZM2G035213	γ-tocopherol methyltransferase	αT	S5_200384686	GBS	5	200,384,686	17,657	13,835	2.77E-05	7.37E-03	0.14	0.31	0.21	251	0.25	0.31	0.24	0.40	0.71
Tocochromanol Pathway	GRMZM2G035213	γ-tocopherol methyltransferase	γT/(γT+αT)	S5_200384686	GBS	5	200,384,686	17,657	13,835	1.07E-04	2.85E-02	0.14	0.31	0.21	251	0.29	0.33	-0.08	2.00	-0.04
Tocochromanol Pathway	GRMZM2G035213	γ-tocopherol methyltransferase	αT	S5_200435003	GBS	5	200,435,003	67,974	64,152	1.74E-04	3.33E-02	0.11	0.12	0.13	251	0.25	0.30	0.25	0.40	0.75

Tocochromanol Pathway	GRMZM2G035213	γ-tocopherol methyltransferase	αT	S5_200435045	GBS	5	200,435,045	68,016	64,194	1.40E-04	2.85E-02	0.12	0.12	0.13	251	0.25	0.30	-0.24	0.40	-0.5
Tocochromanol Pathway	GRMZM2G035213	γ-tocopherol methyltransferase	αT/γT	S5_200435108	GBS	5	200,435,108	68,079	64,257	3.28E-10	5.00E-07	0.25	0.44	0.21	246	0.24	0.38	-0.06	0.15	-0.34
Tocochromanol Pathway	GRMZM2G035213	γ-tocopherol methyltransferase	αT3	S5_200435108	GBS	5	200,435,108	68,079	64,257	2.65E-05	3.57E-02	0.26	0.44	0.21	248	0.20	0.26	-0.01	0.05	-0.18
Tocochromanol Pathway	GRMZM2G035213	γ-tocopherol methyltransferase	αT	S5_200435108	GBS	5	200,435,108	68,079	64,257	4.39E-10	6.71E-07	0.26	0.44	0.21	251	0.25	0.38	-0.30	0.40	-0.59
Tocochromanol Pathway	GRMZM2G035213	γ-tocopherol methyltransferase	γT/(γT+αT)	S5_200435108	GBS	5	200,435,108	68,079	64,257	1.13E-10	1.73E-07	0.27	0.44	0.21	251	0.29	0.42	0.11	2.00	0.05
Tocochromanol Pathway	GRMZM2G035213	γ-tocopherol methyltransferase	δT/αT	S5_200435108	GBS	5	200,435,108	68,079	64,257	6.37E-09	9.68E-06	0.27	0.44	0.21	246	0.28	0.39	0.47	Log	1.63
Tocochromanol Pathway	GRMZM2G035213	γ-tocopherol methyltransferase	αT/γT	S5_200435117	GBS	5	200,435,117	68,088	64,266	1.79E-07	1.21E-04	0.27	0.19	0.28	246	0.24	0.33	0.05	0.15	0.38
Tocochromanol Pathway	GRMZM2G035213	γ-tocopherol methyltransferase	αT3/γT3	S5_200435117	GBS	5	200,435,117	68,088	64,266	7.91E-06	2.36E-02	0.26	0.19	0.28	246	0.13	0.21	-0.04	-0.15	0.31
Tocochromanol Pathway	GRMZM2G035213	γ-tocopherol methyltransferase	αT3	S5_200435117	GBS	5	200,435,117	68,088	64,266	2.50E-06	1.53E-02	0.26	0.19	0.28	248	0.20	0.27	0.01	0.05	0.22
Tocochromanol Pathway	GRMZM2G035213	γ-tocopherol methyltransferase	αT	S5_200435117	GBS	5	200,435,117	68,088	64,266	1.21E-07	7.41E-05	0.26	0.19	0.28	251	0.25	0.34	0.25	0.40	0.75
Tocochromanol Pathway	GRMZM2G035213	γ-tocopherol methyltransferase	γT/(γT+αT)	S5_200435117	GBS	5	200,435,117	68,088	64,266	1.66E-07	1.13E-04	0.26	0.19	0.28	251	0.29	0.37	-0.09	2.00	-0.05
Tocochromanol Pathway	GRMZM2G035213	γ-tocopherol methyltransferase	δT/αT	S5_200435117	GBS	5	200,435,117	68,088	64,266	7.93E-07	5.35E-04	0.25	0.19	0.28	246	0.28	0.35	-0.41	Log	-0.91
Tocochromanol Pathway	GRMZM2G035213	γ-tocopherol methyltransferase	αT/γT	ss196468352	55K	5	200,435,300	68,271	64,449	2.68E-08	3.13E-05	0.39	0.39	0.37	246	0.24	0.35	-0.05	0.15	-0.29
Tocochromanol Pathway	GRMZM2G035213	γ-tocopherol methyltransferase	αT3/γT3	ss196468352	55K	5	200,435,300	68,271	64,449	5.91E-06	2.36E-02	0.40	0.39	0.37	246	0.13	0.21	0.04	-0.15	-0.23
Tocochromanol Pathway	GRMZM2G035213	γ-tocopherol methyltransferase	αT	ss196468352	55K	5	200,435,300	68,271	64,449	8.32E-10	1.02E-06	0.40	0.39	0.37	251	0.25	0.38	-0.27	0.40	-0.54
Tocochromanol Pathway	GRMZM2G035213	γ-tocopherol methyltransferase	γT/(γT+αT)	ss196468352	55K	5	200,435,300	68,271	64,449	7.67E-09	7.82E-06	0.40	0.39	0.37	251	0.29	0.39	0.09	2.00	0.04
Tocochromanol Pathway	GRMZM2G035213	γ-tocopherol methyltransferase	γT3/(γT3+αT3)	ss196468352	55K	5	200,435,300	68,271	64,449	3.05E-05	4.66E-02	0.40	0.39	0.37	251	0.16	0.22	0.06	1.35	0.04
Tocochromanol Pathway	GRMZM2G035213	γ-tocopherol methyltransferase	δT/αT	ss196468352	55K	5	200,435,300	68,271	64,449	2.87E-08	3.48E-05	0.41	0.39	0.37	246	0.28	0.38	0.42	Log	1.42
Tocochromanol Pathway	GRMZM2G035213	γ-tocopherol methyltransferase	αT/γT	S5_200437468	GBS	5	200,437,468	70,439	66,617	9.02E-08	7.84E-05	0.27	0.45	0.24	246	0.24	0.34	-0.05	0.15	-0.29
Tocochromanol Pathway	GRMZM2G035213	γ-tocopherol methyltransferase	αT	S5_200437468	GBS	5	200,437,468	70,439	66,617	2.29E-08	2.00E-05	0.28	0.45	0.24	251	0.25	0.35	-0.26	0.40	-0.53
Tocochromanol Pathway	GRMZM2G035213	γ-tocopherol methyltransferase	γT/(γT+αT)	S5_200437468	GBS	5	200,437,468	70,439	66,617	4.13E-08	3.16E-05	0.28	0.45	0.24	251	0.29	0.38	0.09	2.00	0.04
Tocochromanol Pathway	GRMZM2G035213	γ-tocopherol methyltransferase	δT/αT	S5_200437468	GBS	5	200,437,468	70,439	66,617	3.72E-07	3.23E-04	0.29	0.45	0.24	246	0.28	0.36	0.40	Log	1.34
Tocochromanol Pathway	GRMZM2G035213	γ-tocopherol methyltransferase	αT/γT	ss196517251	55K	5	200,437,606	70,577	66,755	4.45E-07	2.71E-04	0.45	0.23	0.40	246	0.24	0.33	-0.04	0.15	-0.24
Tocochromanol Pathway	GRMZM2G035213	γ-tocopherol methyltransferase	αT	ss196517251	55K	5	200,437,606	70,577	66,755	1.38E-06	5.62E-04	0.46	0.23	0.40	251	0.25	0.33	-0.21	0.40	-0.45
Tocochromanol Pathway	GRMZM2G035213	γ-tocopherol methyltransferase	γT/(γT+αT)	ss196517251	55K	5	200,437,606	70,577	66,755	2.99E-06	1.53E-03	0.46	0.23	0.40	251	0.29	0.35	0.07	2.00	0.03
Tocochromanol Pathway	GRMZM2G035213	γ-tocopherol methyltransferase	δT/αT	ss196517251	55K	5	200,437,606	70,577	66,755	5.53E-06	3.05E-03	0.47	0.23	0.40	246	0.28	0.34	0.35	Log	1.14
Tocochromanol Pathway	GRMZM2G035213	γ-tocopherol methyltransferase	αT/γT	S5_200438801	GBS	5	200,438,801	71,772	67,950	7.67E-06	2.90E-03	0.10	0.06	0.13	246	0.24	0.31	0.06	0.15	0.47
Tocochromanol Pathway	GRMZM2G035213	γ-tocopherol methyltransferase	αT	S5_200438801	GBS	5	200,438,801	71,772	67,950	4.30E-06	1.46E-03	0.10	0.06	0.13	251	0.25	0.32	0.31	0.40	0.96
Tocochromanol Pathway	GRMZM2G035213	γ-tocopherol methyltransferase	γT/(γT+αT)	S5_200438801	GBS	5	200,438,801	71,772	67,950	2.09E-04	4.58E-02	0.10	0.06	0.13	251	0.29	0.33	-0.09	2.00	-0.05
Tocochromanol Pathway	GRMZM2G035213	γ-tocopherol methyltransferase	αT	ss196512031	55K	5	200,491,310	124,281	120,459	1.09E-04	2.38E-02	0.40	0.35	0.36	251	0.25	0.30	0.17	0.40	0.48
Tocochromanol Pathway	GRMZM2G035213	γ-tocopherol methyltransferase	γT/(γT+αT)	ss196512031	55K	5	200,491,310	124,281	120,459	1.07E-04	2.85E-02	0.40	0.35	0.36	251	0.29	0.33	-0.06	2.00	-0.03
Tocochromanol Pathway	GRMZM2G035213	γ-tocopherol methyltransferase	δT/αT	ss196512031	55K	5	200,491,310	124,281	120,459	1.11E-05	4.51E-03	0.40	0.35	0.36	246	0.28	0.34	-0.33	Log	-0.76
Tocochromanol Pathway	GRMZM2G035213	γ-tocopherol methyltransferase	αT	ss196468372	55K	5	200,598,221	231,192	227,370	2.53E-04	3.97E-02	0.25	0.18	0.27	251	0.25	0.30	-0.19	0.40	-0.41
Tocochromanol Pathway	GRMZM2G173358	homogentisic acid geranylgeranyl transferase	Total Tocotrienols	S9_92346116	GBS	9	92,346,116	-137,433	-141,152	6.37E-06	3.76E-02	0.41	0.43	0.44	250	0.20	0.27	0.03	0.15	0.22
Tocochromanol Pathway	GRMZM2G173358	homogentisic acid geranylgeranyl transferase	γT3	S9_92346116	GBS	9	92,346,116	-137,433	-141,152	3.10E-06	4.76E-03	0.41	0.43	0.44	250	0.20	0.27	0.03	0.10	0.34
Tocochromanol Pathway	GRMZM2G173358	homogentisic acid geranylgeranyl transferase	Total Tocopherols/Total Tocotrienols	S9_92548696	GBS	9	92,548,696	65,147	61,428	1.28E-05	3.91E-02	0.39	0.38	0.40	247	0.19	0.26	-0.01	0.05	-0.20
Tocochromanol	GRMZM2G173358	homogentisic acid	αT3	S9_92553908	GBS	9	92,553,908	70,359	66,640	2.92E-05	3.57E-02	0.43	0.05	0.38	248	0.20	0.26	0.01	0.05	0.22

Pathway		geranylgeranyl transferase																		
Tocochromanol Pathway	GRMZM2G173358	homogentisic acid geranylgeranyl transferase	Total Tocopherols/Total Tocotrienols	S9_92554465	GBS	9	92,554,465	70,916	67,197	2.45E-06	1.49E-02	0.26	0.00	0.32	247	0.19	0.27	-0.01	0.05	-0.24
Tocochromanol Pathway	GRMZM2G173358	homogentisic acid geranylgeranyl transferase	Total Tocotrienols	S9_92718671	GBS	9	92,718,671	235,122	231,403	5.43E-05	4.69E-02	0.08	0.00	0.13	250	0.20	0.26	-0.05	0.15	-0.29
Tocochromanol Pathway	GRMZM2G173358	homogentisic acid geranylgeranyl transferase	γT3/(γT3+αT3)	S9_92718671	GBS	9	92,718,671	235,122	231,403	1.45E-05	2.96E-02	0.08	0.00	0.13	251	0.16	0.23	-0.10	1.35	-0.08
Tocochromanol Pathway	GRMZM2G173358	homogentisic acid geranylgeranyl transferase	γT3	S9_92718671	GBS	9	92,718,671	235,122	231,403	4.48E-07	9.16E-04	0.08	0.00	0.13	250	0.20	0.28	-0.05	0.10	-0.4
Tocochromanol Pathway	GRMZM2G173358	homogentisic acid geranylgeranyl transferase	Total Tocotrienols	S9_92718674	GBS	9	92,718,674	235,125	231,406	5.43E-05	4.69E-02	0.08	0.00	0.13	250	0.20	0.26	-0.05	0.15	-0.29
Tocochromanol Pathway	GRMZM2G173358	homogentisic acid geranylgeranyl transferase	γT3/(γT3+αT3)	S9_92718674	GBS	9	92,718,674	235,125	231,406	1.45E-05	2.96E-02	0.08	0.00	0.13	251	0.16	0.23	-0.10	1.35	-0.08
Tocochromanol Pathway	GRMZM2G173358	homogentisic acid geranylgeranyl transferase	γT3	S9_92718674	GBS	9	92,718,674	235,125	231,406	4.48E-07	9.16E-04	0.08	0.00	0.13	250	0.20	0.28	-0.05	0.10	-0.4
Tocochromanol Pathway	GRMZM2G173358	homogentisic acid geranylgeranyl transferase	Total Tocotrienols	S9_92718709	GBS	9	92,718,709	235,160	231,441	5.43E-05	4.69E-02	0.08	0.00	0.13	250	0.20	0.26	-0.05	0.15	-0.29
Tocochromanol Pathway	GRMZM2G173358	homogentisic acid geranylgeranyl transferase	γT3/(γT3+αT3)	S9_92718709	GBS	9	92,718,709	235,160	231,441	1.45E-05	2.96E-02	0.08	0.00	0.13	251	0.16	0.23	-0.10	1.35	-0.08
Tocochromanol Pathway	GRMZM2G173358	homogentisic acid geranylgeranyl transferase	γT3	S9_92718709	GBS	9	92,718,709	235,160	231,441	4.48E-07	9.16E-04	0.08	0.00	0.13	250	0.20	0.28	-0.05	0.10	-0.4

Table S7b Statistically significant results from the candidate gene association study of 20 tocochromanol grain traits with the three SNPs within ZmVTE4 identified in the multi-locus mixed-model (MLMM) analysis included as covariates. SNPs that were significantly associated with the indicated trait at 5% FDR are shown.

***a priori* candidate gene pathway**	**RefGen_v2 Gene ID**	**RefGen_v2 Annotated Gene Function**	**Trait**	**SNP ID**	**SNP Source**	**Chr**	**Position**	**Distance from Gene ORF Start**	**Distance from Gene ORF Finish**	***P*-value**	**FDR Adjusted *P*-value**	**Minor Allele Frequency (MAF)**	**MAF Tropical (18% of 252 Lines)**	**MAF Temperate (82% of 252 Lines)**	**Sample Size**	**R^2_{LR} from Model without SNP**	**R^2_{LR} from Model with SNP**	**Effect Size**	**Lambda from Box-Cox Procedure**	**Back-Transformed Effect Estimates**
Aromatic Head Group	GRMZM2G437912	prephenate dehydratase	Total Tocotrienols	S2_59013838	GBS	2	59,013,838	-23,405	-25,340	2.16E-05	4.40E-02	0.06	0.40	0.09	250	0.20	0.27	0.06	0.15	0.47
Aromatic Head Group	GRMZM2G437912	prephenate dehydratase	Total Tocotrienols	S2_59013840	GBS	2	59,013,840	-23,403	-25,338	2.16E-05	4.40E-02	0.06	0.40	0.09	250	0.20	0.27	-0.06	0.15	-0.34
Aromatic Head Group	GRMZM2G070218	shikimate kinase	δT/αT	S5_207660287	GBS	5	207,660,287	-44,669	-47,594	7.82E-06	4.75E-02	0.13	0.15	0.16	246	0.56	0.60	-0.36	0.00	-0.82
Prenyl Group Synthesis	GRMZM2G027059	4-hydroxy-3-methylbut-2-enyldiphosphate reductase	δT3	ss196519818	55K	1	272,871,696	-65,140	-68,806	1.49E-04	4.80E-02	0.32	0.09	0.38	247	0.12	0.18	-0.06	-0.20	0.36
Prenyl Group Synthesis	GRMZM2G027059	4-hydroxy-3-methylbut-2-enyldiphosphate reductase	δT3	ss196519822	55K	1	272,874,639	-62,197	-65,863	1.39E-04	4.72E-02	0.33	0.09	0.38	247	0.12	0.18	-0.06	-0.20	0.36
Prenyl Group Synthesis	GRMZM2G172032	2-C-methyl-D-erythritol 4-phosphate cytidyltransferase	δT3/(γT3+αT3)	S8_164626861	GBS	8	164,626,861	-122,078	-125,510	4.09E-05	1.19E-02	0.14	0.50	0.10	248	0.10	0.17	-0.04	-0.10	0.5
Tocochromanol Pathway	GRMZM2G009785	tocopherol cyclase	δT3/(γT3+αT3)	S5_133331094	GBS	5	133,331,094	-170,834	-187,401	3.88E-05	1.19E-02	0.41	0.47	0.41	248	0.10	0.17	0.03	-0.10	-0.26
Tocochromanol Pathway	GRMZM2G009785	tocopherol cyclase	δT3/αT3	S5_133331094	GBS	5	133,331,094	-170,834	-187,401	6.18E-05	3.42E-02	0.41	0.47	0.41	246	0.14	0.20	0.05	-0.15	-0.28
Tocochromanol Pathway	GRMZM2G009785	tocopherol cyclase	δT3	S5_133331094	GBS	5	133,331,094	-170,834	-187,401	5.02E-06	3.83E-03	0.41	0.47	0.41	247	0.12	0.20	0.06	-0.20	-0.25
Tocochromanol Pathway	GRMZM2G009785	tocopherol cyclase	δT3/(γT3+αT3)	S5_133331096	GBS	5	133,331,096	-170,832	-187,399	1.60E-05	6.10E-03	0.40	0.44	0.41	248	0.10	0.17	-0.03	-0.10	0.36
Tocochromanol Pathway	GRMZM2G009785	tocopherol cyclase	δT3/αT3	S5_133331096	GBS	5	133,331,096	-170,832	-187,399	2.10E-05	2.76E-02	0.40	0.44	0.41	246	0.14	0.20	-0.05	-0.15	0.41
Tocochromanol Pathway	GRMZM2G009785	tocopherol cyclase	δT3	S5_133331096	GBS	5	133,331,096	-170,832	-187,399	1.15E-06	1.75E-03	0.40	0.44	0.41	247	0.12	0.21	-0.06	-0.20	0.36
Tocochromanol Pathway	GRMZM2G009785	tocopherol cyclase	δT3/(γT3+αT3)	S5_133331106	GBS	5	133,331,106	-170,822	-187,389	1.60E-05	6.10E-03	0.40	0.47	0.41	248	0.10	0.17	0.03	-0.10	-0.26
Tocochromanol Pathway	GRMZM2G009785	tocopherol cyclase	δT3/αT3	S5_133331106	GBS	5	133,331,106	-170,822	-187,389	2.10E-05	2.76E-02	0.40	0.47	0.41	246	0.14	0.20	0.05	-0.15	-0.28
Tocochromanol Pathway	GRMZM2G009785	tocopherol cyclase	δT3	S5_133331106	GBS	5	133,331,106	-170,822	-187,389	1.15E-06	1.75E-03	0.40	0.47	0.41	247	0.12	0.21	0.06	-0.20	-0.25
Tocochromanol Pathway	GRMZM2G009785	tocopherol cyclase	δT3/(γT3+αT3)	ss196465630	55K	5	133,332,323	-169,605	-186,172	6.91E-05	1.76E-02	0.32	0.11	0.36	248	0.10	0.16	-0.03	-0.10	0.36
Tocochromanol Pathway	GRMZM2G009785	tocopherol cyclase	δT3/(γT3+αT3)	ss196465628	55K	5	133,333,095	-168,833	-185,400	4.48E-05	1.24E-02	0.42	0.47	0.42	248	0.10	0.17	-0.03	-0.10	0.36
Tocochromanol Pathway	GRMZM2G009785	tocopherol cyclase	δT3/αT3	ss196465628	55K	5	133,333,095	-168,833	-185,400	9.53E-05	4.83E-02	0.42	0.47	0.42	246	0.14	0.19	-0.05	-0.15	0.41
Tocochromanol Pathway	GRMZM2G009785	tocopherol cyclase	δT3	ss196465628	55K	5	133,333,095	-168,833	-185,400	4.21E-06	3.67E-03	0.42	0.47	0.42	247	0.12	0.20	-0.06	-0.20	0.36
Tocochromanol Pathway	GRMZM2G009785	tocopherol cyclase	δT3/(γT3+αT3)	S5_133333397	GBS	5	133,333,397	-168,531	-185,098	6.99E-06	4.59E-03	0.43	0.47	0.43	248	0.10	0.18	0.03	-0.10	-0.26
Tocochromanol Pathway	GRMZM2G009785	tocopherol cyclase	δT3/(γT3+αT3)	ss196465626	55K	5	133,333,397	-168,531	-185,098	2.53E-05	8.58E-03	0.42	0.47	0.42	248	0.10	0.17	0.03	-0.10	-0.26
Tocochromanol Pathway	GRMZM2G009785	tocopherol cyclase	δT3/αT3	S5_133333397	GBS	5	133,333,397	-168,531	-185,098	2.27E-05	2.76E-02	0.43	0.47	0.43	246	0.14	0.20	0.05	-0.15	-0.28
Tocochromanol Pathway	GRMZM2G009785	tocopherol cyclase	δT3/αT3	ss196465626	55K	5	133,333,397	-168,531	-185,098	5.49E-05	3.42E-02	0.42	0.47	0.42	246	0.14	0.20	0.05	-0.15	-0.28
Tocochromanol Pathway	GRMZM2G009785	tocopherol cyclase	δT3	S5_133333397	GBS	5	133,333,397	-168,531	-185,098	3.88E-07	1.19E-03	0.43	0.47	0.43	247	0.12	0.22	0.06	-0.20	-0.25
Tocochromanol Pathway	GRMZM2G009785	tocopherol cyclase	δT3	ss196465626	55K	5	133,333,397	-168,531	-185,098	1.85E-06	1.88E-03	0.42	0.47	0.42	247	0.12	0.21	0.06	-0.20	-0.25
Tocochromanol Pathway	GRMZM2G009785	tocopherol cyclase	δT3/(γT3+αT3)	S5_133333561	GBS	5	133,333,561	-168,367	-184,934	8.69E-06	4.59E-03	0.42	0.48	0.43	248	0.10	0.18	0.03	-0.10	-0.26
Tocochromanol Pathway	GRMZM2G009785	tocopherol cyclase	δT3/αT3	S5_133333561	GBS	5	133,333,561	-168,367	-184,934	2.06E-05	2.76E-02	0.42	0.48	0.43	246	0.14	0.20	0.05	-0.15	-0.28
Tocochromanol Pathway	GRMZM2G009785	tocopherol cyclase	δT3	S5_133333561	GBS	5	133,333,561	-168,367	-184,934	3.67E-07	1.19E-03	0.42	0.48	0.43	247	0.12	0.22	0.06	-0.20	-0.25
Tocochromanol Pathway	GRMZM2G009785	tocopherol cyclase	δT3/(γT3+αT3)	S5_133335078	GBS	5	133,335,078	-166,850	-183,417	1.50E-05	6.10E-03	0.30	0.33	0.29	248	0.10	0.17	-0.03	-0.10	0.36
Tocochromanol Pathway	GRMZM2G009785	tocopherol cyclase	δT3/αT3	S5_133335078	GBS	5	133,335,078	-166,850	-183,417	5.98E-05	3.42E-02	0.30	0.33	0.29	246	0.14	0.20	-0.06	-0.15	0.51
Tocochromanol Pathway	GRMZM2G009785	tocopherol cyclase	δT3	S5_133335078	GBS	5	133,335,078	-166,850	-183,417	1.49E-06	1.82E-03	0.30	0.33	0.29	247	0.12	0.21	-0.06	-0.20	0.36
Tocochromanol Pathway	GRMZM2G009785	tocopherol cyclase	δT3/(γT3+αT3)	S5_133338747	GBS	5	133,338,747	-163,181	-179,748	2.95E-05	9.47E-03	0.37	0.17	0.41	248	0.10	0.17	-0.03	-0.10	0.36
Tocochromanol Pathway	GRMZM2G009785	tocopherol cyclase	δT3/γT3	S5_133338747	GBS	5	133,338,747	-163,181	-179,748	4.80E-05	2.93E-02	0.37	0.17	0.41	249	0.13	0.19	0.01	0.05	0.22
Tocochromanol Pathway	GRMZM2G009785	tocopherol cyclase	δT/γT	PZA00524.2	4K	5	133,338,936	-162,992	-179,559	1.64E-05	4.42E-02	0.41	0.23	0.39	249	0.14	0.21	-0.02	0.30	-0.07
Tocochromanol Pathway	GRMZM2G009785	tocopherol cyclase	δT/γT	S5_133338936	GBS	5	133,338,936	-162,992	-179,559	1.80E-05	4.42E-02	0.47	0.17	0.40	249	0.14	0.21	0.02	0.30	0.07
Tocochromanol Pathway	GRMZM2G009785	tocopherol cyclase	δT3/(γT3+αT3)	S5_133499169	GBS	5	133,499,169	-2,759	-19,326	1.98E-06	3.02E-03	0.39	0.15	0.44	248	0.10	0.19	-0.03	-0.10	0.36
Tocochromanol Pathway	GRMZM2G009785	tocopherol cyclase	δT3/αT3	S5_133499169	GBS	5	133,499,169	-2,759	-19,326	6.13E-05	3.42E-02	0.39	0.15	0.44	246	0.14	0.20	-0.05	-0.15	0.41
Tocochromanol Pathway	GRMZM2G009785	tocopherol cyclase	δT3/γT3	S5_133499169	GBS	5	133,499,169	-2,759	-19,326	8.05E-05	4.10E-02	0.39	0.15	0.44	249	0.13	0.19	0.01	0.05	0.22
Tocochromanol Pathway	GRMZM2G009785	tocopherol cyclase	δT3	S5_133499169	GBS	5	133,499,169	-2,759	-19,326	1.31E-04	4.71E-02	0.39	0.15	0.44	247	0.12	0.18	-0.05	-0.20	0.29
Tocochromanol Pathway	GRMZM2G009785	tocopherol cyclase	δT3/(γT3+αT3)	S5_133499269	GBS	5	133,499,269	-2,659	-19,226	4.23E-06	4.59E-03	0.39	0.18	0.46	248	0.10	0.18	0.03	-0.10	-0.26
Tocochromanol Pathway	GRMZM2G009785	tocopherol cyclase	δT3/(γT3+αT3)	S5_133501858	GBS	5	133,501,858	-70	-16,637	1.58E-07	9.62E-04	0.39	0.19	0.46	248	0.10	0.21	0.04	-0.10	-0.32
Tocochromanol Pathway	GRMZM2G009785	tocopherol cyclase	δT3/αT3	S5_133501858	GBS	5	133,501,858	-70	-16,637	1.55E-05	2.76E-02	0.39	0.19	0.46	246	0.14	0.21	0.06	-0.15	-0.32
Tocochromanol Pathway	GRMZM2G009785	tocopherol cyclase	δT3/γT3	S5_133501858	GBS	5	133,501,858	-70	-16,637	1.34E-06	8.18E-03	0.39	0.19	0.46	249	0.13	0.22	-0.01	0.05	-0.18
Tocochromanol Pathway	GRMZM2G009785	tocopherol cyclase	δT3	S5_133501858	GBS	5	133,501,858	-70	-16,637	7.85E-06	5.32E-03	0.39	0.19	0.46	247	0.12	0.20	0.06	-0.20	-0.25
Tocochromanol Pathway	GRMZM2G009785	tocopherol cyclase	δT3/(γT3+αT3)	S5_133501992	GBS	5	133,501,992	64	-16,503	1.37E-06	2.79E-03	0.39	0.14	0.46	248	0.10	0.19	0.03	-0.10	-0.26
Tocochromanol Pathway	GRMZM2G009785	tocopherol cyclase	δT3/αT3	S5_133501992	GBS	5	133,501,992	64	-16,503	5.72E-05	3.42E-02	0.39	0.14	0.46	246	0.14	0.20	0.05	-0.15	-0.28
Tocochromanol Pathway	GRMZM2G009785	tocopherol cyclase	δT3/γT3	S5_133501992	GBS	5	133,501,992	64	-16,503	4.15E-06	8.46E-03	0.39	0.14	0.46	249	0.13	0.21	-0.01	0.05	-0.18
Tocochromanol Pathway	GRMZM2G009785	tocopherol cyclase	δT3	S5_133501992	GBS	5	133,501,992	64	-16,503	1.92E-05	1.06E-02	0.39	0.14	0.46	247	0.12	0.19	0.06	-0.20	-0.25
Tocochromanol Pathway	GRMZM2G009785	tocopherol cyclase	δT3/(γT3+αT3)	PZB00969.1	4K	5	133,502,506	578	-15,989	1.17E-06	2.79E-03	0.40	0.14	0.46	248	0.10	0.19	0.03	-0.10	-0.26
Tocochromanol Pathway	GRMZM2G009785	tocopherol cyclase	δT3/(γT3+αT3)	ss196416168	55K	5	133,502,506	578	-15,989	1.74E-05	6.24E-03	0.40	0.13	0.46	248	0.10	0.17	0.03	-0.10	-0.26
Tocochromanol Pathway	GRMZM2G009785	tocopherol cyclase	δT3/αT3	PZB00969.1	4K	5	133,502,506	578	-15,989	6.09E-05	3.42E-02	0.40	0.14	0.46	246	0.14	0.20	0.05	-0.15	-0.28
Tocochromanol Pathway	GRMZM2G009785	tocopherol cyclase	δT3/γT3	PZB00969.1	4K	5	133,502,506	578	-15,989	2.74E-06	8.36E-03	0.40	0.14	0.46	249	0.13	0.21	-0.01	0.05	-0.18
Tocochromanol Pathway	GRMZM2G009785	tocopherol cyclase	δT3/γT3	ss196416168	55K	5	133,502,506	578	-15,989	3.19E-05	2.17E-02	0.40	0.13	0.46	249	0.13	0.20	-0.01	0.05	-0.18

Tocochromanol Pathway	GRMZM2G009785	tocopherol cyclase	δT3	PZB00969.1	4K	5	133,502,506	578	-15,989	1.65E-05	1.01E-02	0.40	0.14	0.46	247	0.12	0.19	0.06	-0.20	-0.25
Tocochromanol Pathway	GRMZM2G009785	tocopherol cyclase	δT3	ss196416168	55K	5	133,502,506	578	-15,989	1.63E-04	4.97E-02	0.40	0.13	0.46	247	0.12	0.18	0.05	-0.20	-0.22
Tocochromanol Pathway	GRMZM2G009785	tocopherol cyclase	δT3/(γT3+αT3)	ss196465634	55K	5	133,510,613	8,685	-7,882	9.77E-06	4.59E-03	0.41	0.14	0.46	248	0.10	0.18	-0.03	-0.10	0.36
Tocochromanol Pathway	GRMZM2G009785	tocopherol cyclase	δT3/γT3	ss196465634	55K	5	133,510,613	8,685	-7,882	1.39E-05	1.84E-02	0.41	0.14	0.46	249	0.13	0.20	0.01	0.05	0.22
Tocochromanol Pathway	GRMZM2G009785	tocopherol cyclase	δT3	ss196465634	55K	5	133,510,613	8,685	-7,882	1.03E-04	4.71E-02	0.41	0.14	0.46	247	0.12	0.18	-0.05	-0.20	0.29
Tocochromanol Pathway	GRMZM2G009785	tocopherol cyclase	δT3/(γT3+αT3)	PZB02491.1	4K	5	133,517,065	15,137	-1,430	8.41E-06	4.59E-03	0.40	0.14	0.46	248	0.10	0.18	-0.03	-0.10	0.36
Tocochromanol Pathway	GRMZM2G009785	tocopherol cyclase	δT3/γT3	PZB02491.1	4K	5	133,517,065	15,137	-1,430	7.72E-05	4.10E-02	0.40	0.14	0.46	249	0.13	0.19	0.01	0.05	0.22
Tocochromanol Pathway	GRMZM2G009785	tocopherol cyclase	δT3	PZB02491.1	4K	5	133,517,065	15,137	-1,430	6.16E-05	3.13E-02	0.40	0.14	0.46	247	0.12	0.18	-0.05	-0.20	0.29
Tocochromanol Pathway	GRMZM2G009785	tocopherol cyclase	δT3/(γT3+αT3)	S5_133618308	GBS	5	133,618,308	116,380	99,813	9.54E-06	4.59E-03	0.40	0.10	0.48	248	0.10	0.18	0.03	-0.10	-0.26
Tocochromanol Pathway	GRMZM2G009785	tocopherol cyclase	δT3/γT3	S5_133618308	GBS	5	133,618,308	116,380	99,813	2.36E-05	1.84E-02	0.40	0.10	0.48	249	0.13	0.20	-0.01	0.05	-0.18
Tocochromanol Pathway	GRMZM2G009785	tocopherol cyclase	δT3	S5_133618308	GBS	5	133,618,308	116,380	99,813	1.31E-04	4.71E-02	0.40	0.10	0.48	247	0.12	0.18	0.05	-0.20	-0.22
Tocochromanol Pathway	GRMZM2G009785	tocopherol cyclase	δT3/(γT3+αT3)	S5_133618309	GBS	5	133,618,309	116,381	99,814	9.54E-06	4.59E-03	0.40	0.10	0.48	248	0.10	0.18	0.03	-0.10	-0.26
Tocochromanol Pathway	GRMZM2G009785	tocopherol cyclase	δT3/γT3	S5_133618309	GBS	5	133,618,309	116,381	99,814	2.36E-05	1.84E-02	0.40	0.10	0.48	249	0.13	0.20	-0.01	0.05	-0.18
Tocochromanol Pathway	GRMZM2G009785	tocopherol cyclase	δT3	S5_133618309	GBS	5	133,618,309	116,381	99,814	1.31E-04	4.71E-02	0.40	0.10	0.48	247	0.12	0.18	0.05	-0.20	-0.22
Tocochromanol Pathway	GRMZM2G009785	tocopherol cyclase	δT3/(γT3+αT3)	S5_133618344	GBS	5	133,618,344	116,416	99,849	9.54E-06	4.59E-03	0.40	0.10	0.48	248	0.10	0.18	0.03	-0.10	-0.26
Tocochromanol Pathway	GRMZM2G009785	tocopherol cyclase	δT3/γT3	S5_133618344	GBS	5	133,618,344	116,416	99,849	2.36E-05	1.84E-02	0.40	0.10	0.48	249	0.13	0.20	-0.01	0.05	-0.18
Tocochromanol Pathway	GRMZM2G009785	tocopherol cyclase	δT3	S5_133618344	GBS	5	133,618,344	116,416	99,849	1.31E-04	4.71E-02	0.40	0.10	0.48	247	0.12	0.18	0.05	-0.20	-0.22
Tocochromanol Pathway	GRMZM2G009785	tocopherol cyclase	δT/γT	S5_133618788	GBS	5	133,618,788	116,860	100,293	2.89E-05	4.42E-02	0.49	0.11	0.42	249	0.14	0.20	0.02	0.30	0.07
Tocochromanol Pathway	GRMZM2G009785	tocopherol cyclase	δT3/(γT3+αT3)	S5_133618788	GBS	5	133,618,788	116,860	100,293	1.85E-04	4.33E-02	0.49	0.11	0.42	248	0.10	0.16	-0.03	-0.10	0.36
Tocochromanol Pathway	GRMZM2G009785	tocopherol cyclase	δT/γT	S5_133618810	GBS	5	133,618,810	116,882	100,315	2.89E-05	4.42E-02	0.49	0.11	0.42	249	0.14	0.20	0.02	0.30	0.07
Tocochromanol Pathway	GRMZM2G009785	tocopherol cyclase	δT3/(γT3+αT3)	S5_133618810	GBS	5	133,618,810	116,882	100,315	1.85E-04	4.33E-02	0.49	0.11	0.42	248	0.10	0.16	-0.03	-0.10	0.36
Tocochromanol Pathway	GRMZM2G009785	tocopherol cyclase	δT3/(γT3+αT3)	S5_133691297	GBS	5	133,691,297	189,369	172,802	5.54E-06	4.59E-03	0.37	0.10	0.46	248	0.10	0.18	0.03	-0.10	-0.26
Tocochromanol Pathway	GRMZM2G009785	tocopherol cyclase	δT3/γT3	S5_133691297	GBS	5	133,691,297	189,369	172,802	2.41E-05	1.84E-02	0.37	0.10	0.46	249	0.13	0.20	-0.01	0.05	-0.18
Tocochromanol Pathway	GRMZM2G009785	tocopherol cyclase	δT3/(γT3+αT3)	ss196465642	55K	5	133,728,050	226,122	209,555	5.07E-05	1.35E-02	0.40	0.11	0.46	248	0.10	0.17	-0.03	-0.10	0.36
Tocochromanol Pathway	GRMZM2G173358	homogentisic acid geranylgeranyl transferase	Total Tocotrienols	S9_92346116	GBS	9	92,346,116	-137,433	-141,152	7.27E-06	4.40E-02	0.41	0.43	0.44	250	0.20	0.27	0.03	0.15	0.22
Tocochromanol Pathway	GRMZM2G173358	homogentisic acid geranylgeranyl transferase	γT3	S9_92346116	GBS	9	92,346,116	-137,433	-141,152	2.33E-06	3.56E-03	0.41	0.43	0.44	250	0.20	0.28	0.03	0.10	0.34
Tocochromanol Pathway	GRMZM2G173358	homogentisic acid geranylgeranyl transferase	Total Tocopherols/Total Tocotrienols	S9_92548696	GBS	9	92,548,696	65,147	61,428	1.02E-05	3.13E-02	0.39	0.38	0.40	247	0.19	0.26	-0.01	0.05	-0.21
Tocochromanol Pathway	GRMZM2G173358	homogentisic acid geranylgeranyl transferase	Total Tocopherols/Total Tocotrienols	S9_92554465	GBS	9	92,554,465	70,916	67,197	2.75E-06	1.67E-02	0.26	0.00	0.32	247	0.19	0.27	-0.01	0.05	-0.24
Tocochromanol Pathway	GRMZM2G173358	homogentisic acid geranylgeranyl transferase	γT3/(γT3+αT3)	S9_92718671	GBS	9	92,718,671	235,122	231,403	5.56E-06	1.13E-02	0.08	0.00	0.13	251	0.20	0.27	-0.10	1.35	-0.08
Tocochromanol Pathway	GRMZM2G173358	homogentisic acid geranylgeranyl transferase	γT3	S9_92718671	GBS	9	92,718,671	235,122	231,403	4.49E-07	9.16E-04	0.08	0.00	0.13	250	0.20	0.29	-0.05	0.10	-0.4
Tocochromanol Pathway	GRMZM2G173358	homogentisic acid geranylgeranyl transferase	γT3/(γT3+αT3)	S9_92718674	GBS	9	92,718,674	235,125	231,406	5.56E-06	1.13E-02	0.08	0.00	0.13	251	0.20	0.27	-0.10	1.35	-0.08
Tocochromanol Pathway	GRMZM2G173358	homogentisic acid geranylgeranyl transferase	γT3	S9_92718674	GBS	9	92,718,674	235,125	231,406	4.49E-07	9.16E-04	0.08	0.00	0.13	250	0.20	0.29	-0.05	0.10	-0.4
Tocochromanol Pathway	GRMZM2G173358	homogentisic acid geranylgeranyl transferase	γT3/(γT3+αT3)	S9_92718709	GBS	9	92,718,709	235,160	231,441	5.56E-06	1.13E-02	0.08	0.00	0.13	251	0.20	0.27	-0.10	1.35	-0.08
Tocochromanol Pathway	GRMZM2G173358	homogentisic acid geranylgeranyl transferase	γT3	S9_92718709	GBS	9	92,718,709	235,160	231,441	4.49E-07	9.16E-04	0.08	0.00	0.13	250	0.20	0.29	-0.05	0.10	-0.4

Table S7c Statistically significant results from the candidate gene association study of 20 tocochromanol grain traits with the two SNPs in the region including ZmVTE1 identified in the multi-locus mixed-model (MLMM) analysis included as covariates. SNPs that were significantly associated with the indicated trait at 5% FDR are shown.

a priori candidate gene pathway	RefGen_v2 Gene ID	RefGen_v2 Annotated Gene Function	Trait	SNP ID	SNP Source	Chr	Position	Distance from Gene ORF Start	Distance from Gene ORF Finish	*P*-value	FDR Adjusted *P*-value	Minor Allele Frequency (MAF)	MAF Tropical (18% of 252 Lines)	MAF Temperate (82% of 252 Lines)	Sample Size	R^2_{LR} from Model without SNP	R^2_{LR} from Model with SNP	Effect Size	Lambda from Box-Cox Procedure	Back-Transformed Effect Estimates
Aromatic Head Group	GRMZM2G437912	prephenate dehydratase	γT3	S2_59013838	GBS	2	59,013,838	-23,405	-25,340	3.44E-05	3.01E-02	0.06	0.40	0.09	250	0.23	0.28	0.04	0.10	0.47
Aromatic Head Group	GRMZM2G437912	prephenate dehydratase	Total Tocotrienols	S2_59013838	GBS	2	59,013,838	-23,405	-25,340	1.45E-05	2.96E-02	0.06	0.40	0.09	250	0.23	0.29	0.06	0.15	0.43
Aromatic Head Group	GRMZM2G437912	prephenate dehydratase	Total Tocotrienols	S2_59013840	GBS	2	59,013,840	-23,403	-25,338	1.45E-05	2.96E-02	0.06	0.40	0.09	250	0.23	0.29	-0.06	0.15	-0.32
Aromatic Head Group	GRMZM2G437912	prephenate dehydratase	γT3	S2_59013840	GBS	2	59,013,840	-23,403	-25,338	3.44E-05	3.01E-02	0.06	0.40	0.09	250	0.23	0.28	-0.04	0.10	-0.33
Aromatic Head Group	GRMZM2G573867	3-dehydroquinate synthase	δT/αT	S2_196295236	GBS	2	196,295,236	-64,391	-68,456	1.71E-04	4.51E-02	0.06	0.04	0.07	246	0.28	0.33	-0.48	0.00	-0.83
Aromatic Head Group	GRMZM2G573867	3-dehydroquinate synthase	αT	S2_196514167	GBS	2	196,514,167	154,540	150,475	9.47E-06	2.90E-03	0.06	0.02	0.07	251	0.25	0.32	-0.36	0.40	-0.67
Aromatic Head Group	GRMZM2G138624	isochorismatase hydrolase	αT3	ss196456226	55K	4	134,020,078	96,788	94,084	1.27E-05	2.70E-02	0.45	0.45	0.46	248	0.21	0.28	0.01	0.05	0.14
Aromatic Head Group	GRMZM2G124365	chorismate mutase	αT	S8_173041758	GBS	8	173,041,758	-61,455	-65,990	2.70E-04	4.59E-02	0.12	0.18	0.22	251	0.25	0.30	-0.23	0.40	-0.49
Aromatic Head Group	GRMZM2G124365	chorismate mutase	δT/αT	S8_173070782	GBS	8	173,070,782	-32,431	-36,966	1.50E-04	4.16E-02	0.18	0.04	0.33	246	0.28	0.33	-0.36	0.00	-1.05
Aromatic Head Group	GRMZM2G124365	chorismate mutase	Total Tocotrienols	S8_173243067	GBS	8	173,243,067	139,854	135,319	5.41E-05	4.95E-02	0.09	0.20	0.09	250	0.23	0.28	0.05	0.15	-0.3
Aromatic Head Group	GRMZM2G124365	chorismate mutase	αT3	S8_173243067	GBS	8	173,243,067	139,854	135,319	4.36E-05	4.88E-02	0.09	0.20	0.09	248	0.21	0.27	0.01	0.05	0.2
Prenyl Group Synthesis	GRMZM2G027059	4-hydroxy-3-methylbut-2-enyldiphosphate reductase	αT	S1_273098217	GBS	1	273,098,217	161,381	157,715	3.50E-05	8.56E-03	0.22	0.24	0.24	251	0.25	0.31	-0.21	0.40	-0.44
Prenyl Group Synthesis	AC209374.4_FG002	2-C-methyl-D-erythritol 2,4-cyclodiphosphate synthase	αT/γT	S5_196278264	GBS	5	196,278,264	-1,031	-2,773	1.29E-04	3.57E-02	0.25	0.17	0.30	246	0.25	0.29	0.04	0.15	0.29
Prenyl Group Synthesis	AC209374.4_FG002	2-C-methyl-D-erythritol 2,4-cyclodiphosphate synthase	αT	S5_196278264	GBS	5	196,278,264	-1,031	-2,773	1.36E-04	2.87E-02	0.25	0.17	0.30	251	0.25	0.30	0.19	0.40	0.55
Prenyl Group Synthesis	AC209374.4_FG002	2-C-methyl-D-erythritol 2,4-cyclodiphosphate synthase	γT/(γT+αT)	S5_196278264	GBS	5	196,278,264	-1,031	-2,773	6.18E-05	2.10E-02	0.25	0.17	0.30	251	0.29	0.34	-0.07	2.00	-0.04
Prenyl Group Synthesis	AC209374.4_FG002	2-C-methyl-D-erythritol 2,4-cyclodiphosphate synthase	δT/αT	S5_196278264	GBS	5	196,278,264	-1,031	-2,773	2.46E-05	8.78E-03	0.25	0.17	0.30	246	0.28	0.34	-0.36	0.00	-0.84
Prenyl Group Synthesis	GRMZM2G133082	isopentenyl pyrophosphate isomerase	γT/(γT+αT)	S6_147333828	GBS	6	147,333,828	202,712	197,149	2.19E-04	4.79E-02	0.07	0.22	0.05	251	0.29	0.33	-0.11	2.00	-0.06
Prenyl Group Synthesis	GRMZM2G133082	isopentenyl pyrophosphate isomerase	γT/(γT+αT)	S6_147333833	GBS	6	147,333,833	202,717	197,154	2.19E-04	4.79E-02	0.07	0.22	0.05	251	0.29	0.33	-0.11	2.00	-0.06
Prenyl Group Synthesis	GRMZM2G493395	1-deoxy-D-xylulose 5-phosphate synthase	αT/γT	ss196475603	55K	7	13,959,219	-118,633	-121,856	1.58E-05	4.81E-03	0.30	0.37	0.30	246	0.25	0.31	0.04	0.15	0.3
Prenyl Group Synthesis	GRMZM2G493395	1-deoxy-D-xylulose 5-phosphate synthase	γT/(γT+αT)	ss196475603	55K	7	13,959,219	-118,633	-121,856	4.39E-05	1.58E-02	0.30	0.37	0.30	251	0.29	0.34	-0.07	2.00	-0.03
Tocochromanol Pathway	GRMZM2G035213	γ-tocopherol methyltransferase	αT	S5_200117524	GBS	5	200,117,524	-249,505	-253,327	3.88E-05	9.14E-03	0.21	0.02	0.27	251	0.25	0.31	0.22	0.40	0.66
Tocochromanol Pathway	GRMZM2G035213	γ-tocopherol methyltransferase	αT	S5_200117526	GBS	5	200,117,526	-249,503	-253,325	2.14E-04	3.97E-02	0.22	0.07	0.27	251	0.25	0.30	0.20	0.40	0.73
Tocochromanol Pathway	GRMZM2G035213	γ-tocopherol methyltransferase	αT	S5_200119597	GBS	5	200,119,597	-247,432	-251,254	1.95E-04	3.73E-02	0.33	0.18	0.36	251	0.25	0.30	0.17	0.40	0.44
Tocochromanol Pathway	GRMZM2G035213	γ-tocopherol methyltransferase	αT	S5_200119623	GBS	5	200,119,623	-247,406	-251,228	2.80E-04	4.64E-02	0.35	0.27	0.37	251	0.25	0.30	0.16	0.40	-0.47
Tocochromanol Pathway	GRMZM2G035213	γ-tocopherol methyltransferase	γT/(γT+αT)	S5_200130689	GBS	5	200,130,689	-236,340	-240,162	7.59E-05	2.21E-02	0.07	0.33	0.08	251	0.29	0.34	0.12	2.00	0.06
Tocochromanol Pathway	GRMZM2G035213	γ-tocopherol methyltransferase	δT/αT	S5_200130689	GBS	5	200,130,689	-236,340	-240,162	1.82E-04	4.61E-02	0.07	0.33	0.08	246	0.28	0.33	0.54	0.00	-0.83
Tocochromanol Pathway	GRMZM2G035213	γ-tocopherol methyltransferase	γT/(γT+αT)	S5_200292465	GBS	5	200,292,465	-74,564	-78,386	7.49E-05	2.21E-02	0.34	0.34	0.28	251	0.29	0.34	-0.06	2.00	-0.03
Tocochromanol Pathway	GRMZM2G035213	γ-tocopherol methyltransferase	αT/γT	S5_200293693	GBS	5	200,293,693	-73,336	-77,158	1.02E-05	3.64E-03	0.41	0.34	0.37	246	0.25	0.31	0.04	0.15	0.29
Tocochromanol Pathway	GRMZM2G035213	γ-tocopherol methyltransferase	αT	S5_200293693	GBS	5	200,293,693	-73,336	-77,158	3.70E-06	1.33E-03	0.41	0.34	0.37	251	0.25	0.32	0.20	0.40	0.58
Tocochromanol Pathway	GRMZM2G035213	γ-tocopherol methyltransferase	γT/(γT+αT)	S5_200293693	GBS	5	200,293,693	-73,336	-77,158	1.59E-06	8.86E-04	0.41	0.34	0.37	251	0.29	0.36	-0.07	2.00	-0.04
Tocochromanol Pathway	GRMZM2G035213	γ-tocopherol methyltransferase	δT/αT	S5_200293693	GBS	5	200,293,693	-73,336	-77,158	2.21E-05	8.39E-03	0.41	0.34	0.37	246	0.28	0.34	-0.31	0.00	-0.73
Tocochromanol Pathway	GRMZM2G035213	γ-tocopherol methyltransferase	αT	ss196468356	55K	5	200,300,836	-66,193	-70,015	9.23E-05	2.09E-02	0.43	0.20	0.49	251	0.25	0.30	-0.17	0.40	-0.37
Tocochromanol Pathway	GRMZM2G035213	γ-tocopherol methyltransferase	δT/αT	ss196468356	55K	5	200,300,836	-66,193	-70,015	4.99E-06	2.53E-03	0.43	0.20	0.49	246	0.28	0.35	0.34	0.00	1.09
Tocochromanol Pathway	GRMZM2G035213	γ-tocopherol methyltransferase	αT/γT	S5_200318615	GBS	5	200,318,615	-48,414	-52,236	3.21E-06	1.51E-03	0.22	0.27	0.44	246	0.25	0.32	0.05	0.15	0.37
Tocochromanol Pathway	GRMZM2G035213	γ-tocopherol methyltransferase	αT	S5_200318615	GBS	5	200,318,615	-48,414	-52,236	1.42E-06	5.81E-04	0.22	0.27	0.44	251	0.25	0.33	0.25	0.40	0.74
Tocochromanol Pathway	GRMZM2G035213	γ-tocopherol methyltransferase	γT/(γT+αT)	S5_200318615	GBS	5	200,318,615	-48,414	-52,236	2.36E-05	9.65E-03	0.22	0.27	0.44	251	0.29	0.34	-0.08	2.00	-0.04
Tocochromanol Pathway	GRMZM2G035213	γ-tocopherol methyltransferase	δT/αT	S5_200318615	GBS	5	200,318,615	-48,414	-52,236	9.52E-05	2.89E-02	0.22	0.27	0.44	246	0.28	0.33	-0.34	0.00	-0.8
Tocochromanol Pathway	GRMZM2G035213	γ-tocopherol methyltransferase	αT/γT	PZB02283.1	4K	5	200,367,532	503	-3,319	1.04E-12	2.11E-09	0.20	0.14	0.22	246	0.25	0.42	0.08	0.15	0.64
Tocochromanol Pathway	GRMZM2G035213	γ-tocopherol methyltransferase	αT/γT	ss196416269	55K	5	200,367,532	503	-3,319	1.04E-12	2.11E-09	0.20	0.13	0.22	246	0.25	0.42	0.08	0.15	0.64
Tocochromanol Pathway	GRMZM2G035213	γ-tocopherol methyltransferase	αT	PZB02283.1	4K	5	200,367,532	503	-3,319	1.01E-13	2.07E-10	0.20	0.14	0.22	251	0.25	0.44	0.40	0.40	1.31
Tocochromanol Pathway	GRMZM2G035213	γ-tocopherol methyltransferase	αT	ss196416269	55K	5	200,367,532	503	-3,319	1.01E-13	2.07E-10	0.20	0.13	0.22	251	0.25	0.44	0.40	0.40	1.31
Tocochromanol Pathway	GRMZM2G035213	γ-tocopherol methyltransferase	γT/(γT+αT)	PZB02283.1	4K	5	200,367,532	503	-3,319	4.98E-12	1.02E-08	0.20	0.14	0.22	251	0.29	0.44	-0.13	2.00	-0.07
Tocochromanol Pathway	GRMZM2G035213	γ-tocopherol methyltransferase	γT/(γT+αT)	ss196416269	55K	5	200,367,532	503	-3,319	4.98E-12	1.02E-08	0.20	0.13	0.22	251	0.29	0.44	-0.13	2.00	-0.07
Tocochromanol Pathway	GRMZM2G035213	γ-tocopherol methyltransferase	δT/αT	PZB02283.1	4K	5	200,367,532	503	-3,319	2.46E-11	4.98E-08	0.20	0.14	0.22	246	0.28	0.43	-0.63	0.00	-1.27
Tocochromanol Pathway	GRMZM2G035213	γ-tocopherol methyltransferase	δT/αT	ss196416269	55K	5	200,367,532	503	-3,319	2.46E-11	4.98E-08	0.20	0.13	0.22	246	0.28	0.43	-0.63	0.00	-1.27
Tocochromanol Pathway	GRMZM2G035213	γ-tocopherol methyltransferase	αT/γT	ss196468362	55K	5	200,369,124	2,095	-1,727	1.04E-12	2.11E-09	0.20	0.13	0.22	246	0.25	0.42	0.08	0.15	0.64
Tocochromanol Pathway	GRMZM2G035213	γ-tocopherol methyltransferase	αT	ss196468362	55K	5	200,369,124	2,095	-1,727	1.01E-13	2.07E-10	0.20	0.13	0.22	251	0.25	0.44	0.40	0.40	1.31
Tocochromanol Pathway	GRMZM2G035213	γ-tocopherol methyltransferase	γT/(γT+αT)	ss196468362	55K	5	200,369,124	2,095	-1,727	4.98E-12	1.02E-08	0.20	0.13	0.22	251	0.29	0.44	-0.13	2.00	-0.07
Tocochromanol Pathway	GRMZM2G035213	γ-tocopherol methyltransferase	δT/αT	ss196468362	55K	5	200,369,124	2,095	-1,727	2.46E-11	4.98E-08	0.20	0.13	0.22	246	0.28	0.43	-0.63	0.00	-1.27
Tocochromanol Pathway	GRMZM2G035213	γ-tocopherol methyltransferase	αT/γT	S5_200369481	GBS	5	200,369,481	2,452	-1,370	5.14E-07	2.85E-04	0.11	0.36	0.09	246	0.25	0.33	0.07	0.15	0.57

Tocochromanol Pathway	GRMZM2G035213	γ-tocopherol methyltransferase	αT	S5_200369481	GBS	5	200,369,481	2,452	-1,370	1.81E-08	1.58E-05	0.11	0.36	0.09	251	0.25	0.36	0.38	0.40	1.23
Tocochromanol Pathway	GRMZM2G035213	γ-tocopherol methyltransferase	γT/(γT+αT)	S5_200369481	GBS	5	200,369,481	2,452	-1,370	5.10E-09	6.24E-06	0.11	0.36	0.09	251	0.29	0.40	-0.14	2.00	-0.07
Tocochromanol Pathway	GRMZM2G035213	γ-tocopherol methyltransferase	δT/(γT+αT)	S5_200369481	GBS	5	200,369,481	2,452	-1,370	7.53E-06	4.61E-02	0.11	0.36	0.09	251	0.21	0.27	-0.03	0.35	-0.07
Tocochromanol Pathway	GRMZM2G035213	γ-tocopherol methyltransferase	δT/αT	S5_200369481	GBS	5	200,369,481	2,452	-1,370	5.29E-07	4.02E-04	0.11	0.36	0.09	246	0.28	0.36	-0.57	0.00	-1.18
Tocochromanol Pathway	GRMZM2G035213	γ-tocopherol methyltransferase	αT/γT	S5_200369508	GBS	5	200,369,508	2,479	-1,343	7.52E-07	3.82E-04	0.18	0.11	0.22	246	0.25	0.33	0.06	0.15	0.44
Tocochromanol Pathway	GRMZM2G035213	γ-tocopherol methyltransferase	αT3/γT3	S5_200369508	GBS	5	200,369,508	2,479	-1,343	1.30E-05	2.64E-02	0.18	0.11	0.22	246	0.15	0.21	-0.05	-0.15	0.39
Tocochromanol Pathway	GRMZM2G035213	γ-tocopherol methyltransferase	αT	S5_200369508	GBS	5	200,369,508	2,479	-1,343	6.77E-07	3.19E-04	0.18	0.11	0.22	251	0.25	0.33	0.28	0.40	0.85
Tocochromanol Pathway	GRMZM2G035213	γ-tocopherol methyltransferase	γT/(γT+αT)	S5_200369508	GBS	5	200,369,508	2,479	-1,343	5.11E-07	3.13E-04	0.18	0.11	0.22	251	0.29	0.37	-0.10	2.00	-0.05
Tocochromanol Pathway	GRMZM2G035213	γ-tocopherol methyltransferase	γT3/(γT3+αT3)	S5_200369508	GBS	5	200,369,508	2,479	-1,343	5.08E-05	4.44E-02	0.18	0.11	0.22	251	0.18	0.24	-0.07	1.35	-0.05
Tocochromanol Pathway	GRMZM2G035213	γ-tocopherol methyltransferase	δT/αT	S5_200369508	GBS	5	200,369,508	2,479	-1,343	8.74E-06	3.79E-03	0.18	0.11	0.22	246	0.28	0.35	-0.44	0.00	-0.98
Tocochromanol Pathway	GRMZM2G035213	γ-tocopherol methyltransferase	αT/γT	S5_200369534	GBS	5	200,369,534	2,505	-1,317	5.38E-06	2.34E-03	0.08	0.00	0.12	246	0.25	0.31	-0.07	0.15	-0.36
Tocochromanol Pathway	GRMZM2G035213	γ-tocopherol methyltransferase	αT	S5_200369534	GBS	5	200,369,534	2,505	-1,317	2.74E-06	1.05E-03	0.08	0.00	0.12	251	0.25	0.32	-0.34	0.40	-0.64
Tocochromanol Pathway	GRMZM2G035213	γ-tocopherol methyltransferase	γT/(γT+αT)	S5_200369534	GBS	5	200,369,534	2,505	-1,317	6.52E-05	2.10E-02	0.08	0.00	0.12	251	0.29	0.34	0.10	2.00	0.05
Tocochromanol Pathway	GRMZM2G035213	γ-tocopherol methyltransferase	δT/αT	S5_200369534	GBS	5	200,369,534	2,505	-1,317	6.74E-05	2.16E-02	0.08	0.00	0.12	246	0.28	0.33	0.49	0.00	1.74
Tocochromanol Pathway	GRMZM2G035213	γ-tocopherol methyltransferase	αT/γT	S5_200369625	GBS	5	200,369,625	2,596	-1,226	2.92E-05	8.47E-03	0.30	0.48	0.49	246	0.25	0.30	-0.04	0.15	-0.23
Tocochromanol Pathway	GRMZM2G035213	γ-tocopherol methyltransferase	αT	S5_200369625	GBS	5	200,369,625	2,596	-1,226	6.30E-08	4.28E-05	0.30	0.48	0.49	251	0.25	0.35	-0.24	0.40	-0.49
Tocochromanol Pathway	GRMZM2G035213	γ-tocopherol methyltransferase	γT/(γT+αT)	S5_200369625	GBS	5	200,369,625	2,596	-1,226	7.75E-06	3.65E-03	0.30	0.48	0.49	251	0.29	0.35	0.07	2.00	0.03
Tocochromanol Pathway	GRMZM2G035213	γ-tocopherol methyltransferase	δT/αT	S5_200369625	GBS	5	200,369,625	2,596	-1,226	2.40E-06	1.46E-03	0.30	0.48	0.49	246	0.28	0.35	0.35	0.00	1.14
Tocochromanol Pathway	GRMZM2G035213	γ-tocopherol methyltransferase	αT/γT	S5_200369644	GBS	5	200,369,644	2,615	-1,207	1.40E-05	4.50E-03	0.13	0.12	0.22	246	0.25	0.31	-0.05	0.15	-0.29
Tocochromanol Pathway	GRMZM2G035213	γ-tocopherol methyltransferase	αT	S5_200369644	GBS	5	200,369,644	2,615	-1,207	1.20E-05	3.33E-03	0.13	0.12	0.22	251	0.25	0.31	-0.25	0.40	-0.52
Tocochromanol Pathway	GRMZM2G035213	γ-tocopherol methyltransferase	αT/γT	S5_200369665	GBS	5	200,369,665	2,636	-1,186	1.40E-05	4.50E-03	0.12	0.12	0.22	246	0.25	0.31	0.05	0.15	0.39
Tocochromanol Pathway	GRMZM2G035213	γ-tocopherol methyltransferase	αT	S5_200369665	GBS	5	200,369,665	2,636	-1,186	1.20E-05	3.33E-03	0.12	0.12	0.22	251	0.25	0.31	0.25	0.40	0.76
Tocochromanol Pathway	GRMZM2G035213	γ-tocopherol methyltransferase	αT/γT	S5_200369667	GBS	5	200,369,667	2,638	-1,184	1.90E-07	1.44E-04	0.10	0.00	0.20	246	0.25	0.34	-0.07	0.15	-0.36
Tocochromanol Pathway	GRMZM2G035213	γ-tocopherol methyltransferase	αT	S5_200369667	GBS	5	200,369,667	2,638	-1,184	2.13E-07	1.19E-04	0.10	0.00	0.20	251	0.25	0.34	-0.33	0.40	-0.63
Tocochromanol Pathway	GRMZM2G035213	γ-tocopherol methyltransferase	γT/(γT+αT)	S5_200369667	GBS	5	200,369,667	2,638	-1,184	1.48E-05	6.46E-03	0.10	0.00	0.20	251	0.29	0.35	0.09	2.00	0.05
Tocochromanol Pathway	GRMZM2G035213	γ-tocopherol methyltransferase	δT/αT	S5_200369667	GBS	5	200,369,667	2,638	-1,184	7.99E-06	3.74E-03	0.10	0.00	0.20	246	0.28	0.35	0.48	0.00	1.67
Tocochromanol Pathway	GRMZM2G035213	γ-tocopherol methyltransferase	αT	ss196468364	55K	5	200,370,065	3,036	-786	3.26E-05	8.38E-03	0.35	0.39	0.31	251	0.25	0.31	0.18	0.40	0.51
Tocochromanol Pathway	GRMZM2G035213	γ-tocopherol methyltransferase	αT	PZB02424.2	4K	5	200,370,309	3,280	-542	4.68E-07	2.39E-04	0.17	0.09	0.19	251	0.25	0.34	-0.26	0.40	-0.54
Tocochromanol Pathway	GRMZM2G035213	γ-tocopherol methyltransferase	αT/γT	ss196468368	55K	5	200,371,057	4,028	206	7.53E-06	2.87E-03	0.13	0.13	0.15	246	0.25	0.31	-0.05	0.15	-0.31
Tocochromanol Pathway	GRMZM2G035213	γ-tocopherol methyltransferase	αT	ss196468368	55K	5	200,371,057	4,028	206	7.87E-06	2.53E-03	0.13	0.13	0.15	251	0.25	0.32	-0.27	0.40	-0.55
Tocochromanol Pathway	GRMZM2G035213	γ-tocopherol methyltransferase	γT/(γT+αT)	ss196468368	55K	5	200,371,057	4,028	206	2.65E-05	1.01E-02	0.13	0.13	0.15	251	0.29	0.34	0.09	2.00	0.04
Tocochromanol Pathway	GRMZM2G035213	γ-tocopherol methyltransferase	δT/αT	ss196468368	55K	5	200,371,057	4,028	206	6.12E-05	2.07E-02	0.13	0.13	0.15	246	0.28	0.33	0.43	0.00	1.45
Tocochromanol Pathway	GRMZM2G035213	γ-tocopherol methyltransferase	γT/(γT+αT)	S5_200382117	GBS	5	200,382,117	15,088	11,266	1.39E-04	3.56E-02	0.06	0.07	0.07	251	0.29	0.33	-0.11	2.00	-0.06
Tocochromanol Pathway	GRMZM2G035213	γ-tocopherol methyltransferase	γT/(γT+αT)	S5_200382141	GBS	5	200,382,141	15,112	11,290	1.39E-04	3.56E-02	0.06	0.07	0.07	251	0.29	0.33	-0.11	2.00	-0.06
Tocochromanol Pathway	GRMZM2G035213	γ-tocopherol methyltransferase	αT/γT	S5_200382168	GBS	5	200,382,168	15,139	11,317	2.81E-08	2.86E-05	0.41	0.38	0.40	246	0.25	0.35	-0.05	0.15	-0.29
Tocochromanol Pathway	GRMZM2G035213	γ-tocopherol methyltransferase	αT	S5_200382168	GBS	5	200,382,168	15,139	11,317	3.30E-08	2.52E-05	0.41	0.38	0.40	251	0.25	0.35	-0.25	0.40	-0.51
Tocochromanol Pathway	GRMZM2G035213	γ-tocopherol methyltransferase	γT/(γT+αT)	S5_200382168	GBS	5	200,382,168	15,139	11,317	7.60E-09	6.65E-06	0.41	0.38	0.40	251	0.29	0.39	0.09	2.00	0.05
Tocochromanol Pathway	GRMZM2G035213	γ-tocopherol methyltransferase	δT/αT	S5_200382168	GBS	5	200,382,168	15,139	11,317	5.45E-08	5.52E-05	0.41	0.38	0.40	246	0.28	0.38	0.42	0.00	1.4
Tocochromanol Pathway	GRMZM2G035213	γ-tocopherol methyltransferase	αT	S5_200384686	GBS	5	200,384,686	17,657	13,835	3.29E-05	8.38E-03	0.14	0.31	0.21	251	0.25	0.31	0.24	0.40	0.7
Tocochromanol Pathway	GRMZM2G035213	γ-tocopherol methyltransferase	γT/(γT+αT)	S5_200384686	GBS	5	200,384,686	17,657	13,835	1.48E-04	3.61E-02	0.14	0.31	0.21	251	0.29	0.33	-0.07	2.00	-0.04
Tocochromanol Pathway	GRMZM2G035213	γ-tocopherol methyltransferase	αT	S5_200435003	GBS	5	200,435,003	67,974	64,152	1.92E-04	3.73E-02	0.11	0.12	0.13	251	0.25	0.30	0.25	0.40	0.47
Tocochromanol Pathway	GRMZM2G035213	γ-tocopherol methyltransferase	αT	S5_200435045	GBS	5	200,435,045	68,016	64,194	1.50E-04	3.06E-02	0.12	0.12	0.13	251	0.25	0.30	-0.24	0.40	0.56
Tocochromanol Pathway	GRMZM2G035213	γ-tocopherol methyltransferase	αT/γT	S5_200435108	GBS	5	200,435,108	68,079	64,257	3.12E-10	4.75E-07	0.27	0.44	0.21	246	0.25	0.38	-0.06	0.15	-0.35
Tocochromanol Pathway	GRMZM2G035213	γ-tocopherol methyltransferase	αT3	S5_200435108	GBS	5	200,435,108	68,079	64,257	4.80E-05	4.88E-02	0.27	0.44	0.21	248	0.21	0.27	-0.01	0.05	0.13
Tocochromanol Pathway	GRMZM2G035213	γ-tocopherol methyltransferase	αT	S5_200435108	GBS	5	200,435,108	68,079	64,257	3.30E-10	5.04E-07	0.27	0.44	0.21	251	0.25	0.38	-0.31	0.40	-0.6
Tocochromanol Pathway	GRMZM2G035213	γ-tocopherol methyltransferase	γT/(γT+αT)	S5_200435108	GBS	5	200,435,108	68,079	64,257	1.15E-10	1.76E-07	0.27	0.44	0.21	251	0.29	0.42	0.11	2.00	0.05
Tocochromanol Pathway	GRMZM2G035213	γ-tocopherol methyltransferase	δT/αT	S5_200435108	GBS	5	200,435,108	68,079	64,257	1.95E-09	2.97E-06	0.27	0.44	0.21	246	0.28	0.40	0.50	0.00	1.75
Tocochromanol Pathway	GRMZM2G035213	γ-tocopherol methyltransferase	αT/γT	S5_200435117	GBS	5	200,435,117	68,088	64,266	2.42E-07	1.64E-04	0.26	0.19	0.28	246	0.25	0.33	0.05	0.15	0.38
Tocochromanol Pathway	GRMZM2G035213	γ-tocopherol methyltransferase	αT3/γT3	S5_200435117	GBS	5	200,435,117	68,088	64,266	9.48E-06	2.64E-02	0.26	0.19	0.28	246	0.15	0.22	-0.04	-0.15	0.32
Tocochromanol Pathway	GRMZM2G035213	γ-tocopherol methyltransferase	αT3	S5_200435117	GBS	5	200,435,117	68,088	64,266	1.60E-06	9.78E-03	0.26	0.19	0.28	248	0.21	0.29	0.01	0.05	0.16
Tocochromanol Pathway	GRMZM2G035213	γ-tocopherol methyltransferase	αT	S5_200435117	GBS	5	200,435,117	68,088	64,266	1.52E-07	9.30E-05	0.26	0.19	0.28	251	0.25	0.34	0.25	0.40	0.75
Tocochromanol Pathway	GRMZM2G035213	γ-tocopherol methyltransferase	γT/(γT+αT)	S5_200435117	GBS	5	200,435,117	68,088	64,266	2.25E-07	1.53E-04	0.26	0.19	0.28	251	0.29	0.37	-0.09	2.00	-0.05
Tocochromanol Pathway	GRMZM2G035213	γ-tocopherol methyltransferase	γT3/(γT3+αT3)	S5_200435117	GBS	5	200,435,117	68,088	64,266	4.56E-05	4.44E-02	0.26	0.19	0.28	251	0.18	0.24	-0.06	1.35	-0.04
Tocochromanol Pathway	GRMZM2G035213	γ-tocopherol methyltransferase	δT/αT	S5_200435117	GBS	5	200,435,117	68,088	64,266	6.96E-07	4.70E-04	0.26	0.19	0.28	246	0.28	0.36	-0.41	0.00	-0.92
Tocochromanol Pathway	GRMZM2G035213	γ-tocopherol methyltransferase	αT/γT	ss196468352	55K	5	200,435,300	68,271	64,449	2.56E-08	2.86E-05	0.41	0.39	0.37	246	0.25	0.35	-0.05	0.15	-0.29
Tocochromanol Pathway	GRMZM2G035213	γ-tocopherol methyltransferase	αT3/γT3	ss196468352	55K	5	200,435,300	68,271	64,449	6.70E-06	2.64E-02	0.41	0.39	0.37	246	0.15	0.22	0.04	-0.15	-0.22
Tocochromanol Pathway	GRMZM2G035213	γ-tocopherol methyltransferase	αT	ss196468352	55K	5	200,435,300	68,271	64,449	9.58E-10	1.17E-06	0.41	0.39	0.37	251	0.25	0.38	-0.27	0.40	-0.55
Tocochromanol Pathway	GRMZM2G035213	γ-tocopherol methyltransferase	γT/(γT+αT)	ss196468352	55K	5	200,435,300	68,271	64,449	6.65E-09	6.65E-06	0.41	0.39	0.37	251	0.29	0.39	0.09	2.00	0.05
Tocochromanol Pathway	GRMZM2G035213	γ-tocopherol methyltransferase	γT3/(γT3+αT3)	ss196468352	55K	5	200,435,300	68,271	64,449	2.81E-05	3.69E-02	0.41	0.39	0.37	251	0.18	0.24	0.06	1.35	0.04

Tocochromanol Pathway	GRMZM2G035213	γ-tocopherol methyltransferase	δT/αT	ss196468352	55K	5	200,435,300	68,271	64,449	2.92E-08	3.55E-05	0.41	0.39	0.37	246	0.28	0.38	0.42	0.00	1.42
Tocochromanol Pathway	GRMZM2G035213	γ-tocopherol methyltransferase	αT/γT	S5_200437468	GBS	5	200,437,468	70,439	66,617	7.35E-08	6.39E-05	0.29	0.45	0.24	246	0.25	0.34	-0.05	0.15	-0.3
Tocochromanol Pathway	GRMZM2G035213	γ-tocopherol methyltransferase	αT	S5_200437468	GBS	5	200,437,468	70,439	66,617	1.73E-08	1.58E-05	0.29	0.45	0.24	251	0.25	0.36	-0.27	0.40	-0.54
Tocochromanol Pathway	GRMZM2G035213	γ-tocopherol methyltransferase	γT/(γT+αT)	S5_200437468	GBS	5	200,437,468	70,439	66,617	3.36E-08	2.57E-05	0.29	0.45	0.24	251	0.29	0.38	0.09	2.00	0.05
Tocochromanol Pathway	GRMZM2G035213	γ-tocopherol methyltransferase	δT/αT	S5_200437468	GBS	5	200,437,468	70,439	66,617	1.49E-07	1.29E-04	0.29	0.45	0.24	246	0.28	0.37	0.42	0.00	1.42
Tocochromanol Pathway	GRMZM2G035213	γ-tocopherol methyltransferase	αT/γT	ss196517251	55K	5	200,437,606	70,577	66,755	4.82E-07	2.85E-04	0.47	0.23	0.40	246	0.25	0.33	-0.05	0.15	-0.26
Tocochromanol Pathway	GRMZM2G035213	γ-tocopherol methyltransferase	αT	ss196517251	55K	5	200,437,606	70,577	66,755	1.33E-06	5.80E-04	0.47	0.23	0.40	251	0.25	0.33	-0.21	0.40	-0.45
Tocochromanol Pathway	GRMZM2G035213	γ-tocopherol methyltransferase	γT/(γT+αT)	ss196517251	55K	5	200,437,606	70,577	66,755	3.40E-06	1.73E-03	0.47	0.23	0.40	251	0.29	0.36	0.07	2.00	0.04
Tocochromanol Pathway	GRMZM2G035213	γ-tocopherol methyltransferase	δT/αT	ss196517251	55K	5	200,437,606	70,577	66,755	3.35E-06	1.85E-03	0.47	0.23	0.40	246	0.28	0.35	0.35	0.00	1.16
Tocochromanol Pathway	GRMZM2G035213	γ-tocopherol methyltransferase	αT/γT	S5_200438801	GBS	5	200,438,801	71,772	67,950	6.92E-06	2.81E-03	0.10	0.06	0.13	246	0.25	0.31	0.06	0.15	0.48
Tocochromanol Pathway	GRMZM2G035213	γ-tocopherol methyltransferase	αT	S5_200438801	GBS	5	200,438,801	71,772	67,950	4.48E-06	1.52E-03	0.10	0.06	0.13	251	0.25	0.32	0.31	0.40	0.95
Tocochromanol Pathway	GRMZM2G035213	γ-tocopherol methyltransferase	γT/(γT+αT)	S5_200438801	GBS	5	200,438,801	71,772	67,950	1.81E-04	4.27E-02	0.10	0.06	0.13	251	0.29	0.33	-0.09	2.00	-0.04
Tocochromanol Pathway	GRMZM2G035213	γ-tocopherol methyltransferase	αT	ss196512031	55K	5	200,491,310	124,281	120,459	1.11E-04	2.43E-02	0.40	0.35	0.36	251	0.25	0.30	0.17	0.40	0.48
Tocochromanol Pathway	GRMZM2G035213	γ-tocopherol methyltransferase	γT/(γT+αT)	ss196512031	55K	5	200,491,310	124,281	120,459	1.08E-04	3.00E-02	0.40	0.35	0.36	251	0.29	0.34	-0.06	2.00	-0.03
Tocochromanol Pathway	GRMZM2G035213	γ-tocopherol methyltransferase	δT/αT	ss196512031	55K	5	200,491,310	124,281	120,459	1.13E-05	4.57E-03	0.40	0.35	0.36	246	0.28	0.34	-0.33	0.00	-0.77
Tocochromanol Pathway	GRMZM2G035213	γ-tocopherol methyltransferase	αT	ss196468372	55K	5	200,598,221	231,192	227,370	2.59E-04	4.59E-02	0.25	0.18	0.27	251	0.25	0.30	-0.19	0.40	-0.4
Tocochromanol Pathway	GRMZM2G173358	homogentisic acid geranylgeranyl transferase	γT3/(γT3+αT3)	S9_92313446	GBS	9	92,313,446	-170,103	-173,822	3.01E-05	3.69E-02	0.45	0.27	0.49	251	0.18	0.24	-0.06	1.35	-0.04
Tocochromanol Pathway	GRMZM2G173358	homogentisic acid geranylgeranyl transferase	γT3	S9_92313446	GBS	9	92,313,446	-170,103	-173,822	1.24E-05	1.52E-02	0.45	0.27	0.49	250	0.23	0.29	-0.03	0.10	-0.23
Tocochromanol Pathway	GRMZM2G173358	homogentisic acid geranylgeranyl transferase	Total Tocotrienols	S9_92346116	GBS	9	92,346,116	-137,433	-141,152	1.48E-06	9.08E-03	0.41	0.43	0.44	250	0.23	0.30	0.04	0.15	0.27
Tocochromanol Pathway	GRMZM2G173358	homogentisic acid geranylgeranyl transferase	γT3	S9_92346116	GBS	9	92,346,116	-137,433	-141,152	6.82E-07	1.05E-03	0.41	0.43	0.44	250	0.23	0.31	0.03	0.10	0.34
Tocochromanol Pathway	GRMZM2G173358	homogentisic acid geranylgeranyl transferase	Total Tocopherols/Total Tocotrienols	S9_92548696	GBS	9	92,548,696	65,147	61,428	5.84E-06	1.78E-02	0.39	0.38	0.40	247	0.21	0.28	-0.01	0.05	-0.21
Tocochromanol Pathway	GRMZM2G173358	homogentisic acid geranylgeranyl transferase	γT3	S9_92548696	GBS	9	92,548,696	65,147	61,428	5.89E-05	4.52E-02	0.40	0.38	0.40	250	0.23	0.28	0.03	0.10	0.30
Tocochromanol Pathway	GRMZM2G173358	homogentisic acid geranylgeranyl transferase	Total Tocopherols/Total Tocotrienols	S9_92554465	GBS	9	92,554,465	70,916	67,197	1.52E-06	9.26E-03	0.26	0.00	0.32	247	0.21	0.29	-0.01	0.05	-0.25
Tocochromanol Pathway	GRMZM2G173358	homogentisic acid geranylgeranyl transferase	αT3	S9_92553908	GBS	9	92,553,908	70,359	66,640	1.33E-05	2.70E-02	0.43	0.05	0.38	248	0.21	0.28	0.01	0.05	-0.12
Tocochromanol Pathway	GRMZM2G173358	homogentisic acid geranylgeranyl transferase	Total Tocotrienols	S9_92718671	GBS	9	92,718,671	235,122	231,403	6.46E-05	4.95E-02	0.08	0.00	0.13	250	0.23	0.28	-0.05	0.15	-0.3
Tocochromanol Pathway	GRMZM2G173358	homogentisic acid geranylgeranyl transferase	γT3/(γT3+αT3)	S9_92718671	GBS	9	92,718,671	235,122	231,403	2.36E-05	3.69E-02	0.08	0.00	0.13	251	0.18	0.24	-0.10	1.35	-0.07
Tocochromanol Pathway	GRMZM2G173358	homogentisic acid geranylgeranyl transferase	γT3	S9_92718671	GBS	9	92,718,671	235,122	231,403	6.20E-07	1.05E-03	0.08	0.00	0.13	250	0.23	0.31	-0.05	0.10	-0.41
Tocochromanol Pathway	GRMZM2G173358	homogentisic acid geranylgeranyl transferase	Total Tocotrienols	S9_92718674	GBS	9	92,718,674	235,125	231,406	6.46E-05	4.95E-02	0.08	0.00	0.13	250	0.23	0.28	-0.05	0.15	0.36
Tocochromanol Pathway	GRMZM2G173358	homogentisic acid geranylgeranyl transferase	γT3/(γT3+αT3)	S9_92718674	GBS	9	92,718,674	235,125	231,406	2.36E-05	3.69E-02	0.08	0.00	0.13	251	0.18	0.24	-0.10	1.35	-0.07
Tocochromanol Pathway	GRMZM2G173358	homogentisic acid geranylgeranyl transferase	γT3	S9_92718674	GBS	9	92,718,674	235,125	231,406	6.20E-07	1.05E-03	0.08	0.00	0.13	250	0.23	0.31	-0.05	0.10	-0.41
Tocochromanol Pathway	GRMZM2G173358	homogentisic acid geranylgeranyl transferase	Total Tocotrienols	S9_92718709	GBS	9	92,718,709	235,160	231,441	6.46E-05	4.95E-02	0.08	0.00	0.13	250	0.23	0.28	-0.05	0.15	0.36
Tocochromanol Pathway	GRMZM2G173358	homogentisic acid geranylgeranyl transferase	γT3/(γT3+αT3)	S9_92718709	GBS	9	92,718,709	235,160	231,441	2.36E-05	3.69E-02	0.08	0.00	0.13	251	0.18	0.24	-0.10	1.35	-0.07
Tocochromanol Pathway	GRMZM2G173358	homogentisic acid geranylgeranyl transferase	γT3	S9_92718709	GBS	9	92,718,709	235,160	231,441	6.20E-07	1.05E-03	0.08	0.00	0.13	250	0.23	0.31	-0.05	0.10	-0.41

Table S7d Statistically significant results from the candidate gene association study of 20 tocochromanol grain traits with all five SNPs identified in the multi-locus mixed-model (MLMM) analysis included as covariates. SNPs that were significantly associated with the indicated trait at 5% FDR are shown.

a priori candidate gene pathway	RefGen_v2 Gene ID	RefGen_v2 Annotated Gene Function	Trait	SNP ID	SNP Source	Chr	Position	Distance from Gene ORF Start	Distance from Gene ORF Finish	*P*-value	FDR Adjusted *P*-value	Minor Allele Frequency (MAF)	MAF Tropical (18% of 252 Lines)	MAF Temperate (82% of 252 Lines)	Sample Size	R^2_{LR} from Model without SNP	R^2_{LR} from Model with SNP	Effect Size	Lambda from Box-Cox Procedure	Back-Transformed Effect Estimates
Aromatic Head Group	GRMZM2G437912	prephenate dehydratase	Total Tocotrienols	S2_59013838	GBS	2	59,013,838	-23,405	-25,340	1.62E-05	3.25E-02	0.06	0.40	0.09	250	0.23	0.29	0.06	0.15	0.47
Aromatic Head Group	GRMZM2G437912	prephenate dehydratase	Total Tocotrienols	S2_59013840	GBS	2	59,013,840	-23,403	-25,338	1.62E-05	3.25E-02	0.06	0.40	0.09	250	0.23	0.29	-0.06	0.15	-0.34
Tocochromanol Pathway	GRMZM2G173358	homogentisic acid geranylgeranyl transferase	Total Tocotrienols	S9_92346116	GBS	9	92,346,116	-137,433	-141,152	1.75E-06	1.05E-02	0.41	0.43	0.44	250	0.23	0.31	0.04	0.15	0.30
Tocochromanol Pathway	GRMZM2G173358	homogentisic acid geranylgeranyl transferase	γT3/(γT3+αT3)	S9_92718671	GBS	9	92,718,671	235,122	231,403	8.05E-06	1.61E-02	0.08	0.00	0.13	251	0.22	0.29	-0.10	1.35	-0.08
Tocochromanol Pathway	GRMZM2G173358	homogentisic acid geranylgeranyl transferase	γT3/(γT3+αT3)	S9_92718674	GBS	9	92,718,674	235,125	231,406	8.05E-06	1.61E-02	0.08	0.00	0.13	251	0.22	0.29	-0.10	1.35	-0.08
Tocochromanol Pathway	GRMZM2G173358	homogentisic acid geranylgeranyl transferase	γT3/(γT3+αT3)	S9_92718709	GBS	9	92,718,709	235,160	231,441	8.05E-06	1.61E-02	0.08	0.00	0.13	251	0.22	0.29	-0.10	1.35	-0.08
Aromatic Head Group	GRMZM2G437912	prephenate dehydratase	γT3	S2_59013838	GBS	2	59,013,838	-23,405	-25,340	4.99E-05	3.76E-02	0.06	0.40	0.09	250	0.23	0.29	0.04	0.10	0.48
Aromatic Head Group	GRMZM2G437912	prephenate dehydratase	γT3	S2_59013840	GBS	2	59,013,840	-23,403	-25,338	4.99E-05	3.76E-02	0.06	0.40	0.09	250	0.23	0.29	-0.04	0.10	-0.34
Tocochromanol Pathway	GRMZM2G173358	homogentisic acid geranylgeranyl transferase	γT3	S9_92313446	GBS	9	92,313,446	-170,103	-173,822	1.29E-05	1.56E-02	0.45	0.27	0.49	250	0.23	0.29	-0.03	0.10	-0.26
Tocochromanol Pathway	GRMZM2G173358	homogentisic acid geranylgeranyl transferase	γT3	S9_92346116	GBS	9	92,346,116	-137,433	-141,152	4.41E-07	8.50E-04	0.41	0.43	0.44	250	0.23	0.32	0.03	0.10	0.34
Tocochromanol Pathway	GRMZM2G173358	homogentisic acid geranylgeranyl transferase	Total Tocopherols/Total Tocotrienols	S9_92548696	GBS	9	92,548,696	65,147	61,428	1.11E-05	3.33E-02	0.39	0.38	0.40	247	0.22	0.29	-0.01	0.05	-0.21
Tocochromanol Pathway	GRMZM2G173358	homogentisic acid geranylgeranyl transferase	γT3	S9_92548696	GBS	9	92,548,696	65,147	61,428	4.13E-05	3.76E-02	0.40	0.38	0.40	250	0.23	0.29	0.03	0.10	0.34
Tocochromanol Pathway	GRMZM2G173358	homogentisic acid geranylgeranyl transferase	Total Tocopherols/Total Tocotrienols	S9_92554465	GBS	9	92,554,465	70,916	67,197	3.14E-06	1.88E-02	0.26	0.00	0.32	247	0.22	0.30	-0.01	0.05	-0.24
Tocochromanol Pathway	GRMZM2G173358	homogentisic acid geranylgeranyl transferase	γT3	S9_92718671	GBS	9	92,718,671	235,122	231,403	5.65E-07	8.50E-04	0.08	0.00	0.13	250	0.23	0.32	-0.05	0.10	-0.40
Tocochromanol Pathway	GRMZM2G173358	homogentisic acid geranylgeranyl transferase	γT3	S9_92718674	GBS	9	92,718,674	235,125	231,406	5.65E-07	8.50E-04	0.08	0.00	0.13	250	0.23	0.32	-0.05	0.10	-0.40
Tocochromanol Pathway	GRMZM2G173358	homogentisic acid geranylgeranyl transferase	γT3	S9_92718709	GBS	9	92,718,709	235,160	231,441	5.65E-07	8.50E-04	0.08	0.00	0.13	250	0.23	0.32	-0.05	0.10	-0.40
Tocochromanol Pathway	GRMZM2G173358	homogentisic acid geranylgeranyl transferase	γT3	ss196492047	55K	9	92,344,750	-138,799	-142,518	5.80E-05	3.88E-02	0.40	0.50	0.40	250	0.23	0.29	-0.02	0.10	-0.18
